# The global burden of adolescent and young adult cancer in 2019: a systematic analysis for the Global Burden of Disease Study 2019

**DOI:** 10.1016/S1470-2045(21)00581-7

**Published:** 2022-01

**Authors:** GBD 2019 Adolescent Young Adult Cancer Collaborators, Elysia M Alvarez, Lisa M Force, Rixing Xu, Kelly Compton, Dan Lu, Hannah Jacqueline Henrikson, Jonathan M Kocarnik, James D Harvey, Alyssa Pennini, Frances E Dean, Weijia Fu, Martina T Vargas, Theresa H M Keegan, Hany Ariffin, Ronald D Barr, Yana Arturovna Erdomaeva, D Sanjeeva Gunasekera, Yetunde O John-Akinola, Tyler G Ketterl, Tezer Kutluk, Marcio Henrique Malogolowkin, Prashant Mathur, Venkatraman Radhakrishnan, Lynn Ann Gloeckler Ries, Carlos Rodriguez-Galindo, Garik Barisovich Sagoyan, Iyad Sultan, Behzad Abbasi, Mohsen Abbasi-Kangevari, Zeinab Abbasi-Kangevari, Hedayat Abbastabar, Michael Abdelmasseh, Sherief Abd-Elsalam, Amir Abdoli, Haimanot Abebe, Aidin Abedi, Hassan Abidi, Hassan Abolhassani, Hiwa Abubaker Ali, Eman Abu-Gharbieh, Basavaprabhu Achappa, Juan Manuel Acuna, Isaac Akinkunmi Adedeji, Oyelola A Adegboye, Qorinah Estiningtyas Sakilah Adnani, Shailesh M Advani, Muhammad Sohail Afzal, Mohamad Aghaie Meybodi, Bahman Ahadinezhad, Bright Opoku Ahinkorah, Sajjad Ahmad, Sepideh Ahmadi, Muktar Beshir Ahmed, Tarik Ahmed Rashid, Yusra Ahmed Salih, Wajeeha Aiman, Gizachew Taddesse Akalu, Hanadi Al Hamad, Fares Alahdab, Abdulhadi A AlAmodi, Fahad Mashhour Alanezi, Turki M Alanzi, Adugnaw Zeleke Alem, Dejene Tsegaye Alem, Yosef Alemayehu, Fadwa Naji Alhalaiqa, Robert Kaba Alhassan, Saqib Ali, Gianfranco Alicandro, Vahid Alipour, Syed Mohamed Aljunid, Motasem Alkhayyat, Sunitha Alluri, Nihad A Almasri, Sadeq Ali Al-Maweri, Sami Almustanyir, Rajaa M Al-Raddadi, Nelson Alvis-Guzman, Edward Kwabena Ameyaw, Saeed Amini, Hubert Amu, Robert Ancuceanu, Catalina Liliana Andrei, Tudorel Andrei, Fereshteh Ansari, Alireza Ansari-Moghaddam, Davood Anvari, Anayochukwu Edward Anyasodor, Jalal Arabloo, Morteza Arab-Zozani, Ayele Mamo Argaw, Muhammad Arshad, Judie Arulappan, Armin Aryannejad, Zatollah Asemi, Mohammad Asghari Jafarabadi, Mohammad Reza Atashzar, Prince Atorkey, Alok Atreya, Sameh Attia, Avinash Aujayeb, Marcel Ausloos, Leticia Avila-Burgos, Atalel Fentahun Awedew, Beatriz Paulina Ayala Quintanilla, Alemu Degu Ayele, Solomon Shitu Ayen, Mohammed A Azab, Sina Azadnajafabad, Hiva Azami, Mohammadreza Azangou-Khyavy, Amirhossein Azari Jafari, Ghasem Azarian, Ahmed Y Azzam, Saeed Bahadory, Jianjun Bai, Atif Amin Baig, Jennifer L Baker, Maciej Banach, Till Winfried Bärnighausen, Francesco Barone-Adesi, Fabio Barra, Amadou Barrow, Huda Basaleem, Abdul-Monim Mohammad Batiha, Masoud Behzadifar, Niguss Cherie Bekele, Rebuma Belete, Uzma Iqbal Belgaumi, Arielle Wilder Bell, Alemshet Yirga Berhie, Devidas S Bhagat, Akshaya Srikanth Bhagavathula, Nikha Bhardwaj, Pankaj Bhardwaj, Sonu Bhaskar, Krittika Bhattacharyya, Vijayalakshmi S Bhojaraja, Sadia Bibi, Ali Bijani, Antonio Biondi, Setognal Birara, Tone Bjørge, Obasanjo Afolabi Bolarinwa, Srinivasa Rao Bolla, Archith Boloor, Dejana Braithwaite, Hermann Brenner, Norma B Bulamu, Katrin Burkart, Maria Teresa Bustamante-Teixeira, Nadeem Shafique Butt, Zahid A Butt, Florentino Luciano Caetano dos Santos, Chao Cao, Yin Cao, Giulia Carreras, Ferrán Catalá-López, Francieli Cembranel, Ester Cerin, Raja Chandra Chakinala, Promit Ananyo Chakraborty, Vijay Kumar Chattu, Pankaj Chaturvedi, Akhilanand Chaurasia, Prachi P Chavan, Odgerel Chimed-Ochir, Jee-Young Jasmine Choi, Devasahayam J Christopher, Dinh-Toi Chu, Michael T Chung, Joao Conde, Vera Marisa Costa, Omar B Da'ar, Omid Dadras, Saad M A Dahlawi, Xiaochen Dai, Giovanni Damiani, Emanuele D'Amico, Lalit Dandona, Rakhi Dandona, Parnaz Daneshpajouhnejad, Amira Hamed Darwish, Ahmad Daryani, Fernando Pio De la Hoz, Sisay Abebe Debela, Takele Gezahegn G Demie, Getu Debalkie Demissie, Zeleke Geto Demissie, Edgar Denova-Gutiérrez, Meseret Derbew Molla, Rupak Desai, Abebaw Alemayehu Desta, Deepak Dhamnetiya, Samath Dhamminda Dharmaratne, Mandira Lamichhane Dhimal, Meghnath Dhimal, Mostafa Dianatinasab, Mojtaba Didehdar, Mengistie Diress, Shirin Djalalinia, Huyen Phuc Do, Saeid Doaei, Fariba Dorostkar, Wendel Mombaque dos Santos, Thomas M Drake, Michael Ekholuenetale, Iman El Sayed, Maysaa El Sayed Zaki, Maha El Tantawi, Hassan El-Abid, Mostafa Ahmed Elbahnasawy, Iffat Elbarazi, Hala Rashad Elhabashy, Muhammed Elhadi, Shaimaa I El-Jaafary, Daniel Berhanie Enyew, Ryenchindorj Erkhembayar, Babak Eshrati, Sharareh Eskandarieh, Mohammed Faisaluddin, Jawad Fares, Umar Farooque, Abidemi Omolara Fasanmi, Wafa Fatima, José Miguel P Ferreira de Oliveira, Simone Ferrero, Lorenzo Ferro Desideri, Getahun Fetensa, Irina Filip, Florian Fischer, James L Fisher, Masoud Foroutan, Takeshi Fukumoto, Peter Andras Gaal, Mohamed M Gad, Piyada Gaewkhiew, Silvano Gallus, Tushar Garg, Teferi Gebru Gebremeskel, Belete Negese Belete Gemeda, Tamiru Getachew, Mansour Ghafourifard, Seyyed-Hadi Ghamari, Ahmad Ghashghaee, Fariba Ghassemi, Nermin Ghith, Ali Gholami, Jamshid Gholizadeh Navashenaq, Syed Amir Gilani, Themba G Ginindza, Abraham Tamirat Gizaw, James C Glasbey, Amit Goel, Mahaveer Golechha, Pouya Goleij, Davide Golinelli, Sameer Vali Gopalani, Giuseppe Gorini, Houman Goudarzi, Bárbara Niegia Garcia Goulart, Ayman Grada, Mohammed Ibrahim Mohialdeen Gubari, Maximiliano Ribeiro Guerra, Avirup Guha, Bhawna Gupta, Sapna Gupta, Veer Bala Gupta, Vivek Kumar Gupta, Rasool Haddadi, Nima Hafezi-Nejad, Alemayehu Hailu, Arvin Haj-Mirzaian, Rabih Halwani, Randah R Hamadeh, Mitiku Teshome Hambisa, Sajid Hameed, Samer Hamidi, Shafiul Haque, Sanam Hariri, Josep Maria Haro, Ahmed I Hasaballah, S M Mahmudul Hasan, Seyedeh Melika Hashemi, Treska S Hassan, Soheil Hassanipour, Simon I Hay, Khezar Hayat, Sultan H Hebo, Golnaz Heidari, Mohammad Heidari, Brenda Yuliana Herrera-Serna, Claudiu Herteliu, Demisu Zenbaba Heyi, Kamal Hezam, Michael K Hole, Ramesh Holla, Nobuyuki Horita, Md Mahbub Hossain, Mohammad Bellal Hossain, Mohammad-Salar Hosseini, Mostafa Hosseini, Ali Hosseinzadeh, Mehdi Hosseinzadeh, Mihaela Hostiuc, Sorin Hostiuc, Mowafa Househ, Mohamed Hsairi, Junjie Huang, Nawfal R Hussein, Bing-Fang Hwang, Segun Emmanuel Ibitoye, Olayinka Stephen Ilesanmi, Irena M Ilic, Milena D Ilic, Kaire Innos, Lalu Muhammad Irham, Rakibul M Islam, Sheikh Mohammed Shariful Islam, Nahlah Elkudssiah Ismail, Gaetano Isola, Masao Iwagami, Louis Jacob, Farhad Jadidi-Niaragh, Vardhmaan Jain, Mihajlo Jakovljevic, Roksana Janghorban, Amirreza Javadi Mamaghani, Shubha Jayaram, Ranil Jayawardena, Seyed Behzad Jazayeri, Rime Jebai, Ravi Prakash Jha, Tamas Joo, Nitin Joseph, Farahnaz Joukar, Mikk Jürisson, Billingsley Kaambwa, Ali Kabir, Leila R Kalankesh, Feroze Kaliyadan, Zul Kamal, Ashwin Kamath, Himal Kandel, Sitanshu Sekhar Kar, Ibraheem M Karaye, Amirali Karimi, Bekalu Getnet Kassa, Joonas H Kauppila, Phillip M Kemp Bohan, Andre Pascal Kengne, Amene Abebe Kerbo, Mohammad Keykhaei, Yousef Saleh Khader, Himanshu Khajuria, Nastaran Khalili, Neda Khalili, Ejaz Ahmad Khan, Gulfaraz Khan, Maseer Khan, Md Nuruzzaman Khan, Moien AB Khan, Javad Khanali, Maryam Khayamzadeh, Omid Khosravizadeh, Jagdish Khubchandani, Roba Khundkar, Min Seo Kim, Yun Jin Kim, Adnan Kisa, Sezer Kisa, Katarzyna Kissimova-Skarbek, Ali-Asghar Kolahi, Jacek A Kopec, Rajasekaran Koteeswaran, Sindhura Lakshmi Koulmane Laxminarayana, Ai Koyanagi, Nuworza Kugbey, G Anil Kumar, Nithin Kumar, Alexander Kwarteng, Carlo La Vecchia, Qing Lan, Iván Landires, Savita Lasrado, Paolo Lauriola, Caterina Ledda, Sang-woong Lee, Wei-Chen Lee, Yeong Yeh Lee, Yo Han Lee, James Leigh, Elvynna Leong, Bingyu Li, Jiarui Li, Ming-Chieh Li, Stephen S Lim, Xuefeng Liu, Stany W Lobo, Joana A Loureiro, Alessandra Lugo, Raimundas Lunevicius, Hassan Magdy Abd El Razek, Muhammed Magdy Abd El Razek, Morteza Mahmoudi, Azeem Majeed, Alaa Makki, Shilpa Male, Mohammad-Reza Malekpour, Reza Malekzadeh, Ahmad Azam Malik, Mohammed A Mamun, Navid Manafi, Fariborz Mansour-Ghanaei, Borhan Mansouri, Mohammad Ali Mansournia, Santi Martini, Seyedeh Zahra Masoumi, Clara N Matei, Manu Raj Mathur, Colm McAlinden, Ravi Mehrotra, Walter Mendoza, Ritesh G Menezes, Alexios-Fotios A Mentis, Tuomo J Meretoja, Amanual Getnet Mersha, Mohamed Kamal Mesregah, Tomislav Mestrovic, Junmei Miao Jonasson, Bartosz Miazgowski, Irmina Maria Michalek, Ted R Miller, Alemu Basazin Mingude, Seyyedmohammadsadeq Mirmoeeni, Hamed Mirzaei, Sanjeev Misra, Prasanna Mithra, Karzan Abdulmuhsin Mohammad, Mokhtar Mohammadi, Seyyede Momeneh Mohammadi, Abdollah Mohammadian-Hafshejani, Reza Mohammadpourhodki, Arif Mohammed, Shafiu Mohammed, Teroj Abdulrahman Mohammed, Nagabhishek Moka, Ali H Mokdad, Mariam Molokhia, Sara Momtazmanesh, Lorenzo Monasta, Mohammad Ali Moni, Ghobad Moradi, Yousef Moradi, Maliheh Moradzadeh, Rahmatollah Moradzadeh, Paula Moraga, Shane Douglas Morrison, Ebrahim Mostafavi, Amin Mousavi Khaneghah, Christine Mpundu-Kaambwa, Sumaira Mubarik, Lillian Mwanri, Ashraf F Nabhan, Shankar Prasad Nagaraju, Chie Nagata, Mohsen Naghavi, Mukhammad David Naimzada, Luigi Naldi, Vinay Nangia, Atta Abbas Naqvi, Sreenivas Narasimha Swamy, Aparna Ichalangod Narayana, Biswa Prakash Nayak, Vinod C Nayak, Javad Nazari, Sabina Onyinye Nduaguba, Ionut Negoi, Serban Mircea Negru, Seyed Aria Nejadghaderi, Samata Nepal, Sandhya Neupane Kandel, Haruna Asura Nggada, Cuong Tat Nguyen, Chukwudi A Nnaji, Hamed Nosrati, Hasti Nouraei, Ali Nowroozi, Virginia Nuñez-Samudio, Vincent Ebuka Nwatah, Chimezie Igwegbe Nzoputam, Bogdan Oancea, Oluwakemi Ololade Odukoya, Ayodipupo Sikiru Oguntade, In-Hwan Oh, Andrew T Olagunju, Tinuke O Olagunju, Babayemi Oluwaseun Olakunde, Mojisola Morenike Oluwasanu, Emad Omar, Ahmed Omar Bali, Sokking Ong, Obinna E Onwujekwe, Doris V Ortega-Altamirano, Nikita Otstavnov, Stanislav S Otstavnov, Bilcha Oumer, Mayowa O Owolabi, Mahesh P A, Alicia Padron-Monedero, Jagadish Rao Padubidri, Keyvan Pakshir, Adrian Pana, Anamika Pandey, Shahina Pardhan, Fatemeh Pashazadeh Kan, Maja Pasovic, Jenil R Patel, Siddhartha Pati, Sanjay M Pattanshetty, Uttam Paudel, Renato B Pereira, Mario F P Peres, Arokiasamy Perianayagam, Maarten J Postma, Hadi Pourjafar, Akram Pourshams, Akila Prashant, Thejodhar Pulakunta, Mirza Muhammad Fahd Fahd Qadir, Mohammad Rabiee, Navid Rabiee, Amir Radfar, Raghu Anekal Radhakrishnan, Ata Rafiee, Alireza Rafiei, Sima Rafiei, Fakher Rahim, Shadi Rahimzadeh, Mosiur Rahman, Muhammad Aziz Rahman, Amir Masoud Rahmani, Aashish Rajesh, Vajiheh Ramezani-Doroh, Kamal Ranabhat, Priyanga Ranasinghe, Chythra R Rao, Sowmya J Rao, Sina Rashedi, Mahsa Rashidi, Mohammad-Mahdi Rashidi, Goura Kishor Rath, David Laith Rawaf, Salman Rawaf, Lal Rawal, Reza Rawassizadeh, Mohammad Sadegh Razeghinia, Misganu Teshoma Regasa, Andre M N Renzaho, Maryam Rezaei, Negar Rezaei, Nima Rezaei, Mohsen Rezaeian, Aziz Rezapour, Sahba Rezazadeh-Khadem, Abanoub Riad, Ligia Estefania Rios Lopez, Jefferson Antonio Buendia Rodriguez, Luca Ronfani, Gholamreza Roshandel, Godfrey M Rwegerera, Maha Mohamed Saber-Ayad, Siamak Sabour, Basema Saddik, Erfan Sadeghi, Saeid Sadeghian, Umar Saeed, Amirhossein Sahebkar, KM Saif-Ur-Rahman, S Mohammad Sajadi, Sarvenaz Salahi, Sana Salehi, Marwa Rashad Salem, Hamideh Salimzadeh, Abdallah M Samy, Juan Sanabria, Francesco Sanmarchi, Arash Sarveazad, Brijesh Sathian, Monika Sawhney, Susan M Sawyer, Mete Saylan, Ione Jayce Ceola Schneider, Abdul-Aziz Seidu, Mario Šekerija, Endalew Gemechu Sendo, Sadaf G Sepanlou, Allen Seylani, Kenbon Seyoum, Feng Sha, Omid Shafaat, Masood Ali Shaikh, Erfan Shamsoddin, Mohammed Shannawaz, Rajesh Sharma, Sara Sheikhbahaei, Adithi Shetty, B Suresh Kumar Shetty, Pavanchand H Shetty, Jae Il Shin, Reza Shirkoohi, K M Shivakumar, Parnian Shobeiri, Soraya Siabani, Migbar Mekonnen Sibhat, Sudeep K Siddappa Malleshappa, Negussie Boti Sidemo, Diego Augusto Santos Silva, Guilherme Silva Julian, Achintya Dinesh Singh, Jasvinder A Singh, Jitendra Kumar Singh, Surjit Singh, Abiy H Sinke, Yitagesu Sintayehu, Valentin Yurievich Skryabin, Anna Aleksandrovna Skryabina, Lee Smith, Ahmad Sofi-Mahmudi, Mohammad Sadegh Soltani-Zangbar, Suhang Song, Emma Elizabeth Spurlock, Paschalis Steiropoulos, Kurt Straif, Ranjeeta Subedi, Mu'awiyyah Babale Sufiyan, Rizwan Suliankatchi Abdulkader, Saima Sultana, Viktória Szerencsés, Miklós Szócska, Seidamir Pasha Tabaeian, Rafael Tabarés-Seisdedos, Mohammadreza Tabary, Takahiro Tabuchi, Hooman Tadbiri, Majid Taheri, Amir Taherkhani, Ken Takahashi, Mircea Tampa, Ker-Kan Tan, Vivian Y Tat, Ahmad Tavakoli, Abdelghani Tbakhi, Arash Tehrani-Banihashemi, Mohamad-Hani Temsah, Fisaha Haile Tesfay, Bekele Tesfaye, Jarnail Singh Thakur, Rekha Thapar, Aravind Thavamani, Arulmani Thiyagarajan, Nihal Thomas, Ruoyan Tobe-Gai, Munkhsaikhan Togtmol, Seyed Abolfazl Tohidast, Hamid Reza Tohidinik, Musliu Adetola Tolani, Daniel Nigusse Tollosa, Mathilde Touvier, Marcos Roberto Tovani-Palone, Eugenio Traini, Bach Xuan Tran, Mai Thi Ngoc Tran, Jaya Prasad Tripathy, Biruk Shalmeno Tusa, Gebresilasea Gendisha Ukke, Irfan Ullah, Saif Ullah, Krishna Kishore Umapathi, Bhaskaran Unnikrishnan, Era Upadhyay, Tolassa Wakayo Ushula, Marco Vacante, Sahel Valadan Tahbaz, Shoban Babu Varthya, Massimiliano Veroux, Paul J Villeneuve, Francesco S Violante, Vasily Vlassov, Giang Thu Vu, Yasir Waheed, Ning Wang, Paul Ward, Adisu Birhanu Weldesenbet, Yi Feng Wen, Ronny Westerman, Andrea Sylvia Winkler, Befikadu Legesse Wubishet, Suowen Xu, Seyed Hossein Yahyazadeh Jabbari, Lin Yang, Sanni Yaya, Vahid Yazdi-Feyzabadi, Taklo Simeneh Yazie, Sisay Shewasinad Yehualashet, Alex Yeshaneh, Yigizie Yeshaw, Birhanu Wubale Yirdaw, Naohiro Yonemoto, Mustafa Z Younis, Zabihollah Yousefi, Chuanhua Yu, Ismaeel Yunusa, Vesna Zadnik, Mazyar Zahir, Telma Zahirian Moghadam, Mohammad Zamani, Maryam Zamanian, Hamed Zandian, Fariba Zare, Mikhail Sergeevich Zastrozhin, Anasthasia Zastrozhina, Jianrong Zhang, Zhi-Jiang Zhang, Arash Ziapour, Mohammad Zoladl, Christopher J L Murray, Christina Fitzmaurice, Archie Bleyer, Nickhill Bhakta

## Abstract

**Background:**

In estimating the global burden of cancer, adolescents and young adults with cancer are often overlooked, despite being a distinct subgroup with unique epidemiology, clinical care needs, and societal impact. Comprehensive estimates of the global cancer burden in adolescents and young adults (aged 15–39 years) are lacking. To address this gap, we analysed results from the Global Burden of Diseases, Injuries, and Risk Factors Study (GBD) 2019, with a focus on the outcome of disability-adjusted life-years (DALYs), to inform global cancer control measures in adolescents and young adults.

**Methods:**

Using the GBD 2019 methodology, international mortality data were collected from vital registration systems, verbal autopsies, and population-based cancer registry inputs modelled with mortality-to-incidence ratios (MIRs). Incidence was computed with mortality estimates and corresponding MIRs. Prevalence estimates were calculated using modelled survival and multiplied by disability weights to obtain years lived with disability (YLDs). Years of life lost (YLLs) were calculated as age-specific cancer deaths multiplied by the standard life expectancy at the age of death. The main outcome was DALYs (the sum of YLLs and YLDs). Estimates were presented globally and by Socio-demographic Index (SDI) quintiles (countries ranked and divided into five equal SDI groups), and all estimates were presented with corresponding 95% uncertainty intervals (UIs). For this analysis, we used the age range of 15–39 years to define adolescents and young adults.

**Findings:**

There were 1·19 million (95% UI 1·11–1·28) incident cancer cases and 396 000 (370 000–425 000) deaths due to cancer among people aged 15–39 years worldwide in 2019. The highest age-standardised incidence rates occurred in high SDI (59·6 [54·5–65·7] per 100 000 person-years) and high-middle SDI countries (53·2 [48·8–57·9] per 100 000 person-years), while the highest age-standardised mortality rates were in low-middle SDI (14·2 [12·9–15·6] per 100 000 person-years) and middle SDI (13·6 [12·6–14·8] per 100 000 person-years) countries. In 2019, adolescent and young adult cancers contributed 23·5 million (21·9–25·2) DALYs to the global burden of disease, of which 2·7% (1·9–3·6) came from YLDs and 97·3% (96·4–98·1) from YLLs. Cancer was the fourth leading cause of death and tenth leading cause of DALYs in adolescents and young adults globally.

**Interpretation:**

Adolescent and young adult cancers contributed substantially to the overall adolescent and young adult disease burden globally in 2019. These results provide new insights into the distribution and magnitude of the adolescent and young adult cancer burden around the world. With notable differences observed across SDI settings, these estimates can inform global and country-level cancer control efforts.

**Funding:**

Bill & Melinda Gates Foundation, American Lebanese Syrian Associated Charities, St Baldrick's Foundation, and the National Cancer Institute.

## Introduction

Adolescents and young adults represent a heterogenous population consisting of individuals aged 15–39 years.^1^, ^2^, ^3^ This formative time in life is unique, with several physical, emotional, and psychosocial changes, and with individuals potentially beginning or advancing their careers, higher education, relationships, and having children. The definitions and cutoffs of the age range for adolescents and young adults vary, but this age group is generally described as a subpopulation that is in transition between childhood and older adulthood.^1^

Adolescents and young adults develop cancers commonly found and treated in the paediatric population as well as the more common carcinomas seen in adults.^4^, ^5^ Additionally, some cancers are more prevalent in this age group than in younger or older individuals, such as Hodgkin lymphoma and gonadal germ cell tumours.^6^, ^7^ As a consequence, from a health-care delivery perspective, adolescent and young adult patients with cancer might struggle to find care that is optimal for both their cancer type and their age-related treatment needs.^1^ Additionally, adolescent and young adult patients often face social and financial challenges, which might result in inequities in access to appropriate care, timely diagnosis, and treatment.^1^, ^3^, ^8^ Although adolescents and young adults have not seen the same improvements in cancer survival as younger and older cohorts for certain cancers, including acute myeloid leukaemia and soft tissue sarcomas,^9^ this population has not historically been a major focus of cancer control programmes and research development.^10^ Instead, based on historical precedent, adolescents and young adults are often grouped with adult patients in clinical care and clinical trials, and, as a consequence, comprehensive assessments of the cancer burden and epidemiological patterns in this age group are largely unknown or unreported in many settings.^1^

**Research in context**

**Evidence before this study**

Adolescents and young adults with cancer represent a transition population within the cancer continuum between children and older adults. As adolescents and young adults with cancer are treated by a variety of specialists, their unique epidemiology and clinical care needs are often overlooked. Although improvements in survival for children and adults with cancer are reported in high-income countries, less incremental progress has been observed among adolescents and young adults. Added complexities of cancer in this age group include the potential impact of a cancer diagnosis on starting or caring for their families and careers, access to care, diagnostic delays, and abandonment of therapy—issues that exist globally. Previous work assessing the global burden of adolescent and young adult cancer has focused on incidence and mortality, and has occasionally used a more restrictive age range than presented in this study. International adolescent and young adult cancer incidence patterns across time have been reported with data from Cancer Incidence in Five Continents reports, and national-level estimates have been reported from select, primarily high-income, countries. These publications have begun to raise awareness of adolescents and young adults as a distinctive population within the oncology community globally. However, to our knowledge, no previous publication has incorporated the impact of morbidity or done a comparative analysis of cancer within the broader context of the adolescent and young adult disease burden. We searched PubMed for English-language research articles describing the global burden of adolescent and young adult cancers between Jan 1, 2010, and Feb 1, 2021, using the terms “adolescent and young adult or adolescent or young adult or AYA” and “oncology or cancer or neoplasm or tumor or malignancy” and “global or worldwide or international” and “incidence or mortality or morbidity or burden or prevalence or survival”, and identified no additional comprehensive adolescent and young adult global cancer estimate reports.

**Added value of this study**

We share for the first time, the formal global analysis of the cancer burden in individuals aged 15–39 years in 2019, using disability-adjusted life-years (DALYs) estimated by the Global Burden of Diseases, Injuries, and Risk Factors Study (GBD) 2019.GBD 2019 is a valuable global health resource used to inform government health policy decisions around the world when comprehensive data might be absent. The global burden of cancer in terms of mortality and DALYs is substantial in the adolescent and young adult population. The global distribution of the adolescent and young adult cancer burden is unique, reflecting the shift from cancers that primarily affect children (eg, acute lymphoblastic leukaemia) to those that primarily affect adults (eg, carcinomas), and including cancers that occur most often in adolescents and young adults (eg, testicular cancers). Although high Socio-demographic Index (SDI) countries had the highest age-standardised incidence rates, they also had the lowest age-standardised mortality rates when compared to non-high SDI (low, low-middle, middle, and high-middle SDI) countries.

**Implications of all the available evidence**

The relative burden of deaths and DALYs due to adolescent and young adult cancer is high globally, concentrated primarily in non-high SDI settings. These estimates are crucial for comparing the burden of cancer to other causes of deaths and DALYs in adolescents and young adults and might be used to inform health policy and resource allocation priorities. Focus on adolescents and young adults as a distinct cancer population in the development of cancer control programmes is crucial to improving outcomes.

Previous studies have reported on global cancer incidence and mortality patterns of adolescents and young adults.^4^, ^5^, ^11^ One study used incidence and mortality estimates from GLOBOCAN 2012 for individuals aged 20–39 years, another reported incidence and mortality estimates from GLOBOCAN 2018 for individuals aged 15–39 years, and a third study reported international cancer incidence trends in individuals aged 15–39 years using data from the Cancer Incidence in Five Continents series, a publication comprising data from a subset of countries around the world with high-quality population-based cancer registries.^4^, ^5^, ^11^ However, global differences in measures that incorporate both morbidity and mortality due to adolescent and young adult cancers remain unexplored. Consideration of more comprehensive disease burden metrics is especially relevant in adolescents and young adults, whose disease burden might put a strain on their evolving careers and families.^1^, ^12^

The Global Burden of Diseases, Injuries, and Risk Factors Study (GBD) is the only global disease burden estimation framework that provides estimates of disability-adjusted life-years (DALYs) for cancer as a metric to complement incidence and mortality data. DALYs are a key measure of disease burden that include both fatal and non-fatal impacts of disease, and are used in the development of national and global health policy.^13^ GBD estimates disease burden for more than 300 diseases and injuries, allowing for comparative analyses with other causes of morbidity and mortality in adolescents and young adults. To our knowledge, no formal GBD analysis has previously been done of the global burden of cancer in the adolescent and young adult population. In this study, we aimed to analyse and report adolescent and young adult cancer burden estimates, using the most encompassing definition of adolescents and young adults (ie, individuals aged 15–39 years),^2^, ^3^ with a focused analysis on DALY estimates. DALYs represent an important comprehensive assessment of cancer burden in this distinctive population, adding to existing estimates of disease burden with more classic measures, and are crucial to informing cancer control strategies that address health disparities and inequities in this population.

## Methods

### GBD study overview

GBD was established to provide global disease burden metrics that are comprehensive and comparable over time. Estimates produced include incidence, prevalence, mortality, years of life lost (YLLs), years lived with disability (YLDs), and DALYs, measures that can each be used to describe different aspects of the adolescent and young adult cancer burden. Estimates are generated for each disease and injury and are reported by age group, sex, location, and year. Each GBD iteration replaces the previous round of GBD estimates for the entire estimated time series, so that updates to data and methods in the new GBD round are applied consistently across time. The present analysis was based on GBD 2019 estimates.^13^, ^14^ GBD 2019 was done in accordance with the Guideline for Accurate and Transparent Health Estimates Reporting (appendix pp 5, 6).^15^ Data sources used in GBD 2019 are available online and are further outlined in the appendix (p 10). This manuscript was produced as part of the GBD Collaborator Network and in accordance with the GBD Protocol. Analyses were completed with Python (versions 3.6.2 and 3.6.7), Stata (version 13), and R (versions 3.5.0 and 3.4.1).

### Definitions

Although the definition of the age range for adolescents and young adults varies, particularly in the upper age limit,^12^, ^16^, ^17^ we used the age range of 15–39 years in this study, since this is the most encompassing age range definition recommended in oncology, is endorsed by the US National Cancer Institute and the AYA Working Group of the European Society for Medical Oncology and the European Society for Paediatric Oncology,^3^ and allows for comparability with other studies on adolescent and young adult cancer.^1^ Individuals aged 15–39 years have also experienced the least progress in survival outcomes in most countries.^11^ Data for this age range are available online with the GBD Results Tool and for subsets of this age range with the GBD Compare data visualisation tool or GBD Results Tool. As there are differences in the preferred age range used to define adolescents and young adults around the world, results of the narrower age range of 15–29 years are presented in the appendix (pp 115–122).

All malignant cancer types, as defined in the tenth revision of the International Classification of Diseases, chapter II (Neoplasms),^18^ were categorised into 32 cancer groups in this analysis, called causes in GBD and this Article. Non-melanoma skin cancers were excluded, since they are not a major cause of mortality in this age range. The cause “other malignant neoplasms” in GBD includes estimates for cancers not included in any other GBD cancer cause, such as bone cancers and soft tissue sarcomas (see appendix p 11 for more details about cancer mapping). The adolescent and young adult age group was compared to children (aged 0–14 years) and older adults (aged ≥40 years) in specific analyses. The focus of this analysis was on global and regional estimates, although GBD 2019 also produces estimates at the national and, for select countries, subnational level. National and subnational estimates are available in the GBD Compare and GBD Results tools online. Select results are presented by quintiles of the Socio-demographic Index (SDI; countries ranked and divided into five equal SDI groups), which is a composite measure of income per capita, total fertility rate (age <25 years), and average educational fulfilment (for those aged ≥15 years), and is a useful summary measure of a country's overall social and economic development that allows for analyses of disease burden patterns across different resource contexts (appendix p 56).^14^ All cancer rates were reported per 100 000 person-years. The GBD world population standard was used for the calculation of age-standardised rates (appendix p 56).

### Estimation of cancer burden

The GBD cancer estimation process begins with a focus on mortality. Data sources include vital registration systems, verbal autopsies, and population-based cancer registration systems. Some cancer registries report incidence only; therefore, mortality-to-incidence ratios (MIRs) were used to convert cancer registry incidence data to estimates of mortality, increasing data availability in locations that might not have mortality data, but have active cancer registries. Using a spatiotemporal Gaussian process regression, MIRs were modelled for all combinations of age, sex, year, and location with incidence data from cancer registries and mortality data from cancer registries or high-quality vital statistics registries (elaborated in the appendix pp 25, 26).^13^ Estimates of mortality obtained with MIRs were combined with vital registration and verbal autopsy mortality data and used as inputs in cancer type and sex-specific Cause of Death Ensemble models (CODEm).^19^ The CODEm methodology uses all available mortality data to select the optimal model or models on the basis of out-of-sample predictive validity (appendix p 28). Cause-specific mortality estimates were then scaled to independently modelled all-cause mortality with CoDCorrect to ensure consistency.^13^

Incidence estimates were obtained by dividing the mortality estimates by the corresponding MIR for each cancer type. Survival estimates based on MIRs were used to model 10-year prevalence for each cancer cause (appendix pp 50, 51). Prevalence for each cancer cause was divided into distinct phases of cancer treatment to estimate YLDs. For cohorts that survived beyond 10 years from diagnosis, two phases were estimated for the 10-year time period after diagnosis: diagnosis or treatment; and remission. After the 10-year period, the disability risk was returned to the baseline of the general population without a cancer diagnosis. For cohorts that did not survive beyond 10 years from diagnosis, two additional phases were estimated: the metastatic or disseminated phase; and the terminal phase. YLD estimates were generated by multiplying each phase prevalence by a phase-specific disability weight, representative of the health loss magnitude associated with a specified health outcome. Disability weights are measured on a scale of 0 (full health) to 1 (equivalent to death; appendix p 55). YLLs were calculated as the standard life expectancy at the age of death multiplied by age-specific cancer deaths.^14^ DALY estimates were the sum of the YLD and YLL estimates. Proportional DALYs for each cancer cause and 5-year age group were calculated as the mean of 1000 proportion draws of the absolute number of DALYs for each cancer cause and age group divided by the total number of cancer DALYs within the same age group. Proportional DALYs for each SDI were calculated as the mean of 1000 proportion draws of the absolute number of DALYs for each cancer cause within each SDI quintile and divided by the total number of DALYs in each SDI quintile (appendix p 56). An additional analysis was done to identify the proportion of adolescent and young adult cancer cases covered by the WHO Global Initiative for Childhood Cancer (appendix p 56). Further detailed descriptions of the methods are provided in the appendix (pp 7–57) and in GBD 2019 summary publications.^13^, ^14^

### Uncertainty analysis

Final point estimates are reported with 95% uncertainty intervals (UIs). 95% UIs are 95% ranges calculated as the range from the 2·5th to the 97·5th percentile on the basis of the distribution of 1000 draws at each GBD cancer estimation step, with uncertainty propagated through each step (appendix p 57).

### Role of the funding source

The funders of this study had no role in the design of the GBD cancer estimation process, collection or analysis of data, interpretation of results, or in the writing of this manuscript.

## Results

There were an estimated 1·19 million (95% UI 1·11–1·28) incident cancer cases and 396 000 (370 000–425 000) deaths among individuals aged 15–39 years worldwide in 2019 (table). The highest age-standardised incidence rates were seen in high SDI (59·6 [54·5–65·7] per 100 000 person-years) and high-middle SDI (53·2 [48·8–57·9] per 100 000 person-years) countries, while the highest age-standardised mortality rates from cancer in adolescents and young adults were seen in middle SDI (13·6 [12·6–14·8] per 100 000 person-years) and low-middle SDI (14·2 [12·9–15·6] per 100 000 person-years) regions. Adolescent and young adult cancers contributed 23·5 million (21·9–25·2) DALYs to the global burden of disease in 2019 (table), of which 2·7% (1·9–3·6) came from YLDs and 97·3% (96·4–98·1) from YLLs (appendix p 79). The majority (91·4% [91·0–91·8]) of the worldwide absolute adolescent and young adult cancer DALY burden is concentrated in non-high SDI (low, low-middle, middle, and high-middle SDI) quintiles. Overall, high SDI settings have the highest age-standardised incidence rate (59·6 [54·5–65·7] per 100 000 person-years), but the lowest age-standardised DALY rate (564·3 [542·8–590·1] per 100 000 person-years). Breast cancer (10·6% [10·0–11·2]), followed by brain and CNS cancer (7·4% [6·0–8·0]), colon and rectum cancer (7·0% [6·6–7·3]), and stomach cancer (6·7% [6·5–7·0]) were the four greatest contributors to the DALY burden globally for both sexes combined, of separately categorised cancers (appendix p 81). If leukaemias were considered as a single group, given that they are treated by haematologist-oncologists and have a similar diagnostic approach, rather than as individual leukaemia subtypes, leukaemias would be the largest categorised cancer group contributing to the global cancer DALY burden (12·0% [10·9–12·8]), greater than that of breast cancer. The “other malignant neoplasms” category, the aggregated cancer cause category for cancers not separately estimated in the GBD framework, comprised the highest proportion of the adolescent and young adult cancer DALY burden globally (13·7% [12·8–14·5]; appendix p 81). A focused analysis of individuals aged 15–29 years is provided in the appendix (pp 115–122).

**Table:** Adolescent and young adult cancer burden globally and by SDI quintile in 2019

		**DALYs, thousands (95% UI)**	**Age-standardised DALY rate per 100 000 (95% UI)**	**Incidence, thousands (95% UI)**	**Age-standardised incidence rate per 100 000 (95% UI)**	**Mortality, thousands (95% UI)**	**Age-standardised mortality rate per 100 000 (95% UI)**
Global		23 500 (21 900–25 200)	782·2 (730·8–838·1)	1190 (1110–1280)	39·7 (36·9–42·6)	396 (370–425)	13·2 (12·3–14·1)
SDI quintiles							
	High SDI quintile	2020 (1940–2110)	564·3 (542·8–590·1)	213 (195–235)	59·6 (54·5–65·7)	33·4 (32·2–34·7)	9·2 (8·9–9·6)
	High-middle SDI quintile	4520 (4200–4840)	801·4 (745·8–857·8)	302 (277–329)	53·2 (48·8–57·9)	76·6 (71·1–82·2)	13·4 (12·4–14·3)
	Middle SDI quintile	7780 (7190–8410)	810·1 (748·4–876·6)	369 (339–401)	38·3 (35·1–41·6)	132 (122–143)	13·6 (12·6–14·8)
	Low-middle SDI quintile	5970 (5420–6530)	836·7 (760·1–915·5)	209 (188–229)	29·4 (26·5–32·2)	101 (91·4–110)	14·2 (12·9–15·6)
	Low SDI quintile	3190 (2770–3630)	781·1 (678·2–890·0)	101 (86·3–115)	25·0 (21·4–28·7)	53·5 (46·5–60·9)	13·3 (11·6–15·2)
Cancers							
	Breast cancer	2490 (2260–2720)	82·1 (74·4–89·8)	170 (154–186)	5·6 (5·1–6·1)	43·1 (39·1–47·3)	1·4 (1·3–1·6)
	Brain and CNS cancer	1750 (1380–1940)	58·4 (46·2–64·9)	61·5 (48·2–69·1)	2·1 (1·6–2·3)	29·1 (23·0–32·3)	1·0 (0·8–1·1)
	Colon and rectum cancer	1630 (1510–1760)	53·9 (49·9–58·1)	76·1 (70·2–82·9)	2·5 (2·3–2·7)	28·4 (26·2–30·5)	0·9 (0·9–1·0)
	Stomach cancer	1570 (1450–1700)	52·0 (47·8–56·2)	49·0 (45·0–53·1)	1·6 (1·5–1·8)	27·9 (25·7–30·2)	0·9 (0·8–1·0)
	Cervical cancer	1560 (1 320–1780)	51·4 (43·5–58·7)	119 (99·6–135)	3·9 (3·3–4·5)	27·2 (22·9–31·1)	0·9 (0·8–1·0)
	Tracheal, bronchus, and lung cancer	1390 (1270–1510)	45·8 (42·0–50·0)	32·6 (29·7–35·5)	1·1 (1·0–1·2)	24·8 (22·7–27·0)	0·8 (0·7–0·9)
	Non-Hodgkin lymphoma	1280 (1190–1380)	42·8 (39·8–46·4)	52·4 (47·0–58·7)	1·8 (1·6–2·0)	20·8 (19·3–22·6)	0·7 (0·6–0·8)
	Liver cancer	1050 (938–1160)	34·6 (31·0–38·4)	25·4 (22·7–28·4)	0·8 (0·8–0·9)	18·6 (16·6–20·7)	0·6 (0·5–0·7)
	Other leukaemia	949 (791–1080)	32·0 (26·6–36·4)	28·8 (23·8–32·8)	1·0 (0·8–1·1)	15·3 (12·7–17·4)	0·5 (0·4–0·6)
	Acute lymphoid leukaemia	766 (634–844)	26·1 (21·7–28·8)	38·7 (32·2–43·2)	1·3 (1·1–1·5)	11·7 (9·64–12·9)	0·4 (0·3–0·4)
	Acute myeloid leukaemia	748 (678–858)	25·2 (22·9–28·9)	20·2 (18·2–22·8)	0·7 (0·6–0·8)	12·2 (11·0–14·0)	0·4 (0·4–0·5)
	Lip and oral cavity cancer	580 (520–644)	19·2 (17·2–21·3)	29·4 (26·3–32·7)	1·0 (0·9–1·1)	10·0 (9·00–11·1)	0·3 (0·3–0·4)
	Ovarian cancer	529 (443–602)	17·6 (14·7–20·0)	35·8 (30·5–41·0)	1·2 (1·0–1·4)	8·90 (7·46–10·1)	0·3 (0·2–0·3)
	Hodgkin lymphoma	508 (432–600)	17·1 (14·5–20·2)	33·4 (29·9–40·5)	1·1 (1·0–1·4)	8·09 (6·85–9·52)	0·3 (0·2–0·3)
	Pancreatic cancer	421 (387–463)	13·9 (12·8–15·3)	9·40 (8·59–10·3)	0·3 (0·3–0·3)	7·61 (6·98–8·39)	0·3 (0·2–0·3)
	Nasopharynx cancer	363 (334–394)	12·1 (11·1–13·1)	28·6 (25·3–32·3)	0·9 (0·8–1·1)	6·08 (5·60–6·65)	0·2 (0·2–0·2)
	Testicular cancer	349 (319–383)	11·7 (10·6–12·8)	57·4 (51·6–65·1)	1·9 (1·7–2·2)	5·35 (4·92–5·84)	0·2 (0·2–0·2)
	Oesophageal cancer	344 (308–382)	11·3 (10·1–12·6)	8·09 (7·27–8·97)	0·3 (0·2–0·3)	6·21 (5·57–6·90)	0·2 (0·2–0·2)
	Chronic myeloid leukaemia	295 (261–335)	9·8 (8·7–11·2)	9·20 (8·34–10·2)	0·3 (0·3–0·3)	4·96 (4·39–5·62)	0·2 (0·1–0·2)
	Malignant skin melanoma	259 (216–318)	8·6 (7·2–10·5)	37·3 (30·5–46·4)	1·2 (1·0–1·5)	4·25 (3·55–5·21)	0·1 (0·1–0·2)
	Other pharynx cancer	245 (211–276)	8·1 (7·0–9·1)	7·10 (6·26–7·92)	0·2 (0·2–0·3)	4·36 (3·76–4·91)	0·1 (0·1–0·2)
	Kidney cancer	239 (220–264)	7·9 (7·3–8·8)	21·1 (19·3–23·3)	0·7 (0·6–0·8)	4·02 (3·69–4·43)	0·1 (0·1–0·1)
	Thyroid cancer	191 (168–214)	6·4 (5·6–7·1)	46·8 (40·6–51·7)	1·6 (1·3–1·7)	2·85 (2·52–3·17)	0·1 (0·1–0·1)
	Gallbladder and biliary tract cancer	133 (113–147)	4·4 (3·7–4·9)	3·84 (3·29–4·26)	0·1 (0·1–0·1)	2·39 (2·03–2·66)	0·1 (0·1–0·1)
	Larynx cancer	128 (118–140)	4·2 (3·9–4·6)	4·21 (3·88–4·58)	0·1 (0·1–0·2)	2·25 (2·06–2·47)	0·1 (0·1–0·1)
	Bladder cancer	124 (113–137)	4·1 (3·7–4·5)	14·1 (12·6–15·8)	0·5 (0·4–0·5)	2·05 (1·85–2·28)	0·1 (0·1–0·1)
	Uterine cancer	110 (85·1–124)	3·6 (2·8–4·1)	19·4 (15·8–22·0)	0·6 (0·5–0·7)	1·81 (1·39–2·04)	0·1 (0·0–0·1)
	Multiple myeloma	95·6 (74·3–107)	3·2 (2·5–3·5)	2·93 (2·26–3·34)	0·1 (0·1–0·1)	1·68 (1·31–1·88)	0·1 (0·0–0·1)
	Chronic lymphoid leukaemia	61·4 (52·8–69·4)	2·0 (1·8–2·3)	4·26 (3·65–4·88)	0·1 (0·1–0·2)	1·01 (0·872–1·14)	0·0 (0·0–0·0)
	Mesothelioma	56·4 (44·1–67·9)	1·9 (1·5–2·2)	1·47 (1·15–1·79)	0·0 (0·0–0·1)	0·990 (0·777–1·20)	0·0 (0·0–0·0)
	Prostate cancer	54·3 (47·2–66·1)	1·8 (1·6–2·2)	5·47 (4·78–6·55)	0·2 (0·2–0·2)	0·876 (0·757–1·06)	0·0 (0·0–0·0)
	Other malignant neoplasms	3230 (2920–3530)	109·1 (98·8–119·4)	141 (130–154)	4·8 (4·4–5·2)	51·5 (46·6–56·2)	1·7 (1·6–1·9)

Estimates are for individuals aged 15–39 years, both sexes combined. Values in parentheses are 95% uncertainty intervals (UIs). Rates are reported per 100 000 person-years. Cancer types are listed in order of global DALY burden, with the exception of “other malignant neoplasms”, which are listed last. Other malignant neoplasms are cancers without a detailed GBD cause separately listed. Other leukaemia included leukaemias not otherwise specified. Non-melanoma skin cancers were not included in this analysis. SDI categories do not sum precisely to the global total as GBD 2019 does not provide separate estimates for all locations globally and an adjustment factor is made between all estimated locations, which have corresponding SDI values, and the global estimate. DALYs=disability-adjusted life years. UI=uncertainty interval. GBD=Global Burden of Diseases, Injuries, and Risk Factors Study. SDI=Socio-demographic Index.

**Figure 1:**
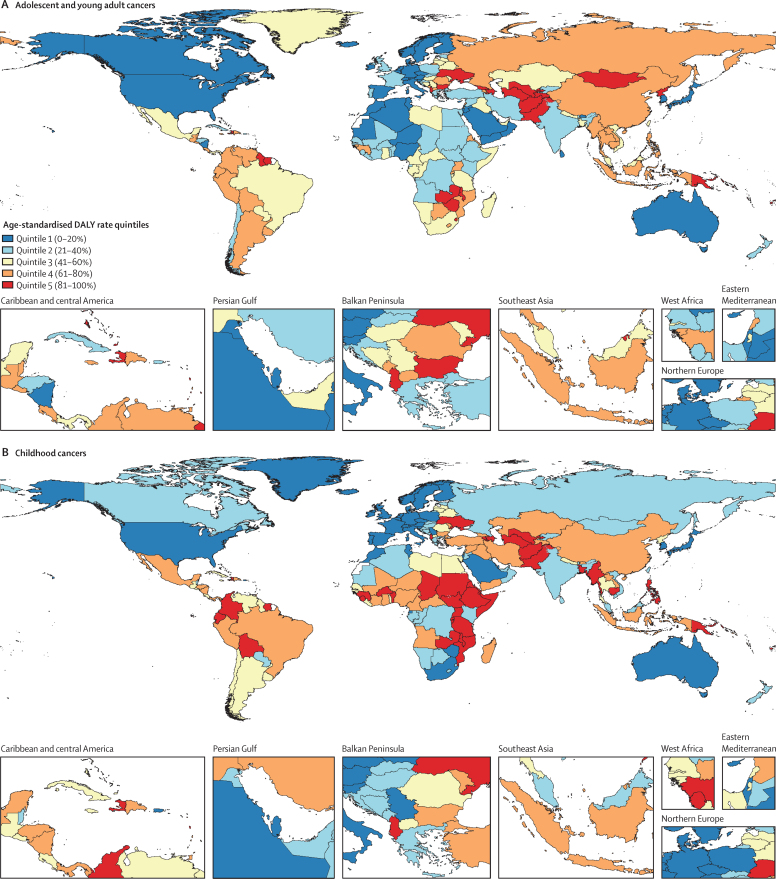

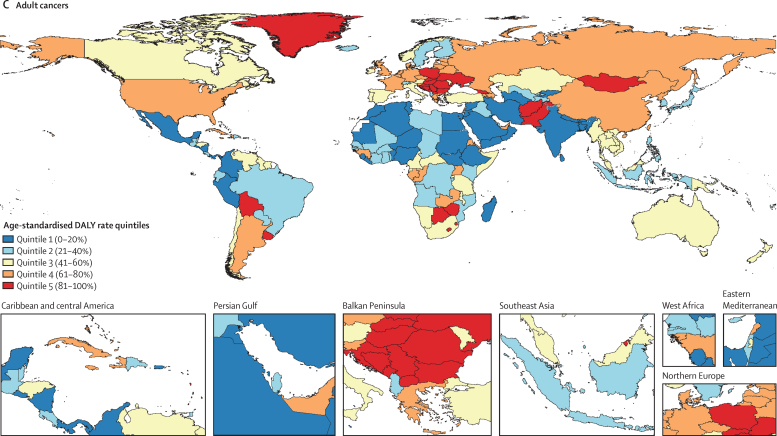
Global map of age-standardised DALY rates for both sexes combined in 2019, categorised by age-standardised DALY rate quintiles, excluding non-melanoma skin cancers, for malignant adolescent and young adult cancers (A), malignant childhood cancers (B), and malignant adult cancers (C) Quintiles are based on age-standardised DALY rates per 100 000 person-years. For adolescent and young adult cancers (age 15–39 years), quintile 1 (0–20%) corresponds to less than 597 DALYs per 100 000 person-years, quintile 2 (21–40%) corresponds to 597 to less than 729 DALYs per 100 000 person-years, quintile 3 (41–60%) corresponds to 729 to less than 833 DALYs per 100 000 person-years, quintile 4 (61–80%) corresponds to 833 to less than 1010 DALYs per 100 000 person-years, and quintile 5 (81–100%) corresponds to 1010 or more DALYs per 100 000 person-years. For childhood cancers (age 0–14 years), quintile 1 (0–20%) corresponds to less than 250 DALYs per 100 000 person-years, quintile 2 (21–40%) corresponds to 250 to less than 311 DALYs per 100 000 person-years, quintile 3 (41–60%) corresponds to 311 to less than 396 DALYs per 100 000 person-years, quintile 4 (61–80%) corresponds to 396 to less than 495 DALYs per 100 000 person-years, and quintile 5 (81–100%) corresponds to 495 or more DALYs per 100 000 person-years. For adult cancers (age ≥40 years), quintile 1 (0–20%) corresponds to less than 6680 DALYs per 100 000 person-years, quintile 2 (21–40%) corresponds to 6680 to less than 7390 DALYs per 100 000 person-years, quintile 3 (41–60%) corresponds to 7390 to less than 8580 DALYs per 100 000 person-years, quintile 4 (61–80%) corresponds to 8580 to less than 9890 DALYs per 100 000 person-years, and quintile 5 (81–100%) corresponds to 9890 or more DALYs per 100 000 person-years. There are several geographical locations (shown in white) where estimates are not available (eg, Western Sahara and French Guiana) as they were not modelled locations in GBD 2019. DALY=disability-adjusted life-year. GBD=Global Burden of Diseases, Injuries, and Risk Factors Study.

The greatest burden of cancer in adolescents and young adults in 2019, as represented by age-standardised DALY rates, was concentrated in parts of Asia, southern sub-Saharan Africa, and South America (figure 1A; appendix p 84). The distribution of DALYs due to cancer in adolescents and young adults is distinct from that of children (figure 1B) and older adults (figure 1C). The geographical pattern of age-standardised DALY rate quintiles for adolescent and young adult cancer was similar to the geographical pattern of childhood cancers in high SDI countries and resembled the distribution of adult cancer in low and middle SDI countries (figure 1).

**Figure 2:**
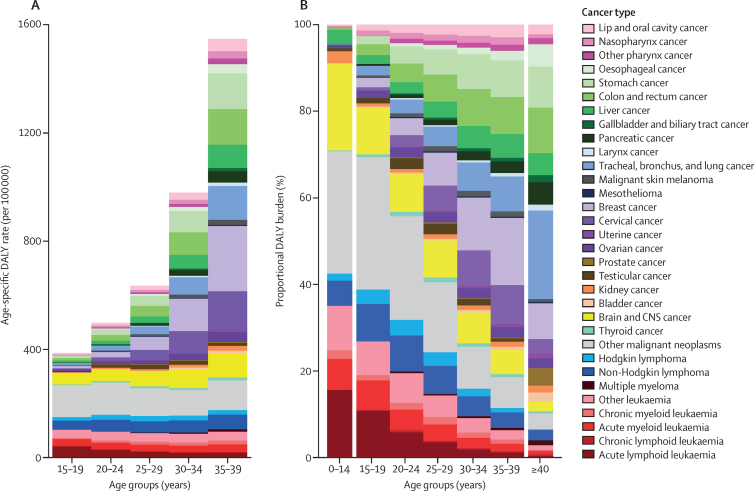
Global age-specific DALY rates (A) and proportional DALY burden (B) of adolescent and young adult cancer types by age group, in 2019, for both sexes combined Rates are expressed per 100 000 person-years. “Other malignant neoplasms” comprise all malignancies without a separate GBD cause category listed; this category does not include non-melanoma skin cancers and myelodysplastic or myeloproliferative neoplasms, which are separate GBD cause categories not included in this analysis. Other leukaemia included leukaemias not otherwise specified. DALY=disability-adjusted life-year. GBD=Global Burden of Diseases, Injuries, and Risk Factors Study.

Of all age groups, individuals aged 35–39 years had the largest contribution to the adolescent and young adult global cancer DALYs (8·4 million [95% UI 7·8–9·0], with corresponding DALY rates of 1547·6 [1441·3–1658·0] per 100 000 person-years; figure 2A). The proportion of DALYs attributed to leukaemias declined with increasing age across the adolescent and young adult population (26·7% [24·8–28·8] of total age group DALYs, corresponding to 0·64 million [0·56–0·72] DALYs in those aged 15–19 years *vs* 6·2% [5·6–6·7] of total age group DALYs, corresponding to 0·52 million [0·46–0·58] DALYs in those aged 35–39 years; appendix p 107). The proportion of DALYs attributed to carcinomas increased with increasing age across the adolescent and young adult population (18·1% [17·3–19·3] of total age group DALYs in those aged 15–19 years, corresponding to 0·43 million [0·40–0·47] DALYs *vs* 73·6% [72·7–75·2] of total age group DALYs, corresponding to 6·2 million [5·7–6·6] DALYs in those aged 35–39 years; figure 2B; appendix p 107). There was a notable proportion of “other malignant neoplasms” across the adolescent and young adult population, which was highest in those aged 15–19 years (30·6% [28·6–32·2] of total age group DALYs, corresponding to 0·73 million [0·65–0·81] DALYs), and lowest in those aged 35–39 years (7·1% [6·5–7·5] of total age group DALYs, corresponding to 0·59 million [0·54–0·65] DALYs; figure 2B). In direct comparisons of the proportional DALY burden for the 15–29-year age group with that of the 30–39-year age group, there is a transition in the predominant cause from leukaemias and lymphomas to carcinomas, especially breast and cervical cancer (appendix p 122).

**Figure 3:**
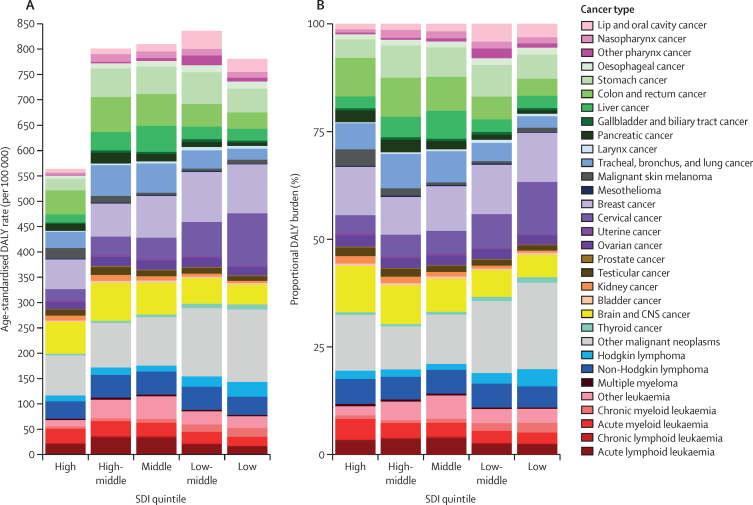
Age-standardised DALY rates (A) and proportional adolescent and young adult cancer DALY burden (B) by Socio-demographic Index, in 2019, for both sexes combined Rates are expressed per 100 000 person-years. “Other malignant neoplasms” comprise all malignancies without a separate GBD cause category listed; this category does not include non-melanoma skin cancers and myelodysplastic or myeloproliferative neoplasms, which are separate GBD cause categories not included in this analysis. Other leukaemia included leukaemias not otherwise specified. DALY=disability-adjusted life-year. GBD=Global Burden of Diseases, Injuries, and Risk Factors Study. SDI=Socio-demographic Index.

When assessed by SDI quintile, age-standardised DALY rates and the proportional DALY burden varied by cancer type (figure 3). Individuals in the high SDI quintile had a lower age-standardised DALY rate (564·3 [95% UI 542·8–590·1] DALYs per 100 000 person-years; figure 3A) than other SDI quintiles. Estimates of the proportion of the DALY burden due to cervical cancer increased with decreasing SDI quintile, having the lowest proportional burden in the high SDI setting (4·1% [3·7–4·4]; figure 3B) and the highest burden in the low SDI setting (12·1% [10·4–14·4]; figure 3B). The adolescent and young adult cancer burden attributed to brain and CNS cancer was highest in the high SDI (10·7% [8·8–11·6]) and high-middle SDI (9·0% [7·2–9·7]) quintiles, compared to the low-middle SDI (6·1% [5·0–6·9]) and low SDI (5·2% [4·0–6·2]) quintiles. The proportion of adolescent and young adult cancers that were in the “other malignant neoplasms” category was highest in the low SDI quintile (20·1% [18·7–22·2]) and lowest in the high-middle SDI quintile (9·9% [9·5–10·6]).

**Figure 4:**
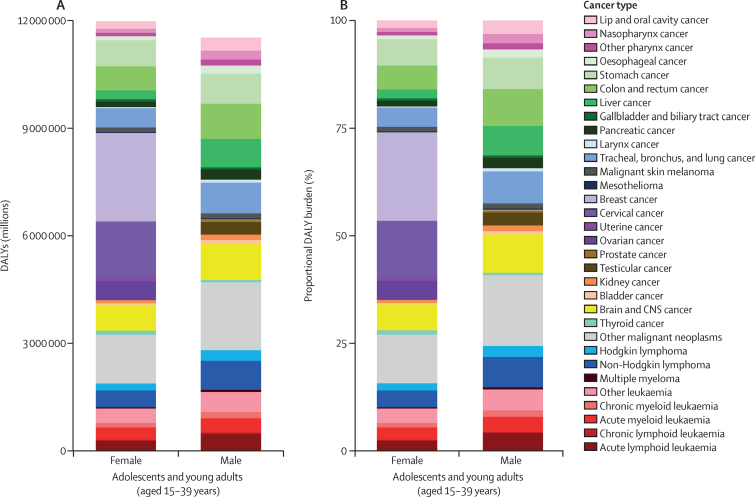
Global absolute (A) and proportional (B) adolescent and young adult cancer DALY burden by sex in 2019 Other malignant neoplasms comprise all malignancies without a separate GBD cause category listed; this category does not include non-melanoma skin cancers and myelodysplastic or myeloproliferative neoplasms, which are separate GBD cause categories not included in this analysis. Other leukaemia included leukaemias not otherwise specified. DALY=disability-adjusted life-year. GBD=Global Burden of Diseases, Injuries, and Risk Factors Study.

The top five causes by absolute DALY burden in females globally in 2019 were breast cancer (2·46 million [95% UI 2·23–2·70] DALYs), cervical cancer (1·56 million [1·32–1·78] DALYs), “other malignant neoplasms” (1·35 million [1·21–1·51] DALYs), stomach cancer (732 000 [653 000–814 000] DALYs), and brain and CNS cancer (722 000 [536 000–827 000] DALYs; figure 4; appendix pp 66–69). The five cancers with the highest absolute DALY burden in males were “other malignant neoplasms” (1·88 million [1·64–2·12] DALYs); brain and CNS cancer (1·03 million [0·76–1·19] DALYs); colon and rectum cancer (973 000 [887 000–1 070 000] DALYs); tracheal, bronchus, and lung cancer (856 000 [766 000–952 000] DALYs); and stomach cancer (842 000 [767 000–928 000] DALYs; figure 4; appendix pp 62–65). In 2019, females had a higher overall incidence of cancer than males globally (686 000 [622 000–751 000] *vs* 509 000 [469 000–549 000] incident cancer cases), but had similar absolute mortality (202 000 [184 000–222 000] *vs* 194 000 [179 000–209 000] deaths; appendix pp 62, 66, 82–83). Breast and cervical cancer combined made up a substantial proportion of the DALY burden globally in females (33·6% [32·3–35·1]). Among the non-sex-specific cancer causes, males had higher absolute DALYs globally in 24 of 27 cancer groups, representing a 13·7% (3·5–25·1) overall higher absolute number of DALYs than females.

**Figure 5:**
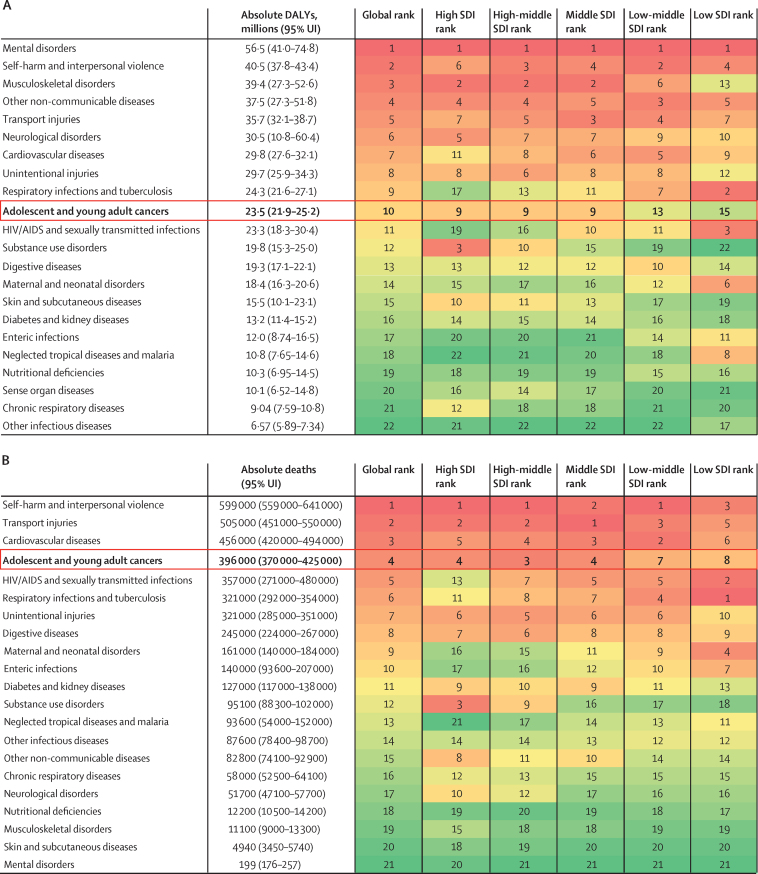
Ranking of absolute DALYs (A) and deaths (B) due to cancer compared to other disease groups in adolescents and young adults in 2019, for both sexes combined, globally and by SDI Disease rank assigned by total absolute DALYs (A) or absolute deaths (B) globally in 2019 in the adolescent and young adult age group (15–39 years), with 1 representing the highest rank. Values in parentheses are 95% uncertainty intervals (UIs). Colour intensity is proportional to rank number (from 1 denoted by dark red to 22 [or 21 in panel B] denoted by dark green). Cancers comprise all malignant neoplasms, excluding non-melanoma skin cancers. Panels A and B included different causes because some causes do not have mortality estimated in this age range. Other non-communicable diseases comprise congenital birth defects; urinary diseases and male infertility; gynaecological diseases; haemoglobinopathies and haemolytic anaemias; endocrine, metabolic, blood, and immune disorders; and oral disorders. Other infectious diseases comprise meningitis, encephalitis, diphtheria, whooping cough, tetanus, measles, varicella and herpes zoster, acute hepatitis, and other unspecified infectious diseases. DALY=disability-adjusted life-year. SDI=Socio-demographic Index.

Rankings of the burden of absolute DALYs and deaths due to adolescent and young adult cancer compared to other diseases in individuals aged 15–39 years, both globally and by SDI quintile, are shown in figure 5. Adolescent and young adult cancer had the tenth highest DALY burden globally (23·5 million [95% UI 21·9–25·2] DALYs; figure 5A) among 22 causes of DALYs at this level in the GBD hierarchy. The inter-category rankings show that cancer ranks higher than other prominent causes of DALYs in high, high-middle, and middle SDI quintiles, compared to low-middle and low SDI quintiles. In adolescents and young adults, deaths from cancer ranked fourth globally (396 000 [370 000–425 000]; figure 5B), among 21 causes of death at this level in the GBD hierarchy, with a higher intra-SDI-quintile ranking in high, high-middle, and middle SDI regions, compared to low-middle and low SDI regions. In comparison, deaths due to cancer ranked 11th globally in those younger than 15 years and second in those older than 39 years (appendix pp 105, 106). In 2019, deaths due to cancer in the adolescent and young adult population were lower than those estimated for transport injuries and cardiovascular and circulatory diseases, but higher than those estimated for HIV/AIDS and sexually transmitted infections, respiratory infections and tuberculosis, and unintentional injuries (figure 5B). More detailed findings are summarised in the appendix (pp 62–114). An additional analysis showed that 8·6% (95% UI 8·2–9·1) of all adolescent and young adult cancer cases are included in the WHO Global Initiative for Childhood Cancer (appendix p 56).

## Discussion

In our analysis of adolescent and young adult cancer, based on data from GBD 2019, we show, to the best of our knowledge, for the first time that the global burden of adolescent and young adult cancer is substantial in terms of DALYs, a measure that is frequently used by governments to inform policy and resource allocation needs. From a descriptive perspective, the age-standardised distribution of adolescent and young adult DALYs was unique compared to both childhood and adult cancers, reflecting an expected but ill-described transition from childhood to adult cancer epidemiological patterns.^20^, ^21^, ^22^ Additionally, when the overall disease burden is studied cross-sectionally within the age range encompassing adolescents and young adults, the global burden of cancer contributed more DALYs to the global disease burden than some high-profile communicable diseases such as HIV/AIDS and sexually transmitted infections. This comparison of cancer with other leading causes of global mortality and DALYs in adolescents and young adults has not been previously documented. These results highlight that cancer is an important contributor to premature death and the disease burden in adolescents and young adults globally, even when compared with some communicable diseases that that are the focus of more active global funding, research, and advocacy efforts.^1^, ^23^ The findings also underscore the need to develop a global strategy to address the cancer burden in this population, which should include the integration of adolescent and young adult cancer into overall cancer control planning and universal health coverage plans.^24^

Because of the substantial burden of adolescent and young adult cancers globally, with the majority of DALYs occurring on the lower end of the SDI spectrum, broader attention to the unique determinants driving cancer outcomes in this age range is needed.^5^ In 2017, the World Health Assembly accepted the global cancer challenge resolution, which stated the importance of including children and adolescents in the development of cancer control programmes.^8^ The World Health Assembly noted in particular that these populations often experience delays and difficulties in accessing care. Unfortunately, the resolution did not address the unique needs of young adults separately, thus reinforcing a gap in current global cancer control paradigms. There is an opportunity for advocates to directly address this gap, petitioning member states and developing an amendment specific to adolescents and young adults by emphasising the barriers faced by these patients.

The psychosocial challenges adolescents and young adults face is an important issue since these challenges are truly unique across the age spectrum and require resources and skills that are often not available to cancer treatment teams.^25^ The age range of adolescents and young adults encompasses their formative years in life and spans the time from completing education, to possibly starting a career and raising children, and potentially contributing to society more broadly. A cancer diagnosis during these years can have a considerable impact on individuals' future life trajectory through major stressors, including feelings of isolation, anxiety and depression, concerns about infertility, discontinuing schooling or work, and financial hardship.^1^, ^12^, ^26^, ^27^, ^28^ Efforts to mitigate the issues distinct to this age group have resulted in the formation of organisations to help support adolescent and young adult patients with cancer. However, although these oncology advocacy efforts focused on adolescents and young adults have been successful in creating awareness campaigns and implementing adolescent and young adult programmes at cancer centres, these efforts have largely been limited to high-income countries.^29^ These initiatives need to be expanded globally, particularly in low SDI settings—which carry a disproportionate burden of adolescent and young adult cancer DALYs—with appropriate local knowledge and champions.

The array of cancer types is also unique in adolescents and young adults compared to children and adults. Even what seems to be the same cancer is often biologically different in adolescents and young adults than in patients of other age groups and thereby might benefit from a different approach to therapy.^6^ For these and other reasons, survival improvements in adolescent and young adult patients with cancer have lagged behind those of children and adults for several cancer types.^9^ Delivery of cancer care to adolescents and young adults should be prioritised and optimised, especially in non-high SDI settings, where the majority of DALYs are reported. At present, adolescent and young adult patients often do not have an obvious health-care home and are frequently grouped into adult oncology service programmes because of age restrictions in paediatric wards or facilities.^12^, ^16^, ^30^ Where a patient receives care has important clinical and policy ramifications, as there is evidence of improvement in survival outcomes for some cancer types (eg, acute lymphoblastic leukaemia) when adolescents and young adults are treated according to paediatric protocols, which are often complex and might be unavailable in adult cancer centres.^31^, ^32^ Furthermore, treatment by specialised adolescent and young adult oncology teams has been associated with improved survival of adolescents and young adults with cancer in some high-income countries, possibly as a result of access to cancer expertise, clinical trials, and multidisciplinary care.^33^ Although access to these centres and programmes is not currently possible in many settings, most adolescent and young adult patients with cancer might benefit from a multidisciplinary treatment approach involving close collaboration between paediatric and medical oncologists.

To improve outcomes in this unique population, a new approach to global cancer control in adolescents and young adults is required. Faced with similar challenges for children and adolescents, the recently launched WHO Global Initiative for Childhood Cancer provides one implementation framework for addressing gaps in access and care. This initiative includes adolescents up to 19 years of age, bridging the lowest ages included in adolescent and young adult oncology, and at least one cancer that predominantly occurs in adolescents and young adults—Hodgkin lymphoma—is an index cancer in this initiative. Although this is excellent news for the younger bounds of the adolescent and young adult spectrum, the Global Initiative for Childhood Cancer initiative covers only 8·6% (95% UI 8·2–9·1) of all adolescent and young adult cancer cases, and the unique needs of and potential synergies with adolescent and young adult cancer care are not specifically addressed. A dedicated initiative similar to the Global Initiative for Childhood Cancer is unlikely in the near future. Therefore, integration of adolescent and young adult cancer policies within WHO cancer initiatives such as the Global Initiative for Childhood Cancer and the WHO Cervical Cancer Elimination Initiative, a cancer that comprises approximately 10·0% (8·5–10·9) of adolescent and young adult cancer cases globally, could be prioritised in the short term. A strategy to integrate specific objectives of relevance to the adolescent and young adult population in these initiatives would immediately cover almost one-fifth of adolescent and young adult cancer cases and provide a template for future global cancer initiatives. Potential areas for collaboration could include integration of human papillomavirus (HPV) vaccination efforts into the Global Initiative for Childhood Cancer, an as-yet untapped opportunity, and inclusion of policies specific to adolescent and young adult patients in the WHO technical packages, such as provisions for referrals and access to expert adolescent and young adult cancer care and appropriate treatment regimens, psychosocial support, and universal health coverage to reduce financial hardship. Intentional collaboration with other WHO cancer initiatives could facilitate progress in both areas and highlight other potential areas of synergy for improving cancer outcomes in adolescents and young adults.

The adolescent and young adult cancer burden estimates presented in this study also underscore the limitations of GBD and possible opportunities to improve future assessments of the global adolescent and young adult cancer burden.^34^ The classification of adolescent and young adult cancers in this study is based on the GBD cancer cause list, which has historically focused on cancers occurring in adulthood. As such, GBD 2019 did not differentiate some of the most common adolescent and young adult cancer types, such as soft tissue sarcomas and bone tumours. These cancers contribute to the substantial proportion of “other malignant neoplasms” in this age range, cancers that do not have their own individual GBD cancer causes. Many of the rarer cancers that fall into this “other malignant neoplasms” category rely on complex multidisciplinary therapy (eg, provided by medical, radiation, and surgical oncologists), and resource allocation could be improved if their global burden was accurately known.^5^ Future studies should use the recently updated recommendations for classification of adolescent and young adult cancers to better characterise the cancer burden in this age group and minimise the number of cancer types falling into the “other malignant neoplasms” category.^34^ Additionally, the quality of the data obtained, especially from low-resource settings, might cause challenges due to underestimates or miscategorisation of less common cancer types.^10^ For instance, there was an observed decrease in the proportion of adolescent and young adult cancer DALYs due to brain and CNS cancers across the SDI spectrum, with the lowest proportion in low SDI settings. As many lower SDI countries do not have population-based cancer registries or robust referral mechanisms, the data upon which these estimates are drawn might be subject to underdiagnosis, misdiagnosis, or under-reporting. Therefore, results in lower SDI settings should be interpreted with caution. However, these modelled results provide a useful contribution towards determining the global burden of adolescent and young adult cancer, especially in regions where such data do not exist or are scarce. An additional limitation of the present analysis is that SDI was applied at the national level, but within-country socio-economic status can vary greatly. Improving global adolescent and young adult cancer burden estimates must be rooted in capacity-building efforts that consider the local context, to ensure identification of incident cancer cases and deaths in the adolescent and young adult population, as well as expansion of and support for population-based cancer registration systems. Another potential limitation of the present analysis is the current approach to YLD estimation, which accounts for only 10 years after cancer diagnosis. Previous studies have shown that late effects, such as cardiomyopathy, can affect the adolescent and young adult population beyond the 10-year cutoff point.^25^, ^35^ This limits the ability to determine the long-term chronic disease burden and competing risks for survivors in this population, which have the potential to be substantial. Additionally, the experience of disability for survivors of childhood cancer might be different to that of the general population. Thus, GBD 2019 might be underestimating the YLDs and DALYs associated with cancer in adolescents and young adults, and future efforts might be needed to identify ways to account for this limitation. Finally, this study focused on estimates from 2019, and thus did not incorporate the direct and indirect effects of the COVID-19 pandemic on the global adolescent and young adult cancer burden. This will be an important area of consideration in future studies as the data become available.

This report of the adolescent and young adult cancer burden from GBD 2019 identified a considerable burden of DALYs due to cancer in the global adolescent and young adult population. The absolute mortality burden in adolescents and young adults is highest in non-high SDI settings, underscoring the need for a global effort to improve outcomes in this population, with collaboration at the regional and country levels, as well as between governments, institutions, academic societies, and patient advocacy and non-profit organisations. Efforts to comprehensively estimate the global burden of cancer in adolescents and young adults are a crucial first step.^10^, ^21^ Adolescent and young adult oncology has historically been less prioritised than cancer disciplines in younger and older patients. Increased awareness of the burden of cancer in this population could lead to targeted interventions for improved outcomes.

## Data sharing

To download the data used in these analyses, please visit the Global Health Data Exchange GBD 2019 website at http://ghdx.healthdata.org/gbd-2019.

## Declaration of interests

R Ancuceanu reports consulting fees from Abbvie; and payment or honoraria for lectures, presentations, speakers bureaus, manuscript writing or educational events from Abbvie, Sandoz and B. Braun, all outside the submitted work. H Ariffin reports payment or honoraria for lectures, presentations, speakers bureaus, manuscript writing or educational events from Amgen, all outside the submitted work. M Atashzar reports support for the present manuscript from medical writing and analysis of data. M Atashzar reports paid consulting fees; and receipt of equipment, materials, drugs, medical writing, gifts or other services from medical writing, all outside the submitted work. P Atorkey reports support for the present manuscript from the School of Medicine and Public Health, University of Newcastle, Australia, Hunter Medical Research Institute, University of Newcastle, Australia, and Hunter New England, Population Health. A Aujayeb reports grants or contracts from Rocket Medical Plc; payment or honoraria for lectures, presentations, speakers bureaus, manuscript writing or educational events from Rocket Medical Plc for talks given on pneumothorax and work done on digital suction device; and leadership or fiduciary role in board, society, committee or advocacy group, paid or unpaid with Mesothelioma UK as Trustee, all outside the submitted work. M Ausloos reports grants or contracts from the Romanian National Authority for Scientific Research and Innovation, CNDS-UEFISCDI, project number PN-III-P4-ID-PCCF-2016-0084 “Understanding and modelling time-space patterns of psychology-related inequalities and polarization”; all outside the submitted work. T Bärnighausen reports grants or contracts from the European Union (Horizon 2020 and EIT Health), German Research Foundation (DFG), US National Institutes of Health, German Ministry of Education and Research, Alexander von Humboldt Foundation, Else-Kräner-Fresenius-Foundation, Wellcome Trust, Bill & Melinda Gates Foundation, KfW, UNAIDS, and WHO; consulting fees from KfW on the OSCAR initiative in Vietnam; participation on a Data Safety Monitoring Board or Advisory Board with NIH-funded study “Healthy Options” as Chair of the Data Safety and Monitoring Board (DSMB), German National Committee on the “Future of Public Health Research and Education”; Chair of the scientific advisory board to the EDCTP Evaluation; Member of the UNAIDS Evaluation Expert Advisory Committee; National Institutes of Health Study Section Member on Population and Public Health Approaches to HIV/AIDS (PPAH), US National Academies of Sciences, Engineering, and Medicine's Committee for the “Evaluation of Human Resources for Health in the Republic of Rwanda under the President's Emergency Plan for AIDS Relief (PEPFAR)”, University of Pennsylvania (UPenn) Population Aging Research Center (PARC) as an External Advisory Board Member; leadership or fiduciary role in board, society, committee or advocacy group, paid or unpaid as a Co-chair of the Global Health Hub Germany (which was initiated by the German Ministry of Health); all outside the submitted work. N Bekele reports participation on a Data Safety Monitoring Board or Advisory Board as Ethical review board member for two years; and leadership or fiduciary role in board, society, committee or advocacy group, paid or unpaid with Wollo University as an unpaid graduate program coordinator for three years, all outside the submitted work. S Bhaskar reports grants or contracts from NSW Ministry of Health, NSW Brain Clot Bank; and leadership or fiduciary role in board, society, committee or advocacy group, paid or unpaid with Rotary Club of Sydney, Australia as Board Director, International Rotary Fellowship of Rotarian Healthcare Professionals (IRFRHP), UK as Board Director, and BMC Neurology as Editorial Board Member, all outside the submitted work. J Conde reports grants or contracts from European Research Council Starting Grant, ERC-StG-2019-848325; patents planned, issued or pending, as Functionalized nanoparticles and compositions for cancer treatment and methods, U.S. Application No. 62/334538 and TRPV2 Antagonists, WO Application No. PCT/PT2018/050035; and support from TargTex S.A. as co-founder and shareholder, all outside the submitted work. X Dai reports support for the present manuscript from Bloomberg Philanthropies and the Bill and Melinda Gates Foundation through their employment at IHME. I Filip reports payment or honoraria for lectures, presentations, speakers bureaus, manuscript writing or educational events from Avicenna Medical and Clinical Research Institute, all outside the submitted work. L Force reports support for the present manuscript from the Bill and Melinda Gates Foundation and American Lebanese Syrian Associated Charities for providing funding, related to their employment at IHME. L. Force reports grants or contracts from St Baldrick's Foundation; leadership or fiduciary role in board, society, committee or advocacy group, unpaid with the Lancet Oncology International Advisory Board; and payments towards federal student loans from the NIH Loan Repayment Award, all outside the submitted work. F Ghassemi reports support for the present manuscript for medical writing and literature review. N Ghith reports grants or contracts from NovoNordisc Foundation through salary covered by grant NNF16OC0021856, all outside the submitted work. A Guha reports grants or contracts from American Heart Association-Strategically Focused Research Network Grant in Disparities in Cardio-Oncology (#847740,#863620), all outside the submitted work. V Gupta reports grants or contracts from National Health and Medical research Council (NHMRC) Australia, all outside the submitted work. J Haro reports grants or contracts from Eli Lilly and Co., all outside the submitted work. H Henrikson reports support for the present manuscript from the Bill and Melinda Gates Foundation, American Lebanese Syrian Associated Charities, and Saint Baldrick's Foundation, all for providing funding, related to their employment at IHME. C Herteliu reports grants or contracts from Romanian National Authority for Scientific Research and Innovation, CNDS-UEFISCDI, project number PN-III-P4-ID-PCCF-2016-0084, grant title “Understanding and modelling time-space patterns of psychology-related inequalities and polarization” as research team member, and from Romanian National Authority for Scientific Research and Innovation, CNDS-UEFISCDI, project number PN-III-P2-2.1-SOL-2020-2-0351, grant title “Approaches within public health management in the context of COVID-19 pandemic,” as project manager, all outside the submitted work. K Innos reports support for the present manuscript from Estonian Research Council, Grant No PRG722. S M S Islam reports grants or contracts from the NHMRC Emerging Leadership Fellowship and the National Heart Foundation of Australia Fellowship, all outside the submitted work. N E Ismail reports leadership or fiduciary role in board, society, committee or advocacy group, paid or unpaid with Malaysian Academy of Pharmacy as an unpaid Council Member, all outside the submitted work. I Karaye reports support for the present manuscript from the Bill and Melinda Gates Foundation, American Lebanese Syrian Associated Charities, and Saint Baldrick's Foundation, all for providing funding, related to their employment at IHME. J Kauppila reports grants or contracts from Sigrid Juselius Foundation, Finnish Cancer Foundation, and Päivikki and Sakari Sohlberg Foundation, all outside the submitted work. T Ketterl reports consulting fees from Fennec Pharmaceuticals, Inc for advisory services, all outside the submitted work. J Kocarnik reports support for the present manuscript from the Bill and Melinda Gates Foundation and American Lebanese Syrian Associated Charities for providing funding, related to their employment at IHME. M-C Li reports support for the present manuscript from Ministry of Science and Technology, Taiwan (MOST 110-2314-B-003-001). J A Loureiro reports support for the present manuscript from Scientific Employment Stimulus (FCT), CEECINST/00049/2018, for salary support and Base Funding, UIDB/00511/2020 of the LEPABE, funded by national funds through the FCT/MCTES (PIDDAC) for research support. M Mahmoudi reports support from the Academic Parity Movement, as co-founder and director; support from Partners in Global Wound Care (PGWC) as Founding Partner; and reports royalties/honoraria for published books, plenary lectures, and licensed patent, all outside the submitted work. O Odukoya reports support for the present manuscript from the Fogarty International Center of the National Institutes of Health under the Award Number K43TW010704. A Pana reports grants or contracts from ARPIM, Amgen, Janssen, Astra Zeneca, Novartis Oncology, BMS, Angelini, and Servier; and participation on a Data Safety Monitoring Board or Advisory Board with Novartis Oncology and Pfizer, all outside the submitted work. M Postma reports stock or stock options in Pharmacoeconomics Advice Groningen and Health-Ecore, all outside the submitted work. A Radfar reports payment or honoraria for lectures, presentations, speakers bureaus, manuscript writing or educational events from Avicenna Medical and Clinical Research Institute, all outside the submitted work. A Riad reports grants or contracts from Masaryk University; and support from Cochrane Collaboration as deputy director of Cochrane Czech Republic center, all outside the submitted work. M Saylan reports support from their employer Bayer, all outside the submitted work. M Šekerija reports payment or honoraria for lectures, presentations, speakers bureaus, manuscript writing or educational events from Roche and Johnson & Johnson, all outside the submitted work. D A Santos Silva reports support for the present manuscript from Coordenação de Aperfeiçoamento de Pessoal de Nível Superior—Brazil (CAPES) and National Council for Scientific and Technological Development (CNPq), Brazil. D A Santos Silva reports grants or contracts from the Coordenação de Aperfeiçoamento de Pessoal de Nível Superior—Brazil (CAPES)—Finance Code 001 and is supported in part by National Council for Scientific and Technological Development (CNPq), Brazil (302028/2018-8), all outside the submitted work. J A Singh reports consulting fees from Crealta/Horizon, Medisys, Fidia, PK Med, Two labs Inc, Adept Field Solutions, Clinical Care options, Clearview healthcare partners, Putnam associates, Focus forward, Navigant consulting, Spherix, MedIQ, Jupiter Life Science, UBM LLC, Trio Health, Medscape, WebMD, and Practice Point communications, and the National Institutes of Health and the American College of Rheumatology; payment or honoraria for lectures, presentations, speakers bureaus, manuscript writing or educational events from Simply Speaking; support for attending meetings and/or travel from OMERACT, an international organization that develops measures for clinical trials and receives arm's length funding from 12 pharmaceutical companies, when traveling to OMERACT meetings; participation on a Data Safety Monitoring Board or Advisory Board as a member of the FDA Arthritis Advisory Committee; leadership or fiduciary role in other board, society, committee or advocacy group, paid or unpaid, with OMERACT as a member of the steering committee, with the Veterans Affairs Rheumatology Field Advisory Committee as a chair member, and with the UAB Cochrane Musculoskeletal Group Satellite Center on Network Meta-analysis as Director and editor; stock or stock options in TPT Global Tech, Vaxart pharmaceuticals, Atyu biopharma, Charlotte's Web Holdings Inc. and previously owned stock options in Amarin, Viking, and Moderna pharmaceuticals; all outside the submitted work.
