# Supplementary material for: The global burden of adolescent and young adult cancer in 2019: a systematic analysis for the Global Burden of Disease Study 2019
Source: Lancet Oncol. 2022 Jan;23(1):27–52. doi: 10.1016/S1470-2045(21)00581-7 (PMC8716339; doi:10.1016/S1470-2045(21)00581-7)
Supplement: Supplementary appendix [file mmc1.pdf]

THE LANCET
Oncology

Supplementary appendix

This appendix formed part of the original submission and has been peer reviewed. We post it as supplied by the authors.

Supplement to: GBD 2019 Adolescent and Young Adult Cancer Collaborators. The global burden of adolescent and young adult cancer in 2019: a systematic analysis for the Global Burden of Disease Study 2019. *Lancet Oncol* 2021; published online Dec 3. https://doi.org/10.1016/S1470-2045(21)00581-7.

<u>Supplementary Content</u>

The global burden of adolescent and young adult cancer in 2019: a systematic analysis for the Global Burden of Disease Study 2019

Please note that portions of this supplement were copied from the supplementary content to the recent GBD publications:

GBD 2019 Cancer Collaborators. Morbidity and mortality for 29 cancer groups by country and territory and Socio-demographic Index, 1990-2019: a systematic analysis for the Global Burden of Disease Study 2019. *JAMA Oncology*. **In Press**.[1];

Force LM, Abdollahpour I, Advani SM, et al. The global burden of childhood and adolescent cancer in 2017: an analysis of the Global Burden of Disease Study 2017. *The Lancet Oncology* 2019; **20:** 1211–25.[2];

and

Vos T, Lim SS, Abbafati C. Global burden of 369 diseases and injuries in 204 countries and territories, 1990–2019: a systematic analysis for the Global Burden of Disease Study 2019. *The Lancet* 2020; **396**: 1204–22.[3]

Table of Contents 2

The Global Burden of Disease (GBD) Study **4**
GATHER Guidelines Checklist **5**
Definition of Indicator **7**
GBD Cancer Estimation Process **8**
Appendix Figure 1: Flowchart of GBD cancer mortality and Years of Life Lost (YLLs) estimation **8**
Appendix Figure 2: Flowchart of GBD cancer incidence and Years Lived with Disability (YLDs) estimation **9**

Data sources **10**
Cancer registry (CR) data sources **10**
Mortality-to-incidence (MIR) ratio data sources **10**
Cancer mortality data in the cause of death (CoD) database other than cancer registry data **10**
Bias of categories of input data **10**

Cancer types estimated in the GBD 2019 study **11**
ICD cancer codes mapped to GBD 2019 cancer causes **11**
Cancers in the GBD cause hierarchy **11**

Data analysis **11**
Cancer registry data processing **11**
Appendix Table 1: List of International Classification of Diseases (ICD) codes mapped to the Global Burden of Disease cause list for cancer incidence data **12**
Appendix Table 2: List of International Classification of Diseases (ICD) codes mapped to the Global Burden of Disease cause list for cancer mortality data **17**
Cause of death database formatting **26**
Appendix Table 3: Restrictions on age and sex by each cancer type in GBD 2019 **27**
CODEm models **28**
Appendix Table 4: GBD 2019 covariates and level of covariates used in cause of death modelling for cancer types estimated **28**
CoDCorrect **49**
Calculating YLLs **50**
Incidence estimation **50**
Prevalence estimation **50**
Appendix Table 5: Duration of four prevalence phases by cancer in GBD 2019 **51**
Appendix Table 6: Lay description of cancer states and corresponding disability weights in GBD 2019 **55**
Calculating Proportional Burden **56**
Reporting Standards **56**
Socio-demographic Index (SDI) Definition and Calculation **56**
Uncertainty Estimation **57**
Limitations **57**

Additional Methodology Tables and Figures **58**
Appendix Figure 3: Map of GBD world super-regions, 2019 **58**
Appendix Figure 4: Map of GBD world regions, 2019 **59**
Appendix Table 7: SDI quintiles for Countries Estimated in GBD 2019 **60**
Appendix Table 8: Population of 15-39 year-old individuals in the locations comprising each SDI Quintile in GBD 2019 **61**

Additional Results in Tables and Figures **62**
Appendix Table 9: Adolescent and young adult cancer DALYs, incidence, and mortality burden globally and by SDI quintile in 2019, males **62**
Appendix Table 10: Adolescent and young adult cancer DALYs, incidence, and mortality burden globally and by SDI quintile in 2019, females **66**

Appendix Table 11: Adolescent and young adult cancer YLD and YLL burden globally and by SDI quintile in 2019, males **70**
Appendix Table 12: Adolescent and young adult cancer YLD and YLL burden globally and by SDI quintile in 2019, females **73**
Appendix Table 13: Adolescent and young adult cancer YLD and YLL burden globally and by SDI quintile in 2019, both sexes combined **76**
Appendix Table 14: Percentage of adolescent and young adult cancer DALY burden made up by YLDs and YLLs globally and by SDI quintile in 2019 **79**
Appendix Figure 4: Percentage of adolescent and young adult (15-39 years) total DALY cancer burden attributable to cancer types, global, 2019, in (A) males, (B) females, and (C) both sexes **81**
Appendix Figure 5: (A) Absolute and (B) proportional adolescent and young adult (15-39) cancer mortality burden, globally, by sex in 2019 **82**
Appendix Figure 6: (A) Absolute and (B) proportional adolescent and young adult (15-39) cancer incidence burden, globally, by sex in 2019 **83**
Appendix Figure 7: Adolescent and young adult (15-39 years) cancer, both sexes combined, 2019 by GBD regions in (A) age-standardised DALY rates, and (B) proportional DALY burden **84**
Appendix Figure 8: Cancer ranking for adolescent and young adult cancer (15-39 years) by total DALYs within global setting, Socio-demographic Index (SDI) quintile, super region, region, and country, both sexes combined, 2019 **85**
Appendix Figure 9: Map of highest DALYs by cancer in adolescent and young adult (15-39 years) males by country, 2019 **99**
Appendix Figure 10: Map of highest DALYs by cancer in adolescent and young adult (15-39 years) females by country, 2019 **100**
Appendix Figure 11: Age-standardised incidence and mortality rates by Socio-demographic Index (SDI) quintiles for adolescent and young adult (15-39 years) cancers, male, 2019 **101**
Appendix Figure 12: Age-standardised incidence and mortality rates by Socio-demographic Index (SDI) quintiles for adolescent and young adult (15-39 years) cancers, female, 2019 **102**
Appendix Figure 13: Age-standardised incidence and mortality rates by Socio-demographic Index (SDI) quintiles for adolescent and young adult (15-39 years) cancers, both sexes combined, 2019 **103**
Appendix Figure 14: Age-standardised DALY rates by Socio-demographic Index (SDI) quintiles for adolescent and young adult (15-39 years) cancers, male and female, 2019 **104**
Appendix Figure 15: Ranking of absolute deaths due to cancer in (A) children (0-14 years) and (B) adults (40+ years), for both sexes combined, globally, and by SDI **105**
Appendix Table 15: Global absolute DALYs for each adolescent and young adult cancer type by age group, in 2019, for both sexes combined **107**

Additional Results in Tables and Figures: Adolescent and young adult cancers analysis reassessed using the age range 15 to 29 years 115
Appendix Table 16: Adolescent and young adult cancers burden globally, by SDI quintile, and by cancer in 2019, ages 15 to 29, both sexes combined **115**
Appendix Figure 16: Global map of age-standardised DALY rates for adolescent and young adult cancers ages 15-29 years for both sexes combined in 2019, categorised by quintile, excluding non-melanoma skin cancers **119**
Appendix Figure 17: Adolescent and young adult (15-29 years) cancer burden by age group for both sexes, 2019, in (A) age-specific DALY rates and (B) proportional DALY burden **120**
Appendix Figure 18: Adolescent and young adult (15-29 years) cancer, both sexes combined, 2019 by Socio-demographic Index (SDI) quintiles in (A) age-standardised DALY rates and (B) proportional DALY burden **121**
Appendix Figure 19: Adolescent and young adult cancer burden by 15-39, 15-29, and 30-39 year age groups for both sexes, 2019, in (A) age-specific DALY rates and (B) proportional DALY burden **122**

References 123

Author Contributions 127

The Global Burden of Disease (GBD) study

The Global Burden of Disease (GBD) study was created in an effort to establish comprehensive and comparable health metrics. A key principle in the GBD approach to estimation of disease burden is that an individual can have only one cause of death, while recognising that this may underestimate disease burden due to intermediate causes of death. In addition to reporting estimates of mortality and years of life lost (YLLs) for over 300 diseases and injuries, the GBD study also quantifies non-fatal components of disease including years lived with disability (YLDs) and disability-adjusted life-years (DALYs), a metric that represents a combination of both the fatal and non-fatal components of disease. The GBD approach uses all relevant data sources, rather than a single type of data. Finally, as there is continual methodological refinement with each GBD iteration, the results in each successive iteration supersede the results of prior GBD studies for the entire newly estimated time series. A protocol for the GBD study can be found online at http://www.healthdata.org/sites/default/files/files/Projects/GBD/GBD_Protocol.pdf.

GATHER[4] Guidelines Checklist

Item #	Checklist item	Reported on page #
**Objectives and funding**		
**1**	Define the indicator(s), populations (including age, sex, and geographic entities), and time period(s) for which estimates were made.	Appendix pg. 7
**2**	List the funding sources for the work.	See main manuscript
**Data Inputs**		
For all data inputs from multiple sources that are synthesised as part of the study:		
**3**	Describe how the data were identified and how the data were accessed.	Appendix pg. 10
**4**	Specify the inclusion and exclusion criteria. Identify all ad-hoc exclusions.	Appendix pg. 10
**5**	Provide information on all included data sources and their main characteristics. For each data source used, report reference information or contact name/institution, population represented, data collection method, year(s) of data collection, sex and age range, diagnostic criteria or measurement method, and sample size, as relevant.	Appendix pg. 10 and http://ghdx.healthdata.org/gbd-2019
**6**	Identify and describe any categories of input data that have potentially important biases (eg, based on characteristics listed in item 5).	Appendix pg. 10
For data inputs that contribute to the analysis but were not synthesised as part of the study:		
**7**	Describe and give sources for any other data inputs.	Appendix pg. 10 and http://ghdx.healthdata.org/gbd-2019
For all data inputs:		
**8**	Provide all data inputs in a file format from which data can be efficiently extracted (eg, a spreadsheet rather than a PDF), including all relevant meta-data listed in item 5. For any data inputs that cannot be shared because of ethical or legal reasons, such as third-party ownership, provide a contact name or the name of the institution that retains the right to the data.	http://ghdx.healthdata.org/gbd-2019
**Data analysis**		
**9**	Provide a conceptual overview of the data analysis method. A diagram may be helpful.	Appendix pg. 8 & 9 (Appendix figures 1, 2)
**10**	Provide a detailed description of all steps of the analysis, including mathematical formulae. This description should cover, as relevant, data cleaning, data pre-processing, data adjustments and weighting of data sources, and mathematical or statistical model(s).	Appendix pg. 11-55
**11**	Describe how candidate models were evaluated and how the final model(s) were selected.	Found in *Section 3: Causes of death modelling methods* of the Supplementary appendix 1 to "GBD 2019 Diseases and Injuries Collaborators. Global burden of 369 diseases and injuries in 204 countries and territories, 1990–2019: a systematic

		analysis for the Global Burden of Disease Study 2019".[3] Details of covariate selection for cancer models can be found in: Appendix pg. 28-49 (Appendix table 4)
**12**	Provide the results of an evaluation of model performance, if done, as well as the results of any relevant sensitivity analysis.	Found in eTable 9 of the Supplementary Appendix to "Morbidity and mortality for 29 cancer groups by country and territory and Socio-demographic Index, 1990-2019: a systematic analysis for the Global Burden of Disease Study 2019".
**13**	Describe methods for calculating uncertainty of the estimates. State which sources of uncertainty were, and were not, accounted for in the uncertainty analysis.	Appendix pg. 57
**14**	State how analytic or statistical source code used to generate estimates can be accessed.	http://ghdx.healthdata.org/gbd-2019/code
**Results and Discussion**		
**15**	Provide published estimates in a file format from which data can be efficiently extracted.	GBD 2019 estimates are available online (https://vizhub.healthdata.org/gbd-compare/ and http://ghdx.healthdata.org/gbd-results-tool)
**16**	Report a quantitative measure of the uncertainty of the estimates (eg, uncertainty intervals).	See main manuscript, "Results"
**17**	Interpret results in light of existing evidence. If updating a previous set of estimates, describe the reasons for changes in estimates.	See main manuscript, "Discussion"
**18**	Discuss limitations of the estimates. Include a discussion of any modelling assumptions or data limitations that affect interpretation of the estimates.	Appendix pg. 57

Definition of Indicator
In this publication, estimates for 32 cancer groups which predominantly represent malignant neoplasms, for both sexes, for the year 2019 and for the five-year GBD age groups (15-19, 20-24, 25-29, 30-34, and 35-39*) included within "adolescent and young adult cancers" are presented globally and for regions which include 204 countries or territories. All ICD-9 codes pertaining to cancer (140-209) and ICD-10 codes (C00-C96) except for non-melanoma skin cancer (ICD-10: C44) and the majority of Kaposi sarcoma (ICD-10: C46) are included in these estimates (see section "5. Cause disaggregation" on pg. 24 of this appendix for more information on Kaposi sarcoma code handling in GBD). For a complete list of ICD codes and their respective GBD causes, refer to Appendix tables 1, 2. Where this analysis specifies "carcinomas", the following cancers are included in this grouping: Bladder cancer; Breast cancer; Cervical cancer; Colon and rectum cancer; Oesophageal cancer; Gallbladder and biliary tract cancer; Kidney cancer; Larynx cancer; Lip and oral cavity cancer; Liver cancer; Nasopharynx cancer; Other pharynx cancer; Ovarian cancer; Pancreatic cancer; Prostate cancer; Stomach cancer; Thyroid cancer; Tracheal, bronchus, and lung cancer; and Uterine cancer.

A complete list of countries and territories estimated in GBD 2019 can be found in "Table S3: GBD location hierarchy with levels" on page 1459 in Supplementary Appendix 1 to "GBD 2019 Diseases and Injuries Collaborators. Global burden of 369 diseases and injuries in 204 countries and territories, 1990–2019: a systematic analysis for the global burden of disease study 2019". We recognise that location-specific results are helpful; although limited country-specific results are reported in this analysis, more detailed estimates can be found in the GBD Results tool, http://ghdx.healthdata.org/gbd-results-tool, and the GBD Compare tool, https://vizhub.healthdata.org/gbd-compare/.

**In order to align with different definitions for the upper age range of "adolescent and young adult" cancer, we have also provided some supplementary GBD 2019 AYA Cancer results focusing on the narrower age range of 15-29 years, as this is the standard age range considered to comprise AYA cancer in some countries.[5–7] These results can be found starting on pg. 115 in this appendix in Appendix table 16 and Appendix figures 16–19.*

GBD Cancer Estimation Process

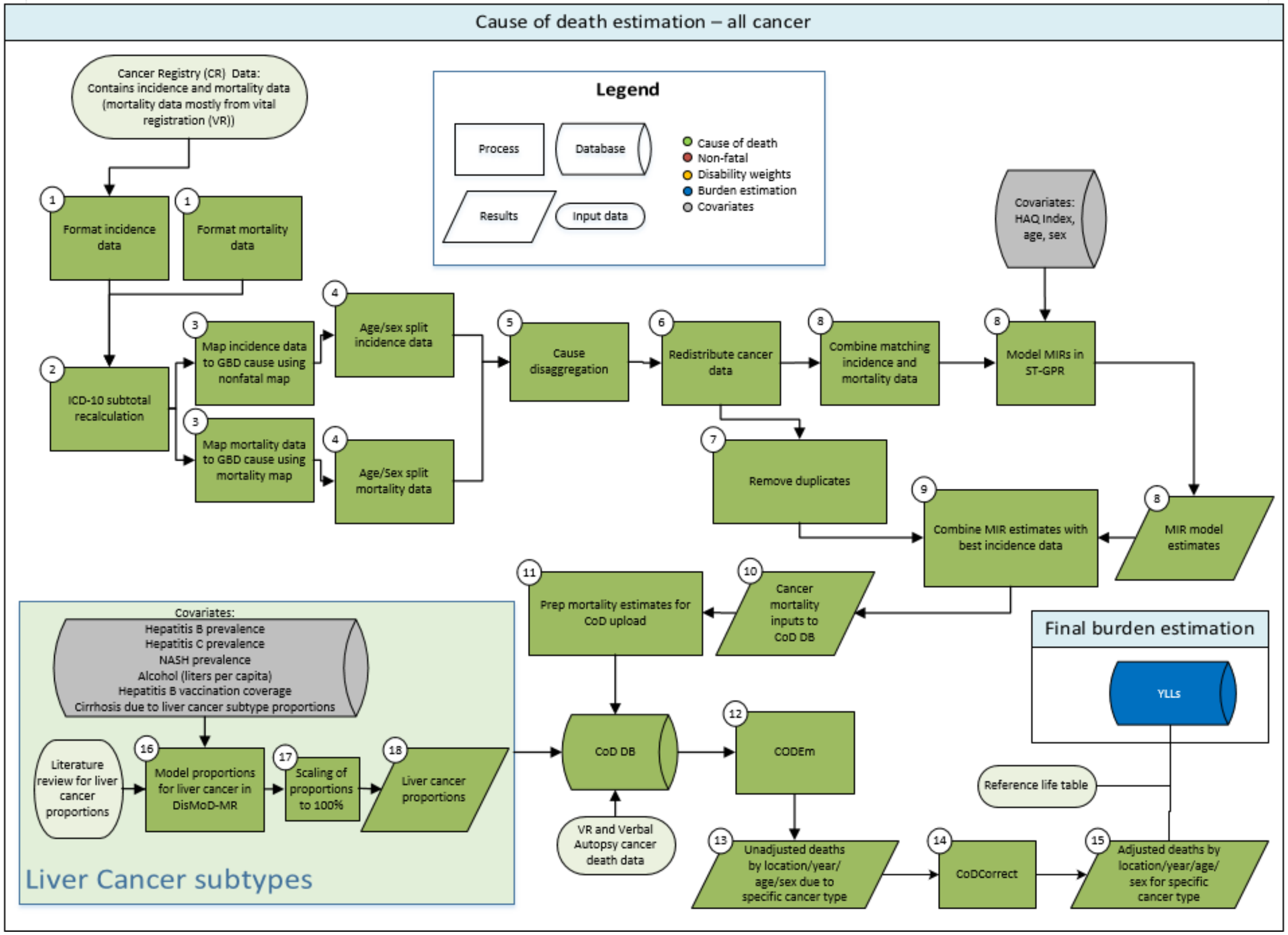

Appendix Figure 1: Flowchart of GBD cancer mortality and Years of Life Lost (YLLs) estimation. Abbreviations: CoD, causes of death; CODEm, cause of death ensemble model; DB, database; DisMod-MR, disease model - Bayesian meta-regression; HAQ Index, Healthcare Access and Quality Index; ICD, International Classification of Diseases; ST-GPR, spaciotemporal Gaussian process regression; MIR, mortality-to-incidence ratio; NASH, nonalcoholic steatohepatitis; VR, vital registration; YLL, years of life lost.

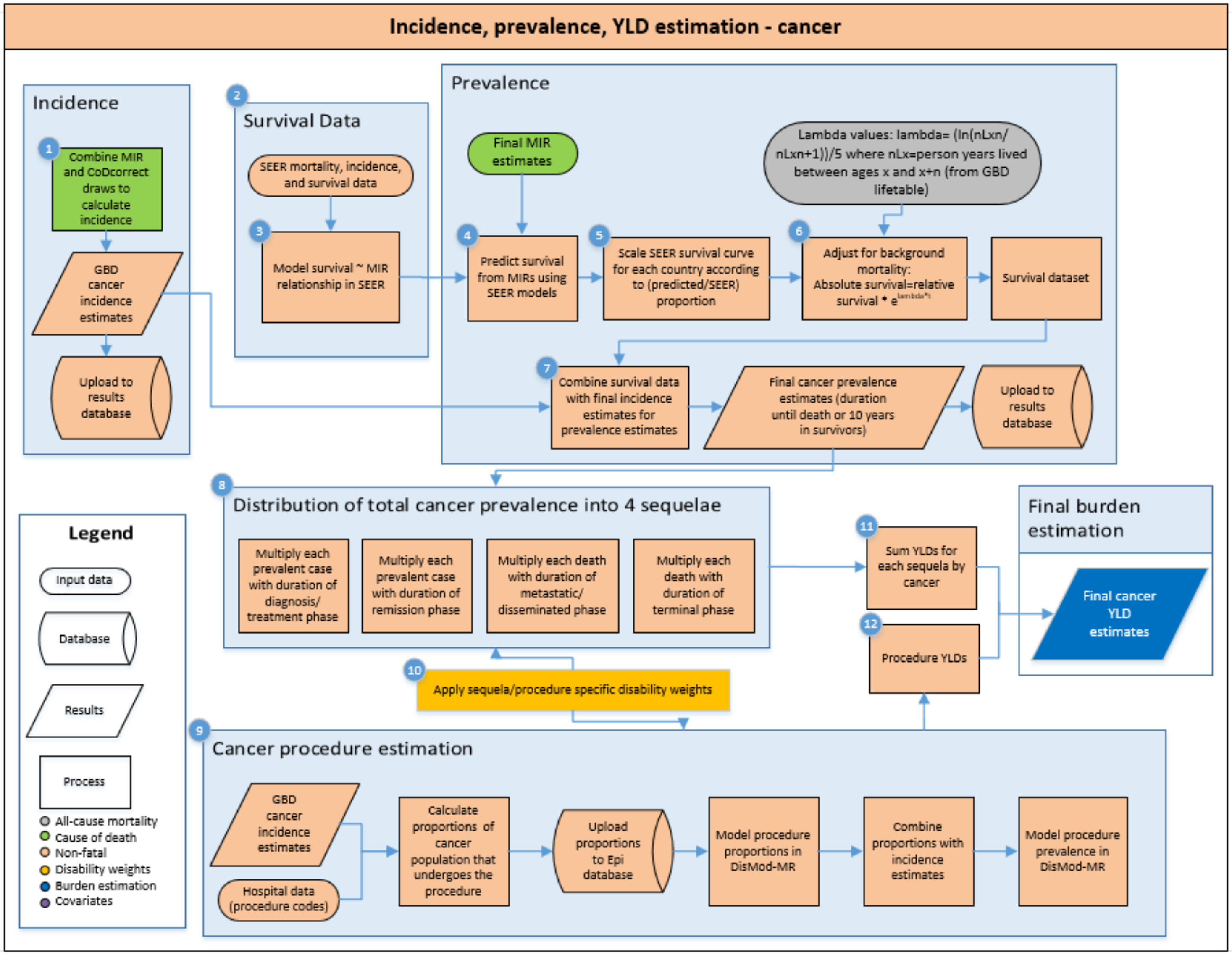

Appendix Figure 2: Flowchart of GBD cancer incidence and Years Lived with Disability (YLDs) estimation. Abbreviations: GBD, Global Burden of Disease Study; MIR, mortality-to-incidence ratio; SEER, Surveillance, Epidemiology and End Results Program; YLD, years lived with disability.

Data sources

Cancer registry (CR) data sources

Cancer incidence and mortality data were sought from individual cancer registries, such as the Surveillance, Epidemiology, and End Results (SEER) Program[8]; provided by collaborators; or downloaded from aggregated databases of cancer registry data such as "Cancer Incidence In Five Continents" (CI5),[9–19] EUREG,[20] or NORDCAN.[21] Only population-based cancer registries were included, with inclusion criteria that they included all cancers (ie, were not specialty registries), reported data for all age groups (except for pediatric cancer registries), and reported data for both sexes. Pathology-based cancer registries were included if they had a defined population. Hospital-based cancer registries were excluded. Redundant cancer registry data were excluded from either the final incidence data input or the MIR model input if a more detailed source (eg, providing more detailed age or diagnostic groups) was available for the same population. Preference was given to registries with national coverage over those with only local coverage, except those from countries where the GBD study provides subnational estimates. Data were excluded if the coverage population was unknown, except for in high SDI quintile locations with full geographic coverage where the GBD estimated population could be substituted. A list of the cancer registries included in our analysis and the years covered can be found in the online GBD citation tool http://ghdx.healthdata.org/gbd-2019. Additionally, CR data sources can be found in eTable 6 of the Supplementary appendix to "Cancer Incidence, Mortality, Years of Life Lost, Years Lived with Disability, and Disability-Adjusted Life Years for 29 Cancer Groups from 2010 to 2019: A Systematic Analysis of Cancer Burden Globally, Nationally, and by Socio-demographic Index for the Global Burden of Disease Study 2019".[1]

Mortality-to-incidence ratio (MIR) data sources

Most cancer registries only report cancer incidence. However, if a cancer registry also reported cancer mortality, mortality data were also extracted. CR sources with matching incidence and mortality data were used in the mortality-to-incidence ratio estimation.[1]

Cancer mortality data in the cause of death (CoD) database other than cancer registry data

In addition to cancer registry data, the GBD cause of death (CoD) database also contains cancer mortality data originating from multiple sources, including vital registration (VR) and verbal autopsy (VA) data. In countries without VR systems, VA studies are a viable data source to inform CoD. VA data are obtained by trained interviewers who use a standardised questionnaire to ask relatives about the signs, symptoms, and demographic characteristics of recently deceased family members. CoD is assigned based on the answers to the questionnaires. A detailed description of the data sources and processing steps for the cause of death database can be found in Supplementary Appendix 1 to the GBD 2019 paper "Global burden of 369 diseases and injuries in 204 countries and territories, 1990–2019: a systematic analysis for the Global Burden of Disease Study 2019" as well as in the online GBD citation tool http://ghdx.healthdata.org/gbd-2019.[3]

Bias of categories of input data

Potential biases of the input data included for the CoD database can also be found in the Supplementary Appendix 1 to the GBD 2019 paper "Global burden of 369 diseases and injuries in 204 countries and territories, 1990–2019: a systematic analysis for the Global Burden of Disease Study 2019".[3] Cancer registry data can be biased in multiple ways. A high proportion of ill-defined cancer cases in the cancer registry data requires redistribution of these cases to other cancers, which introduces a potential for bias. Changes between coding systems can lead to artificial differences in disease estimates; however, we adjust for this bias by mapping the different coding systems to GBD cancer causes. Underreporting of cancers that require advanced diagnostic techniques (eg, leukaemia, brain, pancreatic, and liver cancer) can be an issue in cancer registries from low-income countries. On the other hand, misclassification of

metastatic sites as primary cancer can lead to overestimation of cancer sites that are common sites for metastases (eg, brain cancer). Since many cancer registries are located in urban areas, the representativeness of the registry for the general non-urban population can also be problematic. The accuracy of mortality data reported in cancer registries usually depends on the quality of the vital registration system. If the vital registration system is incomplete or of poor quality, the mortality-to-incidence ratio can be biased to lower ratios.

Cancer types estimated in the GBD 2019 study

ICD cancer codes mapped to GBD 2019 cancer causes

Please refer to Appendix tables 1, 2 in this appendix in section "3. Mapping data to GBD causes" for a list of International Classification of Diseases (ICD) codes mapped to the Global Burden of Disease cause list for causes of death.

Cancers in the GBD cause hierarchy

The Global Burden of Disease (GBD) cause list is organised in a hierarchy. Levels 1 and 2 represent general groupings of causes, while Levels 3 and 4 represent increasingly specific causes. The general Level 1 group "Non-communicable diseases" includes the broad Level 2 group "Total Cancers", which includes all malignant neoplasms. Level 3 represents specific site-based cancers, such as "Stomach cancer" and "Liver cancer", and some Level 3 cancers are further subdivided into Level 4 subtypes (ie, Level 3 "Leukaemia" is divided into Level 4 causes "Acute lymphoid leukaemia", "Chronic lymphoid leukaemia", "Acute myeloid leukaemia", "Chronic myeloid leukaemia", and "Other leukaemia"). This analysis focuses only on the subset of Levels 3 and 4 cancers that are most relevant to the adolescent and young adult (15-39) age group, however all additional cancer groups estimated in GBD 2019 are available in the GBD 2019 Results tool http://ghdx.healthdata.org/gbd-results-tool.

Data analysis

Cancer registry data processing

Cancer registry data goes through multiple processing steps before entering the CoD database.

1. Formatting incidence and mortality data. First, the original data are transformed into standardised files, which included standardisation of format, categorisation, and registry names (#1 in Appendix figure 1).

2. Subtotal recalculation. Some cancer registries report individual codes as well as aggregated totals. An example of this would be where the registry data reports C18, C19, and C20 individually, and also the aggregated group of C18–C20 (colon and rectum cancer). The data processing step, "subtotal recalculation" (#2 in Appendix figure 1), verifies these totals and subtracts the values of any individual codes from the aggregates.

3. Mapping data to GBD causes. In the third step (#3 in in Appendix figure 1), cancer registry incidence data and cancer registry mortality data are mapped to GBD causes. A different map is used for incidence and for mortality data because of the assumption that there are no deaths for certain cancers. One example is benign or in situ neoplasms. Because cancer registries do not collect non-malignant neoplasms in a standardised way, any benign or in situ neoplasms reported in a cancer registry incidence dataset are dropped from that dataset. The same neoplasms reported in a cancer registry mortality dataset are instead mapped to the respective invasive cancer. For example, cases of "ductal carcinoma in situ" in a cancer registry incidence dataset are dropped from the dataset, while deaths from "ductal carcinoma in situ" in a cancer registry mortality dataset are mapped to breast cancer. Maps of ICD-codes to GBD causes for incidence and mortality data can be found in Appendix tables 1, 2.

Appendix Table 1: List of International Classification of Diseases (ICD) codes mapped to the Global Burden of Disease cause list for cancer incidence data

Cause	ICCC3	ICD-10	ICD-9
Lip and oral cavity cancer	XIf1	C00, C00.0, C00.1, C00.2, C00.3, C00.4, C00.5, C00.6, C00.8, C00.9, C01, C01.9, C02, C02.0, C02.1, C02.2, C02.3, C02.4, C02.8, C02.9, C03, C03.0, C03.1, C03.9, C04, C04.0, C04.1, C04.8, C04.9, C05, C05.0, C05.1, C05.2, C05.8, C05.9, C06, C06.0, C06.1, C06.2, C06.8, C06.80, C06.89, C06.9, C07, C07.0, C07.9, C08, C08.0, C08.1, C08.8, C08.9	140, 140.0, 140.1, 140.2, 140.3, 140.4, 140.5, 140.6, 140.7, 140.8, 140.9, 141, 141.0, 141.1, 141.2, 141.3, 141.4, 141.5, 141.6, 141.8, 141.9, 142, 142.0, 142.1, 142.2, 142.3, 142.8, 142.9, 143, 143.0, 143.1, 143.8, 143.9, 144, 144.0, 144.1, 144.4, 144.8, 144.9, 145, 145.0, 145.1, 145.2, 145.3, 145.4, 145.5, 145.6, 145.8, 145.9
Nasopharynx cancer	XIc	C11, C11.0, C11.1, C11.2, C11.3, C11.8, C11.9	147, 147.0, 147.1, 147.2, 147.3, 147.8, 147.9
Other pharynx cancer	NA	C09, C09.0, C09.1, C09.8, C09.9, C1, C10, C10.0, C10.1, C10.2, C10.3, C10.4, C10.8, C10.9, C12, C12.0, C12.9, C13, C13.0, C13.1, C13.2, C13.8, C13.9	146, 146.0, 146.1, 146.2, 146.3, 146.4, 146.5, 146.6, 146.7, 146.8, 146.9, 148, 148.0, 148.1, 148.2, 148.3, 148.4, 148.5, 148.8, 148.9
Oesophageal cancer	NA	C15, C15.0, C15.1, C15.2, C15.3, C15.4, C15.5, C15.8, C15.9	150, 150.0, 150.1, 150.2, 150.3, 150.4, 150.5, 150.6, 150.7, 150.8, 150.9
Stomach cancer	NA	C16, C16.0, C16.1, C16.2, C16.3, C16.4, C16.5, C16.6, C16.7, C16.8, C16.9	151, 151.0, 151.1, 151.2, 151.3, 151.4, 151.5, 151.6, 151.8, 151.9, 209.23
Colon and rectum cancer	XIf2, XIf3	C18, C18.0, C18.1, C18.2, C18.3, C18.4, C18.5, C18.6, C18.7, C18.8, C18.9, C19, C19.0, C19.9, C2, C20, C20.0, C20.8, C20.9, C21, C21.0, C21.1, C21.2, C21.8, C21.9	153, 153.0, 153.1, 153.2, 153.3, 153.4, 153.5, 153.6, 153.7, 153.8, 153.9, 154, 154.0, 154.1, 154.2, 154.3, 154.4, 154.8, 154.9, 209.1, 209.10, 209.11, 209.12, 209.13, 209.14, 209.15, 209.16, 209.17, 569.0, 569.43, 569.44, 569.84, 569.85
Liver cancer	VIIb, VIIc	C22, C22.0, C22.1, C22.3, C22.4, C22.5, C22.7, C22.8	155, 155.0, 155.1, 155.3, 155.5, 155.9
Gallbladder and biliary tract cancer	NA	C23, C23.0, C23.9, C24, C24.0, C24.1, C24.4, C24.8, C24.9	156, 156.0, 156.1, 156.2, 156.3, 156.8, 156.9
Pancreatic cancer	XIIa2	C25, C25.0, C25.1, C25.2, C25.3, C25.4, C25.7, C25.8, C25.9	157, 157.0, 157.1, 157.2, 157.3, 157.4, 157.5, 157.7, 157.8, 157.9
Larynx cancer	NA	C32, C32.0, C32.1, C32.2, C32.3, C32.8, C32.9	161, 161.0, 161.1, 161.2, 161.3, 161.8, 161.9
Tracheal, bronchus, and lung cancer	XIIa3, XIf4	C33, C33.0, C33.2, C33.9, C34, C34.0, C34.00, C34.01, C34.02, C34.1, C34.10, C34.11, C34.12, C34.2, C34.3, C34.30, C34.31, C34.32, C34.4, C34.7, C34.8, C34.80, C34.81, C34.82, C34.9, C34.90, C34.91, C34.92	162, 162.0, 162.1, 162.2, 162.3, 162.4, 162.5, 162.8, 162.9, 209.21
Malignant skin melanoma	XId	C43, C43.0, C43.1, C43.10, C43.11, C43.12, C43.2, C43.20, C43.21, C43.22, C43.3, C43.30, C43.31, C43.39, C43.4, C43.5, C43.51, C43.52, C43.59, C43.6, C43.60, C43.61, C43.62, C43.7, C43.70, C43.71, C43.72, C43.8, C43.9	172, 172.0, 172.1, 172.2, 172.3, 172.4, 172.5, 172.6, 172.7, 172.8, 172.9

Cause	ICCC3	ICD-10	ICD-9
Breast cancer	XIf6	C50, C50.0, C50.01, C50.011, C50.012, C50.019, C50.02, C50.021, C50.022, C50.029, C50.1, C50.11, C50.111, C50.112, C50.119, C50.12, C50.121, C50.122, C50.129, C50.2, C50.21, C50.211, C50.212, C50.219, C50.22, C50.221, C50.222, C50.229, C50.3, C50.31, C50.311, C50.312, C50.319, C50.32, C50.321, C50.322, C50.329, C50.4, C50.41, C50.411, C50.412, C50.419, C50.42, C50.421, C50.422, C50.429, C50.5, C50.51, C50.511, C50.512, C50.519, C50.52, C50.521, C50.522, C50.529, C50.6, C50.61, C50.611, C50.612, C50.619, C50.62, C50.621, C50.622, C50.629, C50.7, C50.8, C50.81, C50.811, C50.812, C50.819, C50.82, C50.821, C50.822, C50.829, C50.9, C50.91, C50.911, C50.912, C50.919, C50.92, C50.921, C50.922, C50.929	174, 174.0, 174.1, 174.2, 174.3, 174.4, 174.5, 174.6, 174.8, 174.9, 175, 175.0, 175.3, 175.9, 610, 610.0, 610.1, 610.2, 610.3, 610.4, 610.8, 610.9
Cervical cancer	XIf7	C53, C53.0, C53.1, C53.3, C53.4, C53.8, C53.9	180, 180.0, 180.1, 180.2, 180.3, 180.4, 180.5, 180.6, 180.8, 180.9, 622.1, 622.10, 622.11, 622.12, 622.2, 622.7
Uterine cancer	NA	C54, C54.0, C54.1, C54.2, C54.3, C54.4, C54.8, C54.9	182, 182.0, 182.1, 182.8, 182.9
Ovarian cancer	NA	C56, C56.0, C56.1, C56.2, C56.4, C56.9	183, 183.0
Prostate cancer	NA	C61, C61.0, C61.9	185, 185.0, 185.9
Testicular cancer	NA	C62, C62.0, C62.00, C62.01, C62.02, C62.1, C62.10, C62.11, C62.12, C62.9, C62.90, C62.91, C62.92	186, 186.0, 186.9
Kidney cancer	VI, VIa, VIa1, VIa2, VIa3, VIa4, VIb, VIc	C64, C64.0, C64.1, C64.2, C64.4, C64.5, C64.6, C64.8, C64.9, C65, C65.0, C65.1, C65.2, C65.9	189.0, 189.1, 189.5, 189.6, 209.24
Bladder cancer	XIf8	C67, C67.0, C67.1, C67.2, C67.3, C67.4, C67.5, C67.6, C67.7, C67.8, C67.9	188, 188.0, 188.1, 188.2, 188.3, 188.4, 188.5, 188.6, 188.7, 188.8, 188.9
Brain and central nervous system cancer	III, IIIa, IIIa1, IIIa2, IIIb, IIIc, IIIc1, IIIc2, IIIc3, IIIc4, IIId, IIId1, IIId2, IIId3, IIIe, IIIe1, IIIe2, IIIe3, IIIe4, IIIe5, IIIf, Xa, Xa1, Xa2, Xa3, Xa4, Xa5, Xa6	C70, C70.0, C70.1, C70.5, C70.6, C70.9, C71, C71.0, C71.1, C71.2, C71.3, C71.4, C71.5, C71.6, C71.7, C71.8, C71.9, C72, C72.0, C72.1, C72.2, C72.20, C72.21, C72.22, C72.3, C72.30, C72.31, C72.32, C72.4, C72.40, C72.41, C72.42, C72.5, C72.50, C72.59, C72.8, C72.9	191, 191.0, 191.1, 191.2, 191.3, 191.4, 191.5, 191.6, 191.7, 191.8, 191.9, 192, 192.0, 192.1, 192.2, 192.3, 192.4, 192.8, 192.9
Thyroid cancer	XIb	C73, C73.0, C73.1, C73.2, C73.3, C73.4, C73.5, C73.8, C73.9	193, 193.0, 193.2, 193.9

Cause	ICCC3	ICD-10	ICD-9
Mesothelioma	XIIa5	C45, C45.0, C45.1, C45.2, C45.3, C45.4, C45.5, C45.6, C45.7, C45.8, C45.9	NA
Hodgkin lymphoma	IIa	C81, C81.0, C81.00, C81.01, C81.02, C81.03, C81.04, C81.05, C81.06, C81.07, C81.08, C81.09, C81.1, C81.10, C81.11, C81.12, C81.13, C81.14, C81.15, C81.16, C81.17, C81.18, C81.19, C81.2, C81.20, C81.21, C81.22, C81.23, C81.24, C81.25, C81.26, C81.27, C81.28, C81.29, C81.3, C81.30, C81.31, C81.32, C81.33, C81.34, C81.35, C81.36, C81.37, C81.38, C81.39, C81.4, C81.40, C81.41, C81.42, C81.43, C81.44, C81.45, C81.46, C81.47, C81.48, C81.49, C81.5, C81.6, C81.7, C81.70, C81.71, C81.72, C81.73, C81.74, C81.75, C81.76, C81.77, C81.78, C81.79, C81.8, C81.9, C81.90, C81.91, C81.92, C81.93, C81.94, C81.95, C81.96, C81.97, C81.98, C81.99	201, 201.0, 201.00, 201.01, 201.02, 201.03, 201.04, 201.05, 201.06, 201.07, 201.08, 201.1, 201.10, 201.11, 201.12, 201.13, 201.14, 201.15, 201.16, 201.17, 201.18, 201.2, 201.20, 201.21, 201.22, 201.23, 201.24, 201.25, 201.26, 201.27, 201.28, 201.4, 201.40, 201.41, 201.42, 201.43, 201.44, 201.45, 201.46, 201.47, 201.48, 201.5, 201.50, 201.51, 201.52, 201.53, 201.54, 201.55, 201.56, 201.57, 201.58, 201.6, 201.60, 201.61, 201.62, 201.63, 201.64, 201.65, 201.66, 201.67, 201.68, 201.7, 201.70, 201.71, 201.72, 201.73, 201.74, 201.75, 201.76, 201.77, 201.78, 201.9, 201.90, 201.91, 201.92, 201.93, 201.94, 201.95, 201.96, 201.97, 201.98
Non-Hodgkin lymphoma	IIb, IIb1, IIb2, IIb3, IIb4, IIc, IId, IIe	C83.7, C83.70, C83.71, C83.72, C83.73, C83.74, C83.75, C83.76, C83.77, C83.78, C83.79, C83.8, C82, C82.0, C82.00, C82.01, C82.02, C82.03, C82.04, C82.05, C82.06, C82.07, C82.08, C82.09, C82.1, C82.10, C82.11, C82.12, C82.13, C82.14, C82.15, C82.16, C82.17, C82.18, C82.19, C82.2, C82.20, C82.21, C82.22, C82.23, C82.24, C82.25, C82.26, C82.27, C82.28, C82.29, C82.3, C82.30, C82.31, C82.32, C82.33, C82.34, C82.35, C82.36, C82.37, C82.38, C82.39, C82.4, C82.40, C82.41, C82.42, C82.43, C82.44, C82.45, C82.46, C82.47, C82.48, C82.49, C82.5, C82.50, C82.51, C82.52, C82.53, C82.54, C82.55, C82.56, C82.57, C82.58, C82.59, C82.6, C82.60, C82.61, C82.62, C82.63, C82.64, C82.65, C82.66, C82.67, C82.68, C82.69, C82.7, C82.8, C82.80, C82.81, C82.82, C82.83, C82.84, C82.85, C82.86, C82.87, C82.88, C82.89, C82.9, C82.90, C82.91, C82.92, C82.93, C82.94, C82.95, C82.96, C82.97, C82.98, C82.99, C83, C83.0, C83.00, C83.01, C83.02, C83.03, C83.04, C83.05, C83.06, C83.07, C83.08, C83.09, C83.1, C83.10,	200.2, 200.20, 200.21, 200.22, 200.23, 200.24, 200.25, 200.26, 200.27, 200.28, 200, 200.0, 200.00, 200.01, 200.02, 200.03, 200.04, 200.05, 200.06, 200.07, 200.08, 200.1, 200.10, 200.11, 200.12, 200.13, 200.14, 200.15, 200.16, 200.17, 200.18, 200.3, 200.30, 200.31, 200.32, 200.33, 200.34, 200.35, 200.36, 200.37, 200.38, 200.4, 200.40, 200.41, 200.42, 200.43, 200.44, 200.45, 200.46, 200.47, 200.48, 200.5, 200.50, 200.51, 200.52, 200.53, 200.54, 200.55, 200.56, 200.57, 200.58, 200.6, 200.60, 200.61, 200.62, 200.63, 200.64, 200.65, 200.66, 200.67, 200.68, 200.7, 200.70, 200.71, 200.72, 200.73, 200.74, 200.75, 200.76, 200.77, 200.78, 200.8, 200.80, 200.81, 200.82, 200.83, 200.84, 200.85, 200.86, 200.87, 200.88, 200.9, 202, 202.0, 202.00, 202.01, 202.02, 202.03, 202.04, 202.05, 202.06, 202.07, 202.08, 202.1, 202.10, 202.11, 202.12, 202.13, 202.14, 202.15, 202.16, 202.17, 202.18, 202.2, 202.20, 202.21, 202.22, 202.23, 202.24, 202.25, 202.26, 202.27, 202.28, 202.3, 202.30, 202.31, 202.32, 202.33, 202.34, 202.35, 202.36, 202.37, 202.38, 202.4, 202.40, 202.41, 202.42, 202.43, 202.44, 202.45, 202.46, 202.47, 202.48, 202.5, 202.50, 202.51, 202.52, 202.53, 202.54, 202.55, 202.56, 202.57, 202.58, 202.6, 202.60,

Cause	ICCC3	ICD-10	ICD-9
		C83.11, C83.12, C83.13, C83.14, C83.15, C83.16, C83.17, C83.18, C83.19, C83.2, C83.3, C83.30, C83.31, C83.32, C83.33, C83.34, C83.35, C83.36, C83.37, C83.38, C83.39, C83.4, C83.5, C83.50, C83.51, C83.52, C83.53, C83.54, C83.55, C83.56, C83.57, C83.58, C83.59, C83.6, C83.80, C83.81, C83.82, C83.83, C83.84, C83.85, C83.86, C83.87, C83.88, C83.89, C83.9, C83.90, C83.91, C83.92, C83.93, C83.94, C83.95, C83.96, C83.97, C83.98, C83.99, C84, C84.0, C84.00, C84.01, C84.02, C84.03, C84.04, C84.05, C84.06, C84.07, C84.08, C84.09, C84.1, C84.10, C84.11, C84.12, C84.13, C84.14, C84.15, C84.16, C84.17, C84.18, C84.19, C84.2, C84.3, C84.4, C84.40, C84.41, C84.42, C84.43, C84.44, C84.45, C84.46, C84.47, C84.48, C84.49, C84.5, C84.6, C84.60, C84.61, C84.62, C84.63, C84.64, C84.65, C84.66, C84.67, C84.68, C84.69, C84.7, C84.70, C84.71, C84.72, C84.73, C84.74, C84.75, C84.76, C84.77, C84.78, C84.79, C84.8, C84.9, C84.90, C84.91, C84.92, C84.93, C84.94, C84.95, C84.96, C84.97, C84.98, C84.99, C85, C85.0, C85.1, C85.10, C85.11, C85.12, C85.13, C85.14, C85.15, C85.16, C85.17, C85.18, C85.19, C85.2, C85.20, C85.21, C85.22, C85.23, C85.24, C85.25, C85.26, C85.27, C85.28, C85.29, C85.3, C85.4, C85.5, C85.6, C85.7, C85.8, C85.80, C85.81, C85.82, C85.83, C85.84, C85.85, C85.86, C85.87, C85.88, C85.89, C85.9, C85.90, C85.91, C85.92, C85.93, C85.94, C85.95, C85.96, C85.97, C85.98, C85.99, C86, C86.0, C86.1, C86.2, C86.3, C86.4, C86.5, C86.6, C96, C96.0, C96.1, C96.2, C96.3, C96.4, C96.5, C96.6, C96.7, C96.8, C96.9	202.61, 202.62, 202.63, 202.64, 202.65, 202.66, 202.67, 202.68, 202.7, 202.70, 202.71, 202.72, 202.73, 202.74, 202.75, 202.76, 202.77, 202.78, 202.8, 202.80, 202.81, 202.82, 202.83, 202.84, 202.85, 202.86, 202.87, 202.88, 202.9, 202.90, 202.91, 202.92, 202.93, 202.94, 202.95, 202.96, 202.97, 202.98
Multiple myeloma	NA	C88, C88.0, C88.00, C88.01, C88.1, C88.2, C88.20, C88.3, C88.4, C88.40, C88.7, C88.70, C88.71, C88.8, C88.9, C89, C90, C90.0, C90.00, C90.01, C90.02, C90.1, C90.10, C90.11, C90.12, C90.2, C90.20, C90.21, C90.22, C90.3, C90.30, C90.31,	203, 203.0, 203.00, 203.01, 203.02, 203.1, 203.10, 203.11, 203.12, 203.8, 203.80, 203.81, 203.82, 203.9

Cause	ICCC3	ICD-10	ICD-9
		C90.32, C90.4, C90.5, C90.6, C90.7, C90.8, C90.9	
Acute lymphoid leukaemia	Ia, Ia1, Ia2, Ia3, Ia4	C91.0, C91.00, C91.01, C91.02, C91.2, C91.3, C91.30, C91.31, C91.32, C91.6, C91.60, C91.61, C91.62	204.0, 204.00, 204.01, 204.02, 204.2, 204.20, 204.21, 204.22
Chronic lymphoid leukaemia*	Custom mapping	Custom mapping	Custom mapping
Acute myeloid leukaemia	Ib	C92.0, C92.00, C92.01, C92.02, C92.3, C92.30, C92.31, C92.32, C92.4, C92.40, C92.41, C92.42, C92.5, C92.50, C92.51, C92.52, C92.6, C92.60, C92.61, C92.62, C93.0, C93.00, C93.01, C93.02, C94.0, C94.00, C94.01, C94.02, C94.2, C94.20, C94.21, C94.22, C94.4, C94.40, C94.41, C94.42, C94.5	205.0, 205.00, 205.01, 205.02, 205.2, 205.20, 205.21, 205.22, 205.3, 205.30, 205.31, 205.32, 206.0, 206.00, 206.01, 206.02, 207.0, 207.00, 207.01, 207.02, 207.20, 207.8, 207.80, 207.81, 207.82
Chronic myeloid leukaemia	Ic	C92.1, C92.10, C92.11, C92.12, C92.2, C92.20, C92.21, C92.22	205.1, 205.10, 205.11, 205.12
Other leukaemia	Ie	C91.20, C91.70, C92.70, C92.80, C93, C93.1, C93.10, C93.11, C93.12, C93.3, C93.30, C93.31, C93.32, C93.8, C94, C94.1, C94.3, C94.30, C94.31, C94.32, C94.50, C94.6, C94.60, C94.7, C94.70, C94.8, C94.80, C94.81, C94.82, C95, C95.0, C95.00, C95.01, C95.02, C95.1, C95.10, C95.11, C95.12, C95.2, C95.4, C95.6, C95.7, C95.70, C95.9, C95.90, C95.91, C95.92	205.92, 206.1, 206.10, 206.11, 206.12, 207, 207.1, 207.10, 207.11, 207.12, 207.2, 207.21, 207.22, 207.9, 208, 208.0, 208.00, 208.01, 208.02, 208.1, 208.10, 208.11, 208.12, 208.2, 208.20, 208.21, 208.22, 208.4, 208.7, 208.8, 208.80, 208.81, 208.82, 208.9, 208.90, 208.91, 208.92
Other malignant neoplasms	VIII, VIIIa, VIIIb, VIIIc, VIIIc1, VIIIc2, VIIId, VIIId1, VIIId2, VIIId3, VIIId4, VIIIe, XIf9, V, IVa, IVb, XIIa1, XIIa4, XIIa6, XIIb, XIa, XIf10, XIf11, XIf5, Xb, Xb1, Xb2, Xb3, Xb4, Xb5, Xb6, IX, IXa, IXb, IXb1, IXb2, IXb3, IXd, IXd1, IXd10, IXd11, IXd2, IXd3, IXd4, IXd5, IXd6, IXd7, IXd8, IXd9, IXe	C40, C40.0, C40.00, C40.01, C40.02, C40.1, C40.10, C40.11, C40.12, C40.2, C40.20, C40.21, C40.22, C40.3, C40.30, C40.31, C40.32, C40.8, C40.80, C40.81, C40.82, C40.9, C40.90, C40.91, C40.92, C41, C41.0, C41.01, C41.02, C41.1, C41.2, C41.3, C41.4, C41.5, C41.6, C41.7, C41.8, C41.9, C42.0, C42.1, C42.2, C42.3, C42.4, C69.0, C69.00, C69.01, C69.02, C69.1, C69.10, C69.11, C69.12, C69.3, C69.30, C69.31, C69.32, C69.4, C69.40, C69.41, C69.42, C69.5, C69.50, C69.51, C69.52, C69.6, C69.60, C69.61, C69.62, C69.7, C69.8, C69.80, C69.81, C69.82, C69.2, C69.20, C69.21, C69.22, C47, C47.0, C47.1, C47.10, C47.11, C47.12, C47.2, C47.20, C47.21, C47.22, C47.3, C47.4, C47.5, C47.6, C47.8, C47.9, C74.90, C17, C17.0, C17.1, C17.2, C17.3, C17.8, C17.9, C3, C30, C30.0, C30.1, C30.2, C30.3, C30.5, C30.8, C30.9,	170, 170.0, 170.1, 170.2, 170.3, 170.4, 170.5, 170.6, 170.7, 170.8, 170.9, 190, 190.0, 190.1, 190.2, 190.3, 190.4, 190.6, 190.7, 190.8, 190.5, 152, 152.0, 152.1, 152.2, 152.3, 152.4, 152.6, 152.8, 152.9, 158, 158.0, 158.3, 158.4, 158.5, 158.6, 158.8, 158.9, 160, 160.0, 160.1, 160.2, 160.3, 160.4, 160.5, 160.6, 160.8, 160.9, 163, 163.0, 163.1, 163.3, 163.5, 163.8, 163.9, 164, 164.0, 164.1, 164.2, 164.3, 164.8, 164.9, 181, 181.0, 181.9, 183.2, 183.3, 183.4, 183.5, 183.8, 184.0, 184.1, 184.2, 184.3, 184.4, 184.8, 187.1, 187.2, 187.3, 187.4, 187.5, 187.6, 187.7, 187.8, 189.2, 189.3, 189.4, 189.8, 194.1, 194.3, 194.4, 194.5, 194.6, 194.8, 209.0, 209.00, 209.01, 209.02, 209.03, 209.22, 209.25, 209.26, 209.27, 209.31, 209.32, 209.33, 209.34, 209.35, 209.36, 171, 171.0, 171.2, 171.3, 171.4, 171.5, 171.6, 171.7, 171.8, 171.9

Cause	ICCC3	ICD-10	ICD-9
		C31, C31.0, C31.1, C31.2, C31.3, C31.8, C31.9, C37, C37.0, C37.1, C37.2, C37.3, C37.9, C38, C38.0, C38.1, C38.2, C38.3, C38.4, C38.8, C4, C48, C48.0, C48.1, C48.2, C48.8, C48.9, C4A, C5, C51, C51.0, C51.1, C51.2, C51.8, C51.9, C52, C52.0, C52.9, C57, C57.0, C57.00, C57.01, C57.02, C57.1, C57.10, C57.11, C57.12, C57.2, C57.20, C57.21, C57.22, C57.3, C57.4, C57.7, C57.8, C58, C58.0, C58.9, C60, C60.0, C60.1, C60.2, C60.8, C60.9, C63, C63.0, C63.00, C63.01, C63.02, C63.1, C63.10, C63.11, C63.12, C63.2, C63.7, C63.8, C66, C66.0, C66.1, C66.2, C66.9, C68.0, C68.1, C68.8, C7, C75, C75.0, C75.1, C75.2, C75.3, C75.4, C75.5, C75.6, C75.8, C49, C49.0, C49.1, C49.10, C49.11, C49.12, C49.2, C49.20, C49.21, C49.22, C49.3, C49.4, C49.5, C49.6, C49.8, C49.9	

Abbreviations: ICCC3, International Classification of Childhood Cancer, Third Edition; ICD-9, International Classification of Diseases, Ninth Revision; ICD-10, International Classification of Diseases, Tenth Revision; NA, not applicable.

*Chronic lymphoid leukaemia is only modeled for ages 20 years and above in GBD. ICD codes (ICD-9: 204.1, 204.10, 204.11, and 204.12; ICD-10: C91.1, C91.10, C91.11, and C91.12) under 20 years are redistributed (see Section "6. Redistribution" on pg. 24 for more information) to "Acute lymphoid leukaemia", while these ICD codes over 20 years old are mapped directly to "Chronic lymphoid leukaemia".

Appendix Table 2: List of International Classification of Diseases (ICD) codes mapped to the Global Burden of Disease cause list for cancer mortality data

Cause	ICCC3	ICD-10	ICD-9
Lip and oral cavity cancer	XIf1	C00, C00.0, C00.1, C00.2, C00.3, C00.4, C00.5, C00.6, C00.8, C00.9, C01, C01.9, C02, C02.0, C02.1, C02.2, C02.3, C02.4, C02.8, C02.9, C03, C03.0, C03.1, C03.9, C04, C04.0, C04.1, C04.8, C04.9, C05, C05.0, C05.1, C05.2, C05.8, C05.9, C06, C06.0, C06.1, C06.2, C06.8, C06.80, C06.89, C06.9, C07, C07.0, C07.9, C08, C08.0, C08.1, C08.8, C08.9, D00.00, D00.01, D00.02, D00.03, D00.04, D00.05, D00.06, D00.07, D10.0, D10.1, D10.2, D10.3, D10.30, D10.39, D10.4, D10.5, D11, D11.0, D11.7, D11.9, D37.01, D37.02, D37.03, D37.030, D37.031, D37.032, D37.039, D37.04, D37.09	140, 140.0, 140.1, 140.2, 140.3, 140.4, 140.5, 140.6, 140.7, 140.8, 140.9, 141, 141.0, 141.1, 141.2, 141.3, 141.4, 141.5, 141.6, 141.8, 141.9, 142, 142.0, 142.1, 142.2, 142.3, 142.8, 142.9, 143, 143.0, 143.1, 143.8, 143.9, 144, 144.0, 144.1, 144.4, 144.8, 144.9, 145, 145.0, 145.1, 145.2, 145.3, 145.4, 145.5, 145.6, 145.8, 145.9, 210, 210.0, 210.1, 210.2, 210.3, 210.4, 210.5, 210.6, 235, 235.0
Nasopharynx cancer	XIc	C11, C11.0, C11.1, C11.2, C11.3, C11.8, C11.9, D00.08, D10.6, D37.05	147, 147.0, 147.1, 147.2, 147.3, 147.8, 147.9, 210.7, 210.8, 210.9

Cause	ICCC3	ICD-10	ICD-9
Other pharynx cancer	NA	C09, C09.0, C09.1, C09.8, C09.9, C1, C10, C10.0, C10.1, C10.2, C10.3, C10.4, C10.8, C10.9, C12, C12.0, C12.9, C13, C13.0, C13.1, C13.2, C13.8, C13.9, D10.7	146, 146.0, 146.1, 146.2, 146.3, 146.4, 146.5, 146.6, 146.7, 146.8, 146.9, 148, 148.0, 148.1, 148.2, 148.3, 148.4, 148.5, 148.8, 148.9
Oesophageal cancer	NA	C15, C15.0, C15.1, C15.2, C15.3, C15.4, C15.5, C15.8, C15.9, D00.1, D13.0	150, 150.0, 150.1, 150.2, 150.3, 150.4, 150.5, 150.6, 150.7, 150.8, 150.9, 211, 211.0, 230.1
Stomach cancer	NA	C16, C16.0, C16.1, C16.2, C16.3, C16.4, C16.5, C16.6, C16.7, C16.8, C16.9, D00.2, D13.1, D37.1	151, 151.0, 151.1, 151.2, 151.3, 151.4, 151.5, 151.6, 151.8, 151.9, 209.23, 209.63, 211.1, 230.2
Colon and rectum cancer	XIf2, XIf3	C18, C18.0, C18.1, C18.2, C18.3, C18.4, C18.5, C18.6, C18.7, C18.8, C18.9, C19, C19.0, C19.9, C2, C20, C20.0, C20.8, C20.9, C21, C21.0, C21.1, C21.2, C21.8, C21.9, D01.0, D01.1, D01.2, D01.3, D12, D12.0, D12.1, D12.2, D12.3, D12.4, D12.5, D12.6, D12.7, D12.8, D12.9, D37.3, D37.4, D37.5	153, 153.0, 153.1, 153.2, 153.3, 153.4, 153.5, 153.6, 153.7, 153.8, 153.9, 154, 154.0, 154.1, 154.2, 154.3, 154.4, 154.8, 154.9, 209.1, 209.10, 209.11, 209.12, 209.13, 209.14, 209.15, 209.16, 209.17, 209.5, 209.50, 209.51, 209.52, 209.53, 209.54, 209.55, 209.56, 209.57, 211.3, 211.4, 230.3, 230.4, 230.5, 230.6, 569.0, 569.43, 569.44, 569.84, 569.85
Liver cancer	VIIb, VIIc	C22, C22.0, C22.1, C22.3, C22.4, C22.5, C22.7, C22.8, D13.4	155, 155.0, 155.1, 155.3, 155.5, 155.9, 211.5
Gallbladder and biliary tract cancer	NA	C23, C23.0, C23.9, C24, C24.0, C24.1, C24.4, C24.8, C24.9, D13.5	156, 156.0, 156.1, 156.2, 156.3, 156.8, 156.9, 209.65, 209.66, 209.67
Pancreatic cancer	XIIa2	C25, C25.0, C25.1, C25.2, C25.3, C25.4, C25.7, C25.8, C25.9, D13.6, D13.7	157, 157.0, 157.1, 157.2, 157.3, 157.4, 157.5, 157.7, 157.8, 157.9, 211.6, 211.7
Larynx cancer	NA	C32, C32.0, C32.1, C32.2, C32.3, C32.8, C32.9, D02.0, D14.1, D38.0	161, 161.0, 161.1, 161.2, 161.3, 161.8, 161.9, 212.1, 231, 231.0, 235.6
Tracheal, bronchus, and lung cancer	XIIa3, XIf4	C33, C33.0, C33.2, C33.9, C34, C34.0, C34.00, C34.01, C34.02, C34.1, C34.10, C34.11, C34.12, C34.2, C34.3, C34.30, C34.31, C34.32, C34.4, C34.7, C34.8, C34.80, C34.81, C34.82, C34.9, C34.90, C34.91, C34.92, D02.1, D02.2, D02.20, D02.21, D02.22, D02.3, D14.2, D14.3, D14.30, D14.31, D14.32, D38.1	162, 162.0, 162.1, 162.2, 162.3, 162.4, 162.5, 162.8, 162.9, 209.21, 209.61, 212.2, 212.3, 231.1, 231.2, 235.7
Malignant skin melanoma	XId	C43, C43.0, C43.1, C43.10, C43.11, C43.12, C43.2, C43.20, C43.21, C43.22, C43.3, C43.30, C43.31, C43.39, C43.4, C43.5, C43.51, C43.52, C43.59, C43.6, C43.60, C43.61, C43.62, C43.7, C43.70, C43.71, C43.72, C43.8, C43.9, D03, D03.0, D03.1, D03.10, D03.11, D03.12, D03.2, D03.20, D03.21, D03.22, D03.3, D03.30, D03.39, D03.4, D03.5, D03.51, D03.52, D03.59, D03.6, D03.60, D03.61, D03.62, D03.7, D03.70, D03.71, D03.72, D03.8, D03.9, D22, D22.0, D22.1, D22.10, D22.11, D22.12, D22.2, D22.20, D22.21, D22.22, D22.3, D22.30, D22.39, D22.4, D22.5, D22.6, D22.60, D22.61, D22.62, D22.7, D22.70, D22.71, D22.72, D22.9, D23, D23.0, D23.1, D23.10, D23.11, D23.12, D23.2, D23.20, D23.21, D23.22,	172, 172.0, 172.1, 172.2, 172.3, 172.4, 172.5, 172.6, 172.7, 172.8, 172.9

Cause	ICCC3	ICD-10	ICD-9
		D23.3, D23.30, D23.39, D23.4, D23.5, D23.6, D23.60, D23.61, D23.62, D23.7, D23.70, D23.71, D23.72, D23.9	
Breast cancer	XIf6	C50, C50.0, C50.01, C50.011, C50.012, C50.019, C50.02, C50.021, C50.022, C50.029, C50.1, C50.11, C50.111, C50.112, C50.119, C50.12, C50.121, C50.122, C50.129, C50.2, C50.21, C50.211, C50.212, C50.219, C50.22, C50.221, C50.222, C50.229, C50.3, C50.31, C50.311, C50.312, C50.319, C50.32, C50.321, C50.322, C50.329, C50.4, C50.41, C50.411, C50.412, C50.419, C50.42, C50.421, C50.422, C50.429, C50.5, C50.51, C50.511, C50.512, C50.519, C50.52, C50.521, C50.522, C50.529, C50.6, C50.61, C50.611, C50.612, C50.619, C50.62, C50.621, C50.622, C50.629, C50.7, C50.8, C50.81, C50.811, C50.812, C50.819, C50.82, C50.821, C50.822, C50.829, C50.9, C50.91, C50.911, C50.912, C50.919, C50.92, C50.921, C50.922, C50.929, D05, D05.0, D05.00, D05.01, D05.02, D05.1, D05.10, D05.11, D05.12, D05.7, D05.8, D05.80, D05.81, D05.82, D05.9, D05.90, D05.91, D05.92, D24, D24.0, D24.1, D24.2, D24.9, D48.6, D48.60, D48.61, D48.62, D49.3	174, 174.0, 174.1, 174.2, 174.3, 174.4, 174.5, 174.6, 174.8, 174.9, 175, 175.0, 175.3, 175.9, 217, 217.0, 217.8, 233, 233.0, 238.3, 239.3, 610, 610.0, 610.1, 610.2, 610.3, 610.4, 610.8, 610.9
Cervical cancer	XIf7	C53, C53.0, C53.1, C53.3, C53.4, C53.8, C53.9, D06, D06.0, D06.1, D06.7, D06.9, D26.0	180, 180.0, 180.1, 180.2, 180.3, 180.4, 180.5, 180.6, 180.8, 180.9, 219, 219.0, 233.1, 622.1, 622.10, 622.11, 622.12, 622.2, 622.7
Uterine cancer	NA	C54, C54.0, C54.1, C54.2, C54.3, C54.4, C54.8, C54.9, D07.0, D07.1, D07.2, D26.1, D26.7, D26.9	182, 182.0, 182.1, 182.8, 182.9, 233.2
Ovarian cancer	NA	C56, C56.0, C56.1, C56.2, C56.4, C56.9, D27, D27.0, D27.1, D27.9, D39.1, D39.10, D39.11, D39.12	183, 183.0, 220, 220.0, 220.9, 236.2
Prostate cancer	NA	C61, C61.0, C61.9, D07.5, D29.1, D40.0	185, 185.0, 185.9, 222.2, 236.5
Testicular cancer	NA	C62, C62.0, C62.00, C62.01, C62.02, C62.1, C62.10, C62.11, C62.12, C62.9, C62.90, C62.91, C62.92, D29.2, D29.20, D29.21, D29.22, D29.3, D29.30, D29.31, D29.32, D29.4, D29.7, D29.8, D40.1, D40.10, D40.11, D40.12, D40.7, D40.8	186, 186.0, 186.9, 222, 222.0, 222.3, 236.4
Kidney cancer	VI, VIa, VIa1, VIa2, VIa3, VIa4, VIb, VIc	C64, C64.0, C64.1, C64.2, C64.4, C64.5, C64.6, C64.8, C64.9, C65, C65.0, C65.1, C65.2, C65.9, D30.0, D30.00, D30.01, D30.02, D30.1, D30.10, D30.11, D30.12, D41.0, D41.00, D41.01, D41.02, D41.1, D41.10, D41.11, D41.12	189.0, 189.1, 189.5, 189.6, 209.24, 209.64, 223, 223.0, 223.1, 236.91

Cause	ICCC3	ICD-10	ICD-9
Bladder cancer	XIf8	C67, C67.0, C67.1, C67.2, C67.3, C67.4, C67.5, C67.6, C67.7, C67.8, C67.9, D09.0, D30.3, D41.4, D41.7, D41.8, D49.4	188, 188.0, 188.1, 188.2, 188.3, 188.4, 188.5, 188.6, 188.7, 188.8, 188.9, 223.3, 233.7, 236.7, 239.4
Brain and central nervous system cancer	III, IIIa, IIIa1, IIIa2, IIIb, IIIc, IIIc1, IIIc2, IIIc3, IIIc4, IIId, IIId1, IIId2, IIId3, IIIe, IIIe1, IIIe2, IIIe3, IIIe4, IIIe5, IIIf, Xa, Xa1, Xa2, Xa3, Xa4, Xa5, Xa6	C70, C70.0, C70.1, C70.5, C70.6, C70.9, C71, C71.0, C71.1, C71.2, C71.3, C71.4, C71.5, C71.6, C71.7, C71.8, C71.9, C72, C72.0, C72.1, C72.2, C72.20, C72.21, C72.22, C72.3, C72.30, C72.31, C72.32, C72.4, C72.40, C72.41, C72.42, C72.5, C72.50, C72.59, C72.8, C72.9	191, 191.0, 191.1, 191.2, 191.3, 191.4, 191.5, 191.6, 191.7, 191.8, 191.9, 192, 192.0, 192.1, 192.2, 192.3, 192.4, 192.8, 192.9
Thyroid cancer	XIb	C73, C73.0, C73.1, C73.2, C73.3, C73.4, C73.5, C73.8, C73.9, D09.3, D09.8, D34, D34.0, D34.9, D44.0	193, 193.0, 193.2, 193.9, 226, 226.0, 226.9
Mesothelioma	XIIa5	C45, C45.0, C45.1, C45.2, C45.3, C45.4, C45.5, C45.6, C45.7, C45.8, C45.9	NA
Hodgkin lymphoma	IIa	C81, C81.0, C81.00, C81.01, C81.02, C81.03, C81.04, C81.05, C81.06, C81.07, C81.08, C81.09, C81.1, C81.10, C81.11, C81.12, C81.13, C81.14, C81.15, C81.16, C81.17, C81.18, C81.19, C81.2, C81.20, C81.21, C81.22, C81.23, C81.24, C81.25, C81.26, C81.27, C81.28, C81.29, C81.3, C81.30, C81.31, C81.32, C81.33, C81.34, C81.35, C81.36, C81.37, C81.38, C81.39, C81.4, C81.40, C81.41, C81.42, C81.43, C81.44, C81.45, C81.46, C81.47, C81.48, C81.49, C81.5, C81.6, C81.7, C81.70, C81.71, C81.72, C81.73, C81.74, C81.75, C81.76, C81.77, C81.78, C81.79, C81.8, C81.9, C81.90, C81.91, C81.92, C81.93, C81.94, C81.95, C81.96, C81.97, C81.98, C81.99	201, 201.0, 201.00, 201.01, 201.02, 201.03, 201.04, 201.05, 201.06, 201.07, 201.08, 201.1, 201.10, 201.11, 201.12, 201.13, 201.14, 201.15, 201.16, 201.17, 201.18, 201.2, 201.20, 201.21, 201.22, 201.23, 201.24, 201.25, 201.26, 201.27, 201.28, 201.4, 201.40, 201.41, 201.42, 201.43, 201.44, 201.45, 201.46, 201.47, 201.48, 201.5, 201.50, 201.51, 201.52, 201.53, 201.54, 201.55, 201.56, 201.57, 201.58, 201.6, 201.60, 201.61, 201.62, 201.63, 201.64, 201.65, 201.66, 201.67, 201.68, 201.7, 201.70, 201.71, 201.72, 201.73, 201.74, 201.75, 201.76, 201.77, 201.78, 201.9, 201.90, 201.91, 201.92, 201.93, 201.94, 201.95, 201.96, 201.97, 201.98
Non-Hodgkin lymphoma	IIb, IIb1, IIb2, IIb3, IIb4, IIc, IId, IIe	C83.7, C83.70, C83.71, C83.72, C83.73, C83.74, C83.75, C83.76, C83.77, C83.78, C83.79, C83.8, C82, C82.0, C82.00, C82.01, C82.02, C82.03, C82.04, C82.05, C82.06, C82.07, C82.08, C82.09, C82.1, C82.10, C82.11, C82.12, C82.13, C82.14, C82.15, C82.16, C82.17, C82.18, C82.19, C82.2, C82.20, C82.21, C82.22, C82.23, C82.24, C82.25, C82.26, C82.27, C82.28, C82.29, C82.3, C82.30, C82.31, C82.32, C82.33, C82.34, C82.35, C82.36, C82.37, C82.38, C82.39, C82.4, C82.40, C82.41, C82.42, C82.43, C82.44, C82.45, C82.46, C82.47, C82.48, C82.49, C82.5, C82.50, C82.51, C82.52, C82.53, C82.54, C82.55, C82.56, C82.57, C82.58, C82.59, C82.6,	200.2, 200.20, 200.21, 200.22, 200.23, 200.24, 200.25, 200.26, 200.27, 200.28, 200, 200.0, 200.00, 200.01, 200.02, 200.03, 200.04, 200.05, 200.06, 200.07, 200.08, 200.1, 200.10, 200.11, 200.12, 200.13, 200.14, 200.15, 200.16, 200.17, 200.18, 200.3, 200.30, 200.31, 200.32, 200.33, 200.34, 200.35, 200.36, 200.37, 200.38, 200.4, 200.40, 200.41, 200.42, 200.43, 200.44, 200.45, 200.46, 200.47, 200.48, 200.5, 200.50, 200.51, 200.52, 200.53, 200.54, 200.55, 200.56, 200.57, 200.58, 200.6, 200.60, 200.61, 200.62, 200.63, 200.64, 200.65, 200.66, 200.67, 200.68, 200.7, 200.70, 200.71, 200.72, 200.73, 200.74, 200.75, 200.76, 200.77,

Cause	ICCC3	ICD-10	ICD-9
		C82.60, C82.61, C82.62, C82.63, C82.64, C82.65, C82.66, C82.67, C82.68, C82.69, C82.7, C82.8, C82.80, C82.81, C82.82, C82.83, C82.84, C82.85, C82.86, C82.87, C82.88, C82.89, C82.9, C82.90, C82.91, C82.92, C82.93, C82.94, C82.95, C82.96, C82.97, C82.98, C82.99, C83, C83.0, C83.00, C83.01, C83.02, C83.03, C83.04, C83.05, C83.06, C83.07, C83.08, C83.09, C83.1, C83.10, C83.11, C83.12, C83.13, C83.14, C83.15, C83.16, C83.17, C83.18, C83.19, C83.2, C83.3, C83.30, C83.31, C83.32, C83.33, C83.34, C83.35, C83.36, C83.37, C83.38, C83.39, C83.4, C83.5, C83.50, C83.51, C83.52, C83.53, C83.54, C83.55, C83.56, C83.57, C83.58, C83.59, C83.6, C83.80, C83.81, C83.82, C83.83, C83.84, C83.85, C83.86, C83.87, C83.88, C83.89, C83.9, C83.90, C83.91, C83.92, C83.93, C83.94, C83.95, C83.96, C83.97, C83.98, C83.99, C84, C84.0, C84.00, C84.01, C84.02, C84.03, C84.04, C84.05, C84.06, C84.07, C84.08, C84.09, C84.1, C84.10, C84.11, C84.12, C84.13, C84.14, C84.15, C84.16, C84.17, C84.18, C84.19, C84.2, C84.3, C84.4, C84.40, C84.41, C84.42, C84.43, C84.44, C84.45, C84.46, C84.47, C84.48, C84.49, C84.5, C84.6, C84.60, C84.61, C84.62, C84.63, C84.64, C84.65, C84.66, C84.67, C84.68, C84.69, C84.7, C84.70, C84.71, C84.72, C84.73, C84.74, C84.75, C84.76, C84.77, C84.78, C84.79, C84.8, C84.9, C84.90, C84.91, C84.92, C84.93, C84.94, C84.95, C84.96, C84.97, C84.98, C84.99, C85, C85.0, C85.1, C85.10, C85.11, C85.12, C85.13, C85.14, C85.15, C85.16, C85.17, C85.18, C85.19, C85.2, C85.20, C85.21, C85.22, C85.23, C85.24, C85.25, C85.26, C85.27, C85.28, C85.29, C85.3, C85.4, C85.5, C85.6, C85.7, C85.8, C85.80, C85.81, C85.82, C85.83, C85.84, C85.85, C85.86, C85.87, C85.88, C85.89, C85.9, C85.90, C85.91, C85.92, C85.93, C85.94, C85.95, C85.96, C85.97, C85.98, C85.99, C86, C86.0, C86.1, C86.2, C86.3, C86.4, C86.5, C86.6, C96, C96.0, C96.1, C96.2, C96.3, C96.4, C96.5, C96.6, C96.7, C96.8, C96.9	200.78, 200.8, 200.80, 200.81, 200.82, 200.83, 200.84, 200.85, 200.86, 200.87, 200.88, 200.9, 202, 202.0, 202.00, 202.01, 202.02, 202.03, 202.04, 202.05, 202.06, 202.07, 202.08, 202.1, 202.10, 202.11, 202.12, 202.13, 202.14, 202.15, 202.16, 202.17, 202.18, 202.2, 202.20, 202.21, 202.22, 202.23, 202.24, 202.25, 202.26, 202.27, 202.28, 202.3, 202.30, 202.31, 202.32, 202.33, 202.34, 202.35, 202.36, 202.37, 202.38, 202.4, 202.40, 202.41, 202.42, 202.43, 202.44, 202.45, 202.46, 202.47, 202.48, 202.5, 202.50, 202.51, 202.52, 202.53, 202.54, 202.55, 202.56, 202.57, 202.58, 202.6, 202.60, 202.61, 202.62, 202.63, 202.64, 202.65, 202.66, 202.67, 202.68, 202.7, 202.70, 202.71, 202.72, 202.73, 202.74, 202.75, 202.76, 202.77, 202.78, 202.8, 202.80, 202.81, 202.82, 202.83, 202.84, 202.85, 202.86, 202.87, 202.88, 202.9, 202.90, 202.91, 202.92, 202.93, 202.94, 202.95, 202.96, 202.97, 202.98
Multiple myeloma	NA	C88, C88.0, C88.00, C88.01, C88.1, C88.2, C88.20, C88.3, C88.4, C88.40, C88.7, C88.70, C88.71, C88.8, C88.9, C89, C90, C90.0, C90.00, C90.01, C90.02, C90.1, C90.10, C90.11, C90.12, C90.2, C90.20, C90.21, C90.22, C90.3, C90.30,	203, 203.0, 203.00, 203.01, 203.02, 203.1, 203.10, 203.11, 203.12, 203.8, 203.80, 203.81, 203.82, 203.9

Cause	ICCC3	ICD-10	ICD-9
		C90.31, C90.32, C90.4, C90.5, C90.6, C90.7, C90.8, C90.9	
Acute lymphoid leukaemia	Ia, Ia1, Ia2, Ia3, Ia4	C91.0, C91.00, C91.01, C91.02, C91.2, C91.3, C91.30, C91.31, C91.32, C91.6, C91.60, C91.61, C91.62	204.0, 204.00, 204.01, 204.02, 204.2, 204.20, 204.21, 204.22
Chronic lymphoid leukaemia*	Custom mapping	Custom mapping	Custom mapping
Acute myeloid leukaemia	Ib	C92.0, C92.00, C92.01, C92.02, C92.3, C92.30, C92.31, C92.32, C92.4, C92.40, C92.41, C92.42, C92.5, C92.50, C92.51, C92.52, C92.6, C92.60, C92.61, C92.62, C93.0, C93.00, C93.01, C93.02, C94.0, C94.00, C94.01, C94.02, C94.2, C94.20, C94.21, C94.22, C94.4, C94.40, C94.41, C94.42, C94.5	205.0, 205.00, 205.01, 205.02, 205.2, 205.20, 205.21, 205.22, 205.3, 205.30, 205.31, 205.32, 206.0, 206.00, 206.01, 206.02, 207.0, 207.00, 207.01, 207.02, 207.20, 207.8, 207.80, 207.81, 207.82
Chronic myeloid leukaemia	Ic	C92.1, C92.10, C92.11, C92.12, C92.2, C92.20, C92.21, C92.22	205.1, 205.10, 205.11, 205.12
Other leukaemia	Ie	C91.20, C91.70, C92.70, C92.80, C93, C93.1, C93.10, C93.11, C93.12, C93.3, C93.30, C93.31, C93.32, C93.8, C94, C94.1, C94.3, C94.30, C94.31, C94.32, C94.50, C94.6, C94.60, C94.7, C94.70, C94.8, C94.80, C94.81, C94.82, C95, C95.0, C95.00, C95.01, C95.02, C95.1, C95.10, C95.11, C95.12, C95.2, C95.4, C95.6, C95.7, C95.70, C95.9, C95.90, C95.91, C95.92	205.92, 206.1, 206.10, 206.11, 206.12, 207, 207.1, 207.10, 207.11, 207.12, 207.2, 207.21, 207.22, 207.9, 208, 208.0, 208.00, 208.01, 208.02, 208.1, 208.10, 208.11, 208.12, 208.2, 208.20, 208.21, 208.22, 208.4, 208.7, 208.8, 208.80, 208.81, 208.82, 208.9, 208.90, 208.91, 208.92
Other malignant neoplasms	VIII, VIIIa, VIIIb, VIIIc, VIIIc1, VIIIc2, VIIId, VIIId1, VIIId2, VIIId3, VIIId4, VIIIe, XIf9, V, IVa, IVb, XIIa1, XIIa4, XIIa6, XIIb, XIa, XIf10, XIf11, XIf5, Xb, Xb1, Xb2, Xb3, Xb4, Xb5, Xb6, IX, IXa, IXb, IXb1, IXb2, IXb3, IXd, IXd1, IXd10, IXd11, IXd2, IXd3, IXd4, IXd5, IXd6,	C40, C40.0, C40.00, C40.01, C40.02, C40.1, C40.10, C40.11, C40.12, C40.2, C40.20, C40.21, C40.22, C40.3, C40.30, C40.31, C40.32, C40.8, C40.80, C40.81, C40.82, C40.9, C40.90, C40.91, C40.92, C41, C41.0, C41.01, C41.02, C41.1, C41.2, C41.3, C41.4, C41.5, C41.6, C41.7, C41.8, C41.9, C42.0, C42.1, C42.2, C42.3, C42.4, C69.0, C69.00, C69.01, C69.02, C69.1, C69.10, C69.11, C69.12, C69.3, C69.30, C69.31, C69.32, C69.4, C69.40, C69.41, C69.42, C69.5, C69.50, C69.51, C69.52, C69.6, C69.60, C69.61, C69.62, C69.7, C69.8, C69.80, C69.81, C69.82, C69.2, C69.20, C69.21, C69.22, C47, C47.0, C47.1, C47.10, C47.11, C47.12, C47.2, C47.20, C47.21, C47.22, C47.3, C47.4, C47.5, C47.6, C47.8, C47.9, C74.90, C17, C17.0, C17.1, C17.2, C17.3, C17.8, C17.9, C3, C30, C30.0, C30.1, C30.2, C30.3, C30.5, C30.8, C30.9, C31, C31.0, C31.1, C31.2, C31.3, C31.8, C31.9, C37, C37.0, C37.1, C37.2, C37.3, C37.9, C38, C38.0, C38.1, C38.2, C38.3, C38.4, C38.8, C4, C48, C48.0, C48.1, C48.2,	170, 170.0, 170.1, 170.2, 170.3, 170.4, 170.5, 170.6, 170.7, 170.8, 170.9, 190, 190.0, 190.1, 190.2, 190.3, 190.4, 190.6, 190.7, 190.8, 190.5, 152, 152.0, 152.1, 152.2, 152.3, 152.4, 152.6, 152.8, 152.9, 158, 158.0, 158.3, 158.4, 158.5, 158.6, 158.8, 158.9, 160, 160.0, 160.1, 160.2, 160.3, 160.4, 160.5, 160.6, 160.8, 160.9, 163, 163.0, 163.1, 163.3, 163.5, 163.8, 163.9, 164, 164.0, 164.1, 164.2, 164.3, 164.8, 164.9, 181, 181.0, 181.9, 183.2, 183.3, 183.4, 183.5, 183.8, 184.0, 184.1, 184.2, 184.3, 184.4, 184.8, 187.1, 187.2, 187.3, 187.4, 187.5, 187.6, 187.7, 187.8, 189.2, 189.3, 189.4, 189.8, 194.1, 194.3, 194.4, 194.5, 194.6, 194.8, 209.0, 209.00, 209.01, 209.02, 209.03, 209.22, 209.25, 209.26, 209.27, 209.31, 209.32, 209.33, 209.34, 209.35, 209.36, 209.4, 209.40, 209.41, 209.42, 209.43, 211.2, 211.8, 212.0, 212.4, 212.5, 212.6, 212.7, 212.8, 213, 213.0, 213.1, 213.2, 213.3, 213.4, 213.5, 213.6, 213.7, 213.8, 213.9, 221.0, 221.1, 221.2, 221.8, 222.1, 222.8, 223.2, 223.8, 223.81, 223.89, 224, 224.0, 224.1,

Cause	ICCC3	ICD-10	ICD-9
	IXd7, IXd8, IXd9, IXe	C48.8, C48.9, C4A, C5, C51, C51.0, C51.1, C51.2, C51.8, C51.9, C52, C52.0, C52.9, C57, C57.0, C57.00, C57.01, C57.02, C57.1, C57.10, C57.11, C57.12, C57.2, C57.20, C57.21, C57.22, C57.3, C57.4, C57.7, C57.8, C58, C58.0, C58.9, C60, C60.0, C60.1, C60.2, C60.8, C60.9, C63, C63.0, C63.00, C63.01, C63.02, C63.1, C63.10, C63.11, C63.12, C63.2, C63.7, C63.8, C66, C66.0, C66.1, C66.2, C66.9, C68.0, C68.1, C68.8, C7, C75, C75.0, C75.1, C75.2, C75.3, C75.4, C75.5, C75.6, C75.8, D07.4, D09.2, D09.20, D09.21, D09.22, D13.2, D13.3, D13.30, D13.39, D14.0, D15, D15.0, D15.1, D15.2, D15.7, D15.9, D16, D16.0, D16.00, D16.01, D16.02, D16.1, D16.10, D16.11, D16.12, D16.2, D16.20, D16.21, D16.22, D16.3, D16.30, D16.31, D16.32, D16.4, D16.5, D16.6, D16.7, D16.8, D16.9, D28.0, D28.1, D28.7, D29.0, D30.2, D30.20, D30.21, D30.22, D30.4, D30.7, D30.8, D31, D31.0, D31.00, D31.01, D31.02, D31.1, D31.10, D31.11, D31.12, D31.2, D31.20, D31.21, D31.22, D31.3, D31.30, D31.31, D31.32, D31.4, D31.40, D31.41, D31.42, D31.5, D31.50, D31.51, D31.52, D31.6, D31.60, D31.61, D31.62, D31.9, D31.90, D31.91, D31.92, D32, D32.0, D32.1, D32.9, D33, D33.0, D33.1, D33.2, D33.3, D33.4, D33.7, D33.9, D35, D35.0, D35.00, D35.01, D35.02, D35.1, D35.2, D35.3, D35.4, D35.5, D35.6, D35.7, D35.8, D35.9, D36, D36.1, D36.10, D36.11, D36.12, D36.13, D36.14, D36.15, D36.16, D36.17, D36.7, D37.2, D38.2, D38.3, D38.4, D38.5, D39.2, D39.8, D41.2, D41.20, D41.21, D41.22, D41.3, D42, D42.0, D42.1, D42.9, D43, D43.0, D43.1, D43.2, D43.3, D43.4, D43.7, D43.8, D43.9, D44.1, D44.10, D44.11, D44.12, D44.2, D44.3, D44.4, D44.5, D44.6, D44.7, D44.8, D48.0, D48.1, D48.2, D48.3, D48.4, D49.6, D49.81, C49, C49.0, C49.1, C49.10, C49.11, C49.12, C49.2, C49.20, C49.21, C49.22, C49.3, C49.4, C49.5, C49.6, C49.8, C49.9	224.2, 224.3, 224.4, 224.5, 224.6, 224.7, 224.8, 224.9, 225, 225.0, 225.1, 225.2, 225.3, 225.4, 225.8, 225.9, 227, 227.0, 227.1, 227.3, 227.4, 227.5, 227.6, 227.8, 227.9, 228, 228.0, 228.00, 228.01, 228.02, 228.03, 228.04, 228.09, 228.1, 228.9, 229.0, 229.8, 230.7, 230.8, 233.31, 233.32, 233.4, 233.5, 234.0, 234.5, 234.8, 235.4, 235.8, 236.1, 236.99, 237, 237.0, 237.1, 237.2, 237.3, 237.5, 237.6, 237.7, 237.70, 237.71, 237.72, 237.73, 237.79, 237.9, 238.0, 238.1, 239.2, 239.6, 171, 171.0, 171.2, 171.3, 171.4, 171.5, 171.6, 171.7, 171.8, 171.9

Abbreviations: ICCC3, International Classification of Childhood Cancer, Third Edition; ICD-9, International Classification of Diseases, Ninth Revision; ICD-10, International Classification of Diseases, Tenth Revision; NA, not applicable.

*Chronic lymphoid leukaemia is only modeled for ages 20 years and above in GBD. ICD codes (ICD-9: 204.1, 204.10, 204.11, and 204.12; ICD-10: C91.1, C91.10, C91.11, and C91.12) under 20 years are redistributed (see Section "6. Redistribution" on pg. 24 for more information) to "Acute lymphoid leukaemia", while these ICD codes over 20 years old are mapped directly to "Chronic lymphoid leukaemia".

4. Age/sex splitting. In the fourth data processing step (#4 in in Appendix figure 1), cancer registry data are standardised to the GBD age groups. For each cancer, the minimum age group estimated was determined as the youngest age-group where SEER reported at least 50 cases over the period 1990 to 2015.[8] Global age-specific incidence rates are generated using hospital inpatient data as described in Section 4.3 of the Supplementary Appendix 1 to the GBD 2019 paper "Global burden of 369 diseases and injuries in 204 countries and territories, 1990–2019: a systematic analysis for the Global Burden of Disease Study 2019".[3] Reference age-specific mortality rates were generated using aggregated deaths from processed VR data, using the approach described in Section 2.5 of the aforementioned GBD 2019 paper.[3] For incidence or mortality datasets that require age-splitting, age-specific proportions are then generated by applying the reference age-specific rates to the registry population to produce the expected number of cases (or deaths for a mortality dataset) for that registry by age. The expected number of cases (or deaths) for each sex, age, and cancer were normalised to 1, creating final, age-specific proportions. These proportions were then applied to the total number of cases (or deaths) by sex and cancer to get the GBD age group-specific number of cases (or deaths) related to that dataset.

In the rare case that the cancer registry only contains data for both sexes combined, the age-specific cases or deaths are split and reassigned to separate sexes using the same weights that are used for the age-splitting process. Starting from the expected number of deaths, global proportions are generated by sex for each age. For example, if for ages 15-19 years old there are 6 expected deaths for males from cause of death data and 4 expected deaths for females, then 60% of the combined-sex deaths for ages 15-19 years would be assigned to males and the remaining 40% would be assigned to females.

5. Cause disaggregation. In the fifth step (#5 in Appendix figure 1), data for cause entries that are aggregates of GBD causes were redistributed across those GBD causes. Examples of these aggregated causes include some cancer registries reporting ICD-10 codes C00-C14 together as "lip, oral cavity, and pharyngeal cancer". These groups are broken down into subcauses that can be individually mapped to single GBD causes. In this example, the more specific ICD-10 codes within C00-C14 are "lip and oral cavity cancer" (C00-C08), "nasopharynx cancer" (C11), "cancer of other parts of the pharynx" (C09-C10, C12-C13), and "Malignant neoplasm of other and ill-defined sites in the lip, oral cavity, and pharynx" (C14). To redistribute the data, weights were created using the same "rate-applied-to-population" method employed in age-sex splitting (see step four above). For the undefined code (C14 in the example) an "average all cancer" weight was used, calculated on the high-quality cancer registry data from SEER/NORDCAN/CI5 by dividing the sum of the cases across these registries by the combined population across these registries. Then, proportions were generated by subcause for each aggregate cause as in the sex-splitting example above (see step four). The total number of cases from the aggregated group (C00-C14) was recalculated for each subgroup and the undefined code (C14). C14 was then redistributed as a "garbage code" in step six. For two exceptions, C44 (non-melanoma skin cancer) and C46 (Kaposi's sarcoma), fixed proportions were used to redistribute into GBD causes. C46 entries were primarily redistributed to HIV according to age (100% for age <15 years, 95% for age 15-49 years, and 90% for age ≥50 years), with the remainder redistributed to the GBD cancer cause "Other malignant neoplasms".

*6. Redistribution***.** In the sixth step (#6 in in Appendix figure 1), unspecified ICD codes ("garbage codes") such as "ill-defined cancer site" (for example, C76 or C80) are redistributed across relevant causes estimated within the GBD hierarchy. Redistribution of cancer registry incidence and mortality data mirrored the process of the redistribution used in the cause of death database and utilised the same redistribution maps as specified in Section 2.4 of the Supplementary Appendix 1 to the GBD 2019 Diseases and Injuries capstone, "Global burden of 369 diseases and injuries in 204 countries and territories, 1990–2019: a systematic analysis for the Global Burden of Disease Study 2019".[3] Sources and targets of garbage codes can be found in eTable 5 of the Supplementary Appendix to "Cancer Incidence, Mortality, Years of Life Lost, Years Lived with Disability, and Disability-Adjusted Life Years for 29

Cancer Groups from 2010 to 2019: A Systematic Analysis of Cancer Burden Globally, Nationally, and by Socio-demographic Index for the Global Burden of Disease Study 2019”.[1]

7. Removal of duplicates. In the seventh step (#7 in in Appendix figure 1), duplicate or redundant data sources were removed from the processed cancer registry dataset. Duplicate sources were present if, for example, a cancer registry was part of the CI5 database but we also had data from that registry directly. Redundancies occurred and were removed as described in “Cancer Incidence Data Sources”, where more detailed data were available, or when national registry data could replace regionally representative data. From here, two parallel selection processes were run; one to generate input data for the mortality-to-incidence ratio (MIR) models, and one to generate incidence for final mortality estimation. When creating the final incidence input, higher priority was given to registry data from the most standardised source; whereas for the MIR model input, only sources that reported both incidence and mortality were used.

8. Combine matching incidence and mortality data and model MIRs. In the eighth step (#8 in Appendix figure 1), the processed incidence and mortality data from cancer registries were matched by cancer cause, age, sex, year, and location to generate MIRs. The resulting MIRs were used as input for a three-step modelling approach using the general GBD spatiotemporal Gaussian process regression (ST-GPR)[22] approach, with the Healthcare Access and Quality (HAQ) Index as a covariate in the linear mixed effects model using logit transformed MIR as outcome.[23]

$$\text{logit}\left(MIR_{c,a,s,t}\right) = \alpha + \beta_1(HAQIndex_{c,t}) + \sum_{a}^{A} \beta_2 I_a + \beta_3 I_s + \epsilon_{c,a,s,t}$$

MIR: mortality-to-incidence ratio
c: country (or subnational for subnationally modeled locations), a: age group, t: time (years); s: sex
HAQ Index: Healthcare Access and Quality Index
I: indicator variable
$\epsilon_{c,a,s,t}$: error term

Information on ST-GPR can be found in “Section 4.3.3: Spatiotemporal Gaussian process regression (ST-GPR) modelling” in Supplementary Appendix 1 to “Global burden of 369 diseases and injuries in 204 countries and territories, 1990–2019: a systematic analysis for the global burden of disease study 2019”.[3] Predictions were made without the random effects. The ST-GPR model has three main hyper-parameters that control for smoothing across time, age, and geography.[3] These hyper-parameters were adjusted for GBD 2019 in order to improve model performance in locations with sparse data. The time adjustment parameter lambda (λ) aims to borrow strength from neighboring time points (ie, the value in this year is highly correlated with the value in the previous year but less so further back in time). For GBD 2019, lambda was lowered from 2 to 0.05, increasing the weight of more distant years. The age adjustment parameter omega (ω) borrows strength from data in neighboring age groups and was lowered from 1.0 to 0.5, increasing the weight of more distant age groups. The space adjustment parameter zeta (ξ) aims to borrow strength across the hierarchy of geographical locations. Zeta was lowered from 0.95 to 0.01, reducing the weight of more distant geographical data at the region or super region level. For the remaining parameters in the Gaussian process regression, we lowered the amplitude from 2 to 1 (reducing fluctuation from the mean function) and reduced the scale value from 15 to 10 (reducing the time distance over which points are correlated).

Data-cleaning steps for MIR estimation were similar to those for GBD 2017. For each cancer, MIRs from locations in HAQ Index quintiles 1-4 were dropped if they were below the median of MIRs from locations in HAQ Index quintile 5. We also dropped MIRs from locations in HAQ Index quintiles 1-4 if the MIRs were above an outlier threshold calculated as the third quartile + 1.5 * IQR (inter-quartile

range). We dropped all MIR data that were based on fewer than 15 incident cases to avoid excessive variation in the ratio due to small numbers (this threshold was 25 cases in GBD 2017, but was lowered in GBD 2019 in order to include additional data). An exception to this threshold was made for mesothelioma and acute myeloid leukaemia, where instead we dropped MIRs that were based on fewer than ten cases because of lower data availability for these two cancers. For the lower end of the age spectrum where cancers are generally rarer, we also aggregated incidence and mortality to the youngest five-year age bin where SEER[8] reported at least 50 cases from 1990 to 2015, to avoid unstable MIR predictions in young age groups because of too few data. The MIR estimates in this SEER-based minimum age-bin were then copied down to all younger GBD age groups estimated for that cancer.

Since MIRs can be above 1, especially in older age groups and cancers with low cure rates, we used the 95th percentile (by age group) of the cleaned dataset (detailed above) to cap the MIR input data. These "upper cap" values were used to allow MIRs over 1 in some age groups but to constrain the MIRs to a maximum level. Any MIR values over this cap were Winsorised to the cap value. To run the logit model, the input data were first divided by the upper caps to get proportional data ranging from 0 to 1. Model predictions from ST-GPR were then rescaled back by multiplying them by the upper caps. To constrain the MIRs at the lower end, we used the fifth percentile of the cancer and age-specific cleaned MIR input data to Winsorise all model predictions below this lower cap.

9. Generate mortality estimates from incidence and MIRs. Final estimated MIRs were matched with the cleaned cancer registry incidence dataset finalised in the ninth step (#9 in Appendix figure 1) to generate mortality estimates (#10 in Appendix figure 1):

$$MIR_{estimates} * \ incidence_{registry} = mortality_{CR\ inputs}$$

These mortality estimates were then smoothed by a Bayesian noise-reduction algorithm (to deal with zero counts; this is also applied to the VR and VA data), as specified in Section 2.14 of the Supplemenatary Appendix 1 to the GBD 2019 paper "Global burden of 369 diseases and injuries in 204 countries and territories, 1990–2019: a systematic analysis for the Global Burden of Disease Study 2019".[3] These data were uploaded into the CoD database as CR data (#11 in Appendix figure 1). Cancer-specific mortality modelling then followed the general CODEm process[24] using the totality of VA, VR, and CR data.

Cause of death database formatting

Formatting of data sources for the cause of death (CoD) database, including VR and VA data, is similar to many of the steps outlined above for CR data (#11 in Appendix figure 1) and is described in Section 2 of the Supplementary Appendix 1 to the GBD 2019 paper "Global burden of 369 diseases and injuries in 204 countries and territories, 1990–2019: a systematic analysis for the Global Burden of Disease Study 2019".[3]

VA data may not capture cancer deaths as accurately or comprehensively as cancer registries or vital registration systems, but provides a useful contribution to cancer models in locations without VR or CR data. Additional processing and restrictions are performed on VA to ensure quality standards and feasible inputs. More details on VA data processing are provided in the appendix noted above, particularly Sections 2.2 (VA overview), 2.10 (VA cause restrictions), 2.14 (noise reduction), 2.15 (outlier identification), and 2.16 (data quality ratings).

Appendix Table 3: Restrictions on age and sex by each cancer type in GBD 2019

Cause	Minimum age modelled in GBD 2019 (years)*	Maximum age modelled in GBD 2019 (years)*	Age range evaluated for AYA Cancer analysis, based on estimates available in GBD 2019 (years)	Sex restrictions
Acute lymphoid leukaemia	0	95+	15-39	None
Acute myeloid leukaemia	0	95+	15-39	None
Bladder cancer	15	95+	15-39	None
Brain and central nervous system cancer	0	95+	15-39	None
Breast cancer	15	95+	15-39	None
Cervical cancer	15	95+	15-39	Females Only
Chronic lymphoid leukaemia	20	95+	20-39	None
Chronic myeloid leukaemia	0	95+	15-39	None
Colon and rectum cancer	5	95+	15-39	None
Gallbladder and biliary tract cancer	20	95+	20-39	None
Hodgkin lymphoma	1	95+	15-39	None
Kidney cancer	0	95+	15-39	None
Larynx cancer	20	95+	20-39	None
Leukaemia	0	95+	15-39	None
Lip and oral cavity cancer	5	95+	15-39	None
Liver cancer	0	95+	15-39	None
Malignant skin melanoma	0	95+	15-39	None
Mesothelioma	20	95+	20-39	None
Multiple myeloma	20	95+	20-39	None
Nasopharynx cancer	5	95+	15-39	None
Non-Hodgkin lymphoma	1	95+	15-39	None
Oesophageal cancer	20	95+	20-39	None
Other leukaemia	0	95+	15-39	None
Other malignant neoplasms	0	95+	15-39	None
Other pharynx cancer	20	95+	20-39	None
Ovarian cancer	5	95+	15-39	Females Only
Pancreatic cancer	15	95+	15-39	None
Prostate cancer	20	95+	20-39	Males Only
Stomach cancer	15	95+	15-39	None
Testicular cancer	0	95+	15-39	Males Only
Thyroid cancer	5	95+	15-39	None
Tracheal, bronchus, and lung cancer	10	95+	15-39	None
Uterine cancer	20	95+	20-39	Females Only

*Many causes have minimum and maximum age restrictions that start and end outside of the 15-39 year age range that is the focus of this analysis. For the cancers that have minimum age restrictions starting above 15 years (Chronic lymphoid leukaemia, Gallbladder and biliary tract cancer, Larynx cancer, Mesothelioma, Multiple myeloma, Oesophageal cancer, Other pharynx cancer, Prostate cancer, and Uterine cancer), this means that no estimates are available in GBD 2019 or in this GBD 2019 AYA Cancers analysis in the 15-19 year age group.

CODEm models
Mortality estimates for each cancer were generated using the GBD Cause of Death Ensemble model (CODEm, #12 in Appendix figure 1) approach, the methods of which have been described in previous publications.[3,24] Additional details are specified in Section 3.1 of the Supplementary Appendix 1 to the GBD 2019 paper "Global burden of 369 diseases and injuries in 204 countries and territories, 1990–2019: a systematic analysis for the Global Burden of Disease Study 2019".[3] In brief, the CODEm approach is based on several principles: that all types of available data should be used, even if data quality varies; that a diverse set of plausible models with different combinations of covariates should be evaluated; that both individual models and the overall ensemble models should be tested for their predictive validity; and that the best model or sets of models should be chosen based on the out–of-sample predictive validity.

Covariates are provided for potential use in the ensemble based on a possible predictive relationship between the covariate and the specific cancer mortality, with an expected level and direction of association. Generally, Level 1 covariates have a proven strong relationship with the outcome, such as aetiological or biological roles. Level 2 covariates have a strong relationship but not a known direct biological link. Level 3 covariates have a relationship that may be more distal in the causal chain, or are mediated through Level 1 or 2 covariates.[24] The covariates provided to CODEm, as well as their level and direction, differ by cause and sex.

To generate an ensemble model, CODEm generates submodels that evaluate all plausible relationships between covariates and the response variable. Three additive components of data variance are used in CODEm: sampling variance, non-sampling variance, and garbage code redistribution variance. Model performance of all models is evaluated through out-of-sample predictive validity tests. Ensemble models are constructed from the individual models, with the contribution of individual models to the ensemble weighted by the basis of their predictive validity ranking. The final ensemble contains 1000 draws from these individual component models, from which a mean estimate and a 95% uncertainty interval are calculated. The 95% uncertainty interval represents the 0.025 and 0.975 quantiles of the draws.

Appendix Table 4: GBD 2019 covariates and level of covariates used in cause of death modelling for cancer types estimated

Cause	Sex	Covariate	Level	Direction
Acute lymphoid leukaemia	Male	Tobacco (cigarettes per capita)	2	1
Acute lymphoid leukaemia	Male	Cumulative Cigarettes (10 Years)	2	1
Acute lymphoid leukaemia	Male	Cumulative Cigarettes (20 Years)	2	1
Acute lymphoid leukaemia	Male	Education (years per capita)	3	-1
Acute lymphoid leukaemia	Male	LDI (I$ per capita)	3	-1
Acute lymphoid leukaemia	Male	Mean BMI	2	1
Acute lymphoid leukaemia	Male	Log-transformed SEV scalar: Leukaemia	1	1
Acute lymphoid leukaemia	Male	Log-transformed age-standardised SEV scalar: Leukaemia	1	1
Acute lymphoid leukaemia	Male	Socio-demographic Index	3	1
Acute lymphoid leukaemia	Male	Healthcare Access and Quality Index	2	-1
Acute lymphoid leukaemia	Male	Liters of alcohol consumed per capita	2	1
Acute lymphoid leukaemia	Female	Tobacco (cigarettes per capita)	2	1
Acute lymphoid leukaemia	Female	Cumulative Cigarettes (10 Years)	2	1

Cause	Sex	Covariate	Level	Direction
Acute lymphoid leukaemia	Female	Cumulative Cigarettes (20 Years)	2	1
Acute lymphoid leukaemia	Female	Education (years per capita)	3	-1
Acute lymphoid leukaemia	Female	LDI (I$ per capita)	3	-1
Acute lymphoid leukaemia	Female	Mean BMI	2	1
Acute lymphoid leukaemia	Female	Log-transformed SEV scalar: Leukaemia	1	1
Acute lymphoid leukaemia	Female	Log-transformed age-standardised SEV scalar: Leukaemia	1	1
Acute lymphoid leukaemia	Female	Socio-demographic Index	3	1
Acute lymphoid leukaemia	Female	Healthcare Access and Quality Index	2	-1
Acute lymphoid leukaemia	Female	Liters of alcohol consumed per capita	2	1
Acute myeloid leukaemia	Male	Cumulative Cigarettes (10 Years)	2	1
Acute myeloid leukaemia	Male	Cumulative Cigarettes (20 Years)	2	1
Acute myeloid leukaemia	Male	Education (years per capita)	3	-1
Acute myeloid leukaemia	Male	LDI (I$ per capita)	3	1
Acute myeloid leukaemia	Male	Mean BMI	2	1
Acute myeloid leukaemia	Male	Smoking Prevalence	2	1
Acute myeloid leukaemia	Male	Log-transformed SEV scalar: Leukaemia	1	1
Acute myeloid leukaemia	Male	Log-transformed age-standardised SEV scalar: Leukaemia	1	1
Acute myeloid leukaemia	Male	Socio-demographic Index	3	1
Acute myeloid leukaemia	Male	Healthcare Access and Quality Index	2	-1
Acute myeloid leukaemia	Male	Liters of alcohol consumed per capita	2	1
Acute myeloid leukaemia	Female	Cumulative Cigarettes (10 Years)	2	1
Acute myeloid leukaemia	Female	Cumulative Cigarettes (20 Years)	2	1
Acute myeloid leukaemia	Female	Education (years per capita)	3	-1
Acute myeloid leukaemia	Female	LDI (I$ per capita)	3	1
Acute myeloid leukaemia	Female	Mean BMI	2	1
Acute myeloid leukaemia	Female	Smoking Prevalence	2	1
Acute myeloid leukaemia	Female	Log-transformed SEV scalar: Leukaemia	1	1
Acute myeloid leukaemia	Female	Log-transformed age-standardised SEV scalar: Leukaemia	1	1
Acute myeloid leukaemia	Female	Socio-demographic Index	3	1
Acute myeloid leukaemia	Female	Healthcare Access and Quality Index	2	-1
Acute myeloid leukaemia	Female	Liters of alcohol consumed per capita	2	1
Bladder cancer	Male	Cumulative Cigarettes (10 Years)	2	1
Bladder cancer	Male	Diabetes Fasting Plasma Glucose (mmol/L), age-standardised 25+	2	1
Bladder cancer	Male	LDI (I$ per capita)	3	1
Bladder cancer	Male	Smoking Prevalence	1	1
Bladder cancer	Male	Schistosomiasis Prevalence Results	1	1
Bladder cancer	Male	Log-transformed SEV scalar: Bladder C	1	1

Cause	Sex	Covariate	Level	Direction
Bladder cancer	Male	Socio-demographic Index	3	1
Bladder cancer	Male	Healthcare Access and Quality Index	2	-1
Bladder cancer	Male	Age- and sex-specific SEV for Low fruit	3	1
Bladder cancer	Male	Age- and sex-specific SEV for Low vegetables	2	1
Bladder cancer	Male	Liters of alcohol consumed per capita	2	1
Bladder cancer	Female	Cumulative Cigarettes (10 Years)	2	1
Bladder cancer	Female	Diabetes Fasting Plasma Glucose (mmol/L), age-standardised 25+	2	1
Bladder cancer	Female	LDI (I$ per capita)	3	1
Bladder cancer	Female	Smoking Prevalence	1	1
Bladder cancer	Female	Schistosomiasis Prevalence Results	1	1
Bladder cancer	Female	Log-transformed SEV scalar: Bladder C	1	1
Bladder cancer	Female	Socio-demographic Index	3	1
Bladder cancer	Female	Healthcare Access and Quality Index	2	-1
Bladder cancer	Female	Age- and sex-specific SEV for Low fruit	3	1
Bladder cancer	Female	Age- and sex-specific SEV for Low vegetables	2	1
Bladder cancer	Female	Liters of alcohol consumed per capita	2	1
Brain and central nervous system cancer	Male	Cumulative Cigarettes (10 Years)	1	1
Brain and central nervous system cancer	Male	Education (years per capita)	3	-1
Brain and central nervous system cancer	Male	LDI (I$ per capita)	3	-1
Brain and central nervous system cancer	Male	Cholesterol (total, mean per capita)	2	1
Brain and central nervous system cancer	Male	Systolic Blood Pressure (mmHg)	2	1
Brain and central nervous system cancer	Male	Smoking Prevalence	1	1
Brain and central nervous system cancer	Male	Socio-demographic Index	3	1
Brain and central nervous system cancer	Male	Healthcare Access and Quality Index	2	-1
Brain and central nervous system cancer	Male	Age- and sex- specific SEV for low fruit	2	1
Brain and central nervous system cancer	Male	Age- and sex- specific SEV for Low vegetables	2	1
Brain and central nervous system cancer	Male	Age- and sex-specific SEV for High red meat	2	1
Brain and central nervous system cancer	Male	Liters of alcohol consumed per capita	1	1
Brain and central nervous system cancer	Female	Cumulative Cigarettes (10 Years)	1	1
Brain and central nervous system cancer	Female	Education (years per capita)	3	-1
Brain and central nervous system cancer	Female	LDI (I$ per capita)	3	-1

Cause	Sex	Covariate	Level	Direction
Brain and central nervous system cancer	Female	Cholesterol (total, mean per capita)	2	1
Brain and central nervous system cancer	Female	Systolic Blood Pressure (mmHg)	2	1
Brain and central nervous system cancer	Female	Smoking Prevalence	1	1
Brain and central nervous system cancer	Female	Socio-demographic Index	3	1
Brain and central nervous system cancer	Female	Healthcare Access and Quality Index	2	-1
Brain and central nervous system cancer	Female	Age- and sex- specific SEV for low fruit	2	1
Brain and central nervous system cancer	Female	Age- and sex- specific SEV for Low vegetables	2	1
Brain and central nervous system cancer	Female	Age- and sex-specific SEV for High red meat	2	1
Brain and central nervous system cancer	Female	Liters of alcohol consumed per capita	1	1
Breast cancer	Male	Cumulative cigarettes (10 years)	2	1
Breast cancer	Male	Cumulative cigarettes (20 years)	2	1
Breast cancer	Male	Diabetes Fasting Plasma Glucose (mmol/L), age-standardised 25+	2	1
Breast cancer	Male	Mean BMI	1	1
Breast cancer	Male	Total Fertility Rate	1	1
Breast cancer	Male	Socio-demographic Index	2	1
Breast cancer	Male	Age- and sex- specific SEV for low fruit	1	1
Breast cancer	Male	Liters of alcohol consumed per capita	1	1
Breast cancer	Male	Healthcare Access and Quality Index	2	-1
Breast cancer	Male	Age- and sex- specific SEV for low fruit	2	1
Breast cancer	Male	Age- and sex- specific SEV for Low vegetables	2	1
Breast cancer	Male	Liters of alcohol consumed per capita	1	1
Breast cancer	Female	Age-specific fertility rate	2	-1
Breast cancer	Female	Cumulative cigarettes (10 years)	2	1
Breast cancer	Female	Cumulative cigarettes (20 years)	2	1
Breast cancer	Female	Diabetes Fasting Plasma Glucose (mmol/L), age-standardised 25+	2	1
Breast cancer	Female	LDI (I$ per capita)	3	-1
Breast cancer	Female	Mean BMI	1	1
Breast cancer	Female	Smoking Prevalence	2	1
Breast cancer	Female	Total Fertility Rate	2	-1
Breast cancer	Female	Log-transformed SEV scalar: Breast C	1	1
Breast cancer	Female	Socio-demographic Index	3	1
Breast cancer	Female	Healthcare Access and Quality Index	2	-1
Breast cancer	Female	Age- and sex- specific SEV for low fruit	2	1

Cause	Sex	Covariate	Level	Direction
Breast cancer	Female	Age- and sex- specific SEV for Low vegetables	2	1
Breast cancer	Female	Liters of alcohol consumed per capita	1	1
Cervical cancer	Female	Age-specific fertility rate	2	1
Cervical cancer	Female	Cumulative Cigarettes (5 Years)	1	1
Cervical cancer	Female	Education (years per capita)	3	-1
Cervical cancer	Female	LDI (I$ per capita)	3	-1
Cervical cancer	Female	Smoking Prevalence	2	1
Cervical cancer	Female	Total Fertility Rate	2	1
Cervical cancer	Female	Socio-demographic Index	3	-1
Cervical cancer	Female	HIV age-standardised prevalence	1	1
Cervical cancer	Female	Healthcare Access and Quality Index	2	-1
Cervical cancer	Female	Age- and sex- specific SEV for low fruit	2	1
Cervical cancer	Female	Age- and sex- specific SEV for Low vegetables	2	1
Chronic lymphoid leukaemia	Male	Tobacco (cigarettes per capita)	2	1
Chronic lymphoid leukaemia	Male	Cumulative cigarettes (10 years)	2	1
Chronic lymphoid leukaemia	Male	Cumulative cigarettes (15 years)	2	1
Chronic lymphoid leukaemia	Male	Cumulative cigarettes (20 years)	2	1
Chronic lymphoid leukaemia	Male	Cumulative cigarettes (5 years)	2	1
Chronic lymphoid leukaemia	Male	Education (years per capita)	3	-1
Chronic lymphoid leukaemia	Male	LDI (I$ per capita)	3	-1
Chronic lymphoid leukaemia	Male	Mean BMI	2	1
Chronic lymphoid leukaemia	Male	Smoking Prevalence	2	1
Chronic lymphoid leukaemia	Male	Log-transformed SEV scalar: Leukaemia	1	1
Chronic lymphoid leukaemia	Male	Log-transformed age-standardised SEV scalar: Leukaemia	1	1
Chronic lymphoid leukaemia	Male	Socio-demographic Index	3	1
Chronic lymphoid leukaemia	Male	Healthcare Access and Quality Index	2	-1
Chronic lymphoid leukaemia	Male	Liters of alcohol consumed per capita	2	1
Chronic lymphoid leukaemia	Female	Tobacco (cigarettes per capita)	2	1
Chronic lymphoid leukaemia	Female	Cumulative cigarettes (10 years)	2	1
Chronic lymphoid leukaemia	Female	Cumulative cigarettes (15 years)	2	1
Chronic lymphoid leukaemia	Female	Cumulative cigarettes (20 years)	2	1
Chronic lymphoid leukaemia	Female	Cumulative cigarettes (5 years)	2	1
Chronic lymphoid leukaemia	Female	Education (years per capita)	3	-1
Chronic lymphoid leukaemia	Female	LDI (I$ per capita)	3	-1
Chronic lymphoid leukaemia	Female	Mean BMI	2	1
Chronic lymphoid leukaemia	Female	Smoking Prevalence	2	1
Chronic lymphoid leukaemia	Female	Log-transformed SEV scalar: Leukaemia	1	1
Chronic lymphoid leukaemia	Female	Log-transformed age-standardised SEV scalar: Leukaemia	1	1

Cause	Sex	Covariate	Level	Direction
Chronic lymphoid leukaemia	Female	Socio-demographic Index	3	1
Chronic lymphoid leukaemia (dr)	Female	Healthcare Access and Quality Index	2	-1
Chronic lymphoid leukaemia (glb)	Female	Healthcare Access and Quality Index	1	1
Chronic lymphoid leukaemia	Female	Liters of alcohol consumed per capita	2	1
Chronic myeloid leukaemia	Male	Tobacco (cigarettes per capita)	2	1
Chronic myeloid leukaemia	Male	Cumulative cigarettes (10 years)	2	1
Chronic myeloid leukaemia	Male	Cumulative cigarettes (15 years)	2	1
Chronic myeloid leukaemia	Male	Cumulative cigarettes (20 years)	2	1
Chronic myeloid leukaemia	Male	Cumulative cigarettes (5 years)	2	1
Chronic myeloid leukaemia	Male	Education (years per capita)	3	-1
Chronic myeloid leukaemia	Male	LDI (I$ per capita)	3	1
Chronic myeloid leukaemia	Male	Mean BMI	2	1
Chronic myeloid leukaemia	Male	Smoking Prevalence	2	1
Chronic myeloid leukaemia	Male	Log-transformed SEV scalar: Leukaemia	1	1
Chronic myeloid leukaemia	Male	Log-transformed age-standardised SEV scalar: Leukaemia	1	1
Chronic myeloid leukaemia	Male	Socio-demographic Index	3	-1
Chronic myeloid leukaemia	Male	Healthcare Access and Quality Index	2	-1
Chronic myeloid leukaemia	Male	Liters of alcohol consumed per capita	2	1
Chronic myeloid leukaemia	Female	Tobacco (cigarettes per capita)	2	1
Chronic myeloid leukaemia	Female	Cumulative cigarettes (10 years)	2	1
Chronic myeloid leukaemia	Female	Cumulative cigarettes (15 years)	2	1
Chronic myeloid leukaemia	Female	Cumulative cigarettes (20 years)	2	1
Chronic myeloid leukaemia	Female	Cumulative cigarettes (5 years)	2	1
Chronic myeloid leukaemia	Female	Education (years per capita)	3	-1
Chronic myeloid leukaemia	Female	LDI (I$ per capita)	3	1
Chronic myeloid leukaemia	Female	Mean BMI	2	1
Chronic myeloid leukaemia	Female	Smoking Prevalence	2	1
Chronic myeloid leukaemia	Female	Log-transformed SEV scalar: Leukaemia	1	1
Chronic myeloid leukaemia	Female	Log-transformed age-standardised SEV scalar: Leukaemia	1	1
Chronic myeloid leukaemia	Female	Socio-demographic Index	3	-1
Chronic myeloid leukaemia	Female	Healthcare Access and Quality Index	2	-1
Chronic myeloid leukaemia	Female	Liters of alcohol consumed per capita	2	1
Colon and rectum cancer	Male	Tobacco (cigarettes per capita)	1	1
Colon and rectum cancer	Male	Cumulative cigarettes (20 years)	2	1
Colon and rectum cancer	Male	Diabetes Fasting Plasma Glucose (mmol/L), age-standardised 25+	2	1
Colon and rectum cancer	Male	Education (years per capita)	3	-1
Colon and rectum cancer	Male	LDI (I$ per capita)	3	1

Cause	Sex	Covariate	Level	Direction
Colon and rectum cancer	Male	Mean BMI	1	1
Colon and rectum cancer	Male	Log-transformed SEV scalar: Colorect C	1	1
Colon and rectum cancer	Male	Socio-demographic Index	3	1
Colon and rectum cancer	Male	pufa adjusted(percent)	2	-1
Colon and rectum cancer	Male	Healthcare Access and Quality Index	3	-1
Colon and rectum cancer	Male	Total Physical Activity (MET-min/week), Age-specific	1	-1
Colon and rectum cancer	Male	Age- and sex- specific SEV for low fruit	3	1
Colon and rectum cancer	Male	Age- and sex- specific SEV for Low vegetables	2	1
Colon and rectum cancer	Male	Age- and sex-specific SEV for Low nuts and seeds	3	1
Colon and rectum cancer	Male	Age- and sex-specific SEV for Low milk	3	1
Colon and rectum cancer	Male	Age- and sex-specific SEV for High red meat	1	1
Colon and rectum cancer	Male	Age- and sex-specific SEV for Low fibre	2	1
Colon and rectum cancer	Male	Age- and sex-specific SEV for Low calcium	2	1
Colon and rectum cancer	Male	Liters of alcohol consumed per capita	2	1
Colon and rectum cancer	Female	Tobacco (cigarettes per capita)	1	1
Colon and rectum cancer	Female	Cumulative cigarettes (5 years)	2	1
Colon and rectum cancer	Female	Diabetes Fasting Plasma Glucose (mmol/L), age-standardised 25+	2	1
Colon and rectum cancer	Female	Education (years per capita)	3	-1
Colon and rectum cancer	Female	LDI (I$ per capita)	3	1
Colon and rectum cancer	Female	Mean BMI	1	1
Colon and rectum cancer	Female	Log-transformed SEV scalar: Colorect C	1	1
Colon and rectum cancer	Female	Socio-demographic Index	3	1
Colon and rectum cancer	Female	pufa adjusted(percent)	2	-1
Colon and rectum cancer	Female	Healthcare Access and Quality Index	3	-1
Colon and rectum cancer	Female	Total Physical Activity (MET-min/week), Age-specific	1	-1
Colon and rectum cancer	Female	Age- and sex- specific SEV for low fruit	3	1
Colon and rectum cancer	Female	Age- and sex- specific SEV for Low vegetables	2	1
Colon and rectum cancer	Female	Age- and sex-specific SEV for Low nuts and seeds	3	1
Colon and rectum cancer	Female	Age- and sex-specific SEV for Low milk	3	1
Colon and rectum cancer	Female	Age- and sex-specific SEV for High red meat	1	1
Colon and rectum cancer	Female	Age- and sex-specific SEV for Low fibre	2	1
Colon and rectum cancer	Female	Age- and sex-specific SEV for Low calcium	2	1
Colon and rectum cancer	Female	Liters of alcohol consumed per capita	2	1
Gallbladder and biliary tract cancer	Male	Tobacco (cigarettes per capita)	2	1
Gallbladder and biliary tract cancer	Male	Cumulative cigarettes (10 years)	2	1
Gallbladder and biliary tract cancer	Male	Cumulative Cigarettes (5 Years)	2	1

Cause	Sex	Covariate	Level	Direction
Gallbladder and biliary tract cancer	Male	Diabetes Age-Standardised Prevalence (proportion)	2	1
Gallbladder and biliary tract cancer	Male	Education (years per capita)	3	-1
Gallbladder and biliary tract cancer	Male	LDI (I$ per capita)	3	1
Gallbladder and biliary tract cancer	Male	Mean BMI	1	1
Gallbladder and biliary tract cancer	Male	Smoking Prevalence	2	1
Gallbladder and biliary tract cancer	Male	Log-transformed SEV scalar: Gallblad C	1	1
Gallbladder and biliary tract cancer	Male	Socio-demographic Index	3	-1
Gallbladder and biliary tract cancer	Male	Healthcare Access and Quality Index	2	-1
Gallbladder and biliary tract cancer	Male	Age- and sex- specific SEV for low fruit	2	1
Gallbladder and biliary tract cancer	Male	Age- and sex- specific SEV for Low vegetables	2	1
Gallbladder and biliary tract cancer	Male	Liters of alcohol consumed per capita	2	1
Gallbladder and biliary tract cancer	Female	Tobacco (cigarettes per capita)	2	1
Gallbladder and biliary tract cancer	Female	Cumulative cigarettes (10 years)	2	1
Gallbladder and biliary tract cancer	Female	Cumulative Cigarettes (5 Years)	2	1
Gallbladder and biliary tract cancer	Female	Diabetes Age-Standardised Prevalence (proportion)	2	1
Gallbladder and biliary tract cancer	Female	Education (years per capita)	3	-1
Gallbladder and biliary tract cancer	Female	LDI (I$ per capita)	3	1
Gallbladder and biliary tract cancer	Female	Mean BMI	1	1
Gallbladder and biliary tract cancer	Female	Smoking Prevalence	2	1
Gallbladder and biliary tract cancer	Female	Log-transformed SEV scalar: Gallblad C	1	1
Gallbladder and biliary tract cancer	Female	Socio-demographic Index	3	-1
Gallbladder and biliary tract cancer	Female	Healthcare Access and Quality Index	2	-1
Gallbladder and biliary tract cancer	Female	Age- and sex- specific SEV for low fruit	2	1
Gallbladder and biliary tract cancer	Female	Age- and sex- specific SEV for Low vegetables	2	1
Gallbladder and biliary tract cancer	Female	Liters of alcohol consumed per capita	2	1
Hodgkin lymphoma	Male	Education (years per capita)	3	-1

Cause	Sex	Covariate	Level	Direction
Hodgkin lymphoma	Male	LDI (I$ per capita)	3	-1
Hodgkin lymphoma	Male	Socio-demographic Index	3	-1
Hodgkin lymphoma	Male	Healthcare Access and Quality Index	2	-1
Hodgkin lymphoma	Female	Education (years per capita)	3	-1
Hodgkin lymphoma	Female	LDI (I$ per capita)	3	-1
Hodgkin lymphoma	Female	Socio-demographic Index	3	-1
Hodgkin lymphoma	Female	Healthcare Access and Quality Index	2	-1
Kidney cancer	Male	Tobacco (cigarettes per capita)	1	1
Kidney cancer	Male	Cumulative cigarettes (10 years)	1	1
Kidney cancer	Male	Diabetes Age-Standardised Prevalence (proportion)	2	1
Kidney cancer	Male	Education (years per capita)	3	-1
Kidney cancer	Male	LDI (I$ per capita)	3	1
Kidney cancer	Male	Mean BMI	1	1
Kidney cancer	Male	Systolic Blood Pressure (mmHg)	2	1
Kidney cancer	Male	Log-transformed SEV scalar: Kidney C	1	1
Kidney cancer	Male	Socio-demographic Index	3	1
Kidney cancer	Male	Healthcare Access and Quality Index	2	-1
Kidney cancer	Male	Liters of alcohol consumed per capita	2	1
Kidney cancer	Female	Tobacco (cigarettes per capita)	1	1
Kidney cancer	Female	Cumulative cigarettes (10 years)	1	1
Kidney cancer	Female	Diabetes Age-Standardised Prevalence (proportion)	2	1
Kidney cancer	Female	Education (years per capita)	3	-1
Kidney cancer	Female	LDI (I$ per capita)	3	1
Kidney cancer	Female	Mean BMI	1	1
Kidney cancer	Female	Systolic Blood Pressure (mmHg)	2	1
Kidney cancer	Female	Log-transformed SEV scalar: Kidney C	1	1
Kidney cancer	Female	Socio-demographic Index	3	1
Kidney cancer	Female	Healthcare Access and Quality Index	2	-1
Kidney cancer	Female	Liters of alcohol consumed per capita	2	1
Larynx cancer	Male	Cumulative cigarettes (10 years)	2	1
Larynx cancer	Male	Cumulative cigarettes (20 years)	2	1
Larynx cancer	Male	LDI (I$ per capita)	3	1
Larynx cancer	Male	Population Density (over 1000 ppl/sqkm, proportion)	2	1
Larynx cancer	Male	Smoking Prevalence	2	1
Larynx cancer	Male	Log-transformed SEV scalar: Larynx C	1	1
Larynx cancer	Male	Socio-demographic Index	3	1
Larynx cancer	Male	Healthcare Access and Quality Index	2	-1
Larynx cancer	Male	Asbestos consumption (metric tons per year per capita)	2	1
Larynx cancer	Male	Age- and sex- specific SEV for low fruit	2	1

Cause	Sex	Covariate	Level	Direction
Larynx cancer	Male	Age- and sex- specific SEV for Low vegetables	3	1
Larynx cancer	Male	Liters of alcohol consumed per capita	1	1
Larynx cancer	Female	Cumulative cigarettes (10 years)	2	1
Larynx cancer	Female	Cumulative cigarettes (20 years)	2	1
Larynx cancer	Female	LDI (I$ per capita)	3	1
Larynx cancer	Female	Population Density (over 1000 ppl/sqkm, proportion)	2	1
Larynx cancer	Female	Smoking Prevalence	2	1
Larynx cancer	Female	Log-transformed SEV scalar: Larynx C	1	1
Larynx cancer	Female	Socio-demographic Index	3	1
Larynx cancer	Female	Healthcare Access and Quality Index	2	-1
Larynx cancer	Female	Asbestos consumption (metric tons per year per capita)	2	1
Larynx cancer	Female	Age- and sex- specific SEV for low fruit	3	1
Larynx cancer	Female	Age- and sex- specific SEV for Low vegetables	2	1
Larynx cancer	Female	Liters of alcohol consumed per capita	1	1
Leukaemia	Male	Tobacco (cigarettes per capita)	2	1
Leukaemia	Male	Cumulative cigarettes (10 years)	2	1
Leukaemia	Male	Cumulative cigarettes (20 years)	2	1
Leukaemia	Male	Education (years per capita)	3	-1
Leukaemia	Male	LDI (I$ per capita)	3	1
Leukaemia	Male	Mean BMI	2	1
Leukaemia	Male	Log-transformed SEV scalar: Leukaemia	1	1
Leukaemia	Male	Log-transformed age-standardised SEV scalar: Leukaemia	1	1
Leukaemia	Male	Socio-demographic Index	3	-1
Leukaemia	Male	Healthcare Access and Quality Index	2	-1
Leukaemia	Male	Liters of alcohol consumed per capita	2	1
Leukaemia	Female	Tobacco (cigarettes per capita)	2	1
Leukaemia	Female	Cumulative cigarettes (10 years)	2	1
Leukaemia	Female	Cumulative cigarettes (20 years)	2	1
Leukaemia	Female	Education (years per capita)	3	-1
Leukaemia	Female	LDI (I$ per capita)	3	1
Leukaemia	Female	Mean BMI	2	1
Leukaemia	Female	Log-transformed SEV scalar: Leukaemia	1	1
Leukaemia	Female	Log-transformed age-standardised SEV scalar: Leukaemia	1	1
Leukaemia	Female	Socio-demographic Index	3	-1
Leukaemia	Female	Healthcare Access and Quality Index	2	-1
Leukaemia	Female	Liters of alcohol consumed per capita	2	1
Lip and oral cavity cancer	Male	Tobacco (cigarettes per capita)	1	1
Lip and oral cavity cancer	Male	Cumulative cigarettes (10 years)	1	1

Cause	Sex	Covariate	Level	Direction
Lip and oral cavity cancer	Male	Cumulative cigarettes (20 years)	1	1
Lip and oral cavity cancer	Male	Education (years per capita)	3	-1
Lip and oral cavity cancer	Male	LDI (I$ per capita)	3	1
Lip and oral cavity cancer	Male	Log-transformed SEV scalar: Lip oral C	1	1
Lip and oral cavity cancer	Male	Socio-demographic Index	3	1
Lip and oral cavity cancer	Male	Healthcare Access and Quality Index	2	-1
Lip and oral cavity cancer	Male	Age- and sex- specific SEV for low fruit	2	1
Lip and oral cavity cancer	Male	Age- and sex- specific SEV for Low vegetables	2	1
Lip and oral cavity cancer	Male	Age- and sex- specific SEV for High red meat	2	1
Lip and oral cavity cancer	Male	Liters of alcohol consumed per capita	1	1
Lip and oral cavity cancer	Female	Tobacco (cigarettes per capita)	1	1
Lip and oral cavity cancer	Female	Cumulative cigarettes (10 years)	1	1
Lip and oral cavity cancer	Female	Cumulative cigarettes (20 years)	1	1
Lip and oral cavity cancer	Female	Education (years per capita)	3	-1
Lip and oral cavity cancer	Female	LDI (I$ per capita)	3	1
Lip and oral cavity cancer	Female	Log-transformed SEV scalar: Lip oral C	1	1
Lip and oral cavity cancer	Female	Socio-demographic Index	3	1
Lip and oral cavity cancer	Female	Healthcare Access and Quality Index	2	-1
Lip and oral cavity cancer	Female	Age- and sex- specific SEV for low fruit	2	1
Lip and oral cavity cancer	Female	Age- and sex- specific SEV for Low vegetables	2	1
Lip and oral cavity cancer	Female	Age- and sex- specific SEV for High red meat	2	1
Lip and oral cavity cancer	Female	Liters of alcohol consumed per capita	1	1
Liver cancer	Male	Tobacco (cigarettes per capita)	2	1
Liver cancer	Male	Cumulative cigarettes (20 years)	2	1
Liver cancer	Male	Diabetes Fasting Plasma Glucose (mmol/L), age-standardised 25+	2	1
Liver cancer	Male	Education (years per capita)	3	-1
Liver cancer	Male	LDI (I$ per capita)	3	-1
Liver cancer	Male	Mean BMI	2	1
Liver cancer	Male	Log-transformed SEV scalar: Liver C	1	1
Liver cancer	Male	Socio-demographic Index	3	-1
Liver cancer	Male	HIV age-standardised prevalence	1	1
Liver cancer	Male	Healthcare Access and Quality Index	2	-1
Liver cancer	Male	Hepatitis B 3-dose coverage (proportion)	2	-1
Liver cancer	Male	Intravenous drug use (age-standardised proportion)	2	1
Liver cancer	Male	Hepatitis B vaccine coverage (proportion), aged through time	2	-1
Liver cancer	Male	Age- and sex-specific SEV for High red meat	3	1
Liver cancer	Male	Hepatitis B Seroprevalence (HBsAg) age standardised	1	1
Liver cancer	Male	Hepatitis C Seroprevalence (anti-HCV) age standardised	1	1

Cause	Sex	Covariate	Level	Direction
Liver cancer	Male	Liters of alcohol consumed per capita	1	1
Liver cancer	Female	Tobacco (cigarettes per capita)	2	1
Liver cancer	Female	Cumulative cigarettes (20 years)	2	1
Liver cancer	Female	Diabetes Fasting Plasma Glucose (mmol/L), age-standardised 25+	2	1
Liver cancer	Female	Education (years per capita)	3	-1
Liver cancer	Female	LDI (I$ per capita)	3	-1
Liver cancer	Female	Mean BMI	2	1
Liver cancer	Female	Log-transformed SEV scalar: Liver C	1	1
Liver cancer	Female	Socio-demographic Index	3	-1
Liver cancer	Female	HIV age-standardised prevalence	1	1
Liver cancer	Female	Healthcare Access and Quality Index	2	-1
Liver cancer	Female	Hepatitis B 3-dose coverage (proportion)	2	-1
Liver cancer	Female	Intravenous drug use (age-standardised proportion)	2	1
Liver cancer	Female	Hepatitis B vaccine coverage (proportion), aged through time	2	-1
Liver cancer	Female	Age- and sex-specific SEV for High red meat	3	1
Liver cancer	Female	Hepatitis B Seroprevalence (HBsAg) age standardised	1	1
Liver cancer	Female	Hepatitis C Seroprevalence (anti-HCV) age standardised	1	1
Liver cancer	Female	Liters of alcohol consumed per capita	1	1
Malignant skin melanoma	Male	Education (years per capita)	3	-1
Malignant skin melanoma	Male	LDI (I$ per capita)	3	-1
Malignant skin melanoma	Male	Latitude Under 15 (proportion)	2	-1
Malignant skin melanoma	Male	Latitude 15 to 30 (proportion)	2	-1
Malignant skin melanoma	Male	Latitude 30 to 45 (proportion)	2	-1
Malignant skin melanoma	Male	Latitude Over 45 (proportion)	2	-1
Malignant skin melanoma	Male	Socio-demographic Index	3	1
Malignant skin melanoma	Male	Healthcare Access and Quality Index	2	-1
Malignant skin melanoma	Male	Liters of alcohol consumed per capita	1	1
Malignant skin melanoma	Female	Education (years per capita)	3	-1
Malignant skin melanoma	Female	LDI (I$ per capita)	3	-1
Malignant skin melanoma	Female	Latitude Under 15 (proportion)	2	-1
Malignant skin melanoma	Female	Latitude 15 to 30 (proportion)	2	-1
Malignant skin melanoma	Female	Latitude 30 to 45 (proportion)	2	-1
Malignant skin melanoma	Female	Latitude Over 45 (proportion)	2	-1
Malignant skin melanoma	Female	Socio-demographic Index	3	1
Malignant skin melanoma	Female	Healthcare Access and Quality Index	2	-1
Malignant skin melanoma	Female	Liters of alcohol consumed per capita	1	1
Mesothelioma	Male	Cumulative Cigarettes (5 Years)	2	1
Mesothelioma	Male	Education (years per capita)	3	-1

Cause	Sex	Covariate	Level	Direction
Mesothelioma	Male	Gold production (binary)	2	1
Mesothelioma	Male	LDI (I$ per capita)	3	-1
Mesothelioma	Male	Indoor Air Pollution (All Cooking Fuels)	2	1
Mesothelioma	Male	Population Density (over 1000 ppl/sqkm, proportion)	2	1
Mesothelioma	Male	Smoking Prevalence	1	1
Mesothelioma	Male	Log-transformed SEV scalar: Mesothel	1	1
Mesothelioma	Male	Log-transformed age-standardised SEV scalar: Mesothel	1	1
Mesothelioma	Male	Socio-demographic Index	3	1
Mesothelioma	Male	Healthcare Access and Quality Index	2	-1
Mesothelioma	Male	Asbestos consumption (metric tons per year per capita)	1	1
Mesothelioma	Female	Cumulative Cigarettes (5 Years)	2	1
Mesothelioma	Female	Education (years per capita)	3	-1
Mesothelioma	Female	Gold production (binary)	2	1
Mesothelioma	Female	LDI (I$ per capita)	3	-1
Mesothelioma	Female	Indoor Air Pollution (All Cooking Fuels)	2	1
Mesothelioma	Female	Population Density (over 1000 ppl/sqkm, proportion)	2	1
Mesothelioma	Female	Smoking Prevalence	1	1
Mesothelioma	Female	Socio-demographic Index	3	1
Mesothelioma	Female	Healthcare Access and Quality Index	2	-1
Mesothelioma	Female	Asbestos consumption (metric tons per year per capita)	1	1
Multiple myeloma	Male	Tobacco (cigarettes per capita)	1	1
Multiple myeloma	Male	Education (years per capita)	3	-1
Multiple myeloma	Male	LDI (I$ per capita)	3	1
Multiple myeloma	Male	Mean BMI	2	1
Multiple myeloma	Male	Sanitation (proportion with access)	2	-1
Multiple myeloma	Male	Smoking Prevalence	1	1
Multiple myeloma	Male	Improved Water Source (proportion with access)	2	-1
Multiple myeloma	Male	Socio-demographic Index	3	1
Multiple myeloma	Male	Healthcare Access and Quality Index	2	-1
Multiple myeloma	Male	Age- and sex- specific SEV for low fruit	2	1
Multiple myeloma	Male	Age- and sex- specific SEV for Low vegetables	2	1
Multiple myeloma	Male	Age- and sex-specific SEV for High red meat	2	1
Multiple myeloma	Male	Liters of alcohol consumed per capita	1	1
Multiple myeloma	Female	Tobacco (cigarettes per capita)	1	1
Multiple myeloma	Female	Education (years per capita)	3	-1
Multiple myeloma	Female	LDI (I$ per capita)	3	1
Multiple myeloma	Female	Mean BMI	2	1
Multiple myeloma	Female	Sanitation (proportion with access)	2	-1
Multiple myeloma	Female	Smoking Prevalence	1	1

Cause	Sex	Covariate	Level	Direction
Multiple myeloma	Female	Improved Water Source (proportion with access)	2	-1
Multiple myeloma	Female	Socio-demographic Index	3	1
Multiple myeloma	Female	Healthcare Access and Quality Index	2	-1
Multiple myeloma	Female	Age- and sex- specific SEV for low fruit	2	1
Multiple myeloma	Female	Age- and sex- specific SEV for Low vegetables	2	1
Multiple myeloma	Female	Age- and sex-specific SEV for High red meat	2	1
Multiple myeloma	Female	Liters of alcohol consumed per capita	1	1
Nasopharynx cancer	Male	Tobacco (cigarettes per capita)	1	1
Nasopharynx cancer	Male	Cumulative cigarettes (10 years)	1	1
Nasopharynx cancer	Male	Cumulative cigarettes (20 years)	1	1
Nasopharynx cancer	Male	Education (years per capita)	3	-1
Nasopharynx cancer	Male	LDI (I$ per capita)	3	-1
Nasopharynx cancer	Male	Population Density (over 1000 ppl/sqkm, proportion)	2	1
Nasopharynx cancer	Male	Log-transformed SEV scalar: Nasoph C	1	1
Nasopharynx cancer	Male	Socio-demographic Index	3	1
Nasopharynx cancer	Male	Healthcare Access and Quality Index	2	-1
Nasopharynx cancer	Male	Age- and sex- specific SEV for low fruit	3	1
Nasopharynx cancer	Male	Age- and sex- specific SEV for Low vegetables	2	1
Nasopharynx cancer	Male	Liters of alcohol consumed per capita	1	1
Nasopharynx cancer	Female	Tobacco (cigarettes per capita)	1	1
Nasopharynx cancer	Female	Cumulative cigarettes (10 years)	1	1
Nasopharynx cancer	Female	Cumulative cigarettes (20 years)	1	1
Nasopharynx cancer	Female	Education (years per capita)	3	-1
Nasopharynx cancer	Female	LDI (I$ per capita)	3	-1
Nasopharynx cancer	Female	Population Density (over 1000 ppl/sqkm, proportion)	2	1
Nasopharynx cancer	Female	Log-transformed SEV scalar: Nasoph C	1	1
Nasopharynx cancer	Female	Socio-demographic Index	3	1
Nasopharynx cancer	Female	Healthcare Access and Quality Index	2	-1
Nasopharynx cancer	Female	Age- and sex- specific SEV for low fruit	3	1
Nasopharynx cancer	Female	Age- and sex- specific SEV for Low vegetables	2	1
Nasopharynx cancer	Female	Liters of alcohol consumed per capita	1	1
Non-Hodgkin lymphoma	Male	Cumulative cigarettes (10 years)	2	1
Non-Hodgkin lymphoma	Male	Cumulative cigarettes (15 years)	2	1
Non-Hodgkin lymphoma	Male	Cumulative cigarettes (20 years)	2	1
Non-Hodgkin lymphoma	Male	Cumulative Cigarettes (5 Years)	2	1
Non-Hodgkin lymphoma	Male	LDI (I$ per capita)	3	1
Non-Hodgkin lymphoma	Male	Mean BMI	2	1
Non-Hodgkin lymphoma	Male	Smoking Prevalence	2	1
Non-Hodgkin lymphoma	Male	Socio-demographic Index	3	1

Cause	Sex	Covariate	Level	Direction
Non-Hodgkin lymphoma	Male	Healthcare Access and Quality Index	2	-1
Non-Hodgkin lymphoma	Male	Liters of alcohol consumed per capita	2	1
Non-Hodgkin lymphoma	Female	Cumulative cigarettes (10 years)	2	1
Non-Hodgkin lymphoma	Female	Cumulative cigarettes (15 years)	2	1
Non-Hodgkin lymphoma	Female	Cumulative cigarettes (20 years)	2	1
Non-Hodgkin lymphoma	Female	Cumulative Cigarettes (5 Years)	2	1
Non-Hodgkin lymphoma	Female	LDI (I$ per capita)	3	1
Non-Hodgkin lymphoma	Female	Mean BMI	2	1
Non-Hodgkin lymphoma	Female	Smoking Prevalence	2	1
Non-Hodgkin lymphoma	Female	Total Fertility Rate	3	-1
Non-Hodgkin lymphoma	Female	Socio-demographic Index	3	1
Non-Hodgkin lymphoma	Female	Healthcare Access and Quality Index	2	-1
Non-Hodgkin lymphoma	Female	Liters of alcohol consumed per capita	2	1
Oesophageal cancer	Male	Tobacco (cigarettes per capita)	2	1
Oesophageal cancer	Male	Education (years per capita)	3	-1
Oesophageal cancer	Male	LDI (I$ per capita)	3	1
Oesophageal cancer	Male	Mean BMI	1	1
Oesophageal cancer	Male	Indoor Air Pollution (All Cooking Fuels)	2	1
Oesophageal cancer	Male	Sanitation (proportion with access)	3	-1
Oesophageal cancer	Male	Smoking Prevalence	1	1
Oesophageal cancer	Male	Improved Water Source (proportion with access)	3	-1
Oesophageal cancer	Male	Log-transformed age-standardised SEV scalar: Esophag C	1	1
Oesophageal cancer	Male	Socio-demographic Index	3	1
Oesophageal cancer	Male	Healthcare Access and Quality Index	2	-1
Oesophageal cancer	Male	Age- and sex- specific SEV for low fruit	2	1
Oesophageal cancer	Male	Age- and sex- specific SEV for Low vegetables	2	1
Oesophageal cancer	Male	Liters of alcohol consumed per capita	1	1
Oesophageal cancer	Female	Tobacco (cigarettes per capita)	2	1
Oesophageal cancer	Female	Education (years per capita)	3	-1
Oesophageal cancer	Female	LDI (I$ per capita)	3	1
Oesophageal cancer	Female	Mean BMI	1	1
Oesophageal cancer	Female	Indoor Air Pollution (All Cooking Fuels)	2	1
Oesophageal cancer	Female	Sanitation (proportion with access)	3	-1
Oesophageal cancer	Female	Smoking Prevalence	1	1
Oesophageal cancer	Female	Improved Water Source (proportion with access)	3	-1
Oesophageal cancer	Female	Log-transformed age-standardised SEV scalar: Esophag C	1	1
Oesophageal cancer	Female	Socio-demographic Index	3	1
Oesophageal cancer	Female	Healthcare Access and Quality Index	2	-1

Cause	Sex	Covariate	Level	Direction
Oesophageal cancer	Female	Age- and sex- specific SEV for low fruit	2	1
Oesophageal cancer	Female	Age- and sex- specific SEV for Low vegetables	2	1
Oesophageal cancer	Female	Liters of alcohol consumed per capita	1	1
Other leukaemia	Male	Tobacco (cigarettes per capita)	2	1
Other leukaemia	Male	Cumulative cigarettes (10 years)	2	1
Other leukaemia	Male	Cumulative cigarettes (20 years)	2	1
Other leukaemia	Male	Education (years per capita)	3	-1
Other leukaemia	Male	LDI (I$ per capita)	3	1
Other leukaemia	Male	Mean BMI	2	1
Other leukaemia	Male	Log-transformed SEV scalar: Leukaemia	1	1
Other leukaemia	Male	Log-transformed age-standardised SEV scalar: Leukaemia	1	1
Other leukaemia	Male	Socio-demographic Index	3	-1
Other leukaemia	Male	Healthcare Access and Quality Index	2	-1
Other leukaemia	Male	Liters of alcohol consumed per capita	2	1
Other leukaemia	Female	Tobacco (cigarettes per capita)	2	1
Other leukaemia	Female	Cumulative cigarettes (10 years)	2	1
Other leukaemia	Female	Cumulative cigarettes (20 years)	2	1
Other leukaemia	Female	Education (years per capita)	3	-1
Other leukaemia	Female	LDI (I$ per capita)	3	1
Other leukaemia	Female	Mean BMI	2	1
Other leukaemia	Female	Log-transformed SEV scalar: Leukaemia	1	1
Other leukaemia	Female	Log-transformed age-standardised SEV scalar: Leukaemia	1	1
Other leukaemia	Female	Socio-demographic Index	3	-1
Other leukaemia	Female	Healthcare Access and Quality Index	2	-1
Other leukaemia (dr)	Female	Liters of alcohol consumed per capita	1	1
Other leukaemia (glb)	Female	Liters of alcohol consumed per capita	2	1
Other malignant neoplasms	Male	Tobacco (cigarettes per capita)	1	1
Other malignant neoplasms	Male	Education (years per capita)	3	-1
Other malignant neoplasms	Male	LDI (I$ per capita)	3	1
Other malignant neoplasms	Male	Smoking Prevalence	1	1
Other malignant neoplasms	Male	Socio-demographic Index	3	1
Other malignant neoplasms	Male	pufa adjusted(percent)	2	-1
Other malignant neoplasms	Male	Healthcare Access and Quality Index	2	-1
Other malignant neoplasms	Male	Age- and sex- specific SEV for low fruit	2	1
Other malignant neoplasms	Male	Age- and sex- specific SEV for Low vegetables	2	1
Other malignant neoplasms	Male	Age- and sex-specific SEV for Low nuts and seeds	2	1
Other malignant neoplasms	Female	Tobacco (cigarettes per capita)	1	1
Other malignant neoplasms	Female	Education (years per capita)	3	-1

Cause	Sex	Covariate	Level	Direction
Other malignant neoplasms	Female	LDI (I$ per capita)	3	1
Other malignant neoplasms	Female	Smoking Prevalence	1	1
Other malignant neoplasms	Female	Socio-demographic Index	3	1
Other malignant neoplasms	Female	pufa adjusted(percent)	2	-1
Other malignant neoplasms	Female	Healthcare Access and Quality Index	2	-1
Other malignant neoplasms	Female	Age- and sex- specific SEV for low fruit	2	1
Other malignant neoplasms	Female	Age- and sex- specific SEV for Low vegetables	2	1
Other malignant neoplasms	Female	Age- and sex-specific SEV for Low nuts and seeds	2	1
Other pharynx cancer	Male	Cumulative cigarettes (5 years)	2	1
Other pharynx cancer	Male	Education (years per capita)	3	-1
Other pharynx cancer	Male	LDI (I$ per capita)	3	1
Other pharynx cancer	Male	Population Density (over 1000 ppl/sqkm, proportion)	2	1
Other pharynx cancer	Male	Population Density (under 150 ppl/sqkm, proportion)	2	1
Other pharynx cancer	Male	Smoking Prevalence	1	1
Other pharynx cancer	Male	Log-transformed SEV scalar: Oth Phar C	1	1
Other pharynx cancer	Male	Socio-demographic Index	3	1
Other pharynx cancer	Male	Healthcare Access and Quality Index	2	-1
Other pharynx cancer	Male	Age- and sex- specific SEV for low fruit	2	1
Other pharynx cancer	Male	Age- and sex- specific SEV for Low vegetables	2	1
Other pharynx cancer	Male	Liters of alcohol consumed per capita	1	1
Other pharynx cancer	Female	Cumulative cigarettes (5 years)	2	1
Other pharynx cancer	Female	Education (years per capita)	3	-1
Other pharynx cancer	Female	LDI (I$ per capita)	3	1
Other pharynx cancer	Female	Population Density (over 1000 ppl/sqkm, proportion)	2	1
Other pharynx cancer	Female	Population Density (under 150 ppl/sqkm, proportion)	2	1
Other pharynx cancer	Female	Smoking Prevalence	1	1
Other pharynx cancer	Female	Log-transformed SEV scalar: Oth Phar C	1	1
Other pharynx cancer	Female	Socio-demographic Index	3	1
Other pharynx cancer	Female	Healthcare Access and Quality Index	2	-1
Other pharynx cancer	Female	Age- and sex- specific SEV for low fruit	2	1
Other pharynx cancer	Female	Age- and sex- specific SEV for Low vegetables	2	1
Other pharynx cancer	Female	Liters of alcohol consumed per capita	1	1
Ovarian cancer	Female	Contraception (Modern) Prevalence (proportion)	2	-1
Ovarian cancer	Female	Cumulative cigarettes (10 years)	2	1
Ovarian cancer	Female	Cumulative cigarettes (20 years)	2	1
Ovarian cancer	Female	Diabetes Age-Standardised Prevalence (proportion)	2	1
Ovarian cancer	Female	Education (years per capita)	3	-1
Ovarian cancer	Female	LDI (I$ per capita)	3	-1
Ovarian cancer	Female	Mean BMI	2	1

Cause	Sex	Covariate	Level	Direction
Ovarian cancer	Female	Smoking Prevalence	2	1
Ovarian cancer	Female	Total Fertility Rate	2	-1
Ovarian cancer	Female	Log-transformed SEV scalar: Ovary C	1	1
Ovarian cancer	Female	Socio-demographic Index	3	1
Ovarian cancer	Female	energy unadjusted(kcal)	2	1
Ovarian cancer	Female	Healthcare Access and Quality Index	2	-1
Ovarian cancer	Female	Asbestos consumption (metric tons per year per capita)	2	1
Ovarian cancer	Female	Age- and sex- specific SEV for low fruit	3	1
Ovarian cancer	Female	Age- and sex- specific SEV for Low vegetables	3	1
Ovarian cancer	Female	Liters of alcohol consumed per capita	1	1
Pancreatic cancer	Male	Tobacco (cigarettes per capita)	1	1
Pancreatic cancer	Male	Cumulative cigarettes (10 years)	1	1
Pancreatic cancer	Male	Cumulative cigarettes (20 years)	1	1
Pancreatic cancer	Male	Diabetes Fasting Plasma Glucose (mmol/L), age-standardised 25+	2	1
Pancreatic cancer	Male	Diabetes Age-Standardised Prevalence (proportion)	2	1
Pancreatic cancer	Male	Education (years per capita)	3	-1
Pancreatic cancer	Male	LDI (I$ per capita)	3	1
Pancreatic cancer	Male	Mean BMI	1	1
Pancreatic cancer	Male	Log-transformed SEV scalar: Pancreas C	1	1
Pancreatic cancer	Male	Socio-demographic Index	3	1
Pancreatic cancer	Male	energy unadjusted(kcal)	2	1
Pancreatic cancer	Male	Healthcare Access and Quality Index	2	-1
Pancreatic cancer	Male	Age- and sex- specific SEV for low fruit	3	1
Pancreatic cancer	Male	Age- and sex- specific SEV for Low vegetables	3	1
Pancreatic cancer	Male	Age- and sex-specific SEV for High red meat	2	1
Pancreatic cancer	Male	Liters of alcohol consumed per capita	2	1
Pancreatic cancer	Female	Tobacco (cigarettes per capita)	1	1
Pancreatic cancer	Female	Cumulative cigarettes (10 years)	1	1
Pancreatic cancer	Female	Cumulative cigarettes (20 years)	1	1
Pancreatic cancer	Female	Diabetes Fasting Plasma Glucose (mmol/L), age-standardised 25+	2	1
Pancreatic cancer	Female	Diabetes Age-Standardised Prevalence (proportion)	2	1
Pancreatic cancer	Female	Education (years per capita)	3	-1
Pancreatic cancer	Female	LDI (I$ per capita)	3	1
Pancreatic cancer	Female	Mean BMI	1	1
Pancreatic cancer	Female	Log-transformed SEV scalar: Pancreas C	1	1
Pancreatic cancer	Female	Socio-demographic Index	3	1
Pancreatic cancer	Female	energy unadjusted(kcal)	2	1
Pancreatic cancer	Female	Healthcare Access and Quality Index	2	-1

Cause	Sex	Covariate	Level	Direction
Pancreatic cancer	Female	Age- and sex- specific SEV for low fruit	3	1
Pancreatic cancer	Female	Age- and sex- specific SEV for Low vegetables	3	1
Pancreatic cancer	Female	Age- and sex-specific SEV for High red meat	2	1
Pancreatic cancer	Female	Liters of alcohol consumed per capita	2	1
Prostate cancer	Male	Education (years per capita)	3	-1
Prostate cancer	Male	LDI (I$ per capita)	3	-1
Prostate cancer	Male	Smoking Prevalence	2	1
Prostate cancer	Male	Log-transformed SEV scalar: Prostate C	1	1
Prostate cancer	Male	Socio-demographic Index	3	1
Prostate cancer	Male	Healthcare Access and Quality Index	2	-1
Stomach cancer	Male	Tobacco (cigarettes per capita)	1	1
Stomach cancer	Male	Cumulative cigarettes (20 years)	2	1
Stomach cancer	Male	Education (years per capita)	3	-1
Stomach cancer	Male	LDI (I$ per capita)	3	1
Stomach cancer	Male	Mean BMI	2	1
Stomach cancer	Male	Sanitation (proportion with access)	2	-1
Stomach cancer	Male	Improved Water Source (proportion with access)	2	-1
Stomach cancer	Male	Log-transformed SEV scalar: Stomach C	1	1
Stomach cancer	Male	Age- and sex-specific SEV for Unsafe water	2	1
Stomach cancer	Male	Age- and sex-specific SEV for Unsafe sanitation	2	1
Stomach cancer	Male	Socio-demographic Index	3	-1
Stomach cancer	Male	Healthcare Access and Quality Index	2	-1
Stomach cancer	Male	Diet high in sodium	1	1
Stomach cancer	Male	Age- and sex- specific SEV for low fruit	3	1
Stomach cancer	Male	Age- and sex- specific SEV for Low vegetables	3	1
Stomach cancer	Female	Tobacco (cigarettes per capita)	1	1
Stomach cancer	Female	Cumulative cigarettes (20 years)	2	1
Stomach cancer	Female	Education (years per capita)	3	-1
Stomach cancer	Female	LDI (I$ per capita)	3	1
Stomach cancer	Female	Mean BMI	2	1
Stomach cancer	Female	Sanitation (proportion with access)	2	-1
Stomach cancer	Female	Improved Water Source (proportion with access)	2	-1
Stomach cancer	Female	Log-transformed SEV scalar: Stomach C	1	1
Stomach cancer	Female	Age- and sex-specific SEV for Unsafe water	2	1
Stomach cancer	Female	Age- and sex-specific SEV for Unsafe sanitation	2	1
Stomach cancer	Female	Socio-demographic Index	3	-1
Stomach cancer	Female	Healthcare Access and Quality Index	2	-1
Stomach cancer	Female	Diet high in sodium	1	1
Stomach cancer	Female	Age- and sex- specific SEV for low fruit	3	1

Cause	Sex	Covariate	Level	Direction
Stomach cancer	Female	Age- and sex- specific SEV for Low vegetables	3	1
Testicular cancer	Male	Tobacco (cigarettes per capita)	2	1
Testicular cancer	Male	Cumulative cigarettes (10 years)	2	1
Testicular cancer	Male	Cumulative Cigarettes (15 Years)	2	1
Testicular cancer	Male	Cumulative cigarettes (20 years)	2	1
Testicular cancer	Male	Cumulative Cigarettes (5 Years)	2	1
Testicular cancer	Male	Education (years per capita)	3	-1
Testicular cancer	Male	LDI (I$ per capita)	3	1
Testicular cancer	Male	Smoking Prevalence	2	1
Testicular cancer	Male	Socio-demographic Index	3	1
Testicular cancer	Male	Healthcare Access and Quality Index	2	-1
Testicular cancer	Male	Age- and sex- specific SEV for low fruit	2	1
Testicular cancer	Male	Age- and sex- specific SEV for Low vegetables	2	1
Thyroid cancer	Male	Tobacco (cigarettes per capita)	2	1
Thyroid cancer	Male	Education (years per capita)	3	-1
Thyroid cancer	Male	LDI (I$ per capita)	3	1
Thyroid cancer	Male	Mean BMI	2	1
Thyroid cancer	Male	Sanitation (proportion with access)	3	-1
Thyroid cancer	Male	Improved Water Source (proportion with access)	3	-1
Thyroid cancer	Male	Log-transformed SEV scalar: Thyroid C	1	1
Thyroid cancer	Male	Socio-demographic Index	3	1
Thyroid cancer	Male	Healthcare Access and Quality Index	2	-1
Thyroid cancer	Male	Age- and sex- specific SEV for low fruit	3	1
Thyroid cancer	Male	Age- and sex- specific SEV for Low vegetables	2	1
Thyroid cancer	Male	Age- and sex-specific SEV for High red meat	2	1
Thyroid cancer	Male	Liters of alcohol consumed per capita	1	1
Thyroid cancer	Female	Tobacco (cigarettes per capita)	2	1
Thyroid cancer	Female	Education (years per capita)	3	-1
Thyroid cancer	Female	LDI (I$ per capita)	3	1
Thyroid cancer	Female	Mean BMI	2	1
Thyroid cancer	Female	Sanitation (proportion with access)	3	-1
Thyroid cancer	Female	Improved Water Source (proportion with access)	3	-1
Thyroid cancer	Female	Log-transformed SEV scalar: Thyroid C	1	1
Thyroid cancer	Female	Socio-demographic Index	3	1
Thyroid cancer	Female	Healthcare Access and Quality Index	2	-1
Thyroid cancer	Female	Age- and sex- specific SEV for low fruit	3	1
Thyroid cancer	Female	Age- and sex- specific SEV for Low vegetables	2	1
Thyroid cancer	Female	Age- and sex-specific SEV for High red meat	2	1
Thyroid cancer	Female	Liters of alcohol consumed per capita	1	1

Cause	Sex	Covariate	Level	Direction
Tracheal, bronchus, and lung cancer	Male	Cumulative cigarettes (10 years)	2	1
Tracheal, bronchus, and lung cancer	Male	Cumulative cigarettes (20 years)	2	1
Tracheal, bronchus, and lung cancer	Male	Diabetes Fasting Plasma Glucose (mmol/L), age-standardised 25+	2	1
Tracheal, bronchus, and lung cancer	Male	Education (years per capita)	3	-1
Tracheal, bronchus, and lung cancer	Male	LDI (I$ per capita)	3	1
Tracheal, bronchus, and lung cancer	Male	Indoor Air Pollution (All Cooking Fuels)	2	1
Tracheal, bronchus, and lung cancer	Male	Outdoor Air Pollution (PM2.5)	2	1
Tracheal, bronchus, and lung cancer	Male	Smoking Prevalence	1	1
Tracheal, bronchus, and lung cancer	Male	Log-transformed SEV scalar: Lung C	1	1
Tracheal, bronchus, and lung cancer	Male	Log-transformed age-standardised SEV scalar: Lung C	1	1
Tracheal, bronchus, and lung cancer	Male	Socio-demographic Index	3	1
Tracheal, bronchus, and lung cancer	Male	Healthcare Access and Quality Index	2	-1
Tracheal, bronchus, and lung cancer	Male	Residential radon	2	1
Tracheal, bronchus, and lung cancer	Male	Second-hand smoke	2	1
Tracheal, bronchus, and lung cancer	Male	Asbestos consumption (metric tons per year per capita)	1	1
Tracheal, bronchus, and lung cancer	Female	Cumulative cigarettes (10 years)	2	1
Tracheal, bronchus, and lung cancer	Female	Cumulative cigarettes (20 years)	2	1
Tracheal, bronchus, and lung cancer	Female	Diabetes Fasting Plasma Glucose (mmol/L), age-standardised 25+	2	1
Tracheal, bronchus, and lung cancer	Female	Education (years per capita)	3	-1
Tracheal, bronchus, and lung cancer	Female	LDI (I$ per capita)	3	1
Tracheal, bronchus, and lung cancer	Female	Indoor Air Pollution (All Cooking Fuels)	2	1
Tracheal, bronchus, and lung cancer	Female	Outdoor Air Pollution (PM2.5)	2	1
Tracheal, bronchus, and lung cancer	Female	Smoking Prevalence	1	1
Tracheal, bronchus, and lung cancer	Female	Log-transformed SEV scalar: Lung C	1	1
Tracheal, bronchus, and lung cancer	Female	Log-transformed age-standardised SEV scalar: Lung C	1	1
Tracheal, bronchus, and lung cancer	Female	Socio-demographic Index	3	1

Cause	Sex	Covariate	Level	Direction
Tracheal, bronchus, and lung cancer	Female	Healthcare Access and Quality Index	2	-1
Tracheal, bronchus, and lung cancer	Female	Residential radon	2	1
Tracheal, bronchus, and lung cancer	Female	Second-hand smoke	2	1
Tracheal, bronchus, and lung cancer	Female	Asbestos consumption (metric tons per year per capita)	1	1
Uterine cancer	Female	Tobacco (cigarettes per capita)	2	1
Uterine cancer	Female	Cumulative cigarettes (10 years)	2	1
Uterine cancer	Female	Cumulative Cigarettes (5 Years)	2	1
Uterine cancer	Female	Diabetes Age-Standardised Prevalence (proportion)	2	1
Uterine cancer	Female	Education (years per capita)	3	-1
Uterine cancer	Female	LDI (I$ per capita)	3	1
Uterine cancer	Female	Mean BMI	1	1
Uterine cancer	Female	Smoking Prevalence	2	1
Uterine cancer	Female	Total Fertility Rate	2	-1
Uterine cancer	Female	Log-transformed SEV scalar: Uterus C	1	1
Uterine cancer	Female	Socio-demographic Index	3	1
Uterine cancer	Female	Healthcare Access and Quality Index	2	-1
Uterine cancer	Female	Age- and sex- specific SEV for low fruit	2	1
Uterine cancer	Female	Age- and sex- specific SEV for Low vegetables	2	1

BMI = body-mass index
dr = data rich (model);
GBD = Global Burden of Disease Study;
glb = global (model);
HBsAg = Heptatitis B surface antigen;
HCV = Hepatitis C virus;
LDI = lag distributed income per capita (I$): gross domestic product per capita that has been smoothed over the preceding 10 years;
MET = metabolic equivalent of task;
PM2.5 = particulate matter $\leq$ 2.5 micrometres;
PUFA = polyunsaturated fatty acid;
SEV = summary exposure value: for definitions and calculations, please see Section 2.6: "Step 5. Estimate summary exposure values" in the Supplementary Appendix 1 to "GBD 2019 Risk Factors Collaborators. Global burden of 87 risk factors in 204 countries and territories, 1990–2019: a systematic analysis for the Global Burden of Disease Study 2019. Lancet 2020; 396: 1223–49".[22] ; covariates with "C" following a cancer site name refer to a cancer site (eg, uterus C = uterus cancer) and were shortened due to space limitations in covariate names.

CoDCorrect

CODEm models estimate the individual cause-level mortality without taking into account the independently modeled all-cause mortality (#13 in Appendix figure 1). To ensure that all single causes add up to the all-cause mortality and that all child-causes add up to the parent cause, an algorithm called "CoDCorrect" is used (#14 and #15 in Appendix figure 1). Further details on the CoDCorrect algorithm can be found in Section 3.3.2 of the Supplementary Appendix 1 to the GBD 2019 paper "Global burden of 369 diseases and injuries in 204 countries and territories, 1990–2019: a systematic analysis for the

Global Burden of Disease Study 2019”.[3] Final mortality estimates at the 1000-draw level provide an estimated mean mortality with 95% uncertainty interval.

Calculating YLLs

To calculate years of life lost (YLLs), final death estimates after CoDCorrect adjustment are multiplied by the standard GBD life expectancy given the age at death, sex, and location. Further details on GBD life expectancy values can be found in the GBD 2019 paper “Global age-sex-specific fertility, mortality, health life expectancy (HALE), and population estimates in 2014 countries and territories, 1950-2019: a comprehensive demographic analysis for the Global Burden of Disease Study 2019”.[25] Uncertainty is propagated from the CoDCorrect mortality estimates, calculating YLLs for each of the 1000 CoDCorrect draws to provide estimated mean YLLs with corresponding 95% uncertainty intervals.

Incidence estimation

The final GBD cancer mortality estimates (after CoDCorrect adjustment) were transformed to incidence estimates by using the MIRs specific to that cancer cause (#1 in Appendix figure 2). Final mortality estimates at the 1000-draw level were divided by the modeled MIR estimates (also at the 1000-draw level) to generate 1000 draws of incidence estimates (which provides an estimated mean incidence with 95% uncertainty interval). It was assumed that uncertainty in the MIRs is independent of uncertainty in the estimated mortality.

Prevalence estimation

After transforming the final GBD cancer mortality estimates to incidence estimates (step 1 in Appendix figure 2), incidence was combined with annual relative survival estimates from 1 to 10 years after diagnosis (step 7 in the Appendix figure 2). Previous reports suggest that the value of (1 – MIR) may serve as a proxy for 5-year relative survival, with the exact correlation varying slightly by cancer type.[26] Because this correlation varies, we trained cancer-specific prediction models to estimate 5-year survival from MIRs, using data from SEER[8]. We used SEER*Stat[27] to obtain mortality, incidence, and relative survival statistics from the nine SEER registries reporting from 1980-2014 (step 2), by cancer type, sex, 5-year blocks (ie, 1980-84, 1985-1989, etc.), and 5-year age groups (except combining 80+). For each cancer, we modelled SEER 5-year relative survival using MIRs calculated from SEER mortality and incidence. For GBD 2019 we updated this model from the Poisson regression used in GBD 2017[28] to using a generalised linear model with a quasibinomial family and logit link, weighted by the number of index cases (step 3 in Appendix figure 2). To reduce variability due to small samples, we only included MIRs based on at least 25 incident cases (except for the cancers mesothelioma, nasopharynx cancer, and acute lymphoid leukaemia, where MIRs based on at least 10 cases were included). These models were then applied to the GBD MIR estimates to predict an estimated 5-year survival for each age/sex/year/location (step 4). To prevent unrealistic values, predicted 5-year survival values were Winsorised to be between 0% and 100% survival.

To generate yearly survival estimates up to 10 years, we downloaded SEER[8] sex- and age-specific annual 1- through 10-year relative survival data from persons diagnosed between 2001 and 2010 (2001 through 2010 so that all cases had at least 5 years of follow-up, with half having the full 10 years of follow-up). This is updated from GBD 2017, where we downloaded all-ages survival data from persons diagnosed in 2004 (2004 so that all cases had the full 10 years of follow-up).[29] A proportional scalar was calculated as the predicted GBD 5-year survival estimate divided by the SEER 5-year survival statistic, and was then used to generate yearly survival estimates by scaling the 1-10 year SEER curve to the GBD survival predictions under the proportional hazard assumption (step 5).

The estimated relative survival is next transformed into absolute survival estimates (steps 6 and 7 in Appendix figure 2). To account for background mortality in the relative survival estimates, GBD 2019 lifetables were used to calculate lambda (λ) values:[25]

$$\lambda = \frac{\ln\left(\frac{nLx_n}{nLx_{n+1}}\right)}{5}$$

nLx = person-years lived between ages x and x+n (from GBD lifetable).

Absolute survival was then calculated using an exponential survival function:

$$absolute\ survival = relative\ survival * e^{\lambda * t}$$

t = time (in years)

Absolute survival is combined with incidence to estimate the prevalence at each year 1 through 10 after diagnosis, which is then split into the four sequelae (step 8 in the Appendix figure 2). For the purposes of calculating disability due to cancer, survivors beyond 10 years were considered cured. For this group, the survivor population prevalence was divided into two sequelae: 1) diagnosis and primary therapy phase; and 2) controlled phase. For the population that did not survive beyond 10 years, the yearly prevalence was divided into the four sequelae by assigning the fixed durations for each of the (1) diagnosis and primary therapy phase, (2) metastatic phase, and (3) terminal phase, and assigning the remaining prevalence to the (4) controlled phase (step 8 in Appendix figure 2). Appendix table 5 lists the durations of each, along with the sources used to determine their length.[30–35]

Appendix Table 5: Duration of four prevalence phases by cancer in GBD 2019

GBD Cause*	Diagnosis / Treatment (months)*	Remission (months)	Disseminated/ metastatic (months)*	Note	Terminal (months)
Oesophageal cancer	5.0[30]	The remission phase duration is calculated based on the remaining time after attributing other sequelae durations.	4.6[31]	SEER Summary Stage 1997 (Distant site/node involved) 1995-2000	1
Stomach cancer	5.2[30]		3.9[31]	SEER Summary Stage 1997 (Distant site/node involved) 1995-2000	1
Liver cancer	4.0		2.5[31]	SEER Summary Stage 1997 (Distant site/node involved) 1995-2000	1
Larynx cancer	5.3[30]		8.8[31]	SEER Stage IVc	1
Tracheal, bronchus, and lung cancer	3.3[32]		4.5[31]	SEER Summary Stage 1997 (Distant site/node involved) 1995-2000	1
Breast cancer	3.0[32]		17.7[31]	SEER Summary Stage 1997 (Distant site/node involved) 1995-2000	1

GBD Cause*	Diagnosis / Treatment (months)*	Remission (months)	Disseminated/ metastatic (months)*	Note	Terminal (months)
Cervical cancer	4.8[30]		9.2[31]	SEER Summary Stage 1997 (Distant site/node involved) 1995-2000	1
Uterine cancer	4.6[30]		11.6[31]	SEER Summary Stage 1997 (Distant site/node involved) 1995-2000	1
Prostate cancer	4.0[32]		30.4[31]	SEER Summary Stage 1997 (Distant site/node involved) 1995-2000	1
Colon and rectum cancer	4.0[32]		9.7[31]	SEER Summary Stage 1997 (Distant site/node involved) 1995-2000	1
Lip and oral cavity cancer	5.3[30]		9.3[31]	SEER Stage IVc	1
Nasopharynx cancer	5.3[30]	The remission phase duration is calculated based on the remaining time after attributing other sequelae durations.	13.2[31]	SEER Stage IVc	1
Other pharynx cancer	5.3[30]		7.9[31]	SEER Stage IVc	1
Gallbladder and biliary tract cancer	4.0		3.5[31]	SEER Summary Stage 1997 (Distant site/node involved) 1995-2000	1
Pancreatic cancer	4.1[30]		2.5[31]	SEER Summary Stage 1977 (Distant site/node involved) 1995-2000	1
Malignant skin melanoma	2.9[33]		7.2[31]	SEER Summary Stage 1977 (Distant site/node involved) 1995-2000	1
Ovarian cancer	3.2[32]		25.6[31]	SEER Summary Stage 1977 (Distant site/node involved) 1995-2000	1
Testicular cancer	3.7[30]		19.5[31]	SEER Stage III	1
Kidney cancer	5.3[30]		5.4[31]	SEER Summary Stage 1977 (Distant site/node involved) 1995-2000	1
Bladder cancer	5.1[30]		5.8[31]	SEER Summary Stage 1977 (Distant site/node involved) 1995-2000	1

GBD Cause*	Diagnosis / Treatment (months)*	Remission (months)	Disseminated/ metastatic (months)*	Note	Terminal (months)
Brain and central nervous system cancer	5.0		6.9[31]	SEER Median age standardised survival all patients, all years	1
Thyroid cancer	3.0		19.4[31]	SEER Stage IVc	1
Mesothelioma	4.0		7.8[31]	SEER Summary Stage 1977 (Distant site/node involved) 1995-2000	1
Hodgkin lymphoma	3.7[32]		26.0[34]		1
Non-Hodgkin lymphoma	3.7[32]		7.7[34]		1
Multiple myeloma	7.0[30]	The remission phase duration is calculated based on the remaining time after attributing other sequelae durations.	36.8[31]	SEER Median age standardised survival all patients, all years	1
Leukaemia[30]	5.0		43.7[31]	SEER Median age standardised survival all patients, all years	1
Acute lymphoid leukaemia	12.0		7.0[31]	SEER Median age standardised survival all patients, all years	1
Acute myeloid leukaemia	6.0		4.6[31]	SEER Median age standardised survival all patients, all years	1
Chronic lymphoid leukaemia	6.0		48[35]	SEER Median age standardised survival all patients, all years	1
Chronic myeloid leukaemia	6.0		4.6[31]	SEER Median age standardised survival for AML (patients with CML die in blast crisis, which is treated like AML) all patients, all years	1
Other leukaemia	6.0		48.0[35]	SEER Median age standardised survival all patients, all years	1
Other malignant neoplasms	4.4 (mean of other cancer durations)		15.8[31]	SEER Median age standardised survival all patients, all years	1

* Superscripts refer to references used to inform these values.

For cancer-specific procedure sequelae, hospital data were used to estimate the number of cancer patients undergoing mastectomy, laryngectomy, stoma, prostatectomy, and cystectomy (step 9 in Appendix figure 2). Proportions were generated by dividing the rate of procedures generated from the diagnostic codes in the hospital dataset and the coverage population by the GBD age-, and sex-specific disease incidence rates for that country.

To estimate procedure-related disability for each of these five cancers, the procedure proportions (proportion of each cancer population that undergo these procedures) from hospital data were used as input for a proportion model in DisMod-MR 2.1[22] to estimate the proportions for all locations, by age, year, and sex. Details of clinical and claims data processing are available in section 4.3.4 of the appendix to the GBD 2019 paper "Global burden of 369 diseases and injuries in 204 countries and territories, 1990–2019: a systematic analysis for the Global Burden of Disease Study 2019".[3]

Since colostomy or ileostomy procedures are done for reasons other than cancer, a literature review was conducted to determine the proportion of ostomies due to colon and rectum cancer. Based on the results of the literature review that an average of 58% of ostomies are done for colon and rectum cancer, the "all cause" colostomy proportions were multiplied by 0.58.[36–38]

The final procedure proportions were applied to the incident cases of the respective cancers and multiplied with the proportion of the incident population surviving for 10 years to determine the incident cases of the cancer population that underwent procedures and that survived beyond 10 years. These incident cases were used again as an input for DisMod-MR 2.1, with a remission specification of zero and an excess mortality rate prior of 0 to 0.1, as well as with increasing both the age of the population and the year by 10 years to reflect prevalence after that population has survived 10 years. The results from this model are incidence and lifetime prevalent cases of persons with these cancer-related sequelae who have survived beyond 10 years.

Since disability associated with prostatectomy comes from impotence and incontinence, and not from the prostatectomy itself, 18% of the prostatectomy prevalence was assumed to have incontinence and 55% was assumed to have impotence, based on a literature review done for GBD 2013.[39–46] Cases were assigned disability for either impotence or incontinence, but no cases were assigned disability from both.

We assumed that for the population surviving up to 10 years, only the prevalence population being in remission experiences additional disability due to procedures (eg, women suffering from metastatic breast cancer do not experience additional disability due to a mastectomy during this phase). To estimate the prevalence of the cancer population in remission during the first 10 years after diagnosis with and without procedure-related disability, we multiplied the prevalence of the population in the remission phase with the proportion of the population undergoing a procedure. This step allowed us to estimate disability during the remission phase for both the population experiencing disability due to the remission phase alone, as well as the population experiencing disability from the remission phase and the additional procedure-related disability.

Lastly, the procedure sequelae prevalence and general sequelae prevalence were multiplied with their respective disability weights (Appendix table 6) to obtain the number of YLDs (steps 11 and 12 Appendix figure 2). A description of non-procedure disability weights calculations can be found in "Section 4.8: Disability weights" in the Supplementary appendix 1 to "Global burden of 369 diseases and injuries in 204 countries and territories, 1990–2019: a systematic analysis for the Global Burden of Disease Study 2019".[3] In brief, disability weights are created from survey data to represent the magnitude of health loss associated with an outcome. These disability weights range from 0, implying a state equivalent to full health, to 1, a state equivalent to death. The sum of these YLDs is the final YLD estimate associated with each cancer cause.

Appendix Table 6: Lay description of cancer states and corresponding disability weights in GBD 2019

Health state	Lay description	Disability weight (95% uncertainty interval)
Cancer, diagnosis and primary therapy *All cancers*	This person has pain, nausea, fatigue, weight loss and high anxiety.	0.288 (0.193 to 0.399)
Cancer, controlled phase *All cancers*	This person has a chronic disease that requires medication every day and causes some worry but minimal interference with daily activities.	0.049 (0.031 to 0.072)
Cancer, metastatic *All cancers*	This person has severe pain, extreme fatigue, weight loss and high anxiety.	0.451 (0.307 to 0.600)
Terminal phase, with medication *All cancers*	This person has lost a lot of weight and regularly uses strong medication to avoid constant pain. The person has no appetite, feels nauseous, and needs to spend most of the day in bed.	0.540 (0.377 to 0.687)
Mastectomy *Breast cancer*	This person had one of her breasts removed and sometimes has pain or swelling in the arms.	0.036 (0.020 to 0.057)
Stoma *Colon and rectum cancer*	This person has a pouch attached to an opening in the belly to collect and empty stools.	0.095 (0.063 to 0.131)
Laryngectomy *Larynx cancer*	This person has difficulty speaking, and others find it difficult to understand.	0.051 (0.032 to 0.078)
Urinary incontinence *Bladder cancer; Prostate cancer*	This person cannot control urinating.	0.139 (0.094 to 0.198)
Impotence *Prostate cancer*	This person has difficulty in obtaining or maintaining an erection.	0.017 (0.009 to 0.030)

Calculating Proportional Burden

Proportion burden was calculated by taking the proportion of the 1000 draws (numerator draws/denominator draws) to get the proportion draws, then taking the mean and 95% UIs of the 1000 draws to get the proportion mean and 95% UIs. For example, for calculating proportional DALY burden the numerator draws would be the DALY burden for an individual cancer for a specific age group, year, sex, location, while denominator draws would be the DALY burden for the AYA cancer aggregate for a specific age group, year, sex, location.

This manuscript presents that the Global Initiative for Childhood Cancer (GICC)[47] covers only 8.6% [95% UI 8.2–9.1] of all AYA cancer cases. In order to estimate this, the following calculation was used: [(*total cases of AYA cancers globally in 15-19 year olds, both sexes, in 2019) /* (*total cases of AYA cancers globally in 15-39 year olds, both sexes, in 2019*)]

This analysis also includes an assessment of the proportional burden of AYA cancer cases that could be covered by existing WHO cancer initiatives, such as the GICC[47] and the cervical cancer elimination initiative.[48] This proportional incidence burden was calculated as follows:

$$\frac{a+b}{c}$$

a = total cases of AYA cancers globally in 15-19 year olds, both sexes in 2019
b = total cases of cervical cancer globally in 15-39 year old, females, in 2019
c = total cases of AYA cancers globally in 15-39 year olds, both sexes, in 2019

Similar to the above, this calculation took the proportion of the 1000 draws (numerator draws/denominator draws) to get the proportion draws, then took the mean and 95% UIs of the 1000 draws to get the proportion mean and 95% UIs.

Reporting Standards

The GBD world population age standard was used to calculate age-standardised rates presented throughout GBD. In GBD 2019, we used the non-weighted mean of the GBD year's age-specific proportional distributions for national locations with populations greater than 5 million in the GBD year to update the world population age standard.[3] Age-standardised rates for this analysis were calculated across the 15-39 year age group with the same methodology used to calculate this for all ages in the GBD 2019 study. The final values used for the age standard are specified in Appendix table 13 of the GBD 2019 paper "Global age-sex-specific fertility, mortality, health life expectancy (HALE), and population estimates in 204 countries and territories, 1950-2019: a comprehensive demographic analysis for the Global Burden of Disease Study 2019".[25]

Socio-demographic Index (SDI) Definition and Calculation

Socio-demographic Index (SDI) is a summary indicator to represent background levels of social and economic conditions that can influence health outcomes in a given location. This summary indicator comprises three indices: lag-distributed income per capita, mean education for those aged 15 years or older, and total fertility rate for those younger than 25 years of age. Possible values for each of these three indices range from 0 to 1, representing the bounds with which lower or higher values of the level of development for that index would no longer worsen or improve health outcomes, respectively. The composite SDI is the geometric mean of these three indices for a given location-year. For reporting purposes, values were multiplied by 100 to obtain SDI on a scale of 0 to 100. The SDI cutoffs for determining SDI quintiles for analysis were computed by using the country-level estimates of SDI for the year 2019, excluding countries with populations less than 1 million. For GBD 2019 analyses, all locations are assigned to these quintiles according to their SDI value in the year 2019. See Section 6 in Supplementary Appendix 1 to the GBD 2019 Diseases & Injuries capstone[3] for more details regarding

SDI estimation, and page 60 of this Appendix for the SDI quintile estimate for each country in GBD 2019.

Uncertainty Estimation

Uncertainty in cancer estimates begins with the availability of and variability in cancer cause-specific data by age, sex, location and year. The uncertainty in cancer mortality estimates arises from CODEm and CoDCorrect. For more information see the CODEm methodology paper by Foreman et al., and Supplementary Appendix 1 to "Global burden of 369 diseases and injuries in 204 countries and territories, 1990–2019: a systematic analysis for the global burden of disease study 2019".[3,24] Uncertainty in cancer incidence estimates results from both the uncertainty in mortality estimates as well as the uncertainty in the MIR estimates, which result from the ST-GPR models. Uncertainty from the mortality estimates and the MIRs were assumed to be independent. Cancer prevalence uncertainty results from both the incidence uncertainty as well as the uncertainty from survival estimates. These were assumed to be independent. Uncertainty in cancer YLD estimation results from the uncertainty in the prevalence of each cancer sequela and uncertainty in the disability weight and is propagated into the final comorbidity-corrected YLD result. The uncertainty in prevalence and the uncertainty in disability weights are assumed to have no correlation. Cancer YLL uncertainty results from uncertainty in mortality estimates as well as uncertainty in life expectancy estimates. Uncertainty in cancer DALY estimates results from the uncertainty in YLLs and the uncertainty in YLDs, which were assumed to be independent. The same technique for propagating uncertainty elsewhere in the GBD study is applied in the cancer estimation process. In brief, the distribution of each step in the computation process is stored in 1000 draws. The distributions are determined from the data input sampling error, the uncertainty of the model coefficients, and the uncertainty of severity distributions and disability weights. The 1000 draws are used for every step in the process, with final estimates computed using the mean estimate across 1000 draws. The 95% uncertainty intervals are determined by the 25th and 975th ranked values across all 1000 draws.[3] More specific information regarding uncertainty intervals can be found in the GBD 2019 capstone papers.[3,22,25]

Limitations

There are certain limitations to consider when interpreting the GBD mortality cancer estimates. First, even though every effort is made to include the most recently available data for each country, data seeking resources are not limitless and new data cannot always be accessed as soon as they are made available. It is therefore possible that the GBD study does not include all available data sources for cancer incidence or cancer mortality. Second, different redistribution methods can potentially change the cancer estimates substantially if the data sources used for the estimated location contain a large number of undefined causes; however, neglecting to account for these undefined deaths would likely introduce an even greater bias in the disease estimates. Third, using mortality-to-incidence ratios to transform cancer registry incidence data to mortality estimates requires accurate MIRs. For GBD 2019 we have made further refinements to the estimation of MIRs, but the method remains sensitive to under-diagnosis of cancer cases or under-ascertainment of cancer deaths. However, given that the majority of data used for the cancer mortality estimation come from vital registration data and not cancer registry data, this is not a major limitation. Finally, no estimates are available for some locations, such as Western Sahara and French Guiana, as they were not modelled locations in the Global Burden of Diseases, Injuries, and Risk Factors Study 2019. These countries are shaded white in the global map figures included in this paper.

Additional Methodology Tables and Figures

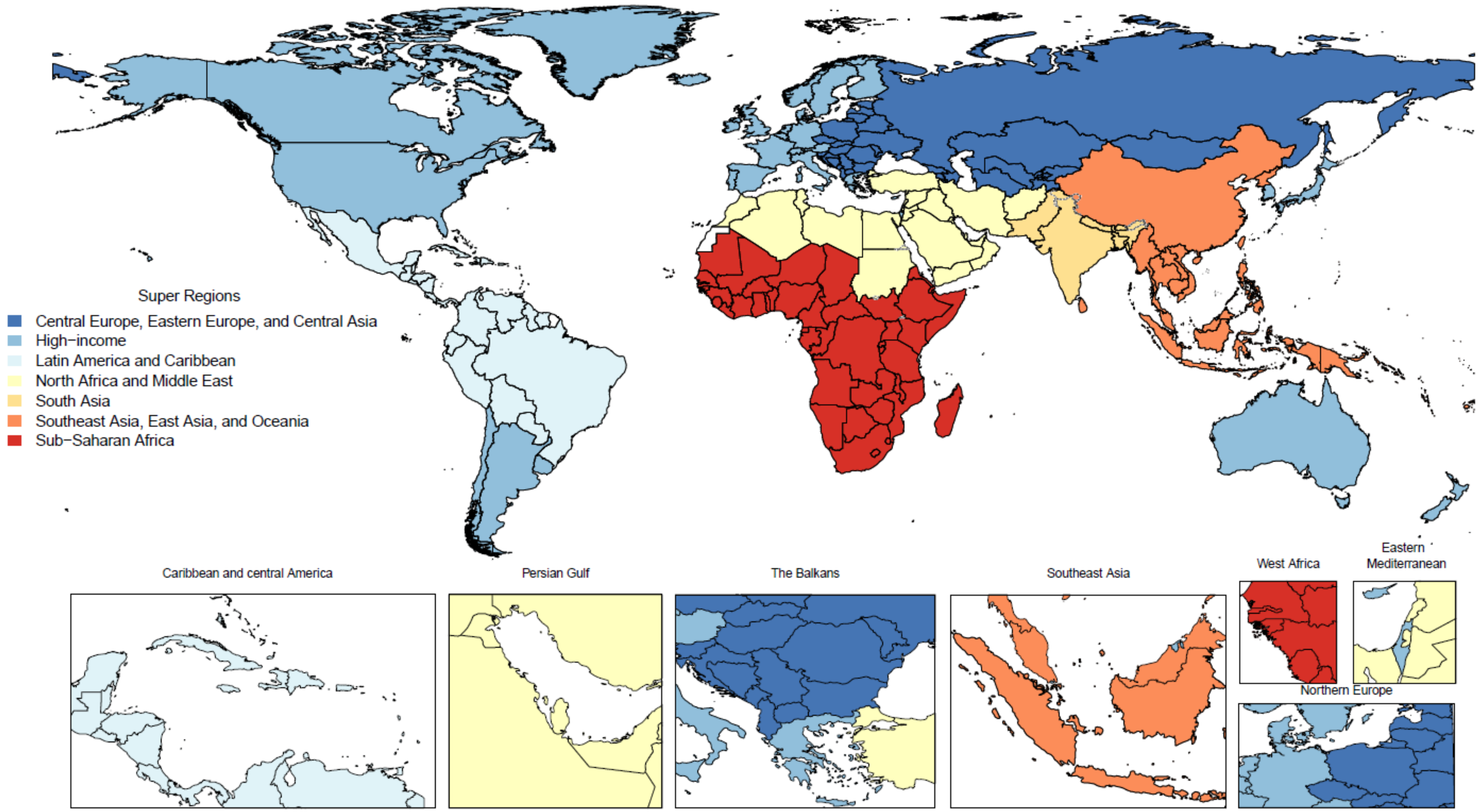

Appendix Figure 3: Map of GBD world super-regions, 2019. GBD=Global Burden of Diseases, Injuries, and Risk Factors Study.

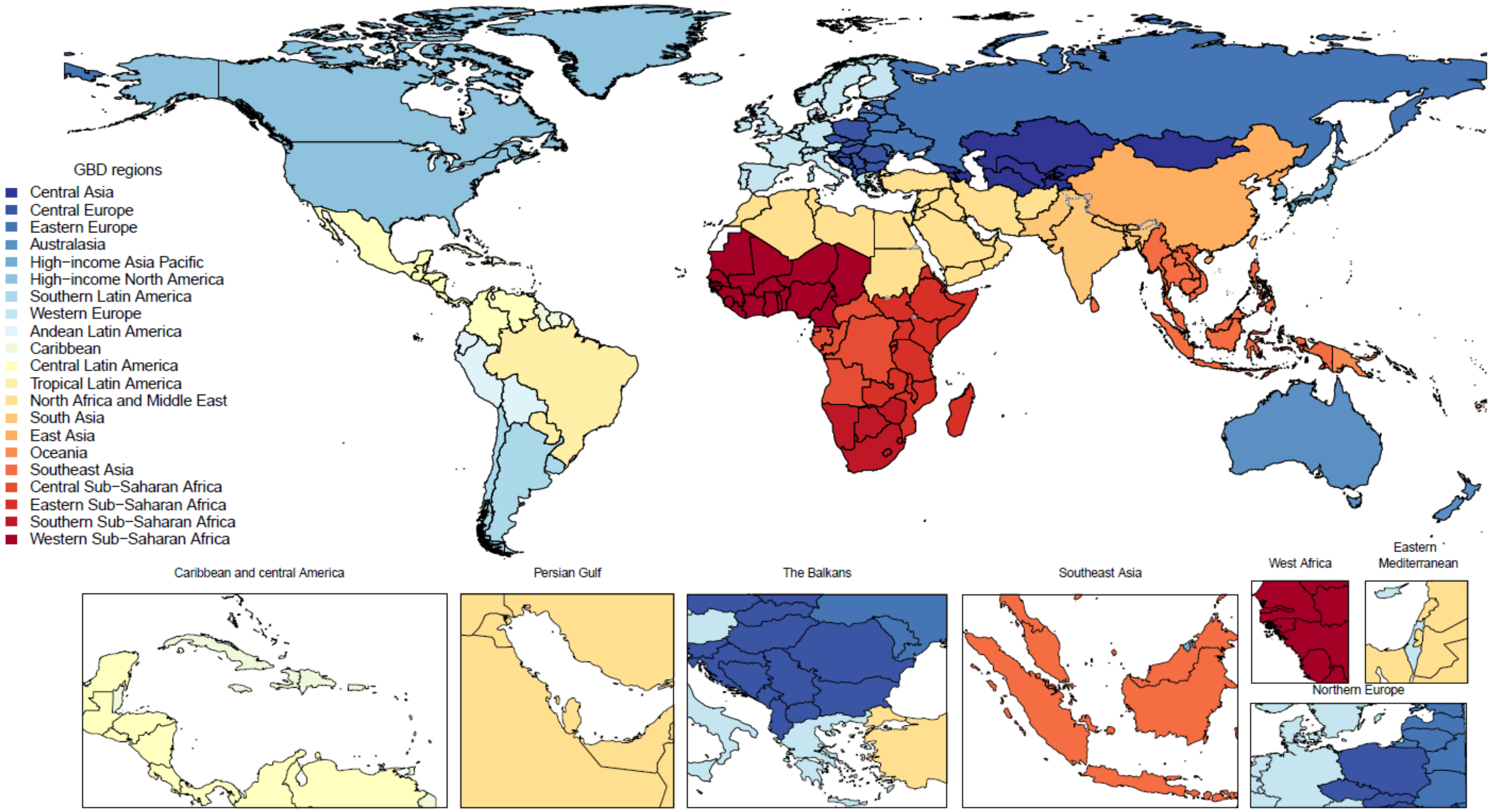

Appendix Figure 4: Map of GBD world regions, 2019. GBD=Global Burden of Diseases, Injuries, and Risk Factors Study.

Appendix Table 7: SDI quintiles for countries estimated in GBD 2019[25]

SDI Quintile	Locations included based on SDI values in 2019 from GBD 2019 results
High SDI	Andorra, Australia, Austria, Belgium, Bermuda, Brunei Darussalam, Canada, Cyprus, Czechia, Denmark, Estonia, Finland, France, Germany, Guam, Iceland, Ireland, Japan, Kuwait, Latvia, Lithuania, Luxembourg, Monaco, Netherlands, New Zealand, Norway, Puerto Rico, Qatar, San Marino, Saudi Arabia, Singapore, Slovakia, Slovenia, South Korea, Sweden, Switzerland, Taiwan (Province of China), United Arab Emirates, United Kingdom, United States of America
High-middle SDI	American Samoa, Antigua and Barbuda, Argentina, Bahamas, Bahrain, Barbados, Belarus, Bosnia and Herzegovina, Bulgaria, Chile, Cook Islands, Croatia, Dominica, Georgia, Greece, Greenland, Hungary, Israel, Italy, Jordan, Kazakhstan, Lebanon, Libya, Malaysia, Malta, Mauritius, Montenegro, Niue, North Macedonia, Northern Mariana Islands, Oman, Palau, Poland, Portugal, Republic of Moldova, Romania, Russia, Saint Kitts and Nevis, Serbia, Seychelles, Spain, Sri Lanka, Trinidad and Tobago, Turkey, Ukraine, United States Virgin Islands, Uruguay
Middle SDI	Albania, Algeria, Armenia, Azerbaijan, Botswana, Brazil, China, Colombia, Costa Rica, Cuba, Ecuador, Egypt, Equatorial Guinea, Fiji, Gabon, Grenada, Guyana, Indonesia, Iran (Islamic Republic of), Iraq, Jamaica, Mexico, Namibia, Nauru, Panama, Paraguay, Peru, Philippines, Saint Lucia, Saint Vincent and the Grenadines, Samoa, South Africa, Suriname, Syrian Arab Republic, Thailand, Tokelau, Tonga, Tunisia, Turkmenistan, Uzbekistan, Vietnam
Low-middle SDI	Angola, Bangladesh, Belize, Bhutan, Bolivia (Plurinational State of), Cape Verde, Cambodia, Cameroon, Congo (Brazzaville), Djibouti, Dominican Republic, El Salvador, Eswatini, Federated States of Micronesia, Ghana, Guatemala, Honduras, India, Kenya, Kiribati, Kyrgyzstan, Laos, Lesotho, Maldives, Marshall Islands, Mauritania, Mongolia, Morocco, Myanmar, Nicaragua, Nigeria, North Korea, Palestine, São Tomé and Príncipe, Sudan, Tajikistan, Timor-Leste, Tuvalu, Vanuatu, Venezuela (Bolivarian Republic of), Zambia, Zimbabwe
Low SDI	Afghanistan, Benin, Burkina Faso, Burundi, Central African Republic, Chad, Comoros, Côte d'Ivoire, DR Congo, Eritrea, Ethiopia, The Gambia, Guinea, Guinea-Bissau, Haiti, Liberia, Madagascar, Malawi, Mali, Mozambique, Nepal, Niger, Pakistan, Papua New Guinea, Rwanda, Senegal, Sierra Leone, Solomon Islands, Somalia, South Sudan, Tanzania, Togo, Uganda, Yemen

SDI values for all estimated GBD 2019 locations in 2019 can be found in Appendix table 16 in the Supplementary appendix 1 to "Global age-sex-specific fertility, mortality, healthy life expectancy (HALE), and population estimates in 204 countries and territories, 1950–2019: a comprehensive demographic analysis for the Global Burden of Disease Study 2019".[25]

Appendix Table 8: Population of 15-39 year-old individuals in the locations comprising each SDI Quintile in GBD 2019

SDI Quintile	Absolute population, both sexes, millions (95% UI)	% of Total population (both sexes) in that SDI quintile that is in the 15-39 year age group (95% UI)	Absolute population, males, in millions (95% UI)	% of Total male population in that SDI quintile that is in the 15-39 year age group (95% UI)	Absolute population, females, in millions (95% UI)	% of Total female population in that SDI quintile that is in the 15-39 year age group (95% UI)	% of Total world 15-39 year-old population (both sexes) that falls in each SDI quintile (95% UI)
High SDI	331 (316 to 347)	32.7 (32.6 to 32.8)	171 (163 to 179)	33.9 (33.8 to 34.0)	160 (152 to 167)	31.5 (31.4 to 31.6)	11.2 (10.6 to 11.8)
High-middle SDI	517 (488 to 544)	36.1 (36.0 to 36.2)	267 (252 to 282)	37.4 (37.3 to 37.5)	250 (236 to 263)	34.9 (34.7 to 35.0)	17.4 (16.8 to 18.0)
Middle SDI	935 (894 to 977)	39.0 (38.8 to 39.2)	472 (451 to 493)	39.2 (38.9 to 39.4)	463 (443 to 483)	38.9 (38.7 to 39.1)	31.5 (30.8 to 32.2)
Low-middle SDI	735 (697 to 775)	41.7 (41.6 to 41.8)	369 (350 to 390)	41.7 (41.6 to 41.7)	366 (347 to 385)	41.7 (41.6 to 41.8)	24.8 (24.0 to 25.6)
Low SDI	448 (429 to 466)	39.7 (39.6 to 39.7)	222 (212 to 231)	39.2 (39.1 to 39.3)	226 (217 to 235)	40.2 (40.1 to 40.2)	15.1 (14.5 to 15.7)

Additional Results in Tables and Figures

Appendix Table 9: Adolescent and young adult cancer DALYs, incidence, and mortality burden globally and by SDI quintile in 2019, males.

	DALYs, thousands (95% UI)	Age-standardised DALY rate (95% UI)	Incidence, thousands (95% UI)	Age-standardised Incidence rate (95% UI)	Mortality, thousands (95% UI)	Age-standardised Mortality rate (95% UI)
**Global, Total AYA Cancers**	11 500 (10 600 - 12 400)	760.3 (702.4 - 819.3)	509 (469 - 549)	33.5 (30.9 - 36.2)	194 (179 - 209)	12.8 (11.8 - 13.8)
**SDI Quintiles, Total AYA Cancers**						
High SDI Countries	1 000 (957 - 1 060)	548.8 (523.4 - 580.2)	94.9 (86.6 - 106)	52.2 (47.6 - 58.4)	16.6 (15.8 - 17.5)	8.9 (8.5 - 9.4)
High-middle SDI Countries	2 460 (2 250 - 2 700)	854.7 (782.2 - 932.6)	144 (131 - 159)	50.1 (45.2 - 55.1)	41.9 (38.1 - 45.7)	14.3 (13.0 - 15.6)
Middle SDI Countries	4 020 (3 660 - 4 410)	834.8 (759.5 - 914.7)	159 (143 - 176)	32.9 (29.7 - 36.4)	68.1 (61.6 - 74.8)	14.1 (12.7 - 15.4)
Low-middle SDI Countries	2 670 (2 400 - 2 980)	742.6 (668.0 - 829.3)	76.0 (67.9 - 84.1)	21.2 (18.9 - 23.4)	44.8 (40.3 - 49.8)	12.6 (11.3 - 14.0)
Low SDI Countries	1 350 (1 160 - 1 540)	649.4 (556.7 - 737.0)	34.4 (29.5 - 39.7)	16.6 (14.2 - 19.1)	22.3 (19.1 - 25.3)	10.9 (9.4 - 12.4)
**Global, per Cancer**						
Acute lymphoid leukaemia	479 (380 - 540)	32.2 (25.6 - 36.3)	22.0 (17.5 - 24.9)	1.5 (1.2 - 1.7)	7.32 (5.73 - 8.25)	0.5 (0.4 - 0.6)
Acute myeloid leukaemia	401 (336 - 500)	26.7 (22.4 - 33.3)	10.3 (8.67 - 12.8)	0.7 (0.6 - 0.9)	6.53 (5.43 - 8.16)	0.4 (0.4 - 0.5)
Bladder cancer	86.4 (76.5 - 97.7)	5.7 (5.0 - 6.4)	10.7 (9.40 - 12.2)	0.7 (0.6 - 0.8)	1.42 (1.26 - 1.61)	0.1 (0.1 - 0.1)

	DALYs, thousands (95% UI)	Age-standardised DALY rate (95% UI)	Incidence, thousands (95% UI)	Age-standardised Incidence rate (95% UI)	Mortality, thousands (95% UI)	Age-standardised Mortality rate (95% UI)
Brain and central nervous system cancer	1 030 (755 - 1 190)	67.8 (50.0 - 78.4)	33.2 (24.2 - 38.2)	2.2 (1.6 - 2.5)	17.2 (12.7 - 19.9)	1.1 (0.8 - 1.3)
Breast cancer	20.9 (18.7 - 23.3)	1.4 (1.2 - 1.5)	1.08 (0.979 - 1.20)	0.1 (0.1 - 0.1)	0.346 (0.307 - 0.388)	0.0 (0.0 - 0.0)
Chronic lymphoid leukaemia	34.9 (26.7 - 41.7)	2.3 (1.8 - 2.7)	2.28 (1.70 - 2.71)	0.1 (0.1 - 0.2)	0.570 (0.437 - 0.681)	0.0 (0.0 - 0.0)
Chronic myeloid leukaemia	174 (145 - 210)	11.5 (9.6 - 13.9)	5.23 (4.51 - 6.01)	0.3 (0.3 - 0.4)	2.93 (2.45 - 3.54)	0.2 (0.2 - 0.2)
Colon and rectum cancer	973 (887 - 1 070)	63.7 (58.1 - 70.1)	46.8 (42.1 - 52.0)	3.1 (2.8 - 3.4)	16.9 (15.4 - 18.6)	1.1 (1.0 - 1.2)
Gallbladder and biliary tract cancer	61.3 (51.6 - 69.3)	4.0 (3.4 - 4.5)	1.87 (1.54 - 2.09)	0.1 (0.1 - 0.1)	1.11 (0.934 - 1.25)	0.1 (0.1 - 0.1)
Hodgkin lymphoma	302 (242 - 381)	20.1 (16.0 - 25.3)	18.1 (15.6 - 22.0)	1.2 (1.0 - 1.5)	4.86 (3.87 - 6.17)	0.3 (0.3 - 0.4)
Kidney cancer	153 (138 - 170)	10.0 (9.1 - 11.2)	12.7 (11.4 - 14.2)	0.8 (0.7 - 0.9)	2.59 (2.34 - 2.90)	0.2 (0.2 - 0.2)
Larynx cancer	90.8 (82.2 - 101)	5.9 (5.4 - 6.6)	3.06 (2.77 - 3.37)	0.2 (0.2 - 0.2)	1.62 (1.46 - 1.81)	0.1 (0.1 - 0.1)
Lip and oral cavity cancer	368 (311 - 425)	24.1 (20.4 - 27.9)	17.2 (14.7 - 19.8)	1.1 (1.0 - 1.3)	6.43 (5.45 - 7.42)	0.4 (0.4 - 0.5)
Liver cancer	795 (690 - 905)	52.0 (45.2 - 59.3)	19.4 (16.7 - 22.1)	1.3 (1.1 - 1.4)	14.2 (12.3 - 16.2)	0.9 (0.8 - 1.1)
Malignant skin melanoma	136 (101 - 180)	8.9 (6.6 - 11.8)	16.0 (11.4 - 22.4)	1.0 (0.7 - 1.5)	2.28 (1.69 - 3.01)	0.1 (0.1 - 0.2)

	DALYs, thousands (95% UI)	Age-standardised DALY rate (95% UI)	Incidence, thousands (95% UI)	Age-standardised Incidence rate (95% UI)	Mortality, thousands (95% UI)	Age-standardised Mortality rate (95% UI)
Mesothelioma	29.8 (26.1 - 34.5)	2.0 (1.7 - 2.3)	0.756 (0.660 - 0.876)	0.0 (0.0 - 0.1)	0.524 (0.460 - 0.607)	0.0 (0.0 - 0.0)
Multiple myeloma	60.0 (45.1 - 69.1)	3.9 (3.0 - 4.5)	1.83 (1.39 - 2.12)	0.1 (0.1 - 0.1)	1.05 (0.801 - 1.21)	0.1 (0.1 - 0.1)
Nasopharynx cancer	249 (225 - 275)	16.4 (14.8 - 18.1)	20.2 (17.3 - 23.5)	1.3 (1.1 - 1.5)	4.18 (3.79 - 4.61)	0.3 (0.2 - 0.3)
Non-Hodgkin lymphoma	809 (747 - 889)	53.7 (49.6 - 59.0)	33.3 (29.8 - 37.4)	2.2 (2.0 - 2.5)	13.2 (12.2 - 14.5)	0.9 (0.8 - 1.0)
Oesophageal cancer	228 (200 - 260)	14.9 (13.1 - 17.0)	5.26 (4.66 - 5.98)	0.3 (0.3 - 0.4)	4.15 (3.65 - 4.75)	0.3 (0.2 - 0.3)
Other leukaemia	552 (437 - 643)	36.8 (29.1 - 42.7)	16.8 (13.2 - 19.3)	1.1 (0.9 - 1.3)	8.89 (7.02 - 10.4)	0.6 (0.5 - 0.7)
Other malignant neoplasms	1 880 (1 640 - 2 120)	125.8 (109.2 - 141.3)	77.4 (68.9 - 85.9)	5.2 (4.6 - 5.7)	30.0 (26.0 - 33.6)	2.0 (1.7 - 2.2)
Other pharynx cancer	157 (130 - 186)	10.3 (8.5 - 12.2)	4.48 (3.83 - 5.16)	0.3 (0.3 - 0.3)	2.82 (2.33 - 3.33)	0.2 (0.2 - 0.2)
Pancreatic cancer	277 (250 - 310)	18.1 (16.3 - 20.3)	6.12 (5.50 - 6.83)	0.4 (0.4 - 0.4)	5.02 (4.54 - 5.63)	0.3 (0.3 - 0.4)
Prostate cancer	54.3 (47.2 - 66.1)	3.6 (3.1 - 4.3)	5.47 (4.78 - 6.55)	0.4 (0.3 - 0.4)	0.876 (0.757 - 1.06)	0.1 (0.0 - 0.1)
Stomach cancer	842 (767 - 928)	55.1 (50.1 - 60.7)	27.9 (25.0 - 31.0)	1.8 (1.6 - 2.0)	15.0 (13.6 - 16.5)	1.0 (0.9 - 1.1)
Testicular cancer	349 (319 - 383)	23.1 (21.1 - 25.3)	57.4 (51.6 - 65.1)	3.8 (3.4 - 4.3)	5.35 (4.92 - 5.84)	0.4 (0.3 - 0.4)

	DALYs, thousands (95% UI)	Age-standardised DALY rate (95% UI)	Incidence, thousands (95% UI)	Age-standardised Incidence rate (95% UI)	Mortality, thousands (95% UI)	Age-standardised Mortality rate (95% UI)
Thyroid cancer	69.1 (61.5 - 76.6)	4.6 (4.1 - 5.0)	12.3 (11.0 - 13.5)	0.8 (0.7 - 0.9)	1.08 (0.969 - 1.19)	0.1 (0.1 - 0.1)
Tracheal, bronchus, and lung cancer	856 (766 - 952)	56.1 (50.2 - 62.3)	19.8 (17.6 - 22.0)	1.3 (1.2 - 1.4)	15.3 (13.7 - 17.0)	1.0 (0.9 - 1.1)

Estimates are for 15–39-year-olds, males. Rates are reported per 100,000 person-years. Age-standardisation used the GBD 2019 world standard. SDI categories do not sum precisely to the global total as the GBD study does not provide separate estimates for all locations globally and an adjustment factor is made between all estimated locations which have corresponding SDI values and the global estimate. Total AYA Cancers=all malignant neoplasms in this age group excluding non-melanoma skin cancers. DALYs=disability-adjusted life-years. SDI=Socio-demographic Index. GBD=Global Burden of Diseases, Injuries, and Risk Factors Study. UI=uncertainty interval. Other malignant neoplasms are cancers without a detailed GBD cause separately listed.

Appendix Table 10: Adolescent and young adult cancer DALYs, incidence, and mortality burden globally and by SDI quintile in 2019, females.

	DALYs, thousands (95% UI)	Age-standardised DALYs rate (95% UI)	Incidence, thousands (95% UI)	Age-standardised Incidence rate (95% UI)	Mortality, thousands (95% UI)	Age-standardised Mortality rate (95% UI)
**Global, Total AYA Cancers**	12 000 (10 900 - 13 100)	803.9 (730.6 - 876.9)	686 (622 - 751)	45.9 (41.7 - 50.3)	202 (184 - 222)	13.6 (12.3 - 14.8)
**SDI Quintiles, Total AYA Cancers**						
High SDI Countries	1 010 (972 - 1 060)	580.9 (556.8 - 609.2)	118 (105 - 133)	67.5 (59.9 - 75.9)	16.8 (16.2 - 17.5)	9.5 (9.1 - 9.9)
High-middle SDI Countries	2 050 (1 850 - 2 270)	744.0 (672.8 - 822.0)	157 (139 - 177)	56.5 (50.0 - 63.6)	34.7 (31.3 - 38.7)	12.4 (11.2 - 13.8)
Middle SDI Countries	3 760 (3 400 - 4 160)	784.2 (708.6 - 865.8)	211 (188 - 235)	43.7 (39.0 - 48.8)	63.7 (57.4 - 70.6)	13.2 (11.9 - 14.6)
Low-middle SDI Countries	3 300 (2 890 - 3 720)	930.5 (814.4 - 1050.0)	133 (117 - 149)	37.5 (33.0 - 42.0)	55.8 (48.7 - 63.0)	15.8 (13.8 - 17.9)
Low SDI Countries	1 840 (1 570 - 2 150)	906.9 (771.0 - 1059.8)	66.3 (55.5 - 77.6)	33.0 (27.6 - 38.9)	31.2 (26.5 - 36.5)	15.6 (13.3 - 18.3)
**Global, per Cancer**						
Acute lymphoid leukaemia	287 (210 - 338)	19.8 (14.6 - 23.3)	16.8 (12.5 - 20.1)	1.1 (0.9 - 1.4)	4.37 (3.21 - 5.14)	0.3 (0.2 - 0.4)
Acute myeloid leukaemia	347 (298 - 397)	23.7 (20.3 - 27.1)	9.87 (8.47 - 11.3)	0.7 (0.6 - 0.8)	5.64 (4.86 - 6.47)	0.4 (0.3 - 0.4)
Bladder cancer	38.1 (34.1 - 42.3)	2.6 (2.3 - 2.8)	3.42 (3.04 - 3.83)	0.2 (0.2 - 0.3)	0.632 (0.563 - 0.701)	0.0 (0.0 - 0.0)
Brain and central nervous system cancer	722 (536 - 827)	48.9 (36.3 - 56.0)	28.3 (21.7 - 33.2)	1.9 (1.5 - 2.2)	11.9 (8.88 - 13.7)	0.8 (0.6 - 0.9)

	DALYs, thousands (95% UI)	Age-standardised DALYs rate (95% UI)	Incidence, thousands (95% UI)	Age-standardised Incidence rate (95% UI)	Mortality, thousands (95% UI)	Age-standardised Mortality rate (95% UI)
Breast cancer	2 470 (2 240 - 2 700)	164.1 (148.7 - 179.5)	169 (153 - 185)	11.2 (10.2 - 12.3)	42.7 (38.8 - 47.0)	2.8 (2.6 - 3.1)
Cervical cancer	1 560 (1 320 - 1 780)	103.6 (87.7 - 118.3)	119 (99.6 - 135)	7.9 (6.6 - 9.0)	27.2 (22.9 - 31.1)	1.8 (1.5 - 2.1)
Chronic lymphoid leukaemia	26.4 (21.0 - 32.8)	1.8 (1.4 - 2.2)	1.99 (1.52 - 2.53)	0.1 (0.1 - 0.2)	0.439 (0.346 - 0.548)	0.0 (0.0 - 0.0)
Chronic myeloid leukaemia	121 (99.3 - 144)	8.2 (6.7 - 9.7)	3.97 (3.43 - 4.56)	0.3 (0.2 - 0.3)	2.02 (1.67 - 2.40)	0.1 (0.1 - 0.2)
Colon and rectum cancer	660 (600 - 723)	44.0 (40.0 - 48.2)	29.3 (26.2 - 32.5)	1.9 (1.7 - 2.2)	11.5 (10.4 - 12.6)	0.8 (0.7 - 0.8)
Gallbladder and biliary tract cancer	71.2 (54.7 - 82.8)	4.7 (3.6 - 5.5)	1.97 (1.56 - 2.28)	0.1 (0.1 - 0.2)	1.28 (0.987 - 1.49)	0.1 (0.1 - 0.1)
Hodgkin lymphoma	206 (160 - 252)	14.0 (10.9 - 17.2)	15.3 (12.9 - 20.3)	1.0 (0.9 - 1.4)	3.23 (2.49 - 3.93)	0.2 (0.2 - 0.3)
Kidney cancer	86.6 (78.5 - 94.8)	5.8 (5.3 - 6.4)	8.47 (7.58 - 9.47)	0.6 (0.5 - 0.6)	1.42 (1.29 - 1.57)	0.1 (0.1 - 0.1)
Larynx cancer	37.3 (33.0 - 42.2)	2.5 (2.2 - 2.8)	1.16 (1.03 - 1.30)	0.1 (0.1 - 0.1)	0.630 (0.557 - 0.715)	0.0 (0.0 - 0.0)
Lip and oral cavity cancer	213 (185 - 244)	14.3 (12.4 - 16.4)	12.3 (10.7 - 14.0)	0.8 (0.7 - 0.9)	3.61 (3.13 - 4.15)	0.2 (0.2 - 0.3)
Liver cancer	252 (218 - 290)	16.9 (14.6 - 19.5)	6.08 (5.33 - 6.91)	0.4 (0.4 - 0.5)	4.38 (3.79 - 5.06)	0.3 (0.3 - 0.3)
Malignant skin melanoma	123 (96.5 - 153)	8.2 (6.5 - 10.2)	21.3 (16.1 - 28.2)	1.4 (1.1 - 1.9)	1.97 (1.57 - 2.45)	0.1 (0.1 - 0.2)

	DALYs, thousands (95% UI)	Age-standardised DALYs rate (95% UI)	Incidence, thousands (95% UI)	Age-standardised Incidence rate (95% UI)	Mortality, thousands (95% UI)	Age-standardised Mortality rate (95% UI)
Mesothelioma	26.6 (16.3 - 37.0)	1.8 (1.1 - 2.5)	0.710 (0.429 - 0.994)	0.0 (0.0 - 0.1)	0.466 (0.287 - 0.655)	0.0 (0.0 - 0.0)
Multiple myeloma	35.6 (25.7 - 41.7)	2.4 (1.7 - 2.8)	1.10 (0.793 - 1.29)	0.1 (0.1 - 0.1)	0.627 (0.454 - 0.734)	0.0 (0.0 - 0.0)
Nasopharynx cancer	114 (101 - 127)	7.6 (6.8 - 8.6)	8.36 (7.04 - 9.96)	0.6 (0.5 - 0.7)	1.90 (1.68 - 2.13)	0.1 (0.1 - 0.1)
Non-Hodgkin lymphoma	467 (423 - 511)	31.7 (28.7 - 34.7)	19.2 (16.9 - 21.8)	1.3 (1.2 - 1.5)	7.61 (6.90 - 8.33)	0.5 (0.5 - 0.6)
Oesophageal cancer	116 (98.6 - 134)	7.7 (6.6 - 8.9)	2.83 (2.41 - 3.28)	0.2 (0.2 - 0.2)	2.06 (1.76 - 2.38)	0.1 (0.1 - 0.2)
Other leukaemia	396 (304 - 484)	27.0 (20.7 - 33.0)	12.0 (9.55 - 14.8)	0.8 (0.6 - 1.0)	6.39 (4.94 - 7.78)	0.4 (0.3 - 0.5)
Other malignant neoplasms	1 350 (1 210 - 1 510)	92.1 (82.7 - 103.1)	63.8 (57.6 - 71.3)	4.4 (3.9 - 4.9)	21.4 (19.3 - 24.0)	1.5 (1.3 - 1.6)
Other pharynx cancer	87.9 (70.3 - 107)	5.9 (4.7 - 7.2)	2.62 (2.17 - 3.10)	0.2 (0.1 - 0.2)	1.54 (1.23 - 1.88)	0.1 (0.1 - 0.1)
Ovarian cancer	529 (443 - 602)	35.5 (29.7 - 40.5)	35.8 (30.5 - 41.0)	2.4 (2.1 - 2.8)	8.90 (7.46 - 10.1)	0.6 (0.5 - 0.7)
Pancreatic cancer	144 (130 - 159)	9.6 (8.7 - 10.6)	3.28 (2.93 - 3.61)	0.2 (0.2 - 0.2)	2.59 (2.34 - 2.85)	0.2 (0.2 - 0.2)
Stomach cancer	732 (653 - 814)	48.8 (43.6 - 54.3)	21.1 (18.8 - 23.5)	1.4 (1.3 - 1.6)	12.9 (11.6 - 14.3)	0.9 (0.8 - 1.0)
Thyroid cancer	122 (101 - 142)	8.2 (6.8 - 9.6)	34.5 (28.8 - 39.1)	2.3 (1.9 - 2.6)	1.77 (1.46 - 2.07)	0.1 (0.1 - 0.1)

	DALYs, thousands (95% UI)	Age-standardised DALYs rate (95% UI)	Incidence, thousands (95% UI)	Age-standardised Incidence rate (95% UI)	Mortality, thousands (95% UI)	Age-standardised Mortality rate (95% UI)
Tracheal, bronchus, and lung cancer	530 (475 - 592)	35.3 (31.6 - 39.4)	12.8 (11.3 - 14.3)	0.9 (0.8 - 1.0)	9.46 (8.46 - 10.6)	0.6 (0.6 - 0.7)
Uterine cancer	110 (85.1 - 124)	7.3 (5.6 - 8.2)	19.4 (15.8 - 22.0)	1.3 (1.0 - 1.5)	1.81 (1.39 - 2.04)	0.1 (0.1 - 0.1)

Estimates are for 15–39-year-olds, females. Rates are reported per 100,000 person-years. Age-standardisation used the GBD 2019 world standard. SDI categories do not sum precisely to the global total as the GBD study does not provide separate estimates for all locations globally and an adjustment factor is made between all estimated locations which have corresponding SDI values and the global estimate. Total AYA Cancers=all malignant neoplasms in this age group excluding non-melanoma skin cancers. DALYs=disability-adjusted life-years. SDI=Socio-demographic Index. GBD=Global Burden of Diseases, Injuries, and Risk Factors Study. UI=uncertainty interval. Other malignant neoplasms are cancers without a detailed GBD cause separately listed.

Appendix Table 11: Adolescent and young adult cancer YLD and YLL burden globally and by SDI quintile in 2019, males.

	Age-standardised YLL rate (95% UI)	YLLs, thousands (95% UI)	Age-standardised YLD rate (95% UI)	YLDs, thousands (95% UI)
**Global, Total AYA Cancers**	743.8 (686.0 - 801.2)	11 300 (10 400 - 12 100)	16.5 (11.5 - 22.1)	250 (174 - 334)
**SDI Quintiles, Total AYA Cancers**				
High SDI Countries	519.9 (497.0 - 546.4)	951 (907 - 1 000)	28.9 (20.0 - 39.4)	52.3 (36.1 - 71.1)
High-middle SDI Countries	828.5 (756.6 - 905.4)	2 390 (2 180 - 2 610)	26.3 (18.2 - 35.6)	75.3 (52.2 - 102)
Middle SDI Countries	818.9 (742.5 - 898.9)	3 950 (3 580 - 4 330)	15.8 (11.2 - 21.3)	76.3 (53.8 - 102)
Low-middle SDI Countries	733.8 (660.4 - 817.7)	2 640 (2 370 - 2 940)	8.8 (6.3 - 11.7)	31.8 (22.7 - 42.4)
Low SDI Countries	642.7 (551.3 - 730.1)	1 340 (1 150 - 1 520)	6.7 (4.6 - 9.0)	14.1 (9.77 - 19.1)
**Global, per Cancer**				
Acute lymphoid leukaemia	31.3 (24.7 - 35.3)	466 (368 - 525)	0.9 (0.6 - 1.2)	13.3 (8.83 - 18.7)
Acute myeloid leukaemia	26.5 (22.2 - 33.0)	398 (333 - 496)	0.2 (0.1 - 0.3)	3.14 (2.14 - 4.33)
Bladder cancer	5.2 (4.6 - 5.9)	79.2 (70.1 - 89.7)	0.5 (0.3 - 0.7)	7.23 (4.86 - 9.93)
Brain and central nervous system cancer	66.8 (49.3 - 77.4)	1 010 (745 - 1 170)	0.9 (0.6 - 1.3)	14.1 (8.80 - 19.5)
Breast cancer	1.3 (1.2 - 1.5)	20.0 (17.8 - 22.4)	0.1 (0.0 - 0.1)	0.884 (0.611 - 1.21)
Chronic lymphoid leukaemia	2.2 (1.7 - 2.6)	33.3 (25.5 - 39.8)	0.1 (0.1 - 0.1)	1.61 (1.04 - 2.25)
Chronic myeloid leukaemia	11.4 (9.4 - 13.7)	172 (143 - 208)	0.1 (0.1 - 0.2)	1.81 (1.26 - 2.48)
Colon and rectum cancer	61.9 (56.3 - 68.2)	945 (860 - 1 040)	1.9 (1.3 - 2.5)	28.3 (19.4 - 38.2)
Gallbladder and biliary tract cancer	4.0 (3.3 - 4.5)	60.8 (51.2 - 68.7)	0.0 (0.0 - 0.0)	0.495 (0.339 - 0.674)
Hodgkin lymphoma	19.5 (15.5 - 24.7)	293 (234 - 372)	0.6 (0.4 - 0.8)	8.96 (6.15 - 12.3)

	Age-standardised YLL rate (95% UI)	YLLs, thousands (95% UI)	Age-standardised YLD rate (95% UI)	YLDs, thousands (95% UI)
Kidney cancer	9.6 (8.7 - 10.8)	146 (132 - 164)	0.4 (0.3 - 0.6)	6.26 (4.24 - 8.59)
Larynx cancer	5.8 (5.2 - 6.4)	88.0 (79.5 - 98.1)	0.2 (0.1 - 0.3)	2.81 (1.90 - 3.98)
Lip and oral cavity cancer	23.6 (20.0 - 27.3)	360 (305 - 417)	0.5 (0.3 - 0.7)	7.35 (5.09 - 10.1)
Liver cancer	51.7 (44.8 - 58.9)	790 (685 - 900)	0.3 (0.2 - 0.4)	4.98 (3.49 - 6.82)
Malignant skin melanoma	8.4 (6.3 - 11.1)	129 (95.9 - 170)	0.5 (0.3 - 0.8)	7.89 (4.65 - 12.0)
Mesothelioma	1.9 (1.7 - 2.2)	29.5 (25.9 - 34.2)	0.0 (0.0 - 0.0)	0.257 (0.179 - 0.339)
Multiple myeloma	3.9 (2.9 - 4.5)	58.9 (44.5 - 68.1)	0.1 (0.0 - 0.1)	1.01 (0.673 - 1.39)
Nasopharynx cancer	15.6 (14.2 - 17.2)	238 (215 - 262)	0.7 (0.5 - 1.0)	11.5 (7.63 - 15.8)
Non-Hodgkin lymphoma	52.5 (48.4 - 57.7)	791 (729 - 869)	1.2 (0.9 - 1.6)	18.4 (12.9 - 24.5)
Oesophageal cancer	14.8 (13.0 - 16.9)	226 (199 - 259)	0.1 (0.1 - 0.2)	1.70 (1.19 - 2.31)
Other leukaemia	36.1 (28.5 - 41.9)	542 (428 - 630)	0.7 (0.5 - 1.0)	10.4 (6.92 - 14.5)
Other malignant neoplasms	123.1 (106.8 - 138.0)	1 850 (1 600 - 2 070)	2.7 (1.8 - 3.6)	39.9 (27.7 - 54.2)
Other pharynx cancer	10.2 (8.4 - 12.1)	156 (128 - 184)	0.1 (0.1 - 0.1)	1.28 (0.897 - 1.71)
Pancreatic cancer	18.0 (16.3 - 20.2)	275 (249 - 309)	0.1 (0.1 - 0.1)	1.40 (0.992 - 1.85)
Prostate cancer	3.3 (2.8 - 4.0)	50.2 (43.4 - 61.1)	0.3 (0.2 - 0.4)	4.15 (2.80 - 5.86)
Stomach cancer	54.4 (49.6 - 60.0)	832 (758 - 918)	0.7 (0.5 - 0.9)	10.1 (7.13 - 13.6)
Testicular cancer	21.2 (19.5 - 23.1)	320 (294 - 350)	1.9 (1.3 - 2.7)	28.6 (19.3 - 40.2)
Thyroid cancer	4.1 (3.7 - 4.6)	62.8 (56.4 - 69.4)	0.4 (0.3 - 0.6)	6.33 (4.14 - 9.06)

	Age-standardised YLL rate (95% UI)	YLLs, thousands (95% UI)	Age-standardised YLD rate (95% UI)	YLDs, thousands (95% UI)
Tracheal, bronchus, and lung cancer	55.7 (49.8 - 62.0)	850 (760 - 946)	0.4 (0.3 - 0.5)	5.55 (3.92 - 7.33)

Estimates are for 15–39-year-old males globally. Rates are reported per 100,000 person-years. Age-standardisation used the GBD 2019 world standard. SDI categories do not sum precisely to the global total as the GBD study does not provide separate estimates for all locations globally and an adjustment factor is made between all estimated locations which have corresponding SDI values and the global estimate. Total AYA Cancers=all malignant neoplasms in this age group excluding non-melanoma skin cancers. YLDs=years lived with disability. YLLs=years of life lost. SDI=Socio-demographic Index. GBD=Global Burden of Diseases, Injuries, and Risk Factors Study. UI=uncertainty interval. Other malignant neoplasms are cancers without a detailed GBD cause separately listed.

Appendix Table 12: Adolescent and young adult cancer YLD and YLL burden globally and by SDI quintile in 2019, females.

	Age-standardised YLL rate (95% UI)	YLLs, thousands (95% UI)	Age-standardised YLD rate (95% UI)	YLDs, thousands (95% UI)
**Global, Total AYA Cancers**	778.9 (706.8 - 852.4)	11 600 (10 500 - 12 700)	25.0 (17.5 - 33.6)	374 (262 - 503)
**SDI Quintiles, Total AYA Cancers**				
High SDI Countries	541.1 (520.7 - 562.9)	945 (910 - 984)	39.8 (27.4 - 54.5)	70.0 (48.2 - 95.9)
High-middle SDI Countries	712.0 (643.0 - 790.2)	1 960 (1 770 - 2 180)	32.0 (22.0 - 44.1)	89.5 (61.7 - 123)
Middle SDI Countries	760.3 (685.4 - 841.2)	3 640 (3 280 - 4 040)	24.0 (16.7 - 32.4)	116 (80.9 - 156)
Low-middle SDI Countries	911.6 (797.1 - 1028.4)	3 230 (2 830 - 3 650)	18.9 (13.1 - 25.5)	66.7 (46.2 - 90.1)
Low SDI Countries	891.0 (757.1 - 1040.9)	1 810 (1 540 - 2 110)	16.0 (10.9 - 22.3)	32.0 (21.7 - 44.8)
**Global, per Cancer**				
Acute lymphoid leukaemia	19.1 (14.1 - 22.5)	276 (203 - 325)	0.7 (0.5 - 1.0)	10.6 (6.92 - 15.2)
Acute myeloid leukaemia	23.5 (20.2 - 26.9)	344 (296 - 394)	0.2 (0.2 - 0.3)	3.20 (2.21 - 4.30)
Bladder cancer	2.4 (2.1 - 2.7)	35.8 (31.9 - 39.7)	0.2 (0.1 - 0.2)	2.33 (1.59 - 3.24)
Brain and central nervous system cancer	48.0 (35.7 - 55.0)	709 (526 - 812)	0.9 (0.6 - 1.2)	12.8 (8.33 - 17.9)
Breast cancer	156.2 (141.5 - 171.5)	2 350 (2 130 - 2 580)	7.9 (5.5 - 10.8)	119 (82.3 - 162)
Cervical cancer	99.6 (83.9 - 113.7)	1 500 (1 260 - 1 710)	4.0 (2.7 - 5.6)	60.5 (41.2 - 83.8)
Chronic lymphoid leukaemia	1.7 (1.3 - 2.1)	25.4 (20.1 - 31.5)	0.1 (0.0 - 0.1)	1.07 (0.675 - 1.58)
Chronic myeloid leukaemia	8.1 (6.6 - 9.6)	120 (97.9 - 142)	0.1 (0.1 - 0.1)	1.42 (0.990 - 1.93)
Colon and rectum cancer	42.8 (39.0 - 47.0)	643 (585 - 705)	1.1 (0.8 - 1.6)	17.2 (12.0 - 23.5)
Gallbladder and biliary tract cancer	4.7 (3.6 - 5.5)	70.7 (54.4 - 82.3)	0.0 (0.0 - 0.0)	0.526 (0.349 - 0.712)

	Age-standardised YLL rate (95% UI)	YLLs, thousands (95% UI)	Age-standardised YLD rate (95% UI)	YLDs, thousands (95% UI)
Hodgkin lymphoma	13.5<br>(10.5 - 16.6)	198<br>(153 - 242)	0.5<br>(0.3 - 0.8)	7.69<br>(5.04 - 11.4)
Kidney cancer	5.5<br>(5.0 - 6.1)	82.3<br>(74.6 - 90.5)	0.3<br>(0.2 - 0.4)	4.27<br>(2.87 - 5.81)
Larynx cancer	2.4<br>(2.1 - 2.7)	35.4<br>(31.3 - 40.2)	0.1<br>(0.1 - 0.2)	1.91<br>(1.18 - 2.86)
Lip and oral cavity cancer	13.9<br>(12.1 - 16.0)	207<br>(180 - 239)	0.4<br>(0.3 - 0.5)	5.37<br>(3.74 - 7.35)
Liver cancer	16.8<br>(14.5 - 19.3)	251<br>(216 - 289)	0.1<br>(0.1 - 0.1)	1.58<br>(1.11 - 2.14)
Malignant skin melanoma	7.5<br>(6.0 - 9.3)	112<br>(90.1 - 140)	0.7<br>(0.4 - 1.1)	10.4<br>(6.33 - 15.9)
Mesothelioma	1.8<br>(1.1 - 2.5)	26.4<br>(16.2 - 36.7)	0.0<br>(0.0 - 0.0)	0.223<br>(0.126 - 0.342)
Multiple myeloma	2.3<br>(1.7 - 2.7)	35.0<br>(25.3 - 41.1)	0.0<br>(0.0 - 0.1)	0.601<br>(0.389 - 0.845)
Nasopharynx cancer	7.4<br>(6.5 - 8.3)	109<br>(96.6 - 123)	0.3<br>(0.2 - 0.4)	4.36<br>(2.96 - 6.04)
Non-Hodgkin lymphoma	31.0<br>(28.1 - 34.0)	457<br>(414 - 500)	0.7<br>(0.5 - 0.9)	10.0<br>(7.00 - 13.5)
Oesophageal cancer	7.6<br>(6.5 - 8.9)	115<br>(97.7 - 133)	0.1<br>(0.0 - 0.1)	0.974<br>(0.678 - 1.33)
Other leukaemia	26.5<br>(20.2 - 32.3)	388<br>(298 - 474)	0.5<br>(0.4 - 0.8)	7.96<br>(5.28 - 11.2)
Other malignant neoplasms	89.9<br>(80.6 - 100.7)	1 310<br>(1 180 - 1 470)	2.1<br>(1.5 - 2.9)	31.1<br>(21.7 - 42.4)
Other pharynx cancer	5.8<br>(4.6 - 7.1)	87.1<br>(69.6 - 107)	0.1<br>(0.0 - 0.1)	0.753<br>(0.512 - 1.02)
Ovarian cancer	34.2<br>(28.5 - 39.0)	509<br>(426 - 581)	1.3<br>(0.9 - 1.8)	19.6<br>(13.6 - 26.4)
Pancreatic cancer	9.6<br>(8.6 - 10.5)	143<br>(130 - 158)	0.1<br>(0.0 - 0.1)	0.766<br>(0.542 - 1.01)
Stomach cancer	48.3<br>(43.2 - 53.7)	725<br>(648 - 806)	0.5<br>(0.3 - 0.6)	6.91<br>(4.93 - 9.27)
Thyroid cancer	7.0<br>(5.8 - 8.3)	104<br>(86.1 - 122)	1.2<br>(0.7 - 1.7)	17.4<br>(11.2 - 25.5)

	**Age-standardised YLL rate (95% UI)**	**YLLs, thousands (95% UI)**	**Age-standardised YLD rate (95% UI)**	**YLDs, thousands (95% UI)**
Tracheal, bronchus, and lung cancer	35.1 (31.4 - 39.2)	527 (471 - 588)	0.2 (0.2 - 0.3)	3.68 (2.58 - 4.91)
Uterine cancer	6.6 (5.1 - 7.5)	99.7 (76.3 - 113)	0.7 (0.5 - 1.0)	10.5 (6.86 - 14.5)

Estimates are for 15–39-year-old females globally. Rates are reported per 100,000 person-years. Age-standardisation used the GBD 2019 world standard. SDI categories do not sum precisely to the global total as the GBD study does not provide separate estimates for all locations globally and an adjustment factor is made between all estimated locations which have corresponding SDI values and the global estimate. Total AYA Cancers=all malignant neoplasms in this age group excluding non-melanoma skin cancers. YLDs=years lived with disability. YLLs=years of life lost. SDI=Socio-demographic Index. GBD=Global Burden of Diseases, Injuries, and Risk Factors Study. UI=uncertainty interval. Other malignant neoplasms are cancers without a detailed GBD cause separately listed.

Appendix Table 13: Adolescent and young adult cancer YLD and YLL burden globally and by SDI quintile in 2019, both sexes combined.

	Age-standardised YLL rate (95% UI)	YLLs, thousands (95% UI)	Age-standardised YLD rate (95% UI)	YLDs, thousands (95% UI)
**Global, Total AYA Cancers**	761.5 (710.7 - 816.6)	22 900 (21 300 - 24 500)	20.7 (14.6 - 27.6)	624 (440 - 831)
**SDI Quintiles, Total AYA Cancers**				
High SDI Countries	530.1 (511.4 - 550.8)	1 900 (1 830 - 1 970)	34.2 (23.6 - 45.9)	122 (84.3 - 164)
High-middle SDI Countries	772.3 (718.6 - 826.4)	4 350 (4 040 - 4 660)	29.1 (20.2 - 39.4)	165 (115 - 222)
Middle SDI Countries	790.2 (729.9 - 854.5)	7 590 (7 010 - 8 220)	19.9 (14.0 - 26.7)	192 (135 - 257)
Low-middle SDI Countries	822.9 (747.7 - 903.2)	5 870 (5 330 - 6 440)	13.8 (9.8 - 18.5)	98.4 (69.9 - 132)
Low SDI Countries	769.7 (669.2 - 877.6)	3 150 (2 730 - 3 580)	11.4 (7.9 - 15.6)	46.1 (31.9 - 63.1)
**Global, per Cancer**				
Acute lymphoid leukaemia	25.3 (21.0 - 28.0)	742 (615 - 820)	0.8 (0.6 - 1.1)	23.9 (16.4 - 33.0)
Acute myeloid leukaemia	25.0 (22.6 - 28.6)	741 (671 - 850)	0.2 (0.2 - 0.3)	6.33 (4.47 - 8.53)
Bladder cancer	3.8 (3.4 - 4.2)	115 (104 - 128)	0.3 (0.2 - 0.4)	9.55 (6.49 - 13.2)
Brain and central nervous system cancer	57.5 (45.6 - 63.9)	1 720 (1 360 - 1 910)	0.9 (0.6 - 1.2)	27.0 (18.0 - 36.8)
Breast cancer	78.1 (70.8 - 85.7)	2 370 (2 150 - 2 600)	3.9 (2.7 - 5.4)	119 (82.9 - 163)
Cervical cancer	49.4 (41.6 - 56.4)	1 500 (1 260 - 1 710)	2.0 (1.4 - 2.8)	60.5 (41.2 - 83.8)
Chronic lymphoid leukaemia	1.9 (1.7 - 2.2)	58.7 (50.6 - 66.5)	0.1 (0.1 - 0.1)	2.68 (1.86 - 3.72)
Chronic myeloid leukaemia	9.7 (8.6 - 11.1)	292 (258 - 331)	0.1 (0.1 - 0.1)	3.23 (2.29 - 4.31)
Colon and rectum cancer	52.4 (48.5 - 56.5)	1 590 (1 470 - 1 710)	1.5 (1.1 - 2.0)	45.5 (31.9 - 61.8)
Gallbladder and biliary tract cancer	4.3 (3.7 - 4.8)	132 (112 - 146)	0.0 (0.0 - 0.0)	1.02 (0.707 - 1.37)
Hodgkin lymphoma	16.5 (14.0 - 19.5)	492 (416 - 580)	0.6 (0.4 - 0.8)	16.6 (11.3 - 23.1)
Kidney cancer	7.6 (7.0 - 8.4)	229 (210 - 252)	0.3 (0.2 - 0.5)	10.5 (7.12 - 14.3)

	Age-standardised YLL rate (95% UI)	YLLs, thousands (95% UI)	Age-standardised YLD rate (95% UI)	YLDs, thousands (95% UI)
Larynx cancer	4.1 (3.7 - 4.5)	123 (113 - 135)	0.2 (0.1 - 0.2)	4.73 (3.13 - 6.82)
Lip and oral cavity cancer	18.8 (16.8 - 20.8)	568 (508 - 628)	0.4 (0.3 - 0.6)	12.7 (8.94 - 17.3)
Liver cancer	34.4 (30.8 - 38.2)	1 040 (932 - 1 160)	0.2 (0.2 - 0.3)	6.56 (4.65 - 8.90)
Malignant skin melanoma	8.0 (6.7 - 9.8)	241 (202 - 296)	0.6 (0.4 - 0.9)	18.3 (11.7 - 27.3)
Mesothelioma	1.8 (1.4 - 2.2)	55.9 (43.7 - 67.4)	0.0 (0.0 - 0.0)	0.479 (0.325 - 0.661)
Multiple myeloma	3.1 (2.4 - 3.5)	94.0 (73.0 - 105)	0.1 (0.0 - 0.1)	1.61 (1.08 - 2.22)
Nasopharynx cancer	11.5 (10.6 - 12.6)	347 (320 - 380)	0.5 (0.4 - 0.7)	15.8 (11.0 - 21.4)
Non-Hodgkin lymphoma	41.9 (38.9 - 45.4)	1 250 (1 160 - 1 350)	1.0 (0.7 - 1.3)	28.4 (20.0 - 38.0)
Oesophageal cancer	11.2 (10.1 - 12.5)	341 (305 - 379)	0.1 (0.1 - 0.1)	2.67 (1.90 - 3.64)
Other leukaemia	31.3 (26.0 - 35.7)	930 (773 - 1 060)	0.6 (0.4 - 0.8)	18.3 (12.7 - 25.3)
Other malignant neoplasms	106.7 (96.5 - 116.9)	3 160 (2 860 - 3 460)	2.4 (1.7 - 3.3)	71.0 (50.1 - 96.5)
Other pharynx cancer	8.0 (6.9 - 9.1)	243 (209 - 274)	0.1 (0.0 - 0.1)	2.04 (1.42 - 2.70)
Ovarian cancer	16.9 (14.1 - 19.3)	509 (426 - 581)	0.7 (0.5 - 0.9)	19.6 (13.6 - 26.4)
Pancreatic cancer	13.8 (12.7 - 15.2)	419 (385 - 461)	0.1 (0.1 - 0.1)	2.16 (1.53 - 2.87)
Prostate cancer	1.7 (1.4 - 2.0)	50.2 (43.4 - 61.1)	0.1 (0.1 - 0.2)	4.15 (2.80 - 5.86)
Stomach cancer	51.4 (47.3 - 55.7)	1 560 (1 430 - 1 690)	0.6 (0.4 - 0.8)	17.0 (12.2 - 22.8)
Testicular cancer	10.7 (9.8 - 11.7)	320 (294 - 350)	1.0 (0.6 - 1.3)	28.6 (19.3 - 40.2)
Thyroid cancer	5.6 (4.9 - 6.2)	167 (148 - 187)	0.8 (0.5 - 1.1)	23.7 (15.6 - 33.9)
Tracheal, bronchus, and lung cancer	45.5 (41.7 - 49.7)	1 380 (1 260 - 1 500)	0.3 (0.2 - 0.4)	9.24 (6.60 - 12.3)
Uterine cancer	3.3 (2.5 - 3.7)	99.7 (76.3 - 113)	0.3 (0.2 - 0.5)	10.5 (6.86 - 14.5)

Estimates are for 15–39-year-olds, both sexes combined. Rates are reported per 100,000 person-years. Age-standardisation used the GBD 2019 world standard. SDI categories do not sum precisely to the global total as the GBD study does not provide separate estimates for all locations globally and an adjustment factor is made between all estimated locations which have corresponding SDI values and the global estimate. Total AYA Cancers=all malignant neoplasms in this age group excluding non-melanoma skin cancers. YLDs=years lived with disability. YLLs=years of life lost. SDI=Socio-demographic Index. GBD=Global Burden of Diseases, Injuries, and Risk Factors Study. UI=uncertainty interval. Other malignant neoplasms are cancers without a detailed GBD cause separately listed.

Appendix Table 14: Percentage of adolescent and young adult cancer DALY burden made up by YLDs and YLLs globally and by SDI quintile in 2019.

Location & Cause	% YLLs (95% UI)	% YLDs (95% UI)
Global Countries, Total AYA Cancers	97.3 (96.4 to 98.1)	2.7 (1.9 to 3.6)
High SDI Countries, Total AYA Cancers	94.0 (92.0 to 95.7)	6.0 (4.3 to 8.0)
High-middle SDI Countries, Total AYA Cancers	96.4 (95.1 to 97.4)	3.6 (2.6 to 4.9)
Middle SDI Countries, Total AYA Cancers	97.5 (96.7 to 98.3)	2.5 (1.7 to 3.3)
Low-middle SDI Countries, Total AYA Cancers	98.4 (97.8 to 98.8)	1.6 (1.2 to 2.2)
Low SDI Countries, Total AYA Cancers	98.6 (98.1 to 99.0)	1.4 (1.0 to 1.9)
Global Acute lymphoid leukaemia	96.9 (95.8 to 97.8)	3.1 (2.2 to 4.2)
Global Acute myeloid leukaemia	99.2 (98.9 to 99.4)	0.8 (0.6 to 1.1)
Global Bladder cancer	92.3 (89.6 to 94.6)	7.7 (5.4 to 10.4)
Global Brain and central nervous system cancer	98.5 (98.0 to 98.9)	1.5 (1.1 to 2.0)
Global Breast cancer	95.2 (93.5 to 96.6)	4.8 (3.4 to 6.5)
Global Cervical cancer	96.1 (94.8 to 97.3)	3.9 (2.7 to 5.2)
Global Chronic lymphoid leukaemia	95.6 (94.0 to 96.9)	4.4 (3.1 to 6.0)
Global Chronic myeloid leukaemia	98.9 (98.6 to 99.2)	1.1 (0.8 to 1.4)
Global Colon and rectum cancer	97.2 (96.3 to 98.0)	2.8 (2.0 to 3.7)
Global Gallbladder and biliary tract cancer	99.2 (99.0 to 99.5)	0.8 (0.5 to 1.0)
Global Hodgkin lymphoma	96.7 (95.5 to 97.7)	3.3 (2.3 to 4.5)
Global Kidney cancer	95.6 (94.0 to 97.0)	4.4 (3.0 to 6.0)
Global Larynx cancer	96.3 (94.7 to 97.6)	3.7 (2.4 to 5.3)
Global Lip and oral cavity cancer	97.8 (97.1 to 98.4)	2.2 (1.6 to 2.9)
Global Liver cancer	99.4 (99.2 to 99.6)	0.6 (0.4 to 0.8)
Global Malignant skin melanoma	93.0 (90.2 to 95.3)	7.0 (4.7 to 9.8)
Global Mesothelioma	99.1 (98.9 to 99.4)	0.9 (0.6 to 1.1)
Global Multiple myeloma	98.3 (97.8 to 98.8)	1.7 (1.2 to 2.2)
Global Nasopharynx cancer	95.6 (94.2 to 96.9)	4.4 (3.1 to 5.8)
Global non-Hodgkin lymphoma	97.8 (97.0 to 98.4)	2.2 (1.6 to 3.0)
Global Oesophageal cancer	99.2 (99.0 to 99.4)	0.8 (0.6 to 1.0)
Global Other leukaemia	98.1 (97.4 to 98.6)	1.9 (1.4 to 2.6)
Global Other malignant neoplasms	97.8 (97.1 to 98.4)	2.2 (1.6 to 2.9)
Global Other pharynx cancer	99.2 (98.9 to 99.4)	0.8 (0.6 to 1.1)
Global Ovarian cancer	96.3 (95.0 to 97.3)	3.7 (2.7 to 5.0)
Global Pancreatic cancer	99.5 (99.3 to 99.6)	0.5 (0.4 to 0.7)
Global Prostate cancer	92.4 (89.7 to 94.7)	7.6 (5.3 to 10.3)
Global Stomach cancer	98.9 (98.6 to 99.2)	1.1 (0.8 to 1.4)
Global Testicular cancer	91.8 (88.9 to 94.4)	8.2 (5.6 to 11.1)
Global Thyroid cancer	87.6 (83.1 to 91.3)	12.4 (8.7 to 16.9)
Global Tracheal, bronchus, and lung cancer	99.3 (99.1 to 99.5)	0.7 (0.5 to 0.9)
Global Uterine cancer	90.5 (87.1 to 93.4)	9.5 (6.6 to 12.9)

Estimates are for 15–39-year-olds, both sexes combined. Total AYA Cancers=all malignant neoplasms in this age group excluding non-melanoma skin cancers. DALYs=disability-adjusted life-years. YLDs=years lived with disability. YLLs=years of life lost. SDI=Socio-demographic Index. UI=uncertainty interval. Other malignant neoplasms are cancers without a detailed GBD cause separately listed.

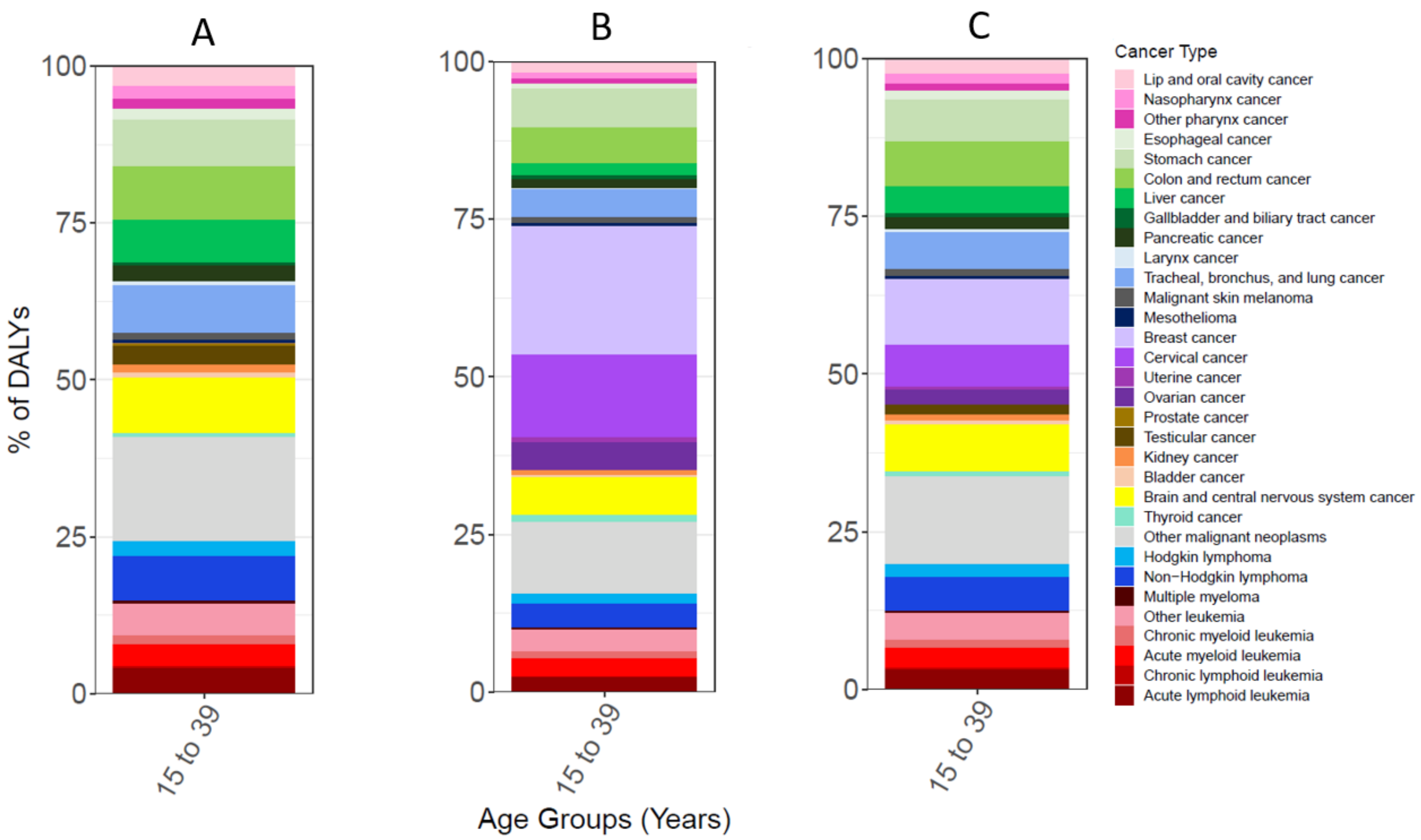

Appendix Figure 4: Percentage of adolescent and young adult (15-39 years) total DALY cancer burden attributable to cancer types, global, 2019, in (A) males, (B) females, and (C) both sexes combined. Other malignant neoplasms: all malignancies without a separate GBD cause category listed; this category does not include non-melanoma skin cancers and myelodysplastic/myeloproliferative neoplasms, which are separate GBD cause categories not included in this analysis. DALYs=disability-adjusted life-years.

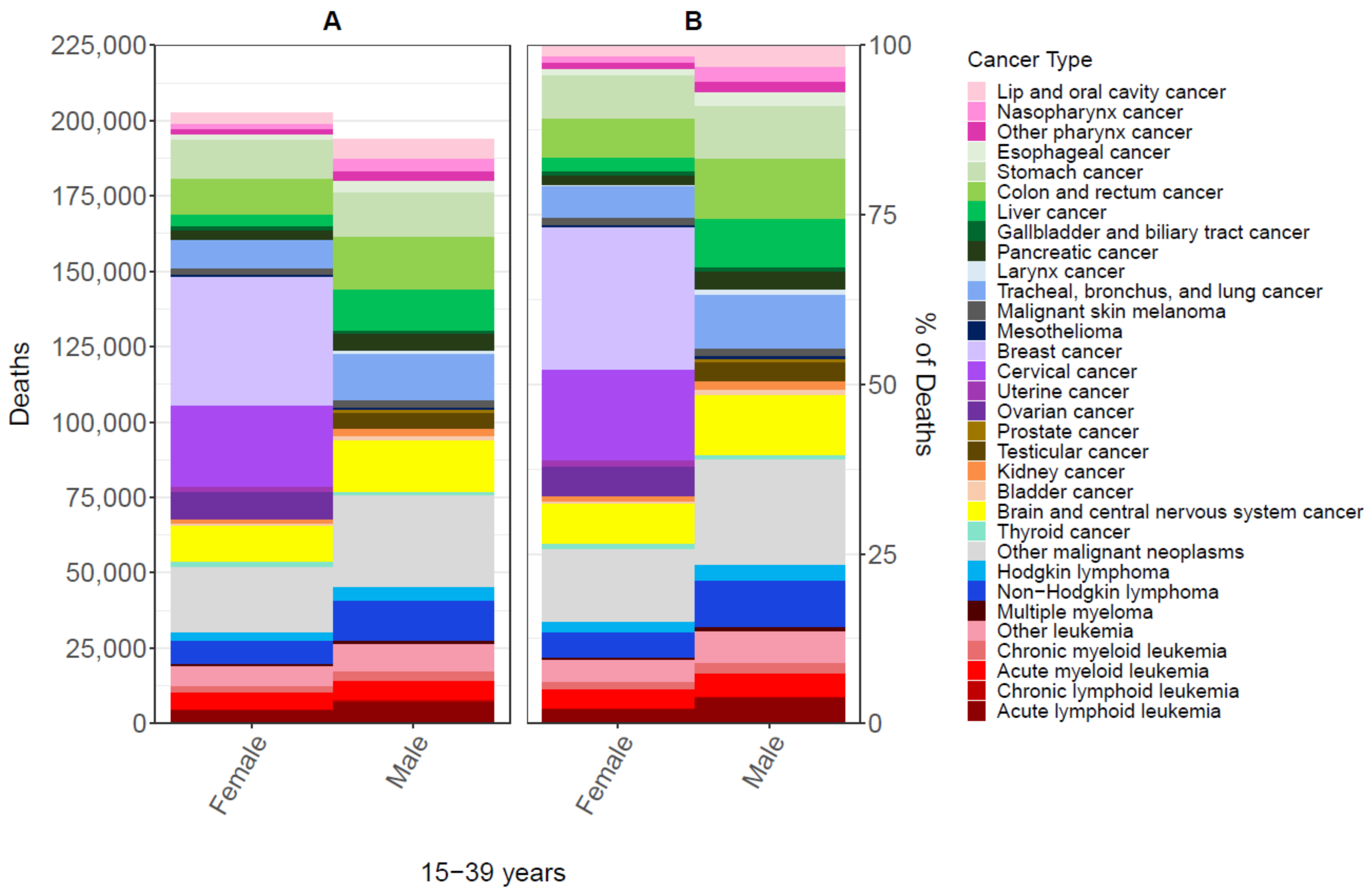

Appendix Figure 5: (A) Absolute and (B) proportional adolescent and young adult (15-39) cancer mortality burden, globally, by sex in 2019. Other malignant neoplasms: all malignancies without a separate GBD cause category listed; this category does not include non-melanoma skin cancers and myelodysplastic/myeloproliferative neoplasms, which are separate GBD cause categories not included in this analysis. Other leukaemia included leukaemias not otherwise specified. DALYs=disability-adjusted life-years. GBD=Global Burden of Diseases, Injuries, and Risk Factors Study.

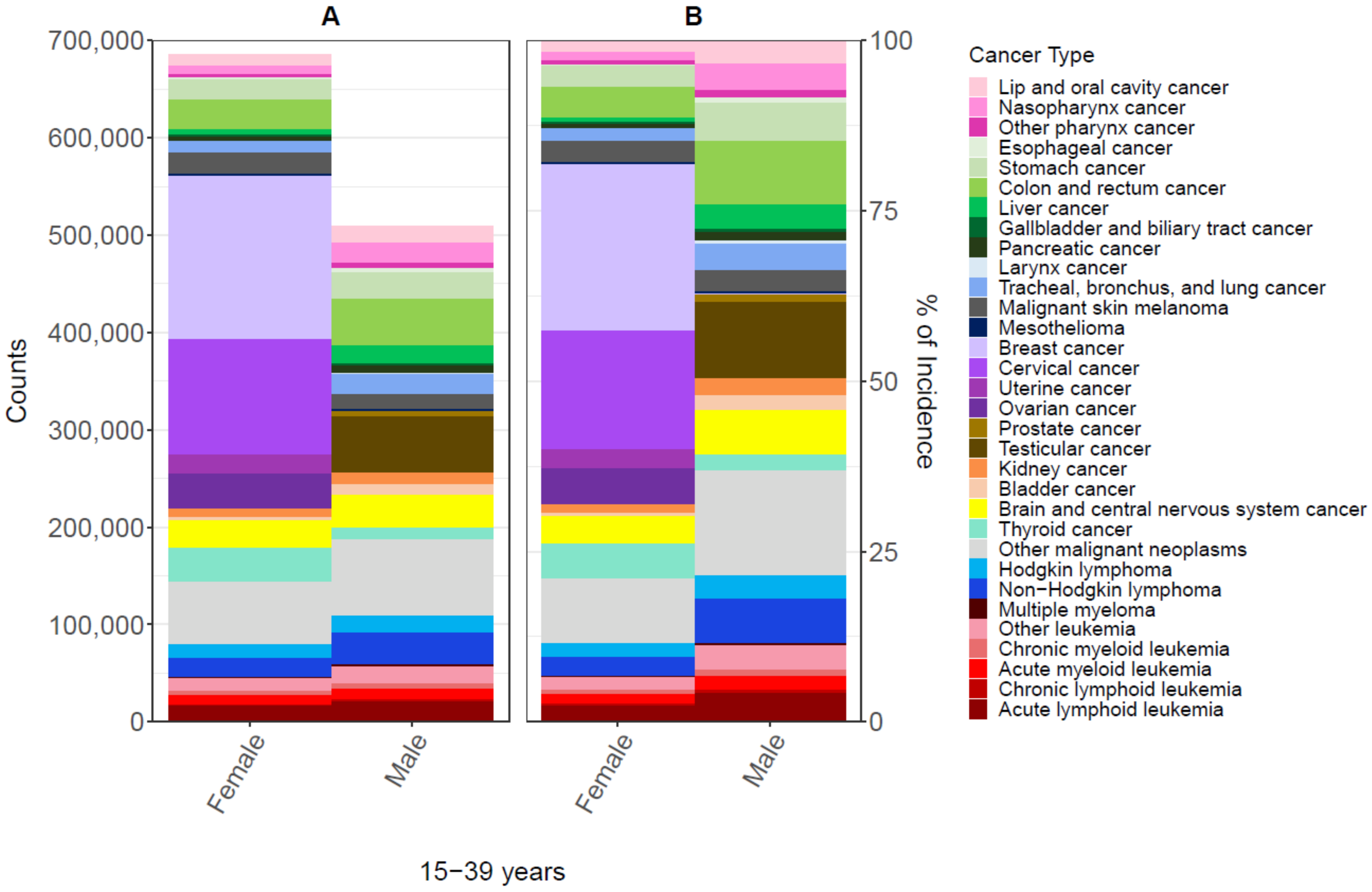

Appendix Figure 6: (A) Absolute and (B) proportional adolescent and young adult (15-39) cancer incidence burden, globally, by sex in 2019. Other malignant neoplasms: all malignancies without a separate GBD cause category listed; this category does not include non-melanoma skin cancers and myelodysplastic/myeloproliferative neoplasms, which are separate GBD cause categories not included in this analysis. Other leukaemia included leukaemias not otherwise specified. DALYs=disability-adjusted life-years. GBD=Global Burden of Diseases, Injuries, and Risk Factors Study.

A

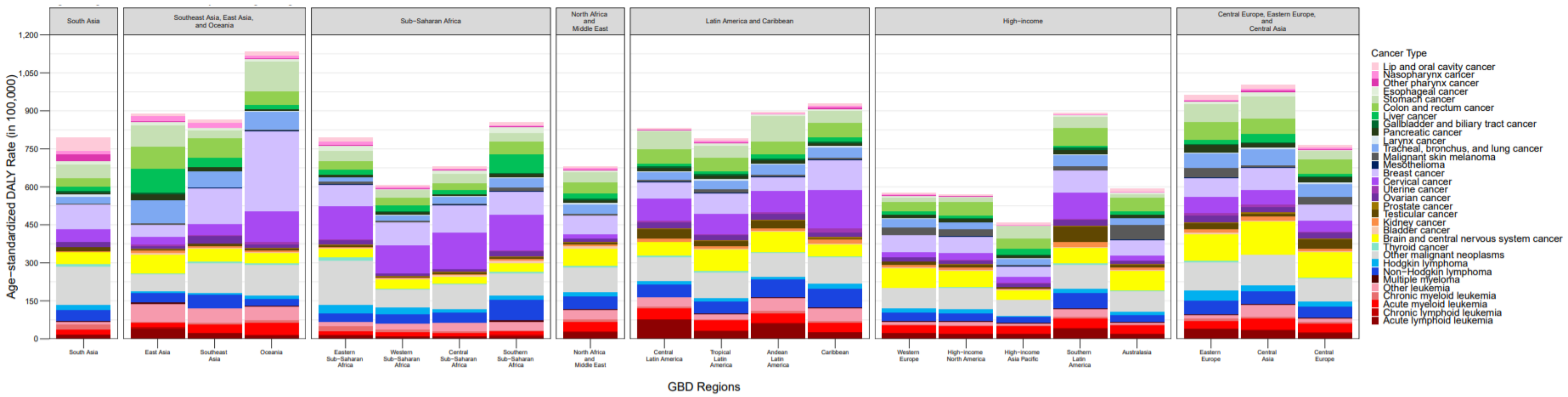

B

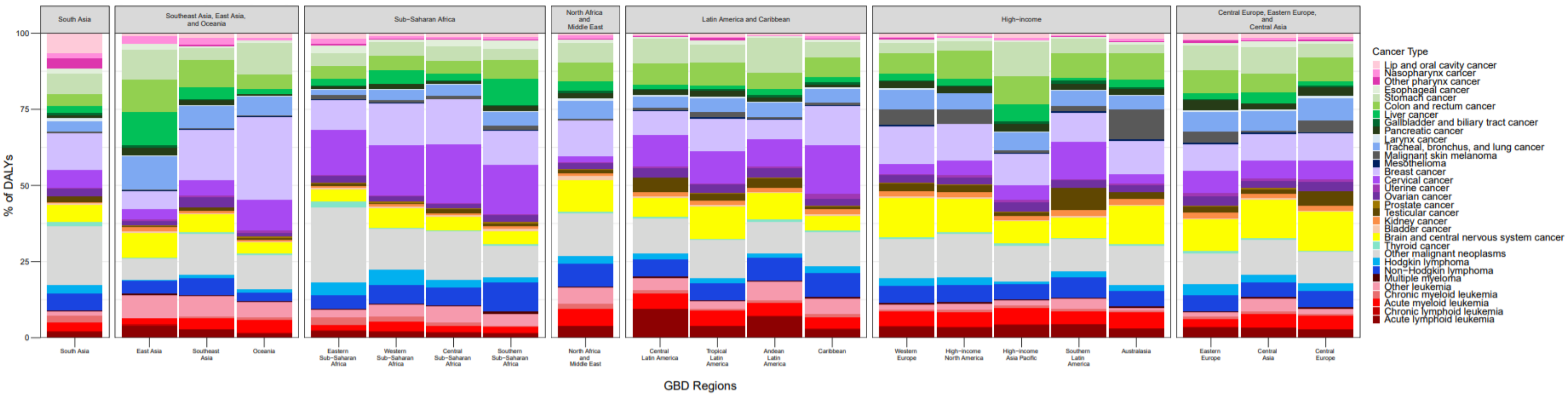

Appendix Figure 7: Adolescent and young adult (15-39 years) cancer, both sexes combined, 2019 by GBD regions in (A) age-standardised DALY rates, and (B) proportional DALY burden. Rates are expressed per 100,000 person-years. Other malignant neoplasms: all malignancies without a separate GBD cause category listed; this category does not include non-melanoma skin cancers and myelodysplastic/myeloproliferative neoplasms, which are separate GBD cause categories not included in this analysis. Other leukaemia included leukaemias not otherwise specified. DALYs=disability-adjusted life-years. GBD 2019 super-regions and regions are defined on appendix pages 58 and 59. GBD=Global Burden of Diseases, Injuries, and Risk Factors Study.

Location	Other malignant neoplasms	Leukaemia	Breast cancer	Brain and central nervous system cancer	Colon and rectum cancer	Stomach cancer	Cervical cancer	Tracheal, bronchus, and lung cancer	Non-Hodgkin lymphoma	Liver cancer	Lip and oral cavity cancer	Ovarian cancer	Hodgkin lymphoma	Pancreatic cancer	Nasopharynx cancer	Testicular cancer	Oesophageal cancer	Malignant skin melanoma	Other pharynx cancer	Kidney cancer	Thyroid cancer	Gallbladder and biliary tract cancer	Larynx cancer	Bladder cancer	Uterine cancer	Multiple myeloma	Mesothelioma	Prostate cancer
Global	1	2	3	4	5	6	7	8	9	10	11	12	13	14	15	16	17	18	19	20	21	22	23	24	25	26	27	28
High SDI	1	3	2	4	5	8	9	6	7	11	17	13	15	12	19	14	18	10	25	16	20	24	27	22	21	23	28	26
High-middle SDI	2	1	5	4	3	7	9	6	8	10	18	12	15	11	13	14	19	16	26	17	23	20	25	22	21	24	27	28
Middle SDI	2	1	3	5	4	7	10	6	9	8	13	11	15	12	14	17	16	22	19	18	20	23	25	21	24	26	27	28
Low-middle SDI	1	3	2	6	8	5	4	9	7	11	10	14	12	18	16	17	15	23	13	21	19	22	20	24	25	26	27	28
Low SDI	1	4	3	6	9	5	2	12	7	11	10	13	8	20	15	17	14	18	19	23	16	24	21	22	25	26	28	27
Central Europe, Eastern Europe, and Central Asia super-region	2	3	4	1	6	5	7	8	9	15	16	12	10	13	22	14	18	11	21	17	23	28	20	24	19	26	25	27
**Central Asia region**	3	1	4	2	7	5	8	6	9	10	16	12	11	14	19	17	13	21	22	15	27	28	20	24	18	25	26	23
Armenia	3	2	4	1	6	7	8	5	9	11	19	12	15	10	22	13	26	17	28	16	24	27	20	21	18	25	14	23
Azerbaijan	2	1	5	3	7	6	8	4	9	15	19	12	10	13	24	16	11	22	25	14	23	27	17	21	20	26	28	18
Georgia	3	2	1	5	7	6	8	4	9	14	20	12	11	13	26	10	21	16	25	17	23	27	18	22	15	24	28	19
Kazakhstan	1	2	5	4	7	6	3	8	9	11	17	10	12	13	22	16	18	15	25	14	21	28	24	23	19	26	27	20
Kyrgyzstan	2	1	6	4	7	3	5	8	9	12	17	10	15	11	18	14	16	20	21	13	22	25	23	24	19	27	28	26
Mongolia	3	4	8	5	7	2	6	9	10	1	16	12	18	13	22	26	11	24	25	15	23	17	28	21	14	20	27	19
Tajikistan	4	3	5	1	6	2	9	7	8	10	18	14	16	15	22	28	11	20	23	12	27	26	19	21	13	25	17	24
Turkmenistan	1	2	4	3	8	5	6	7	11	10	17	13	9	19	20	16	12	22	23	15	25	28	24	26	27	14	21	18
Uzbekistan	3	2	4	1	8	5	9	7	6	10	13	14	11	15	17	18	12	22	21	16	28	26	20	23	19	24	25	27

Location	Other malignant neoplasms	Leukaemia	Breast cancer	Brain and central nervous system cancer	Colon and rectum cancer	Stomach cancer	Cervical cancer	Tracheal, bronchus, and lung cancer	Non-Hodgkin lymphoma	Liver cancer	Lip and oral cavity cancer	Ovarian cancer	Hodgkin lymphoma	Pancreatic cancer	Nasopharynx cancer	Testicular cancer	Oesophageal cancer	Malignant skin melanoma	Other pharynx cancer	Kidney cancer	Thyroid cancer	Gallbladder and biliary tract cancer	Larynx cancer	Bladder cancer	Uterine cancer	Multiple myeloma	Mesothelioma	Prostate cancer
**Central Europe region**	2	3	4	1	5	10	7	6	8	16	17	12	14	13	22	9	19	11	20	15	25	24	21	23	18	26	27	28
Albania	1	3	6	2	7	5	11	4	8	10	15	14	13	12	24	9	21	16	26	17	22	23	20	25	19	27	28	18
Bosnia and Herzegovina	4	3	5	1	2	8	9	6	7	15	18	10	14	13	27	12	21	11	19	16	24	22	20	23	17	26	25	28
Bulgaria	7	2	4	1	6	9	5	3	8	15	18	11	13	12	24	10	21	14	20	17	23	26	19	22	16	25	28	27
Croatia	2	4	3	1	5	10	11	6	7	16	17	12	14	13	26	9	19	8	18	15	25	23	20	22	24	21	27	28
Czechia	2	4	3	1	5	11	6	7	10	16	17	12	14	13	26	8	18	9	21	15	22	20	25	24	19	23	27	28
Hungary	4	3	2	1	6	11	7	5	9	17	14	13	16	12	21	8	20	10	18	15	26	23	19	24	22	25	27	28
Montenegro	5	4	3	1	6	12	7	2	8	14	18	15	9	13	27	10	20	11	23	17	21	24	16	22	19	25	28	26
North Macedonia	5	3	2	1	6	7	8	4	11	14	18	13	15	12	25	9	23	10	21	17	27	22	19	20	16	26	28	24
Poland	2	3	4	1	5	10	11	6	7	18	16	12	14	13	24	8	17	9	20	15	26	21	19	22	23	25	27	28
Romania	2	4	7	1	5	9	3	6	8	17	16	11	14	12	18	10	22	13	19	15	24	26	20	23	21	25	27	28
Serbia	3	4	2	1	6	11	7	5	8	15	17	12	13	14	20	9	19	10	21	16	24	25	22	23	18	26	27	28
Slovakia	2	3	5	1	4	10	6	7	8	17	15	12	13	14	25	9	20	11	18	16	24	21	22	26	19	23	27	28
Slovenia	2	3	4	1	5	9	11	7	8	14	17	12	15	13	27	10	19	6	18	16	23	21	24	22	26	20	25	28
**Eastern Europe region**	2	4	3	1	6	5	7	8	9	16	15	13	11	12	25	14	19	10	20	17	21	28	22	24	18	26	23	27
Belarus	1	2	5	3	7	4	6	9	8	18	17	13	10	14	26	12	19	11	23	15	22	28	21	27	25	24	16	20
Estonia	3	1	4	2	6	5	9	8	7	16	17	12	11	14	23	13	19	10	21	15	18	24	26	25	22	20	28	27
Latvia	2	3	4	1	7	5	9	8	6	15	17	14	11	12	25	13	18	10	19	16	24	26	20	22	21	23	28	27

Location	Other malignant neoplasms	Leukaemia	Breast cancer	Brain and central nervous system cancer	Colon and rectum cancer	Stomach cancer	Cervical cancer	Tracheal, bronchus, and lung cancer	Non-Hodgkin lymphoma	Liver cancer	Lip and oral cavity cancer	Ovarian cancer	Hodgkin lymphoma	Pancreatic cancer	Nasopharynx cancer	Testicular cancer	Oesophageal cancer	Malignant skin melanoma	Other pharynx cancer	Kidney cancer	Thyroid cancer	Gallbladder and biliary tract cancer	Larynx cancer	Bladder cancer	Uterine cancer	Multiple myeloma	Mesothelioma	Prostate cancer
Lithuania	2	3	4	1	6	5	8	9	7	16	17	11	13	12	24	14	18	10	21	15	22	26	20	25	19	23	27	28
Republic of Moldova	2	6	4	1	3	8	5	7	9	18	16	12	11	10	17	14	22	13	19	15	24	27	20	23	21	25	28	26
Russia	2	6	3	1	7	5	4	8	9	14	15	11	12	13	25	16	19	10	22	17	20	27	21	23	18	24	26	28
Ukraine	1	3	5	2	6	4	11	8	7	17	15	13	9	12	23	14	19	10	20	16	25	28	22	26	21	27	18	24
High-income super-region	1	3	2	4	5	9	8	6	7	13	17	12	15	14	23	11	18	10	25	16	20	22	28	21	19	24	26	27
**Australasia region**	1	5	3	2	6	10	9	8	7	11	16	13	15	12	20	14	18	4	26	17	19	25	28	23	22	21	24	27
Australia	2	5	3	1	6	10	9	8	7	11	15	13	14	12	20	16	18	4	26	17	19	25	28	22	23	21	24	27
New Zealand	1	4	2	3	6	9	12	8	7	11	17	13	14	15	20	10	19	5	26	16	18	24	28	23	22	21	27	25
**High-income Asia-Pacific region**	2	1	4	6	5	3	10	7	9	8	13	11	21	12	19	15	22	17	27	14	20	16	28	23	18	24	26	25
Brunei Darussalam	3	1	2	6	5	8	9	7	4	10	13	11	14	15	12	16	24	18	23	17	21	20	28	25	19	22	27	26
Japan	1	2	3	6	4	5	8	7	9	11	13	10	19	12	22	14	20	18	27	15	23	17	28	21	16	24	25	26
Singapore	2	1	3	5	4	10	12	6	7	9	15	11	16	13	8	19	22	17	26	14	20	21	27	23	18	24	28	25
South Korea	3	2	5	7	6	1	10	8	9	4	16	12	19	11	18	22	21	17	27	14	15	13	26	23	20	24	28	25
**High-income North America region**	1	3	2	4	5	10	8	7	6	14	18	12	11	13	23	15	17	9	26	16	20	24	27	22	19	21	28	25
Canada	1	4	3	2	5	10	9	7	6	11	17	13	12	15	22	14	18	8	27	16	21	24	28	19	23	20	25	26
Greenland	4	8	6	5	2	7	1	3	9	13	19	12	18	11	10	14	15	17	20	16	23	21	26	25	28	22	24	27
United States of America	1	3	2	4	5	10	8	7	6	14	18	11	12	13	23	15	17	9	26	16	20	24	27	22	19	21	28	25

Location	Other malignant neoplasms	Leukaemia	Breast cancer	Brain and central nervous system cancer	Colon and rectum cancer	Stomach cancer	Cervical cancer	Tracheal, bronchus, and lung cancer	Non-Hodgkin lymphoma	Liver cancer	Lip and oral cavity cancer	Ovarian cancer	Hodgkin lymphoma	Pancreatic cancer	Nasopharynx cancer	Testicular cancer	Oesophageal cancer	Malignant skin melanoma	Other pharynx cancer	Kidney cancer	Thyroid cancer	Gallbladder and biliary tract cancer	Larynx cancer	Bladder cancer	Uterine cancer	Multiple myeloma	Mesothelioma	Prostate cancer
**Southern Latin America region**	3	1	4	7	5	10	2	9	8	19	18	11	14	12	26	6	17	15	28	13	20	16	25	23	21	22	24	27
Argentina	4	2	3	6	5	10	1	9	7	19	18	11	14	12	26	8	16	15	28	13	20	17	24	22	21	23	25	27
Chile	2	1	5	7	6	8	4	10	9	17	19	11	15	16	25	3	21	14	28	12	18	13	27	22	26	20	23	24
Uruguay	5	1	2	7	6	10	3	8	4	17	16	13	12	14	21	9	18	15	28	11	22	19	23	24	25	20	26	27
**Western Europe region**	1	4	3	2	5	9	10	6	7	15	17	12	11	14	22	13	18	8	24	16	20	25	26	19	23	21	27	28
Andorra	3	2	4	1	7	9	8	5	6	11	18	16	13	12	23	14	17	10	25	15	21	19	26	24	22	20	27	28
Austria	1	4	3	2	8	11	10	6	7	14	17	13	15	12	27	9	18	5	20	16	23	24	26	22	19	21	28	25
Belgium	3	4	1	2	6	11	9	5	7	14	17	10	13	12	25	15	18	8	22	16	23	28	24	19	21	20	26	27
Cyprus	1	4	3	2	6	9	12	7	5	14	17	10	11	13	22	15	19	8	27	16	21	24	25	18	26	20	28	23
Denmark	2	3	4	1	5	14	9	6	8	15	18	11	12	13	26	10	17	7	22	16	20	24	27	19	23	21	25	28
Finland	2	3	4	1	6	9	15	8	5	13	17	10	11	12	27	14	20	7	24	16	18	21	28	23	22	19	26	25
France	1	4	2	3	6	11	9	5	8	13	17	14	12	15	23	10	18	7	20	16	24	27	25	19	21	22	26	28
Germany	2	4	3	1	5	9	10	6	7	15	17	13	14	12	25	11	18	8	21	16	19	20	26	22	24	23	28	27
Greece	4	3	2	1	6	9	11	5	8	16	17	14	7	12	18	13	20	10	28	15	26	24	23	21	19	22	25	27
Iceland	2	4	3	1	8	10	13	5	6	15	18	9	14	11	23	19	17	7	27	12	16	25	28	21	22	20	24	26
Ireland	3	4	2	1	5	11	9	8	7	17	18	10	12	13	23	14	16	6	26	15	21	25	28	19	22	20	24	27
Israel	3	4	1	2	6	9	11	7	5	14	17	12	10	13	22	16	19	8	28	15	18	26	25	21	23	20	24	27
Italy	2	1	3	4	6	9	13	7	5	16	17	12	10	14	18	11	21	8	26	15	19	24	27	23	20	22	25	28

Location	Other malignant neoplasms	Leukaemia	Breast cancer	Brain and central nervous system cancer	Colon and rectum cancer	Stomach cancer	Cervical cancer	Tracheal, bronchus, and lung cancer	Non-Hodgkin lymphoma	Liver cancer	Lip and oral cavity cancer	Ovarian cancer	Hodgkin lymphoma	Pancreatic cancer	Nasopharynx cancer	Testicular cancer	Oesophageal cancer	Malignant skin melanoma	Other pharynx cancer	Kidney cancer	Thyroid cancer	Gallbladder and biliary tract cancer	Larynx cancer	Bladder cancer	Uterine cancer	Multiple myeloma	Mesothelioma	Prostate cancer
Luxembourg	3	2	4	1	8	11	13	5	6	15	16	9	14	12	21	10	17	7	22	18	20	27	25	23	19	24	26	28
Malta	1	4	2	3	7	12	14	6	5	17	18	11	9	13	16	10	19	8	26	15	20	28	24	21	22	23	25	27
Monaco	6	2	4	5	7	14	15	3	1	13	22	11	9	12	23	8	18	10	27	16	21	26	20	19	28	17	25	24
Netherlands	1	4	2	3	7	10	9	6	8	14	18	11	13	15	24	12	17	5	26	16	20	21	28	19	23	22	25	27
Norway	2	5	3	1	6	14	9	8	7	12	18	10	15	13	26	11	20	4	27	16	19	24	28	21	23	17	22	25
Portugal	4	3	2	1	5	8	9	7	6	11	17	13	12	14	19	15	18	10	20	16	25	26	22	23	21	24	28	27
San Marino	1	3	4	2	8	7	11	6	5	14	17	13	10	12	18	16	22	9	26	15	19	23	25	20	28	21	27	24
Spain	1	3	2	4	6	8	11	5	7	14	17	13	12	10	20	16	18	9	24	15	21	27	25	19	22	23	26	28
Sweden	2	4	3	1	6	14	9	7	8	12	17	10	15	11	26	13	19	5	27	16	22	18	28	21	24	20	23	25
Switzerland	1	4	3	2	6	10	12	5	8	13	17	14	15	11	23	9	18	7	22	16	20	24	28	21	26	19	27	25
United Kingdom	2	4	1	3	5	12	9	8	6	13	18	10	11	14	20	17	16	7	23	15	22	26	27	19	24	21	25	28
Latin America and Caribbean super-region	2	1	4	5	7	6	3	9	8	14	18	11	12	13	24	10	17	16	27	15	21	19	23	26	20	22	25	28
**Andean Latin America region**	3	1	7	5	8	2	4	9	6	12	20	11	15	13	27	10	21	17	28	14	18	16	25	23	19	22	26	24
Bolivia (Plurinational State of)	4	1	6	5	8	2	3	9	7	12	19	11	13	15	27	10	21	14	28	17	18	16	24	23	20	22	25	26
Ecuador	3	1	8	6	7	2	4	9	5	15	20	11	12	14	26	10	21	16	28	13	17	19	25	23	18	22	27	24
Peru	3	1	7	4	8	2	5	9	6	12	20	10	15	13	27	11	21	17	28	14	19	16	25	24	18	22	26	23
**Caribbean region**	4	2	3	8	6	7	1	9	5	12	16	11	10	15	20	19	17	18	26	14	21	22	23	25	13	24	28	27

Location	Other malignant neoplasms	Leukaemia	Breast cancer	Brain and central nervous system cancer	Colon and rectum cancer	Stomach cancer	Cervical cancer	Tracheal, bronchus, and lung cancer	Non-Hodgkin lymphoma	Liver cancer	Lip and oral cavity cancer	Ovarian cancer	Hodgkin lymphoma	Pancreatic cancer	Nasopharynx cancer	Testicular cancer	Oesophageal cancer	Malignant skin melanoma	Other pharynx cancer	Kidney cancer	Thyroid cancer	Gallbladder and biliary tract cancer	Larynx cancer	Bladder cancer	Uterine cancer	Multiple myeloma	Mesothelioma	Prostate cancer
Antigua and Barbuda	3	2	1	7	6	8	5	10	4	13	19	9	18	12	23	16	20	14	26	11	22	25	27	24	15	21	28	17
Bahamas	2	3	1	7	6	8	4	9	5	12	18	10	14	15	21	28	17	16	23	11	22	24	25	27	13	19	26	20
Barbados	4	2	1	7	6	8	5	11	3	15	18	9	14	12	21	23	17	19	24	10	22	25	28	26	13	16	27	20
Belize	3	2	4	8	7	6	1	9	5	13	18	14	10	12	21	16	20	17	27	11	25	23	26	24	15	28	19	22
Bermuda	2	3	1	6	5	14	9	7	4	16	19	8	13	11	23	25	17	10	26	12	20	28	27	21	15	22	18	24
Cuba	2	1	6	3	7	12	4	8	5	18	17	11	9	14	22	15	19	16	25	13	21	27	20	23	10	24	26	28
Dominica	3	1	5	9	7	6	4	8	2	14	16	13	10	12	21	22	18	20	24	11	23	26	27	25	15	17	28	19
Dominican Republic	2	1	4	11	5	8	3	7	6	9	13	15	21	17	23	26	16	25	20	10	19	14	18	27	12	24	28	22
Grenada	4	5	3	7	6	9	2	10	1	15	17	8	14	12	21	23	16	18	27	13	20	25	28	24	11	22	26	19
Guyana	3	2	4	9	5	8	1	10	6	15	17	7	11	14	23	16	22	18	28	12	20	24	27	25	13	26	21	19
Haiti	4	3	2	7	8	5	1	9	6	13	16	11	10	19	17	22	18	14	28	15	20	21	26	25	12	23	24	27
Jamaica	5	3	1	8	6	9	2	7	4	11	19	10	14	15	13	23	18	22	24	12	17	21	26	25	16	20	27	28
Puerto Rico	3	2	1	7	5	12	6	8	4	17	19	10	9	14	23	11	20	16	26	13	21	27	24	22	15	18	28	25
Saint Kitts and Nevis	1	2	3	7	6	8	4	12	5	14	17	9	23	13	16	10	18	24	28	11	19	26	27	25	15	22	21	20
Saint Lucia	5	3	4	8	7	6	2	9	1	18	16	10	11	12	21	13	19	17	26	14	23	27	25	24	15	20	28	22
Saint Vincent and the Grenadines	5	4	3	8	6	7	1	10	2	15	16	9	11	13	19	20	22	17	24	14	18	27	23	25	12	26	28	21
Suriname	2	4	3	6	7	10	1	8	5	14	19	9	11	12	16	15	24	18	27	13	20	23	28	26	17	21	25	22
Trinidad and Tobago	3	2	1	8	6	11	5	7	4	14	16	9	12	13	20	15	18	25	26	10	21	22	27	19	17	24	28	23

Location	Other malignant neoplasms	Leukaemia	Breast cancer	Brain and central nervous system cancer	Colon and rectum cancer	Stomach cancer	Cervical cancer	Tracheal, bronchus, and lung cancer	Non-Hodgkin lymphoma	Liver cancer	Lip and oral cavity cancer	Ovarian cancer	Hodgkin lymphoma	Pancreatic cancer	Nasopharynx cancer	Testicular cancer	Oesophageal cancer	Malignant skin melanoma	Other pharynx cancer	Kidney cancer	Thyroid cancer	Gallbladder and biliary tract cancer	Larynx cancer	Bladder cancer	Uterine cancer	Multiple myeloma	Mesothelioma	Prostate cancer
United States Virgin Islands	5	2	1	6	3	10	7	8	4	14	18	9	17	12	20	28	19	13	25	11	23	24	26	27	15	16	21	22
**Central Latin America region**	2	1	5	7	6	4	3	10	8	14	17	11	12	13	25	9	18	16	28	15	19	20	24	23	21	22	27	26
Colombia	2	1	6	5	7	3	4	9	8	15	19	11	12	13	26	10	18	14	28	16	20	17	25	23	22	21	27	24
Costa Rica	2	1	7	8	4	3	6	12	5	11	18	13	9	15	17	10	21	16	25	14	19	20	26	24	23	22	28	27
El Salvador	4	1	7	5	6	3	2	9	8	15	16	10	11	12	23	13	18	22	27	14	20	17	21	25	19	24	28	26
Guatemala	4	1	7	6	5	2	3	8	9	11	17	12	14	15	24	10	16	21	27	13	20	19	23	25	22	26	28	18
Honduras	2	1	7	6	9	4	3	5	10	8	14	11	18	16	28	15	24	22	23	17	12	19	21	26	13	20	25	27
Mexico	2	1	4	8	6	7	3	11	9	15	17	10	12	13	26	5	18	16	28	14	19	20	25	23	22	21	24	27
Nicaragua	3	1	6	5	7	4	2	9	8	12	16	10	14	13	18	11	22	20	28	15	19	17	24	23	21	25	27	26
Panama	2	1	5	4	7	6	3	9	8	12	16	10	15	13	21	11	19	18	26	14	17	22	24	25	23	20	28	27
Venezuela (Bolivarian Republic of)	4	2	3	9	6	5	1	8	7	15	16	10	11	14	22	13	19	17	27	12	20	21	23	26	18	24	28	25
**Tropical Latin America region**	1	2	4	3	6	7	5	9	8	17	18	10	13	12	23	11	15	14	20	16	24	19	21	27	26	25	22	28
Brazil	1	2	4	3	6	7	5	9	8	17	18	10	13	12	23	11	15	14	20	16	25	19	22	27	26	24	21	28
Paraguay	3	2	4	7	5	8	1	9	6	17	16	11	12	13	26	10	18	15	21	14	19	22	23	27	20	25	28	24
North Africa and Middle East super-region	2	1	3	4	7	6	11	8	5	9	21	12	10	13	17	18	15	22	27	16	19	23	20	14	25	24	28	26
**North Africa and Middle East region**	2	1	3	4	7	6	11	8	5	9	21	12	10	13	17	18	15	22	27	16	19	23	20	14	25	24	28	26

Location	Other malignant neoplasms	Leukaemia	Breast cancer	Brain and central nervous system cancer	Colon and rectum cancer	Stomach cancer	Cervical cancer	Tracheal, bronchus, and lung cancer	Non-Hodgkin lymphoma	Liver cancer	Lip and oral cavity cancer	Ovarian cancer	Hodgkin lymphoma	Pancreatic cancer	Nasopharynx cancer	Testicular cancer	Oesophageal cancer	Malignant skin melanoma	Other pharynx cancer	Kidney cancer	Thyroid cancer	Gallbladder and biliary tract cancer	Larynx cancer	Bladder cancer	Uterine cancer	Multiple myeloma	Mesothelioma	Prostate cancer
Afghanistan	1	2	6	5	8	3	9	11	4	7	18	13	10	20	16	23	12	17	26	22	15	19	14	21	25	24	28	27
Algeria	1	4	2	5	7	11	6	10	3	13	18	12	8	14	9	19	24	20	23	17	16	15	21	25	27	22	28	26
Bahrain	3	1	2	6	5	8	13	7	4	12	15	10	11	9	16	27	18	22	25	14	21	23	26	19	24	17	28	20
Egypt	1	2	3	4	7	10	15	8	6	5	21	12	13	11	23	20	17	25	27	14	18	19	16	9	22	24	28	26
Iran (Islamic Republic of)	4	1	3	2	7	5	14	6	8	10	19	11	9	12	26	17	13	18	27	15	20	23	16	21	24	22	28	25
Iraq	3	1	4	2	6	8	13	7	5	10	16	9	11	12	23	15	20	25	27	14	19	24	18	17	22	21	26	28
Jordan	2	1	3	5	6	8	12	7	4	14	13	9	16	11	17	10	19	22	28	15	18	21	25	20	23	24	27	26
Kuwait	3	1	2	4	5	9	11	7	6	12	16	8	14	10	24	25	17	23	27	13	19	21	26	15	20	22	28	18
Lebanon	1	3	2	4	5	9	11	6	8	13	18	10	7	12	22	14	25	17	27	19	15	23	20	16	24	21	28	26
Libya	1	2	3	4	5	10	12	8	7	11	17	14	6	13	9	21	24	23	27	16	18	20	15	19	25	22	28	26
Morocco	2	4	1	5	6	12	7	8	3	17	14	10	9	15	11	19	21	20	24	18	13	23	16	26	25	22	28	27
Oman	1	2	5	4	6	7	13	10	3	8	16	12	9	11	20	23	18	22	27	15	17	25	26	24	28	19	14	21
Palestine	3	1	4	2	6	8	13	5	7	10	16	11	9	12	20	24	21	22	27	14	17	23	26	19	15	18	28	25
Qatar	2	1	3	4	6	8	13	7	5	9	16	14	11	10	20	18	15	23	27	12	24	22	21	25	28	19	26	17
Saudi Arabia	1	2	3	5	6	8	13	7	4	12	17	10	11	9	14	19	18	24	27	15	16	20	25	23	26	22	28	21
Sudan	2	1	4	3	7	5	10	8	6	11	17	13	9	14	21	20	12	19	27	15	18	23	16	22	25	24	28	26
Syrian Arab Republic	4	1	3	2	6	8	12	5	7	9	17	10	14	11	22	20	19	23	26	15	25	27	18	16	13	21	28	24
Tunisia	2	3	1	6	5	8	11	4	9	15	14	12	10	13	7	23	24	19	25	18	16	20	17	21	27	22	28	26

Location	Other malignant neoplasms	Leukaemia	Breast cancer	Brain and central nervous system cancer	Colon and rectum cancer	Stomach cancer	Cervical cancer	Tracheal, bronchus, and lung cancer	Non-Hodgkin lymphoma	Liver cancer	Lip and oral cavity cancer	Ovarian cancer	Hodgkin lymphoma	Pancreatic cancer	Nasopharynx cancer	Testicular cancer	Oesophageal cancer	Malignant skin melanoma	Other pharynx cancer	Kidney cancer	Thyroid cancer	Gallbladder and biliary tract cancer	Larynx cancer	Bladder cancer	Uterine cancer	Multiple myeloma	Mesothelioma	Prostate cancer
Turkey	2	1	4	3	5	7	14	6	8	15	19	11	12	10	18	9	16	17	28	13	23	25	21	22	26	24	20	27
United Arab Emirates	1	4	5	2	7	13	20	10	3	11	17	14	12	6	24	21	8	22	26	9	19	23	15	16	27	18	28	25
Yemen	3	1	4	5	7	2	10	8	6	12	16	14	9	15	19	22	11	17	27	21	18	20	13	23	25	24	28	26
South Asia super-region	1	3	2	7	9	4	6	10	8	14	5	13	12	20	17	15	16	23	11	22	18	21	19	24	26	25	27	28
**South Asia region**	1	3	2	7	9	4	6	10	8	14	5	13	12	20	17	15	16	23	11	22	18	21	19	24	26	25	27	28
Bangladesh	1	3	2	4	12	6	5	10	7	9	8	14	11	19	15	17	16	23	13	22	18	21	20	25	28	24	27	26
Bhutan	1	2	3	4	9	7	5	10	6	14	8	11	12	19	17	16	15	24	13	22	18	20	21	25	27	23	28	26
India	1	3	2	6	9	4	5	11	8	13	7	14	12	20	15	17	16	24	10	22	18	21	19	25	27	23	26	28
Nepal	1	2	4	6	11	5	3	10	7	14	8	9	12	19	16	18	15	23	13	22	17	20	21	25	26	24	27	28
Pakistan	1	4	2	6	9	12	13	10	8	15	3	7	5	23	17	11	14	22	18	25	16	20	19	21	24	26	28	27
Southeast Asia, East Asia, and Oceania super-region	5	1	4	8	2	7	10	3	9	6	16	13	17	12	11	18	14	23	27	15	21	20	25	22	19	24	26	28
**East Asia region**	7	1	8	6	4	5	10	3	9	2	16	14	23	11	12	22	13	21	28	15	24	17	25	18	19	20	26	27
China	7	1	8	6	4	5	10	3	9	2	16	14	23	11	12	22	13	21	28	15	24	17	25	18	19	20	26	27
North Korea	5	1	8	7	6	2	9	4	10	3	16	14	19	12	11	23	13	21	28	15	22	18	25	20	17	24	26	27
Taiwan (Province of China)	5	2	4	8	1	7	14	3	9	11	6	15	24	13	10	18	12	20	16	17	23	19	27	22	21	26	25	28
**Oceania region**	3	2	1	8	7	4	5	6	9	10	11	12	13	18	15	16	17	22	25	24	20	26	28	19	14	23	27	21
American Samoa	1	5	2	10	4	3	8	6	7	9	18	11	17	14	15	27	23	21	24	20	19	26	28	16	12	22	25	13

Location	Other malignant neoplasms	Leukaemia	Breast cancer	Brain and central nervous system cancer	Colon and rectum cancer	Stomach cancer	Cervical cancer	Tracheal, bronchus, and lung cancer	Non-Hodgkin lymphoma	Liver cancer	Lip and oral cavity cancer	Ovarian cancer	Hodgkin lymphoma	Pancreatic cancer	Nasopharynx cancer	Testicular cancer	Oesophageal cancer	Malignant skin melanoma	Other pharynx cancer	Kidney cancer	Thyroid cancer	Gallbladder and biliary tract cancer	Larynx cancer	Bladder cancer	Uterine cancer	Multiple myeloma	Mesothelioma	Prostate cancer
Cook Islands	2	5	1	7	9	6	10	3	8	4	13	12	17	11	24	21	16	22	26	18	19	25	28	15	20	23	27	14
Federated States of Micronesia	4	2	1	10	7	3	5	6	9	8	12	11	15	14	16	24	17	22	25	21	18	26	28	19	13	23	27	20
Fiji	4	2	1	9	5	7	3	10	6	8	12	17	13	15	25	11	18	21	24	20	16	26	28	19	14	23	27	22
Guam	6	2	4	10	3	7	9	1	5	8	14	12	20	13	11	25	15	24	21	16	18	27	28	17	19	23	26	22
Kiribati	2	6	3	13	10	4	1	9	11	5	7	16	14	20	18	8	12	24	19	15	26	23	27	22	17	25	28	21
Marshall Islands	5	2	1	10	7	3	4	6	9	8	12	11	15	16	14	24	17	22	25	21	19	26	28	18	13	23	27	20
Nauru	4	2	1	10	7	3	6	5	8	9	11	12	17	13	15	24	16	23	25	20	19	26	27	18	14	22	28	21
Niue	2	3	1	10	5	7	6	4	8	9	13	11	18	12	15	24	16	22	25	17	20	26	28	19	14	23	27	21
Northern Mariana Islands	2	1	4	9	5	7	8	3	6	10	11	13	22	12	14	25	17	24	16	18	19	26	27	21	15	23	28	20
Palau	1	9	4	11	8	6	3	2	10	7	5	17	21	12	22	28	14	16	20	18	13	25	26	19	27	23	24	15
Papua New Guinea	2	3	1	8	7	4	5	6	10	19	9	11	12	16	13	22	15	21	25	24	20	26	28	17	14	23	27	18
Samoa	1	2	3	9	8	6	4	14	5	10	18	11	7	16	13	12	22	15	26	21	17	24	28	20	19	25	27	23
Solomon Islands	5	4	1	10	8	2	3	7	6	9	12	13	15	17	14	26	16	20	25	23	19	24	27	18	11	22	28	21
Tokelau	3	2	1	10	7	5	4	6	9	8	13	11	16	14	15	24	18	23	25	21	20	26	28	17	12	22	27	19
Tonga	5	8	2	10	11	7	6	9	4	3	13	12	15	14	17	1	19	23	25	22	24	26	28	21	16	20	27	18
Tuvalu	3	2	1	10	7	4	5	6	9	8	12	11	15	16	13	24	17	22	25	21	20	26	27	19	14	23	28	18
Vanuatu	4	1	2	10	8	3	5	6	9	7	11	13	12	17	14	24	16	21	25	22	20	26	28	18	15	23	27	19
**Southeast Asia region**	3	2	1	6	4	10	8	5	7	9	14	11	15	13	12	16	18	22	23	17	19	21	25	26	20	28	24	27

Location	Other malignant neoplasms	Leukaemia	Breast cancer	Brain and central nervous system cancer	Colon and rectum cancer	Stomach cancer	Cervical cancer	Tracheal, bronchus, and lung cancer	Non-Hodgkin lymphoma	Liver cancer	Lip and oral cavity cancer	Ovarian cancer	Hodgkin lymphoma	Pancreatic cancer	Nasopharynx cancer	Testicular cancer	Oesophageal cancer	Malignant skin melanoma	Other pharynx cancer	Kidney cancer	Thyroid cancer	Gallbladder and biliary tract cancer	Larynx cancer	Bladder cancer	Uterine cancer	Multiple myeloma	Mesothelioma	Prostate cancer
Cambodia	2	1	3	6	5	10	8	7	9	4	13	11	14	15	12	17	16	21	26	19	20	24	22	25	18	27	23	28
Indonesia	3	2	1	6	4	10	8	5	7	13	14	9	15	11	12	18	17	21	26	16	20	24	25	22	19	27	23	28
Laos	3	1	2	6	4	9	7	5	8	10	15	11	13	14	12	19	16	21	26	18	20	23	25	22	17	27	24	28
Malaysia	3	1	2	7	4	11	9	8	5	10	13	12	15	16	6	14	19	20	24	18	17	25	21	22	23	27	26	28
Maldives	2	1	4	3	6	12	10	8	5	7	14	9	11	13	23	27	17	19	25	16	18	26	24	22	28	20	15	21
Mauritius	3	2	1	7	4	8	10	6	5	17	12	9	13	11	16	18	15	25	26	14	22	24	21	23	20	19	28	27
Myanmar	3	1	4	7	5	9	8	6	2	11	15	10	14	13	12	19	16	21	26	17	20	24	25	23	18	27	22	28
Philippines	2	1	3	8	4	11	9	5	6	7	14	10	19	13	12	16	20	21	26	15	18	24	23	22	17	28	25	27
Seychelles	6	1	2	5	3	10	7	8	4	12	11	9	15	13	14	17	16	21	20	22	28	27	18	23	24	25	26	19
Sri Lanka	2	1	3	4	7	8	10	6	5	13	12	9	11	16	17	18	14	25	21	15	19	20	26	27	23	24	22	28
Thailand	3	2	5	7	6	9	8	4	10	1	14	11	19	13	12	17	15	25	24	18	22	16	21	23	26	28	20	27
Timor-Leste	2	1	3	4	5	9	8	6	7	10	14	11	13	15	12	16	17	21	26	18	20	25	24	23	19	28	22	27
Vietnam	1	3	2	8	4	6	7	5	10	17	11	12	14	16	9	15	20	22	18	19	13	24	21	25	28	26	23	27
Sub-Saharan Africa super-region	1	4	3	6	8	7	2	11	5	10	14	12	9	17	15	19	13	16	27	20	18	26	22	21	25	23	28	24
**Central sub-Saharan Africa region**	2	4	3	7	8	6	1	9	5	11	15	13	10	17	18	14	12	16	28	19	21	26	24	20	23	25	27	22
Angola	2	4	3	8	6	7	1	9	5	12	15	13	10	16	18	14	11	17	28	19	21	26	23	20	24	25	27	22
Central African Republic	2	4	3	7	8	5	1	9	6	12	13	14	10	18	17	15	11	16	28	21	20	24	22	19	23	26	27	25

Location	Other malignant neoplasms	Leukaemia	Breast cancer	Brain and central nervous system cancer	Colon and rectum cancer	Stomach cancer	Cervical cancer	Tracheal, bronchus, and lung cancer	Non-Hodgkin lymphoma	Liver cancer	Lip and oral cavity cancer	Ovarian cancer	Hodgkin lymphoma	Pancreatic cancer	Nasopharynx cancer	Testicular cancer	Oesophageal cancer	Malignant skin melanoma	Other pharynx cancer	Kidney cancer	Thyroid cancer	Gallbladder and biliary tract cancer	Larynx cancer	Bladder cancer	Uterine cancer	Multiple myeloma	Mesothelioma	Prostate cancer
Congo (Brazzaville)	3	4	2	8	6	7	1	9	5	12	15	11	13	14	19	16	10	17	28	18	22	26	25	20	21	24	27	23
DR Congo	2	4	3	7	9	6	1	8	5	11	15	13	10	17	18	14	12	16	28	20	21	26	24	19	23	25	27	22
Equatorial Guinea	1	4	3	7	6	9	2	8	5	10	16	11	15	14	19	13	12	18	26	17	22	27	25	20	24	23	28	21
Gabon	1	4	2	8	6	9	3	7	5	11	16	10	14	13	20	15	12	18	26	17	22	27	25	19	24	23	28	21
**Eastern sub-Saharan Africa region**	1	4	3	9	8	7	2	17	6	12	15	11	5	19	14	18	10	16	25	20	13	26	23	21	24	22	27	28
Burundi	1	4	3	8	9	5	2	16	7	11	14	13	6	19	12	18	10	15	24	21	17	26	20	22	25	23	27	28
Comoros	1	4	3	9	6	7	2	13	5	12	16	10	8	18	14	19	11	15	25	20	17	26	24	23	21	22	27	28
Djibouti	1	4	3	8	5	7	2	12	6	13	15	11	9	18	14	17	10	16	24	20	19	26	23	21	25	22	27	28
Eritrea	1	4	3	9	7	5	2	11	6	14	15	12	8	19	13	18	10	16	24	20	17	26	22	21	23	25	27	28
Ethiopia	1	2	4	5	8	7	3	16	11	13	14	10	6	20	12	15	18	17	27	19	9	21	23	22	24	25	26	28
Kenya	1	4	2	7	8	6	3	15	5	13	11	14	9	17	12	24	10	16	20	21	18	23	22	25	26	19	27	28
Madagascar	1	4	3	9	7	5	2	14	6	12	16	11	8	19	15	18	10	13	25	20	17	26	23	21	22	24	27	28
Malawi	1	4	3	9	10	13	2	16	6	12	15	11	8	20	21	18	5	7	28	19	14	27	25	17	23	22	24	26
Mozambique	1	4	3	7	8	11	2	13	6	10	19	9	5	17	27	15	16	12	25	18	14	24	20	21	23	22	26	28
Rwanda	1	4	3	9	6	7	2	16	5	10	15	11	8	19	14	18	12	13	24	20	17	26	23	22	25	21	27	28
Somalia	2	3	5	7	10	4	1	17	8	11	14	13	6	19	12	18	9	15	26	23	16	25	20	21	22	24	27	28
South Sudan	1	4	3	9	6	7	2	13	5	12	14	11	8	19	15	18	10	16	23	20	17	26	25	21	22	24	27	28

Location	Other malignant neoplasms	Leukaemia	Breast cancer	Brain and central nervous system cancer	Colon and rectum cancer	Stomach cancer	Cervical cancer	Tracheal, bronchus, and lung cancer	Non-Hodgkin lymphoma	Liver cancer	Lip and oral cavity cancer	Ovarian cancer	Hodgkin lymphoma	Pancreatic cancer	Nasopharynx cancer	Testicular cancer	Oesophageal cancer	Malignant skin melanoma	Other pharynx cancer	Kidney cancer	Thyroid cancer	Gallbladder and biliary tract cancer	Larynx cancer	Bladder cancer	Uterine cancer	Multiple myeloma	Mesothelioma	Prostate cancer
Tanzania	1	4	3	7	6	8	2	12	5	16	15	11	9	19	14	18	10	13	24	20	17	26	25	22	23	21	27	28
Uganda	1	4	3	11	5	8	2	16	7	9	15	12	6	18	13	19	10	14	20	21	17	26	24	25	22	23	28	27
Zambia	1	4	3	9	5	7	2	12	6	16	13	11	8	18	14	19	10	15	25	20	17	26	23	21	22	24	27	28
**Southern sub-Saharan Africa region**	3	6	2	9	7	10	1	8	4	5	16	12	14	13	21	17	11	15	26	19	22	27	23	18	25	20	28	24
Botswana	3	4	2	8	5	9	1	7	6	14	13	10	20	12	21	15	11	16	24	17	28	26	22	18	25	19	27	23
Eswatini	3	5	6	8	7	10	2	9	4	1	15	11	13	14	19	18	12	16	25	17	22	27	23	20	26	21	28	24
Lesotho	3	5	4	10	8	7	1	9	6	2	14	12	13	15	18	17	11	16	27	19	23	28	21	20	24	22	25	26
Namibia	1	5	2	6	9	15	3	13	4	11	8	14	10	16	17	12	22	7	21	19	20	28	18	24	25	23	26	27
South Africa	4	6	3	8	7	10	1	9	2	5	16	12	14	13	21	18	11	15	25	17	24	26	23	19	28	20	27	22
Zimbabwe	2	5	3	11	7	6	1	8	13	4	17	10	15	14	19	21	9	12	27	24	18	25	23	16	22	20	28	26
**Western sub-Saharan Africa region**	3	4	2	5	9	8	1	11	6	10	16	12	7	13	14	20	18	15	28	19	23	25	22	17	26	24	27	21
Benin	3	2	4	7	9	6	1	10	5	8	15	12	11	13	19	20	14	18	27	17	22	24	23	16	25	26	28	21
Burkina Faso	3	4	2	7	8	5	1	9	6	10	15	11	12	13	18	20	14	17	27	19	21	25	23	16	24	26	28	22
Cameroon	4	2	3	8	7	6	1	9	5	17	16	11	13	10	12	18	14	19	27	20	23	25	22	15	26	24	28	21
Cape Verde	3	2	9	4	10	7	6	8	5	1	16	13	20	11	17	22	12	19	23	15	21	27	24	14	26	25	28	18
Chad	3	2	4	8	9	5	1	10	6	7	16	12	11	14	18	19	13	17	26	20	22	24	23	15	25	27	28	21
Côte d'Ivoire	3	2	4	7	8	6	1	10	5	9	16	12	13	11	19	20	14	18	26	17	23	24	22	15	27	25	28	21

Location	Other malignant neoplasms	Leukaemia	Breast cancer	Brain and central nervous system cancer	Colon and rectum cancer	Stomach cancer	Cervical cancer	Tracheal, bronchus, and lung cancer	Non-Hodgkin lymphoma	Liver cancer	Lip and oral cavity cancer	Ovarian cancer	Hodgkin lymphoma	Pancreatic cancer	Nasopharynx cancer	Testicular cancer	Oesophageal cancer	Malignant skin melanoma	Other pharynx cancer	Kidney cancer	Thyroid cancer	Gallbladder and biliary tract cancer	Larynx cancer	Bladder cancer	Uterine cancer	Multiple myeloma	Mesothelioma	Prostate cancer
The Gambia	2	4	5	7	6	10	3	11	8	1	14	12	9	15	20	13	18	16	28	17	19	23	25	21	24	26	27	22
Ghana	6	5	1	3	7	10	2	9	4	8	20	12	16	11	22	18	15	19	27	14	28	23	21	13	26	24	25	17
Guinea	3	6	4	11	8	5	1	10	13	2	9	12	7	18	19	15	20	14	25	21	17	26	24	16	22	27	28	23
Guinea-Bissau	4	3	2	7	9	5	1	10	6	8	15	11	12	13	18	20	14	17	28	19	21	25	23	16	24	26	27	22
Liberia	3	2	4	8	9	6	1	10	5	7	16	11	13	12	19	20	14	18	27	17	22	25	23	15	26	24	28	21
Mali	4	5	6	8	7	2	1	11	9	3	16	13	10	15	22	19	18	14	26	20	17	25	21	12	24	27	28	23
Mauritania	2	3	4	6	9	8	1	10	5	7	17	11	13	12	20	19	14	18	27	16	22	26	24	15	23	25	28	21
Niger	2	3	5	6	7	4	1	8	9	16	15	11	10	13	21	18	12	17	26	19	28	23	22	14	24	25	27	20
Nigeria	2	4	1	6	8	11	3	9	7	10	17	12	5	14	13	26	21	15	27	16	24	23	18	22	28	20	25	19
São Tomé and Príncipe	1	4	3	12	6	8	2	5	7	10	19	9	23	13	24	17	15	20	28	14	16	18	25	11	22	26	27	21
Senegal	2	4	3	7	8	6	1	9	5	10	16	12	11	13	20	19	14	18	27	17	22	24	23	15	26	25	28	21
Sierra Leone	3	2	4	7	9	6	1	10	5	8	15	11	12	13	18	20	14	17	28	19	22	25	23	16	24	26	27	21
Togo	2	4	3	7	9	6	1	10	5	8	15	11	12	13	19	20	14	18	27	17	22	24	23	16	25	26	28	21

Appendix Figure 8: Cancer ranking for adolescent and young adult cancer (15-39 years) by total DALYs within global setting, Socio-demographic Index (SDI) quintile, super region, region, and country, both sexes combined, 2019. Ranking is by cancer type, within each row. Order of locations is global first, then SDI quintiles from high to low SDI quintile, then alphabetical by hierarchy level (alphabetical by super-region, then alphabetical by region, then alphabetical by country). DALYs=disability-adjusted life-years. GBD 2019 super-regions and regions are defined on appendix pages 58 and 59. GBD=Global Burden of Diseases, Injuries, and Risk Factors Study.

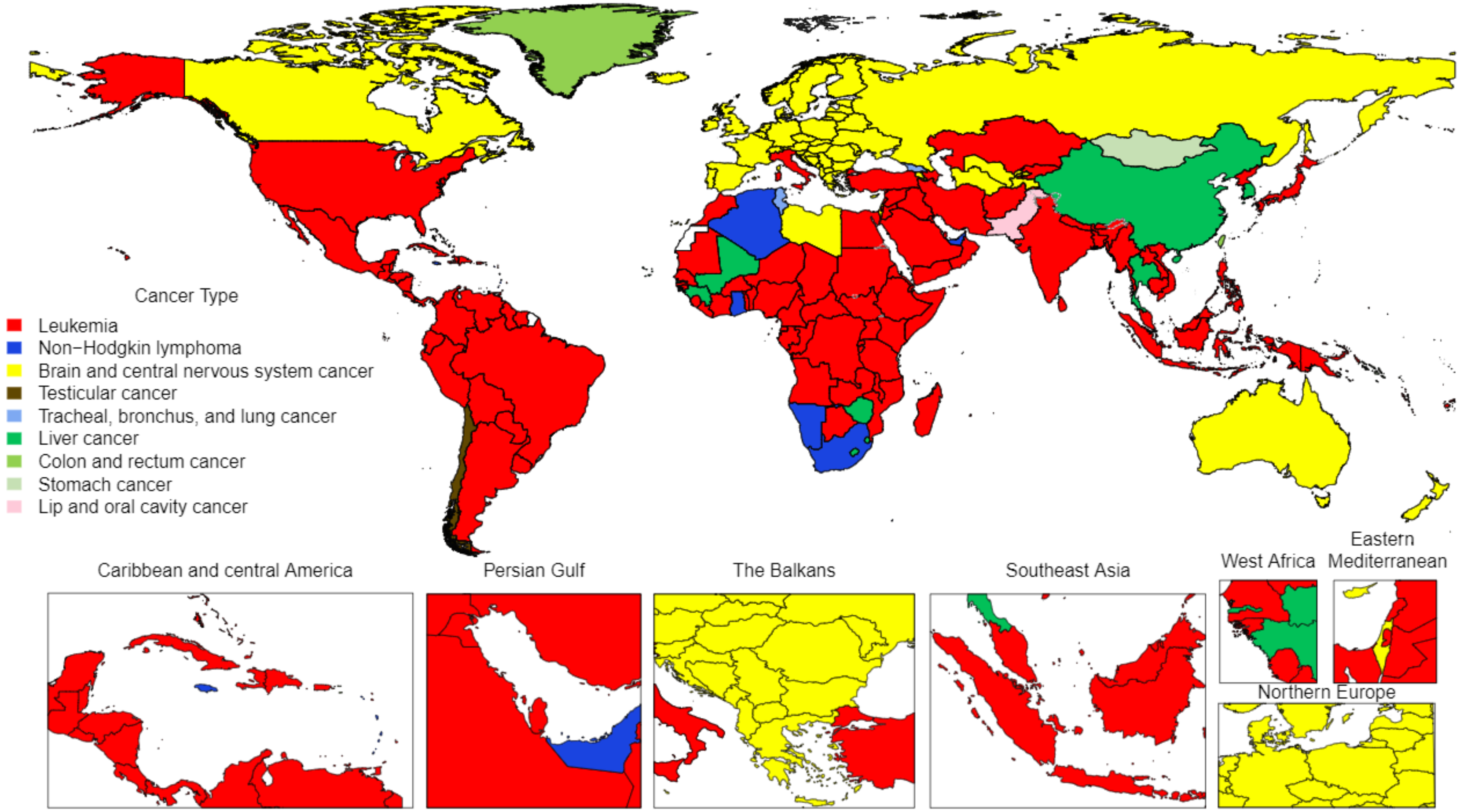

Appendix Figure 9: Map of cancer with the highest DALY burden in adolescent and young adult (15-39 years) males by country, 2019. Other malignant neoplasms, non-melanoma skin cancers, and myelodysplastic/myeloproliferative neoplasms are excluded from this figure. DALYs=disability-adjusted life-years. There are several geographic locations where estimates are not available (e.g., Western Sahara, French Guiana) as they were not modelled locations in the Global Burden of Diseases, Injuries, and Risk Factors 2019 study. Count of countries for which each cancer has the highest DALYs: Leukaemia, 115 countries; Non-Hodgkin lymphoma, 12 countries; Brain and central nervous system cancer, 49 countries; Testicular cancer, 2 countries; Tracheal, bronchus, and lung cancer, 5 countries; Liver cancer, 10 countries; Colon and rectum cancer, 3 countries; Stomach cancer, 7 countries; Lip and oral cavity cancer, 1 country.

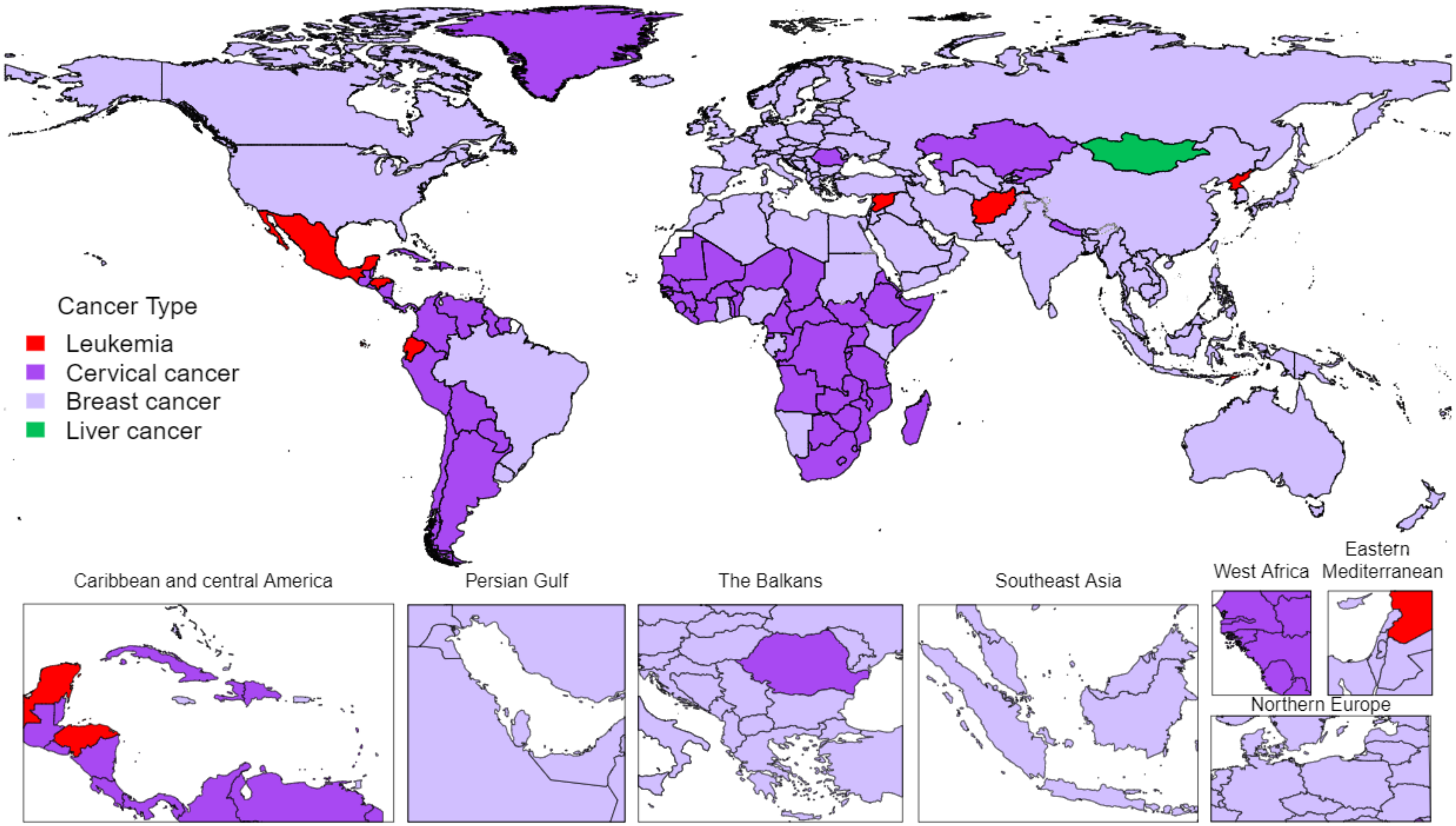

Appendix Figure 10: Map of cancer with the highest DALY burden in adolescent and young adult (15-39 years) females by country, 2019. Other malignant neoplasms, non-melanoma skin cancers, and myelodysplastic/myeloproliferative neoplasms are excluded from this figure. DALYs=disability-adjusted life-years. There are several geographic locations where estimates are not available (e.g., Western Sahara, French Guiana) as they were not modelled locations in the Global Burden of Diseases, Injuries, and Risk Factors 2019 study. Count of countries for which each cancer has the highest DALYs: Leukaemia, 7 countries; Cervical cancer, 70 countries; Breast cancer, 126 countries; Liver cancer, 1 country.

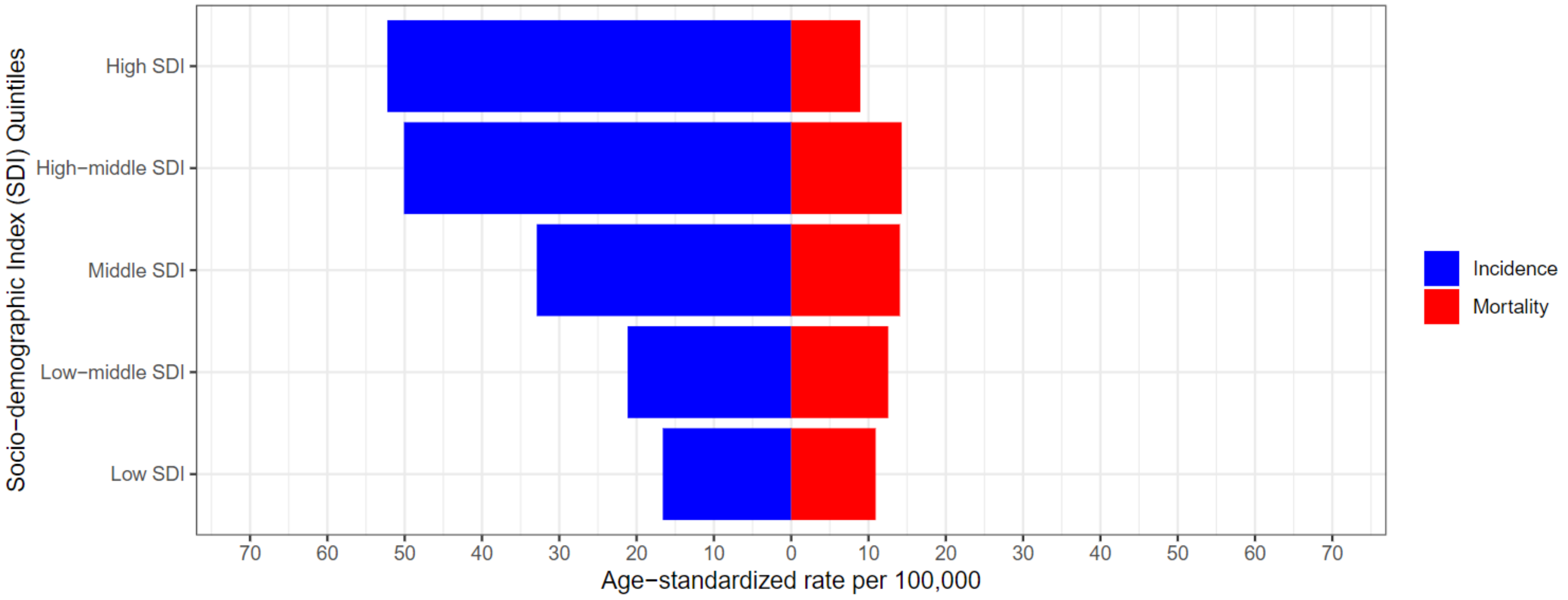

Appendix Figure 11: Age-standardised incidence and mortality rates by Socio-demographic Index (SDI) quintiles for adolescent and young adult (15-39 years) cancers, male, 2019. Rates are expressed per 100,000 person-years. Age-standardisation used the GBD 2019 world standard. GBD=Global Burden of Diseases, Injuries, and Risk Factors 2019 study. Cancers included all malignancies except non-melanoma skin cancers and excluded myelodysplastic/myeloproliferative neoplasms.

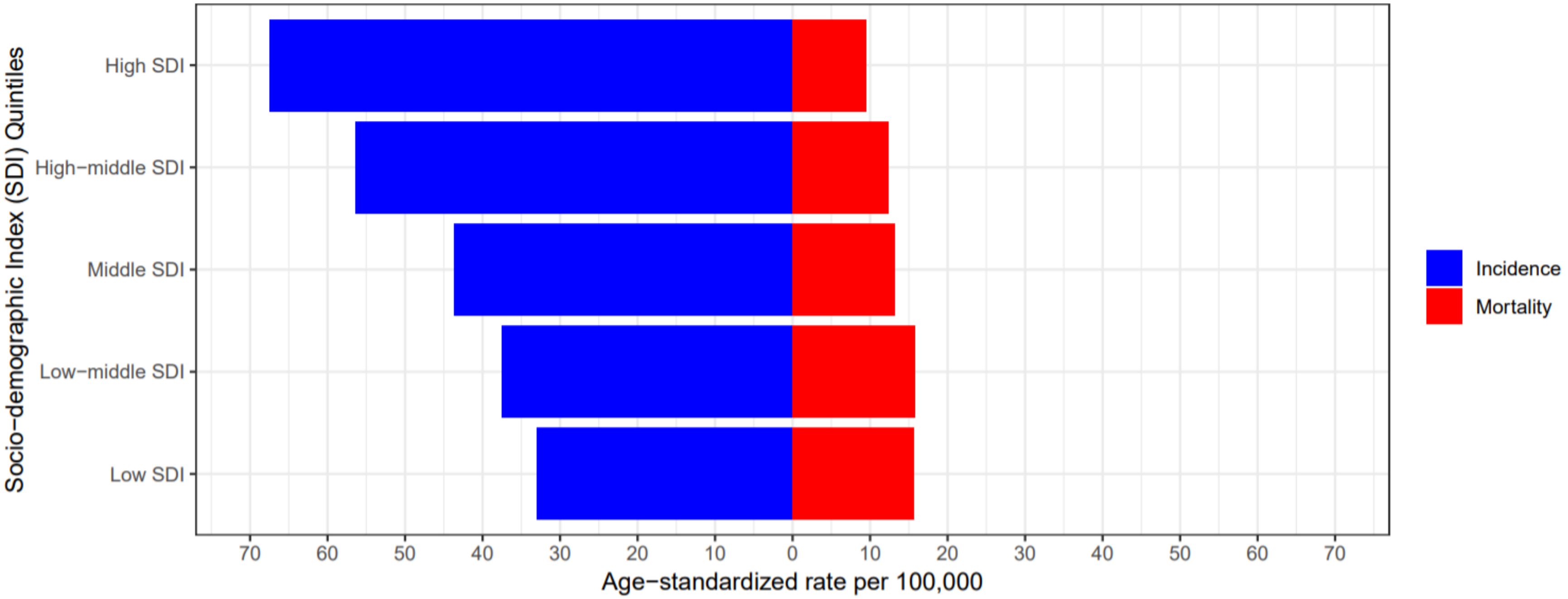

Appendix Figure 12: Age-standardised incidence and mortality rates by Socio-demographic Index (SDI) quintiles for adolescent and young adult (15-39 years) cancers, female, 2019. Rates are expressed per 100,000 person-years. Age-standardisation used the GBD 2019 world standard. GBD=Global Burden of Diseases, Injuries, and Risk Factors 2019 study. Cancers included all malignancies except non-melanoma skin cancers and excluded myelodysplastic/myeloproliferative neoplasms.

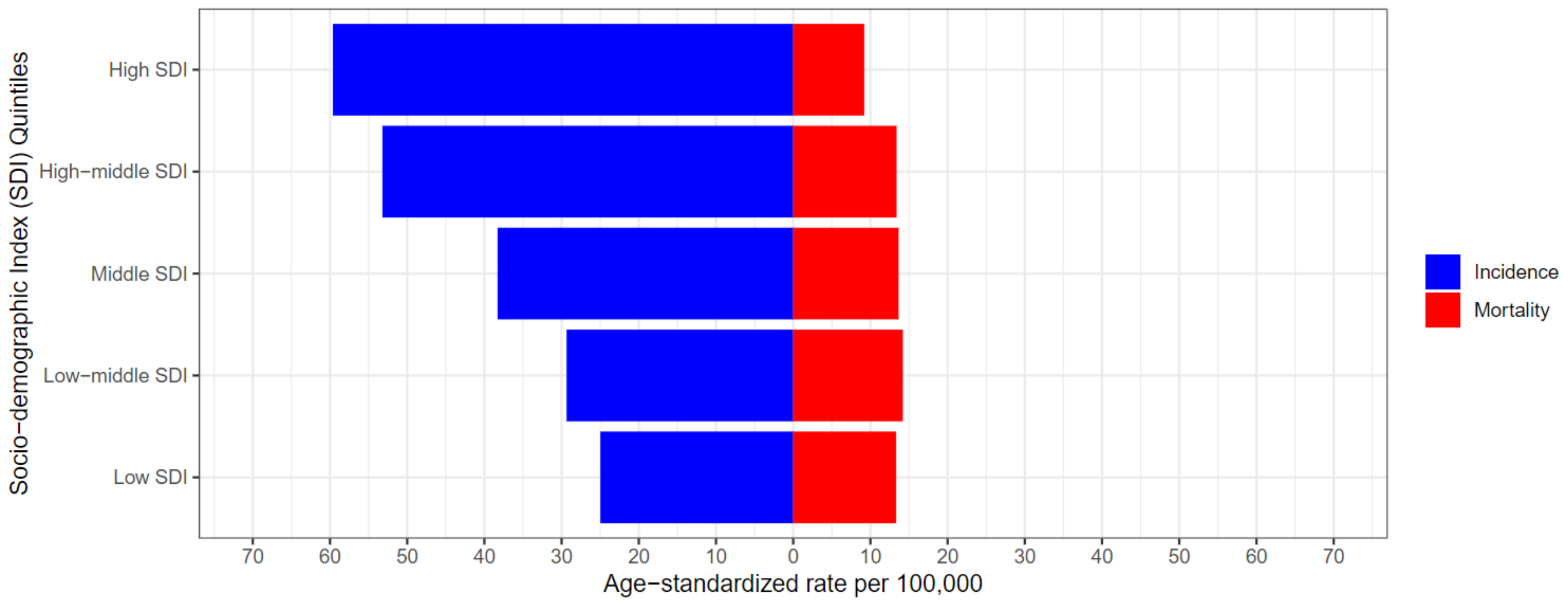

Appendix Figure 13: Age-standardised incidence and mortality rates by Socio-demographic Index (SDI) quintiles for adolescent and young adult (15-39 years) cancers, both sexes combined, 2019. Rates are expressed per 100,000 person-years. Age-standardisation used the GBD 2019 world standard. GBD=Global Burden of Diseases, Injuries, and Risk Factors 2019 study. Cancers included all malignancies except non-melanoma skin cancers and excluded myelodysplastic/myeloproliferative neoplasms.

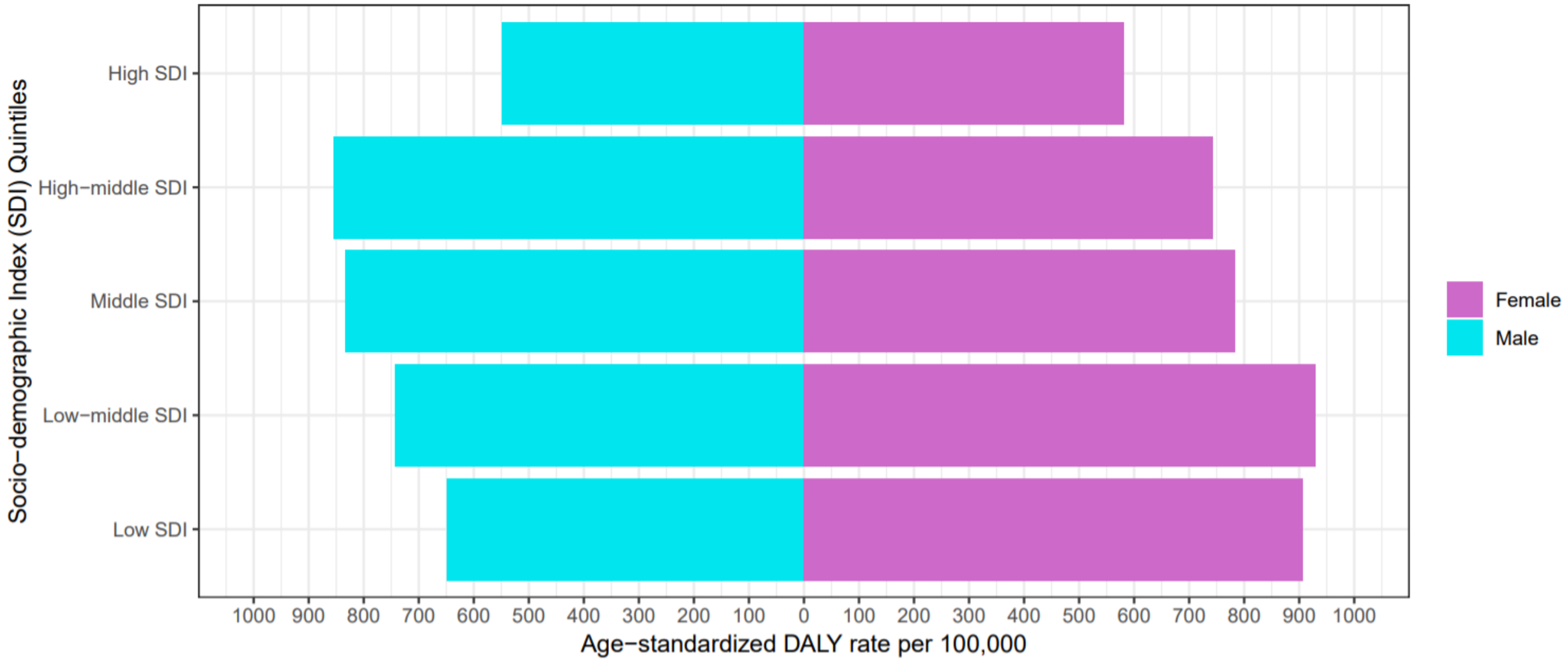

Appendix Figure 14: Age-standardised DALY rates by Socio-demographic Index (SDI) quintiles for adolescent and young adult (15-39 years) cancers, male and female, 2019. Rates are expressed per 100,000 person-years. Age-standardisation used the GBD 2019 world standard. GBD=Global Burden of Diseases, Injuries, and Risk Factors 2019 study. Cancers included all malignancies except non-melanoma skin cancers and excluded myelodysplastic/myeloproliferative neoplasms.

A

**Cause**	**Absolute Deaths (95% UI)**	**Global rank**	**High SDI rank**	**High-middle SDI rank**	**Middle SDI rank**	**Low-middle SDI rank**	**Low SDI rank**
Maternal and neonatal disorders	1 880 000 (1 610 000 - 2 240 000)	1	1	1	1	1	1
Respiratory infections and tuberculosis	785 000 (647 000 - 960 000)	2	7	4	3	2	3
Enteric infections	695 000 (555 000 - 884 000)	3	13	8	4	3	2
Other non-communicable diseases	586 000 (470 000 - 738 000)	4	2	2	2	4	6
Neglected tropical diseases and malaria	428 000 (219 000 - 731 000)	5	19	9	7	6	4
Other infectious diseases	427 000 (309 000 - 593 000)	6	8	7	6	5	5
Unintentional injuries	235 000 (196 000 - 281 000)	7	3	3	5	7	8
HIV/AIDS and sexually transmitted infections	158 000 (101 000 - 252 000)	8	15	10	10	8	7
Nutritional deficiencies	106 000 (83 200 - 135 000)	9	17	16	14	10	9
Transport injuries	99 700 (84 800 - 119 000)	10	5	6	8	11	10
**Childhood cancers**	**98 800 (86 100 - 114 000)**	**11**	**4**	**5**	**9**	**9**	**11**
Digestive diseases	54 600 (43 000 - 69 900)	12	11	14	12	12	13
Self-harm and interpersonal violence	50 800 (45 000 - 56 800)	13	6	11	13	14	12
Cardiovascular diseases	45 600 (38 200 - 55 200)	14	9	12	11	13	14
Neurological disorders	23 000 (19 400 - 27 700)	15	10	13	16	16	16
Diabetes and kidney diseases	23 000 (19 600 - 26 900)	16	14	15	15	15	15
Chronic respiratory diseases	15 100 (12 400 - 18 400)	17	12	17	17	17	17
Skin and subcutaneous diseases	4 550 (2 970 - 5 760)	18	18	18	18	18	18
Musculoskeletal disorders	954 (797 - 1 180)	19	16	19	19	19	19
Mental disorders	4.62 (3.35 - 8.66)	20	20	20	20	20	20
Substance use disorders	0 (0 - 0)	21	21	21	21	21	21

B

**Cause**	**Absolute Deaths (95% UI)**	**Global rank**	**High SDI rank**	**High-middle SDI rank**	**Middle SDI rank**	**Low-middle SDI rank**	**Low SDI rank**
Cardiovascular diseases	18 100 000 (16 600 000 - 19 200 000)	1	1	1	1	1	1

**Adult cancers**	**9 470 000 (8 830 000 - 10 000 000)**	**2**	**2**	**2**	**2**	**2**	**3**
Chronic respiratory diseases	3 900 000 (3 510 000 - 4 220 000)	3	4	3	3	3	4
Diabetes and kidney diseases	2 840 000 (2 620 000 - 3 020 000)	4	5	5	4	5	7
Respiratory infections and tuberculosis	2 580 000 (2 360 000 - 2 780 000)	5	7	7	6	4	2
Digestive diseases	2 260 000 (2 100 000 - 2 390 000)	6	6	6	5	6	6
Neurological disorders	2 150 000 (954 000 - 4 680 000)	7	3	4	7	9	11
Unintentional injuries	1 220 000 (1 050 000 - 1 330 000)	8	8	8	8	8	9
Enteric infections	913 000 (553 000 - 1 500 000)	9	14	14	12	7	5
Transport injuries	675 000 (585 000 - 736 000)	10	12	10	9	10	13
Self-harm and interpersonal violence	596 000 (548 000 - 645 000)	11	10	9	10	12	12
Other non-communicable diseases	468 000 (402 000 - 503 000)	12	9	11	13	13	15
HIV/AIDS and sexually transmitted infections	438 000 (414 000 - 473 000)	13	18	13	11	11	8
Neglected tropical diseases and malaria	226 000 (128 000 - 386 000)	14	19	19	15	15	10
Other infectious diseases	216 000 (198 000 - 239 000)	15	17	17	14	14	14
Substance use disorders	201 000 (185 000 - 211 000)	16	11	12	17	16	18
Nutritional deficiencies	133 000 (120 000 - 147 000)	17	16	18	16	17	16
Musculoskeletal disorders	105 000 (84 900 - 122 000)	18	13	16	18	18	19
Skin and subcutaneous diseases	89 000 (67 100 - 113 000)	19	15	15	19	19	20
Maternal and neonatal disorders	34 900 (30 100 - 40 900)	20	20	20	20	20	17
Mental disorders	114 (104 - 127)	21	21	21	21	21	21

Appendix Figure 15: Ranking of absolute deaths due to cancer in (A) children (0-14 years) and (B) adults (40+ years), for both sexes combined, globally, and by SDI. Disease rank assigned by total absolute deaths globally in 2019 in the (A) pediatric (0–14 years) and (B) adult (40+ years) age groups, with 1 representing the highest rank. Values in parentheses are 95% uncertainty intervals (UIs). Colour intensity is proportional to rank number (from 1 denoted by dark red to 21 denoted by dark green. Cancers comprise all malignant neoplasms, excluding non-melanoma skin cancers. Other non-communicable diseases comprise congenital birth defects; urinary diseases and male infertility; gynaecological diseases; haemoglobinopathies and haemolytic anaemias; endocrine, metabolic, blood, and immune disorders; and oral disorders. Other infectious diseases comprise meningitis, encephalitis, diphtheria, whooping cough, tetanus, measles, varicella and herpes zoster, acute hepatitis, and other unspecified infectious diseases. SDI=Socio-demographic Index, represented in quintiles.

Appendix Table 15: Global absolute DALYs for each adolescent and young adult cancer type by age group, in 2019, for both sexes combined. Other malignant neoplasms comprise all malignancies without a separate GBD cause category listed; this category does not include non-melanoma skin cancers and myelodysplastic or myeloproliferative neoplasms, which are separate GBD cause categories not included in this analysis. Other leukaemia included leukaemias not otherwise specified. Some cancers do not have estimates in this table for the 15-19 year age group due to age restrictions for these causes in GBD estimation (these are modeled starting at age 20 years, for more information see Appendix Table 3). DALY=disability-adjusted life-year. GBD=Global Burden of Diseases, Injuries, and Risk Factors Study. NA=no estimate available as these causes are sex-specific.

Cancer	Age group	Global DALYs, both sexes combined (95% UI)	Global DALYs, males (95% UI)	Global DALYs, females (95% UI)
Acute lymphoid leukaemia	15 to 19	259 000 (215 000 to 291 000)	167 000 (132 000 to 194 000)	92 700 (69 500 to 111 000)
Acute lymphoid leukaemia	20 to 24	172 000 (141 000 to 192 000)	111 000 (85 900 to 126 000)	61 100 (43 900 to 73 000)
Acute lymphoid leukaemia	25 to 29	132 000 (106 000 to 148 000)	78 100 (58 900 to 88 600)	54 400 (39 700 to 67 400)
Acute lymphoid leukaemia	30 to 34	107 000 (81 100 to 120 000)	66 900 (48 800 to 76 800)	39 800 (26 900 to 47 600)
Acute lymphoid leukaemia	35 to 39	95 100 (74 800 to 106 000)	56 400 (42 600 to 64 300)	38 700 (27 300 to 46 500)
Acute myeloid leukaemia	15 to 19	166 000 (147 000 to 202 000)	89 400 (72 800 to 121 000)	76 200 (64 200 to 90 800)
Acute myeloid leukaemia	20 to 24	143 000 (126 000 to 166 000)	75 200 (61 800 to 95 600)	67 400 (57 000 to 78 400)
Acute myeloid leukaemia	25 to 29	146 000 (131 000 to 164 000)	77 900 (63 000 to 97 900)	67 700 (55 100 to 77 700)
Acute myeloid leukaemia	30 to 34	143 000 (129 000 to 160 000)	77 500 (62 500 to 93 700)	65 800 (55 700 to 76 500)
Acute myeloid leukaemia	35 to 39	150 000 (134 000 to 172 000)	80 800 (64 800 to 101 000)	69 600 (59 300 to 80 500)
Bladder cancer	15 to 19	7 120 (6 140 to 8 690)	4 260 (3 550 to 5 560)	2 860 (2 540 to 3 210)
Bladder cancer	20 to 24	10 100 (8 840 to 11 600)	6 670 (5 600 to 7 920)	3 440 (3 020 to 3 860)
Bladder cancer	25 to 29	15 500 (13 700 to 17 700)	9 690 (8 310 to 11 500)	5 840 (5 100 to 6 820)
Bladder cancer	30 to 34	34 300 (30 600 to 38 500)	24 800 (21 700 to 28 500)	9 490 (8 380 to 10 700)
Bladder cancer	35 to 39	57 400 (51 600 to 64 700)	40 900 (35 800 to 47 300)	16 500 (14 600 to 18 600)
Brain and central nervous system cancer	15 to 19	257 000 (199 000 to 292 000)	146 000 (104 000 to 176 000)	110 000 (83 600 to 128 000)

Brain and central nervous system cancer	20 to 24	261 000<br>(203 000 to 292 000)	148 000<br>(105 000 to 172 000)	113 000<br>(86 300 to 131 000)
Brain and central nervous system cancer	25 to 29	331 000<br>(258 000 to 369 000)	190 000<br>(138 000 to 219 000)	140 000<br>(101 000 to 162 000)
Brain and central nervous system cancer	30 to 34	422 000<br>(333 000 to 468 000)	252 000<br>(182 000 to 289 000)	170 000<br>(124 000 to 197 000)
Brain and central nervous system cancer	35 to 39	476 000<br>(372 000 to 532 000)	288 000<br>(216 000 to 335 000)	188 000<br>(137 000 to 216 000)
Breast cancer	15 to 19	53 100<br>(44 100 to 63 600)	2 790<br>(2 460 to 3 160)	50 300<br>(41 400 to 60 700)
Breast cancer	20 to 24	117 000<br>(100 000 to 134 000)	2 780<br>(2 500 to 3 160)	114 000<br>(97 800 to 131 000)
Breast cancer	25 to 29	294 000<br>(260 000 to 328 000)	3 000<br>(2 700 to 3 350)	291 000<br>(257 000 to 325 000)
Breast cancer	30 to 34	723 000<br>(651 000 to 791 000)	4 370<br>(3 790 to 5 070)	718 000<br>(647 000 to 787 000)
Breast cancer	35 to 39	1 300 000<br>(1 190 000 to 1 430 000)	7 960<br>(6 740 to 9 480)	1 300 000<br>(1 180 000 to 1 420 000)
Cervical cancer	15 to 19	17 600<br>(13 300 to 20 500)	NA	17 600<br>(13 300 to 20 500)
Cervical cancer	20 to 24	78 900<br>(63 300 to 93 400)	NA	78 900<br>(63 300 to 93 400)
Cervical cancer	25 to 29	221 000<br>(182 000 to 256 000)	NA	221 000<br>(182 000 to 256 000)
Cervical cancer	30 to 34	484 000<br>(405 000 to 555 000)	NA	484 000<br>(405 000 to 555 000)
Cervical cancer	35 to 39	759 000<br>(652 000 to 875 000)	NA	759 000<br>(652 000 to 875 000)
Chronic lymphoid leukaemia	20 to 24	14 500<br>(12 100 to 16 900)	8 930<br>(6 530 to 11 200)	5 530<br>(4 450 to 6 790)
Chronic lymphoid leukaemia	25 to 29	13 800<br>(11 800 to 16 000)	7 770<br>(5 790 to 9 420)	6 010<br>(4 670 to 7 500)
Chronic lymphoid leukaemia	30 to 34	15 600<br>(13 000 to 17 800)	8 920<br>(6 380 to 10 900)	6 650<br>(5 020 to 8 470)
Chronic lymphoid leukaemia	35 to 39	17 600<br>(15 200 to 20 000)	9 320<br>(7 230 to 11 400)	8 250<br>(6 360 to 10 300)
Chronic myeloid leukaemia	15 to 19	29 900<br>(25 100 to 35 400)	15 800<br>(12 200 to 19 900)	14 100<br>(10 700 to 18 000)
Chronic myeloid leukaemia	20 to 24	47 200<br>(40 400 to 55 400)	26 800<br>(21 000 to 33 400)	20 400<br>(16 200 to 25 300)
Chronic myeloid leukaemia	25 to 29	67 200<br>(58 400 to 79 100)	42 000<br>(34 300 to 52 400)	25 300<br>(19 700 to 31 000)

Chronic myeloid leukaemia	30 to 34	72 000<br>(63 200 to 82 900)	43 100<br>(35 300 to 52 900)	28 900<br>(23 700 to 35 200)
Chronic myeloid leukaemia	35 to 39	78 600<br>(68 600 to 90 100)	46 300<br>(38 200 to 57 000)	32 300<br>(26 300 to 38 900)
Colon and rectum cancer	15 to 19	57 700<br>(52 400 to 63 300)	35 000<br>(30 800 to 39 500)	22 700<br>(20 400 to 25 200)
Colon and rectum cancer	20 to 24	128 000<br>(118 000 to 140 000)	72 700<br>(65 000 to 81 700)	55 500<br>(49 500 to 62 100)
Colon and rectum cancer	25 to 29	239 000<br>(222 000 to 258 000)	140 000<br>(128 000 to 154 000)	98 600<br>(88 500 to 109 000)
Colon and rectum cancer	30 to 34	499 000<br>(462 000 to 540 000)	301 000<br>(273 000 to 334 000)	198 000<br>(179 000 to 218 000)
Colon and rectum cancer	35 to 39	710 000<br>(654 000 to 770 000)	424 000<br>(384 000 to 467 000)	285 000<br>(258 000 to 315 000)
Gallbladder and biliary tract cancer	20 to 24	9 260<br>(7 500 to 10 500)	3 550<br>(2 960 to 3 900)	5 710<br>(4 170 to 6 840)
Gallbladder and biliary tract cancer	25 to 29	17 000<br>(13 900 to 19 300)	6 790<br>(5 670 to 7 790)	10 200<br>(7 580 to 12 200)
Gallbladder and biliary tract cancer	30 to 34	38 100<br>(31 900 to 42 500)	18 600<br>(15 400 to 21 200)	19 500<br>(14 800 to 22 800)
Gallbladder and biliary tract cancer	35 to 39	68 300<br>(58 400 to 76 000)	32 400<br>(27 400 to 36 800)	35 900<br>(27 900 to 41 700)
Hodgkin lymphoma	15 to 19	80 700<br>(68 200 to 97 400)	46 100<br>(35 600 to 60 700)	34 600<br>(27 500 to 45 600)
Hodgkin lymphoma	20 to 24	110 000<br>(93 200 to 130 000)	61 000<br>(48 000 to 78 400)	48 900<br>(37 300 to 62 300)
Hodgkin lymphoma	25 to 29	122 000<br>(103 000 to 145 000)	70 000<br>(56 200 to 91 600)	52 400<br>(39 300 to 66 800)
Hodgkin lymphoma	30 to 34	106 000<br>(88 100 to 125 000)	65 800<br>(51 300 to 81 700)	40 100<br>(29 800 to 49 700)
Hodgkin lymphoma	35 to 39	89 300<br>(73 600 to 106 000)	59 400<br>(46 800 to 75 600)	29 900<br>(22 200 to 35 800)
Kidney cancer	15 to 19	17 900<br>(16 100 to 19 700)	9 420<br>(8 280 to 10 800)	8 440<br>(7 600 to 9 350)
Kidney cancer	20 to 24	25 200<br>(22 800 to 27 700)	13 800<br>(12 200 to 15 700)	11 400<br>(10 200 to 12 600)
Kidney cancer	25 to 29	38 000<br>(34 700 to 41 800)	23 000<br>(20 700 to 25 700)	15 000<br>(13 500 to 16 600)
Kidney cancer	30 to 34	61 000<br>(55 800 to 67 500)	39 800<br>(35 400 to 44 700)	21 200<br>(19 100 to 23 400)
Kidney cancer	35 to 39	97 100<br>(89 000 to 107 000)	66 600<br>(60 000 to 74 400)	30 600<br>(27 500 to 33 800)
Larynx cancer	20 to 24	11 200<br>(10 100 to 12 300)	6 140<br>(5 490 to 6 900)	5 020<br>(4 330 to 5 760)

Larynx cancer	25 to 29	15 000<br>(13 700 to 16 600)	7 950<br>(7 200 to 8 840)	7 090<br>(6 150 to 8 140)
Larynx cancer	30 to 34	31 500<br>(29 000 to 34 600)	22 000<br>(19 900 to 24 700)	9 510<br>(8 290 to 11 000)
Larynx cancer	35 to 39	70 500<br>(63 400 to 78 300)	54 700<br>(48 500 to 62 100)	15 700<br>(13 500 to 18 100)
Lip and oral cavity cancer	15 to 19	32 600<br>(27 700 to 39 100)	18 000<br>(14 000 to 23 000)	14 600<br>(12 100 to 17 500)
Lip and oral cavity cancer	20 to 24	57 900<br>(50 600 to 67 100)	30 200<br>(24 400 to 37 300)	27 700<br>(22 600 to 33 700)
Lip and oral cavity cancer	25 to 29	88 800<br>(77 000 to 103 000)	51 600<br>(42 400 to 62 000)	37 200<br>(30 700 to 44 800)
Lip and oral cavity cancer	30 to 34	154 000<br>(135 000 to 175 000)	96 000<br>(80 200 to 113 000)	58 500<br>(49 700 to 69 100)
Lip and oral cavity cancer	35 to 39	247 000<br>(217 000 to 279 000)	172 000<br>(145 000 to 199 000)	74 700<br>(65 100 to 86 200)
Liver cancer	15 to 19	50 400<br>(44 700 to 57 000)	30 000<br>(25 900 to 35 200)	20 400<br>(17 000 to 24 300)
Liver cancer	20 to 24	78 400<br>(70 200 to 86 700)	49 800<br>(43 500 to 57 200)	28 500<br>(23 800 to 33 500)
Liver cancer	25 to 29	144 000<br>(130 000 to 159 000)	103 000<br>(91 100 to 117 000)	41 000<br>(34 900 to 47 800)
Liver cancer	30 to 34	306 000<br>(272 000 to 344 000)	240 000<br>(208 000 to 276 000)	66 700<br>(56 900 to 77 200)
Liver cancer	35 to 39	467 000<br>(408 000 to 530 000)	372 000<br>(313 000 to 436 000)	95 500<br>(82 800 to 110 000)
Malignant skin melanoma	15 to 19	12 800<br>(10 700 to 16 400)	6 580<br>(5 040 to 9 120)	6 240<br>(4 930 to 8 750)
Malignant skin melanoma	20 to 24	25 400<br>(21 000 to 32 200)	12 100<br>(9 050 to 16 500)	13 300<br>(10 300 to 17 900)
Malignant skin melanoma	25 to 29	45 300<br>(37 600 to 56 200)	22 600<br>(16 800 to 30 100)	22 700<br>(17 900 to 28 800)
Malignant skin melanoma	30 to 34	74 300<br>(60 900 to 91 200)	40 200<br>(29 400 to 52 800)	34 100<br>(26 200 to 42 500)
Malignant skin melanoma	35 to 39	101 000<br>(83 300 to 124 000)	55 000<br>(40 100 to 73 200)	46 300<br>(36 300 to 57 800)
Mesothelioma	20 to 24	7 370<br>(5 440 to 9 340)	3 760<br>(3 090 to 4 440)	3 610<br>(1 960 to 5 450)
Mesothelioma	25 to 29	11 900<br>(8 770 to 15 700)	5 920<br>(5 030 to 6 960)	5 950<br>(3 300 to 9 490)
Mesothelioma	30 to 34	13 900<br>(11 400 to 16 300)	7 830<br>(6 890 to 8 880)	6 030<br>(3 860 to 8 070)
Mesothelioma	35 to 39	23 300<br>(18 200 to 31 200)	12 300<br>(10 500 to 14 500)	11 000<br>(6 680 to 18 700)

Multiple myeloma	20 to 24	12 300<br>(8 460 to 14 700)	7 690<br>(5 100 to 9 490)	4 590<br>(2 920 to 5 690)
Multiple myeloma	25 to 29	14 000<br>(9 450 to 16 600)	9 000<br>(5 920 to 11 000)	4 970<br>(3 250 to 6 120)
Multiple myeloma	30 to 34	25 900<br>(19 900 to 29 100)	16 400<br>(12 300 to 19 500)	9 480<br>(6 630 to 11 300)
Multiple myeloma	35 to 39	43 400<br>(35 100 to 48 800)	26 900<br>(21 100 to 31 700)	16 600<br>(12 400 to 19 200)
Nasopharynx cancer	15 to 19	29 500<br>(26 500 to 32 900)	19 300<br>(16 900 to 22 200)	10 200<br>(8 920 to 11 700)
Nasopharynx cancer	20 to 24	40 600<br>(36 800 to 44 900)	26 500<br>(23 500 to 30 200)	14 000<br>(12 200 to 16 300)
Nasopharynx cancer	25 to 29	54 100<br>(49 000 to 59 700)	35 200<br>(31 100 to 39 700)	18 900<br>(16 400 to 21 600)
Nasopharynx cancer	30 to 34	95 600<br>(86 900 to 106 000)	66 000<br>(58 800 to 73 700)	29 600<br>(26 000 to 33 900)
Nasopharynx cancer	35 to 39	143 000<br>(130 000 to 158 000)	102 000<br>(90 700 to 115 000)	41 000<br>(35 700 to 46 500)
Non-Hodgkin lymphoma	15 to 19	208 000<br>(188 000 to 228 000)	133 000<br>(119 000 to 148 000)	75 000<br>(67 100 to 83 200)
Non-Hodgkin lymphoma	20 to 24	248 000<br>(227 000 to 273 000)	158 000<br>(143 000 to 176 000)	90 000<br>(79 600 to 100 000)
Non-Hodgkin lymphoma	25 to 29	250 000<br>(231 000 to 272 000)	152 000<br>(140 000 to 167 000)	97 900<br>(86 900 to 109 000)
Non-Hodgkin lymphoma	30 to 34	271 000<br>(252 000 to 293 000)	173 000<br>(160 000 to 189 000)	97 500<br>(87 500 to 108 000)
Non-Hodgkin lymphoma	35 to 39	299 000<br>(278 000 to 326 000)	193 000<br>(178 000 to 211 000)	107 000<br>(96 200 to 118 000)
Oesophageal cancer	20 to 24	24 200<br>(20 900 to 27 500)	12 600<br>(11 000 to 14 400)	11 500<br>(9 450 to 14 200)
Oesophageal cancer	25 to 29	41 900<br>(37 100 to 47 800)	22 000<br>(19 200 to 25 300)	19 900<br>(16 300 to 24 100)
Oesophageal cancer	30 to 34	91 700<br>(82 000 to 102 000)	60 400<br>(52 800 to 69 500)	31 200<br>(26 600 to 36 500)
Oesophageal cancer	35 to 39	186 000<br>(167 000 to 209 000)	133 000<br>(117 000 to 153 000)	53 100<br>(45 900 to 61 000)
Other leukaemia	15 to 19	184 000<br>(149 000 to 218 000)	108 000<br>(83 800 to 128 000)	76 600<br>(56 300 to 97 100)
Other leukaemia	20 to 24	203 000<br>(167 000 to 235 000)	121 000<br>(93 100 to 144 000)	82 700<br>(64 300 to 102 000)
Other leukaemia	25 to 29	188 000<br>(154 000 to 213 000)	110 000<br>(83 900 to 129 000)	78 000<br>(58 900 to 96 400)
Other leukaemia	30 to 34	194 000<br>(158 000 to 222 000)	113 000<br>(85 200 to 133 000)	81 100<br>(62 100 to 98 400)

Other leukaemia	35 to 39	179 000<br>(150 000 to 211 000)	101 000<br>(79 500 to 121 000)	77 900<br>(61 500 to 96 900)
Other malignant neoplasms	15 to 19	732 000<br>(651 000 to 811 000)	429 000<br>(362 000 to 487 000)	303 000<br>(266 000 to 339 000)
Other malignant neoplasms	20 to 24	716 000<br>(640 000 to 793 000)	435 000<br>(375 000 to 490 000)	281 000<br>(248 000 to 320 000)
Other malignant neoplasms	25 to 29	620 000<br>(561 000 to 685 000)	349 000<br>(305 000 to 392 000)	272 000<br>(240 000 to 309 000)
Other malignant neoplasms	30 to 34	569 000<br>(514 000 to 620 000)	330 000<br>(287 000 to 369 000)	238 000<br>(213 000 to 266 000)
Other malignant neoplasms	35 to 39	593 000<br>(535 000 to 649 000)	342 000<br>(291 000 to 385 000)	252 000<br>(226 000 to 281 000)
Other pharynx cancer	20 to 24	28 900<br>(23 700 to 34 900)	15 500<br>(11 700 to 20 900)	13 500<br>(10 000 to 17 300)
Other pharynx cancer	25 to 29	36 600<br>(30 900 to 42 300)	18 800<br>(14 900 to 23 200)	17 800<br>(13 800 to 22 500)
Other pharynx cancer	30 to 34	62 500<br>(53 400 to 71 300)	40 800<br>(33 900 to 49 100)	21 700<br>(17 100 to 26 800)
Other pharynx cancer	35 to 39	117 000<br>(102 000 to 133 000)	82 100<br>(67 900 to 96 400)	35 000<br>(28 000 to 43 600)
Ovarian cancer	15 to 19	40 600<br>(33 200 to 47 500)	NA	40 600<br>(33 200 to 47 500)
Ovarian cancer	20 to 24	65 700<br>(53 300 to 77 300)	NA	65 700<br>(53 300 to 77 300)
Ovarian cancer	25 to 29	89 100<br>(71 700 to 103 000)	NA	89 100<br>(71 700 to 103 000)
Ovarian cancer	30 to 34	133 000<br>(112 000 to 153 000)	NA	133 000<br>(112 000 to 153 000)
Ovarian cancer	35 to 39	200 000<br>(168 000 to 231 000)	NA	200 000<br>(168 000 to 231 000)
Pancreatic cancer	15 to 19	10 300<br>(9 300 to 11 500)	6 090<br>(5 350 to 6 960)	4 200<br>(3 790 to 4 590)
Pancreatic cancer	20 to 24	21 600<br>(19 700 to 24 200)	13 100<br>(11 700 to 15 000)	8 540<br>(7 640 to 9 530)
Pancreatic cancer	25 to 29	45 900<br>(42 000 to 50 300)	26 800<br>(24 100 to 30 200)	19 100<br>(17 000 to 21 300)
Pancreatic cancer	30 to 34	122 000<br>(112 000 to 135 000)	82 400<br>(74 100 to 93 000)	39 600<br>(35 600 to 43 600)
Pancreatic cancer	35 to 39	221 000<br>(202 000 to 244 000)	148 000<br>(133 000 to 166 000)	72 900<br>(65 600 to 80 300)
Prostate cancer	20 to 24	8 540<br>(7 450 to 10 800)	8 540<br>(7 450 to 10 800)	NA
Prostate cancer	25 to 29	13 100<br>(11 500 to 16 600)	13 100<br>(11 500 to 16 600)	NA

Prostate cancer	30 to 34	14 800<br>(12 700 to 17 800)	14 800<br>(12 700 to 17 800)	NA
Prostate cancer	35 to 39	17 900<br>(15 100 to 21 300)	17 900<br>(15 100 to 21 300)	NA
Stomach cancer	15 to 19	47 600<br>(42 800 to 53 200)	23 600<br>(20 800 to 27 200)	24 000<br>(20 900 to 27 500)
Stomach cancer	20 to 24	118 000<br>(108 000 to 131 000)	55 900<br>(50 200 to 62 600)	62 600<br>(54 800 to 71 200)
Stomach cancer	25 to 29	223 000<br>(204 000 to 243 000)	108 000<br>(99 100 to 120 000)	115 000<br>(102 000 to 130 000)
Stomach cancer	30 to 34	475 000<br>(438 000 to 515 000)	259 000<br>(236 000 to 286 000)	217 000<br>(192 000 to 242 000)
Stomach cancer	35 to 39	710 000<br>(654 000 to 771 000)	396 000<br>(357 000 to 436 000)	314 000<br>(281 000 to 348 000)
Testicular cancer	15 to 19	30 400<br>(26 700 to 34 300)	30 400<br>(26 700 to 34 300)	NA
Testicular cancer	20 to 24	73 300<br>(65 900 to 81 500)	73 300<br>(65 900 to 81 500)	NA
Testicular cancer	25 to 29	94 200<br>(85 900 to 104 000)	94 200<br>(85 900 to 104 000)	NA
Testicular cancer	30 to 34	86 500<br>(78 800 to 96 200)	86 500<br>(78 800 to 96 200)	NA
Testicular cancer	35 to 39	64 600<br>(58 600 to 71 200)	64 600<br>(58 600 to 71 200)	NA
Thyroid cancer	15 to 19	15 700<br>(13 600 to 18 000)	5 390<br>(4 680 to 6 090)	10 300<br>(8 440 to 12 500)
Thyroid cancer	20 to 24	31 200<br>(26 100 to 37 000)	10 200<br>(8 900 to 11 600)	21 000<br>(16 100 to 26 900)
Thyroid cancer	25 to 29	42 000<br>(35 300 to 48 900)	15 000<br>(13 100 to 17 000)	27 000<br>(21 000 to 33 600)
Thyroid cancer	30 to 34	45 800<br>(39 500 to 51 900)	16 900<br>(14 900 to 19 000)	28 900<br>(23 200 to 34 400)
Thyroid cancer	35 to 39	56 300<br>(49 500 to 63 000)	21 700<br>(19 000 to 24 400)	34 700<br>(28 000 to 40 100)
Tracheal, bronchus, and lung cancer	15 to 19	53 000<br>(47 700 to 58 700)	33 300<br>(28 900 to 37 900)	19 700<br>(17 700 to 21 600)
Tracheal, bronchus, and lung cancer	20 to 24	95 300<br>(86 600 to 105 000)	57 600<br>(50 700 to 65 500)	37 700<br>(33 300 to 42 300)
Tracheal, bronchus, and lung cancer	25 to 29	174 000<br>(157 000 to 191 000)	105 000<br>(92 100 to 117 000)	69 300<br>(61 300 to 78 500)
Tracheal, bronchus, and lung cancer	30 to 34	388 000<br>(353 000 to 424 000)	238 000<br>(212 000 to 266 000)	150 000<br>(133 000 to 168 000)
Tracheal, bronchus, and lung cancer	35 to 39	676 000<br>(618 000 to 739 000)	422 000<br>(378 000 to 473 000)	254 000<br>(227 000 to 284 000)

Uterine cancer	20 to 24	7 320<br>(4 960 to 8 420)	NA	7 320<br>(4 960 to 8 420)
Uterine cancer	25 to 29	16 100<br>(11 900 to 18 400)	NA	16 100<br>(11 900 to 18 400)
Uterine cancer	30 to 34	31 900<br>(23 900 to 36 400)	NA	31 900<br>(23 900 to 36 400)
Uterine cancer	35 to 39	55 000<br>(43 100 to 61 800)	NA	55 000<br>(43 100 to 61 800)

Additional Results Tables and Figures: Adolescent and young adult cancers analysis reassessed using the age range 15 to 29 years

In order to align with different standard definitions for the upper age limit of "Adolescent and young adult", we have also provided some supplementary GBD 2019 AYA Cancer results below focusing on the narrower age range of 15-29 years, as this is the standard age range considered to comprise "AYA" in some countries.[5–7]

Appendix Table 16: Adolescent and young adult cancers burden globally, by SDI quintile, and by cancer in 2019, ages 15 to 29, both sexes combined.

Location & Cause	Incidence, thousands (95% UI)	Age-standardised Incidence rate, per 100,000 (95% UI)	Mortality, thousands (95% UI)	Age-standardised Mortality rate, per 100,000 (95% UI)	YLLs, thousands (95% UI)	Age-standardised YLL rate, per 100,000 (95% UI)	YLDs, thousands (95% UI)	Age-standardised YLD rate, per 100,000 (95% UI)	DALYs, thousands (95% UI)	Age-standardised DALYs rate, per 100,000 (95% UI)
Global Countries, Total AYA Cancers	427 (396 - 458)	23.2 (21.6 - 24.9)	137 (127 - 148)	7.5 (6.9 - 8.1)	9 000 (8 340 - 9 720)	490.8 (454.5 -529.5)	223 (158 - 298)	12.1 (8.6 - 16.2)	9 230 (8 550 - 9 930)	502.9 (465.7 - 541.2)
High SDI Countries, Total AYA Cancers	72.6 (65.9 - 80.3)	37.6 (34.2 - 41.5)	9.58 (9.17 - 9.91)	5.0 (4.8 - 5.2)	624 (597 - 646)	326.5 (311.9 - 338.5)	41.1 (28.4 - 56.0)	21.3 (14.7 - 29.0)	665 (636 - 695)	347.8 (332.6 - 363.2)
High-middle SDI Countries, Total AYA Cancers	93.8 (86.3 - 102)	32.0 (29.5 - 34.7)	21.5 (20.0 - 23.0)	7.3 (6.8 - 7.9)	1 400 (1 300 - 1 500)	481.6 (448.2 - 515.0)	51.5 (35.9 - 69.7)	17.6 (12.3 - 23.8)	1 450 (1 350 - 1 550)	499.2 (464.1 - 532.3)
Middle SDI Countries, Total AYA Cancers	130 (120 - 141)	22.9 (21.1 - 24.8)	44.3 (41.0 - 47.8)	7.8 (7.2 - 8.4)	2 900 (2 680 - 3 130)	510.9 (473.0 - 550.9)	68.2 (48.2 - 91.4)	12.0 (8.5 - 16.1)	2 970 (2 750 - 3 200)	522.9 (484.6 - 564.0)
Low-middle SDI Countries, Total AYA Cancers	86.8 (78.4 – 95.1)	18.1 (16.4 - 19.9)	39.5 (35.8 - 43.3)	8.3 (7.5 - 9.1)	2 600 (2 350 - 2 840)	542.2 (491.5 - 594.1)	41.5 (29.3 - 55.9)	8.7 (6.1 - 11.7)	2 640 (2 390 - 2 880)	550.8 (499.6 - 602.7)
Low SDI Countries, Total AYA Cancers	43.7 (37.4 – 50.4)	14.4 (12.3 - 16.7)	22.5 (19.4 - 25.8)	7.4 (6.4 - 8.5)	1 480 (1 280 - 1 700)	487.7 (421.2 - 558.3)	20.2 (13.7 - 27.9)	6.7 (4.5 - 9.2)	1 500 (1 300 - 1 720)	494.4 (426.6 - 565.6)
Global Acute lymphoid leukaemia	22.8 (19.1 - 25.4)	1.2 (1.1 - 1.4)	8.16 (6.83 - 9.04)	0.4 (0.4 - 0.5)	550 (460 - 610)	30.4 (25.4 - 33.7)	13.5 (9.27 - 18.6)	0.7 (0.5 - 1.0)	564 (472 - 625)	31.1 (26.0 - 34.5)
Global Acute myeloid leukaemia	12.3 (11.0 - 14.2)	0.7 (0.6 - 0.8)	6.76 (6.06 - 7.88)	0.4 (0.3 - 0.4)	450 (402 - 524)	24.7 (22.1 - 28.7)	4.08 (2.85 - 5.55)	0.2 (0.2 - 0.3)	454 (406 - 528)	24.9 (22.3 - 29.0)

Location & Cause	Incidence, thousands (95% UI)	Age-standardised Incidence rate, per 100,000 (95% UI)	Mortality, thousands (95% UI)	Age-standardised Mortality rate, per 100,000 (95% UI)	YLLs, thousands (95% UI)	Age-standardised YLL rate, per 100,000 (95% UI)	YLDs, thousands (95% UI)	Age-standardised YLD rate, per 100,000 (95% UI)	DALYs, thousands (95% UI)	Age-standardised DALYs rate, per 100,000 (95% UI)
Global Bladder cancer	3.39 (3.02 - 3.83)	0.2 (0.2 - 0.2)	0.468 (0.419 - 0.521)	0.0 (0.0 - 0.0)	30.4 (27.2 - 33.9)	1.7 (1.5 - 1.8)	2.32 (1.58 - 3.23)	0.1 (0.1 - 0.2)	32.8 (29.3 - 36.6)	1.8 (1.6 - 2.0)
Global Brain and central nervous system cancer	28.6 (22.3 - 32.1)	1.6 (1.2 - 1.8)	12.7 (9.93 - 14.1)	0.7 (0.5 - 0.8)	836 (653 - 932)	45.7 (35.7 - 50.9)	12.7 (8.41 - 17.5)	0.7 (0.5 - 1.0)	849 (662 - 946)	46.3 (36.1 - 51.7)
Global Breast cancer	28.9 (25.9 - 32.0)	1.6 (1.4 - 1.7)	6.94 (6.12 - 7.78)	0.4 (0.3 - 0.4)	443 (391 - 496)	23.9 (21.0 - 26.7)	20.8 (14.4 - 28.3)	1.1 (0.8 - 1.5)	464 (409 - 519)	25.0 (22.0 - 27.9)
Global Cervical cancer	23.8 (19.8 - 27.2)	1.3 (1.1 - 1.5)	4.83 (3.99 - 5.58)	0.3 (0.2 - 0.3)	305 (252 - 353)	16.4 (13.5 - 18.9)	12.1 (8.00 - 17.0)	0.6 (0.4 - 0.9)	317 (261 - 366)	17.0 (14.0 - 19.6)
Global Chronic lymphoid leukaemia	1.90 (1.61 - 2.21)	0.1 (0.1 - 0.1)	0.423 (0.360 - 0.487)	0.0 (0.0 - 0.0)	27.1 (23.0 - 31.1)	1.5 (1.2 - 1.7)	1.15 (0.780 - 1.61)	0.1 (0.0 - 0.1)	28.2 (24.0 - 32.4)	1.5 (1.3 - 1.8)
Global Chronic myeloid leukaemia	4.33 (3.87 - 4.87)	0.2 (0.2 - 0.3)	2.20 (1.92 - 2.53)	0.1 (0.1 - 0.1)	143 (125 - 164)	7.8 (6.8 - 8.9)	1.53 (1.08 - 2.06)	0.1 (0.1 - 0.1)	144 (126 - 166)	7.8 (6.9 - 9.0)
Global Colon and rectum cancer	18.0 (16.6 - 19.7)	1.0 (0.9 - 1.1)	6.43 (5.97 - 6.94)	0.3 (0.3 - 0.4)	413 (383 - 446)	22.3 (20.7 - 24.1)	11.4 (7.97 - 15.6)	0.6 (0.4 - 0.8)	425 (394 - 458)	22.9 (21.3 - 24.7)
Global Gallbladder and biliary tract cancer	0.736 (0.613 - 0.828)	0.0 (0.0 - 0.0)	0.412 (0.337 - 0.464)	0.0 (0.0 - 0.0)	26.0 (21.3 - 29.4)	1.4 (1.1 - 1.6)	0.201 (0.139 - 0.268)	0.0 (0.0 - 0.0)	26.2 (21.5 - 29.6)	1.4 (1.2 - 1.6)
Global Hodgkin lymphoma	20.3 (18.2 - 25.0)	1.1 (1.0 - 1.4)	4.61 (3.92 - 5.50)	0.3 (0.2 - 0.3)	303 (257 - 361)	16.5 (14.0 - 19.7)	10.2 (6.85 - 14.4)	0.6 (0.4 - 0.8)	313 (267 - 373)	17.1 (14.6 - 20.3)
Global Kidney cancer	6.39 (5.81 - 7.06)	0.3 (0.3 - 0.4)	1.20 (1.10 - 1.32)	0.1 (0.1 - 0.1)	77.8 (71.4 - 85.6)	4.2 (3.9 - 4.7)	3.27 (2.22 - 4.52)	0.2 (0.1 - 0.2)	81.1 (74.3 - 89.1)	4.4 (4.0 - 4.8)
Global Larynx cancer	0.851 (0.784 - 0.929)	0.0 (0.0 - 0.1)	0.382 (0.350 - 0.422)	0.0 (0.0 - 0.0)	24.3 (22.3 - 26.9)	1.3 (1.2 - 1.4)	1.89 (1.11 - 2.91)	0.1 (0.1 - 0.2)	26.2 (23.9 - 28.9)	1.4 (1.3 - 1.6)
Global Lip and oral cavity cancer	8.95 (7.92 - 10.1)	0.5 (0.4 - 0.5)	2.71 (2.40 - 3.07)	0.1 (0.1 - 0.2)	175 (155 - 198)	9.5 (8.4 - 10.7)	3.89 (2.67 - 5.34)	0.2 (0.1 - 0.3)	179 (159 - 203)	9.7 (8.6 - 11.0)

Location & Cause	Incidence, thousands (95% UI)	Age-standardised Incidence rate, per 100,000 (95% UI)	Mortality, thousands (95% UI)	Age-standardised Mortality rate, per 100,000 (95% UI)	YLLs, thousands (95% UI)	Age-standardised YLL rate, per 100,000 (95% UI)	YLDs, thousands (95% UI)	Age-standardised YLD rate, per 100,000 (95% UI)	DALYs, thousands (95% UI)	Age-standardised DALYs rate, per 100,000 (95% UI)
Global Liver cancer	6.50 (5.93 - 7.17)	0.4 (0.3 - 0.4)	4.20 (3.83 - 4.60)	0.2 (0.2 - 0.2)	271 (247 - 298)	14.7 (13.4 - 16.1)	1.81 (1.28 - 2.43)	0.1 (0.1 - 0.1)	273 (249 - 300)	14.8 (13.5 - 16.2)
Global Malignant skin melanoma	12.7 (10.3 - 15.9)	0.7 (0.6 - 0.9)	1.20 (1.02 - 1.50)	0.1 (0.1 - 0.1)	77.2 (65.7 - 96.8)	4.2 (3.6 - 5.2)	6.29 (3.96 - 9.44)	0.3 (0.2 - 0.5)	83.5 (70.6 - 105)	4.5 (3.8 - 5.7)
Global Mesothelioma	0.500 (0.367 - 0.632)	0.0 (0.0 - 0.0)	0.301 (0.224 - 0.378)	0.0 (0.0 - 0.0)	19.1 (14.2 - 24.0)	1.0 (0.8 - 1.3)	0.175 (0.112 - 0.248)	0.0 (0.0 - 0.0)	19.2 (14.3 - 24.1)	1.0 (0.8 - 1.3)
Global Multiple myeloma	0.797 (0.534 - 0.962)	0.0 (0.0 - 0.1)	0.405 (0.279 - 0.479)	0.0 (0.0 - 0.0)	25.8 (17.8 - 30.6)	1.4 (1.0 - 1.6)	0.416 (0.245 - 0.600)	0.0 (0.0 - 0.0)	26.3 (18.1 - 31.0)	1.4 (1.0 - 1.7)
Global Nasopharynx cancer	7.16 (6.38 - 8.05)	0.4 (0.3 - 0.4)	1.84 (1.68 - 2.01)	0.1 (0.1 - 0.1)	120 (110 - 132)	6.5 (6.0 - 7.2)	3.92 (2.75 - 5.36)	0.2 (0.1 - 0.3)	124 (114 - 136)	6.8 (6.2 - 7.4)
Global Non-Hodgkin lymphoma	28.0 (24.8 - 32.0)	1.5 (1.4 - 1.8)	10.5 (9.65 - 11.4)	0.6 (0.5 - 0.6)	691 (636 - 751)	37.8 (34.8 - 41.1)	14.9 (10.3 - 20.2)	0.8 (0.6 - 1.1)	706 (650 - 769)	38.6 (35.6 - 42.1)
Global Oesophageal cancer	1.53 (1.34 - 1.73)	0.1 (0.1 - 0.1)	1.04 (0.903 - 1.17)	0.1 (0.0 - 0.1)	65.6 (57.2 - 74.2)	3.5 (3.1 - 4.0)	0.522 (0.368 - 0.709)	0.0 (0.0 - 0.0)	66.1 (57.6 - 75.0)	3.5 (3.1 - 4.0)
Global Other leukaemia	14.7 (12.1 - 16.6)	0.8 (0.7 - 0.9)	8.54 (7.00 - 9.73)	0.5 (0.4 - 0.5)	566 (465 - 645)	31.0 (25.5 - 35.3)	9.72 (6.70 - 13.4)	0.5 (0.4 - 0.7)	576 (471 - 656)	31.5 (25.8 - 35.9)
Global Other malignant neoplasms	85.3 (77.6 - 93.4)	4.7 (4.3 - 5.1)	30.4 (27.4 - 33.5)	1.7 (1.5 - 1.8)	2 020 (1 820 - 2 230)	111.1 (100.1 - 122.3)	44.5 (31.2 - 60.2)	2.4 (1.7 - 3.3)	2 070 (1 860 - 2 280)	113.5 (102.2 - 124.9)
Global Other pharynx cancer	1.81 (1.56 - 2.09)	0.1 (0.1 - 0.1)	1.02 (0.862 - 1.18)	0.1 (0.0 - 0.1)	65.0 (54.8 - 75.2)	3.5 (3.0 - 4.0)	0.526 (0.357 - 0.699)	0.0 (0.0 - 0.0)	65.5 (55.3 - 76.0)	3.5 (3.0 - 4.1)
Global Ovarian cancer	15.2 (12.6 - 17.4)	0.8 (0.7 - 0.9)	2.88 (2.35 - 3.32)	0.2 (0.1 - 0.2)	188 (153 - 216)	10.2 (8.3 - 11.8)	7.70 (5.14 - 10.6)	0.4 (0.3 - 0.6)	195 (159 - 225)	10.6 (8.6 - 12.2)
Global Pancreatic cancer	1.73 (1.57 - 1.90)	0.1 (0.1 - 0.1)	1.21 (1.11 - 1.33)	0.1 (0.1 - 0.1)	77.3 (71.0 - 85.5)	4.2 (3.8 - 4.6)	0.429 (0.306 - 0.570)	0.0 (0.0 - 0.0)	77.8 (71.2 - 85.9)	4.2 (3.8 - 4.6)
Global Prostate cancer	2.02 (1.79 - 2.51)	0.1 (0.1 - 0.1)	0.317 (0.278 - 0.399)	0.0 (0.0 - 0.0)	20.1 (17.6 - 25.3)	1.1 (0.9 - 1.4)	1.53 (1.02 - 2.19)	0.1 (0.1 - 0.1)	21.7 (19.0 - 26.8)	1.2 (1.0 - 1.4)

Location & Cause	Incidence, thousands (95% UI)	Age-standardised Incidence rate, per 100,000 (95% UI)	Mortality, thousands (95% UI)	Age-standardised Mortality rate, per 100,000 (95% UI)	YLLs, thousands (95% UI)	Age-standardised YLL rate, per 100,000 (95% UI)	YLDs, thousands (95% UI)	Age-standardised YLD rate, per 100,000 (95% UI)	DALYs, thousands (95% UI)	Age-standardised DALYs rate, per 100,000 (95% UI)
Global Stomach cancer	11.4 (10.4 - 12.3)	0.6 (0.6 - 0.7)	6.00 (5.51 - 6.54)	0.3 (0.3 - 0.4)	385 (353 - 419)	20.8 (19.1 - 22.7)	4.08 (2.93 - 5.46)	0.2 (0.2 - 0.3)	389 (357 - 424)	21.0 (19.3 - 22.9)
Global Testicular cancer	29.4 (25.9 - 34.0)	1.6 (1.4 - 1.8)	2.83 (2.58 - 3.11)	0.2 (0.1 - 0.2)	183 (167 - 201)	9.9 (9.1 - 10.9)	14.7 (9.87 - 20.7)	0.8 (0.5 - 1.1)	198 (180 - 217)	10.7 (9.8 - 11.8)
Global Thyroid cancer	16.2 (13.7 - 18.3)	0.9 (0.7 - 1.0)	1.24 (1.07 - 1.42)	0.1 (0.1 - 0.1)	80.6 (69.5 - 92.2)	4.4 (3.8 - 5.0)	8.23 (5.38 - 12.0)	0.4 (0.3 - 0.6)	88.8 (76.4 - 102)	4.8 (4.2 - 5.5)
Global Tracheal, bronchus, and lung cancer	7.43 (6.75 - 8.15)	0.4 (0.4 - 0.4)	4.96 (4.51 - 5.44)	0.3 (0.2 - 0.3)	320 (291 - 351)	17.3 (15.8 - 19.0)	2.17 (1.56 - 2.88)	0.1 (0.1 - 0.2)	322 (293 - 353)	17.4 (15.9 - 19.1)
Global Uterine cancer	3.82 (2.85 - 4.39)	0.2 (0.2 - 0.2)	0.338 (0.244 - 0.385)	0.0 (0.0 - 0.0)	21.3 (15.4 - 24.3)	1.1 (0.8 - 1.3)	2.10 (1.30 - 3.02)	0.1 (0.1 - 0.2)	23.4 (16.9 - 26.5)	1.3 (0.9 - 1.4)

Estimates are for 15–29-year-olds, both sexes combined (this differs from the main text table 1 which shows incidence, mortality, and DALYs numbers and rates for 15–39-year-olds). Rates are reported per 100,000 person-years. Age-standardisation used the GBD 2019 world standard. SDI categories do not sum precisely to the global total as the GBD study does not provide separate estimates for all locations globally and an adjustment factor is made between all estimated locations which have corresponding SDI values and the global estimate. Total AYA Cancers=all malignant neoplasms in this age group excluding NMSC. DALYs=disability-adjusted life-years. YLDs=years lived with disability. YLLs=years of life lost. GBD=Global Burden of Diseases, Injuries, and Risk Factors Study. UI=uncertainty interval. SDI=Socio-demographic Index. Other malignant neoplasms are cancers without a detailed GBD cause separately listed. Other leukaemia included leukaemias not otherwise specified.

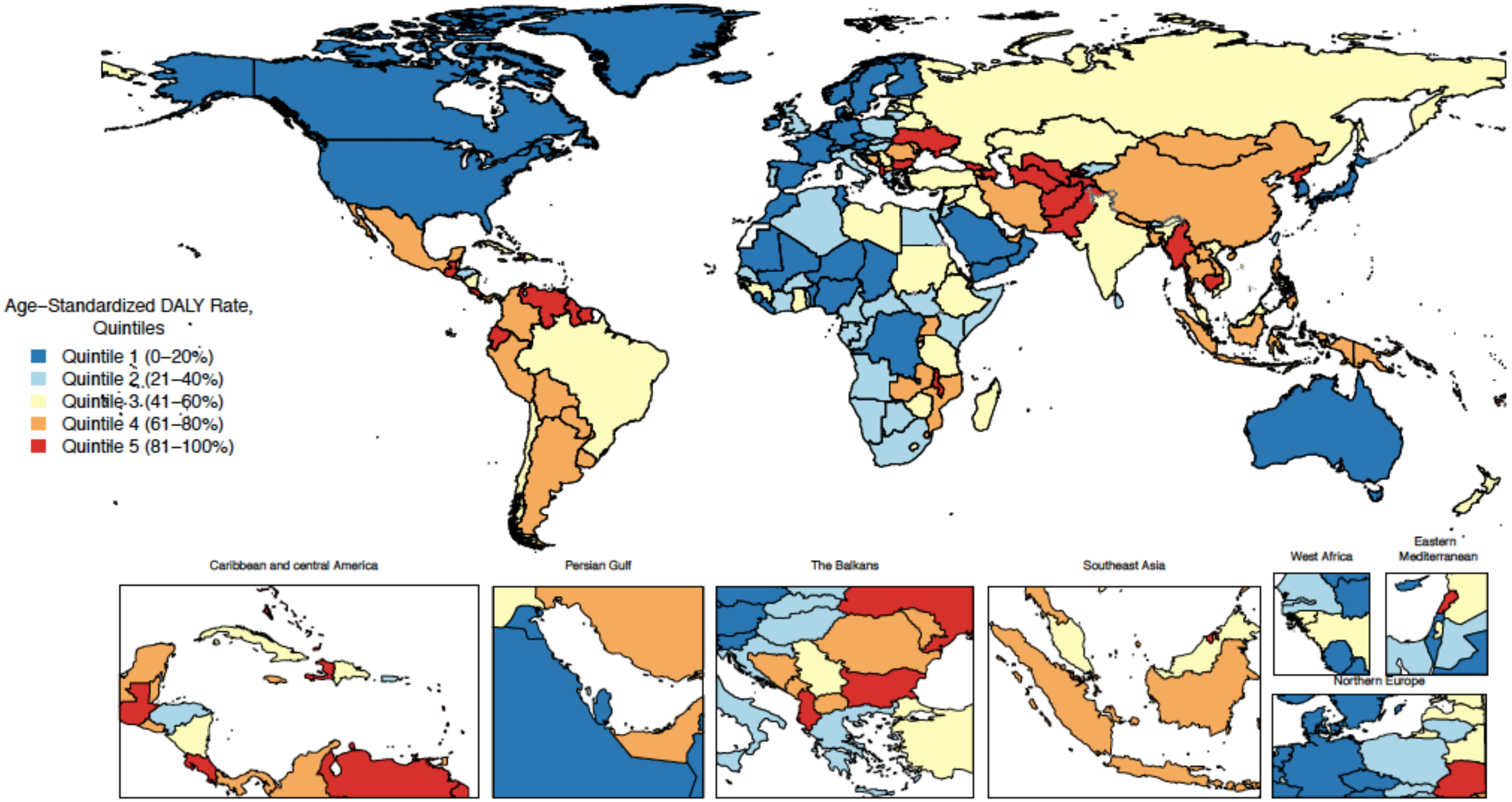

Appendix Figure 16: Global map of age-standardised DALY rates for adolescent and young adult cancers ages 15-29 years for both sexes combined in 2019, categorised by quintile, excluding non-melanoma skin cancers. Rates are expressed per 100,000 person-years. DALYs=disability-adjusted life-years. For adolescent and young adult cancers: Quintile 1 (0-20%): < 376, Quintile 2 (21-40%): 376 to < 445, Quintile 3 (41-60%): 445 to < 527, Quintile 4 (61-80%): 527 to < 639, Quintile 5 (81-100%): ≥ 639. Western Sahara and French Guiana are colored white in the map as they were not modelled locations in the Global Burden of Diseases, Injuries, and Risk Factors Study 2019, and therefore no estimates were produced for these areas.

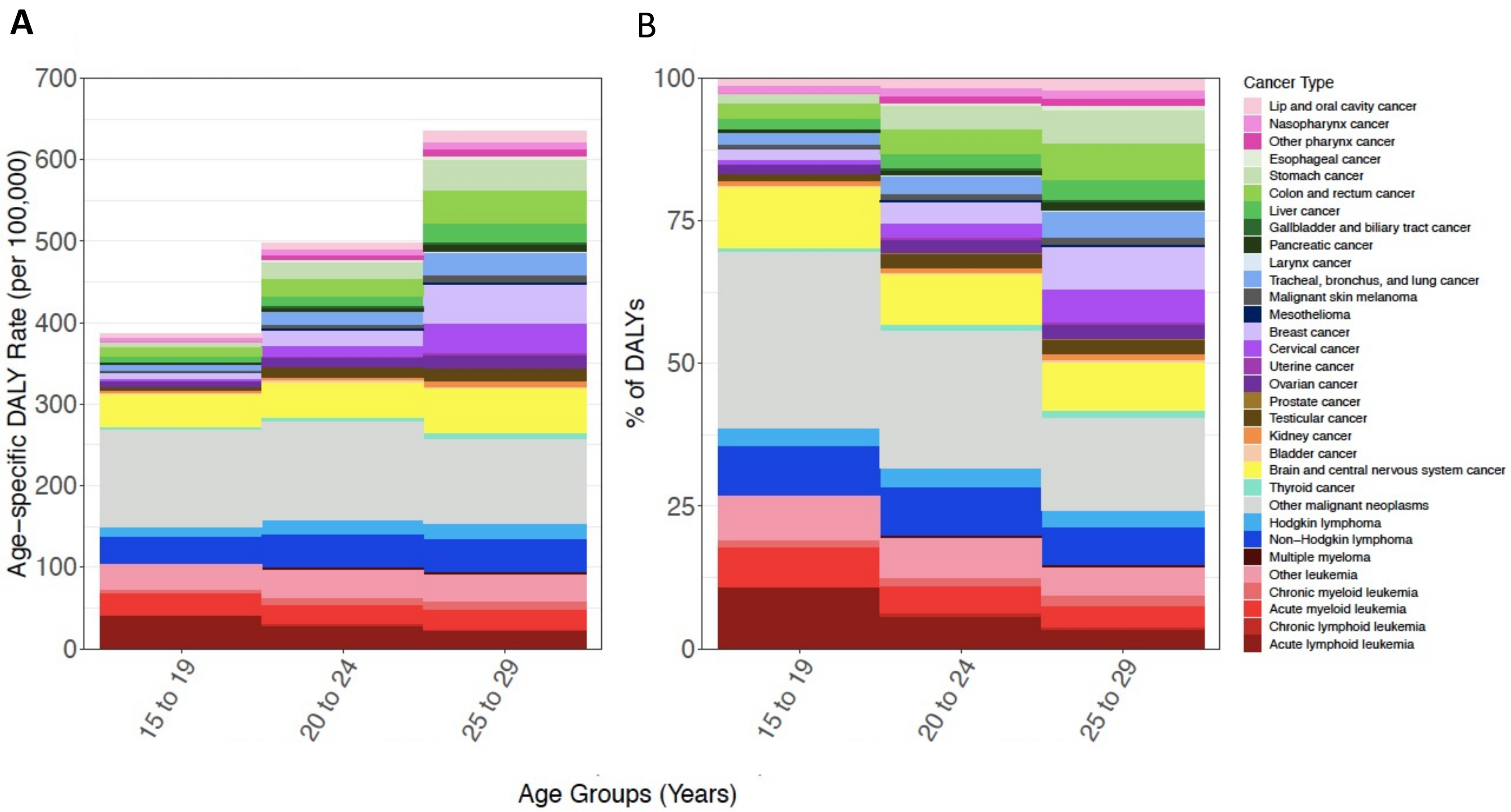

Appendix Figure 17: Adolescent and young adult (15-29 years) cancer burden by age group for both sexes, 2019, in (A) age-specific DALY rates and (B) proportional DALY burden. Rates are expressed per 100,000 person-years. Other malignant neoplasms: all malignancies without a separate GBD cause category listed; this category does not include non-melanoma skin cancers and myelodysplastic/myeloproliferative neoplasms, which are separate GBD cause categories not included in this analysis. Other leukaemia included leukaemias not otherwise specified. DALYs=disability-adjusted life-years.

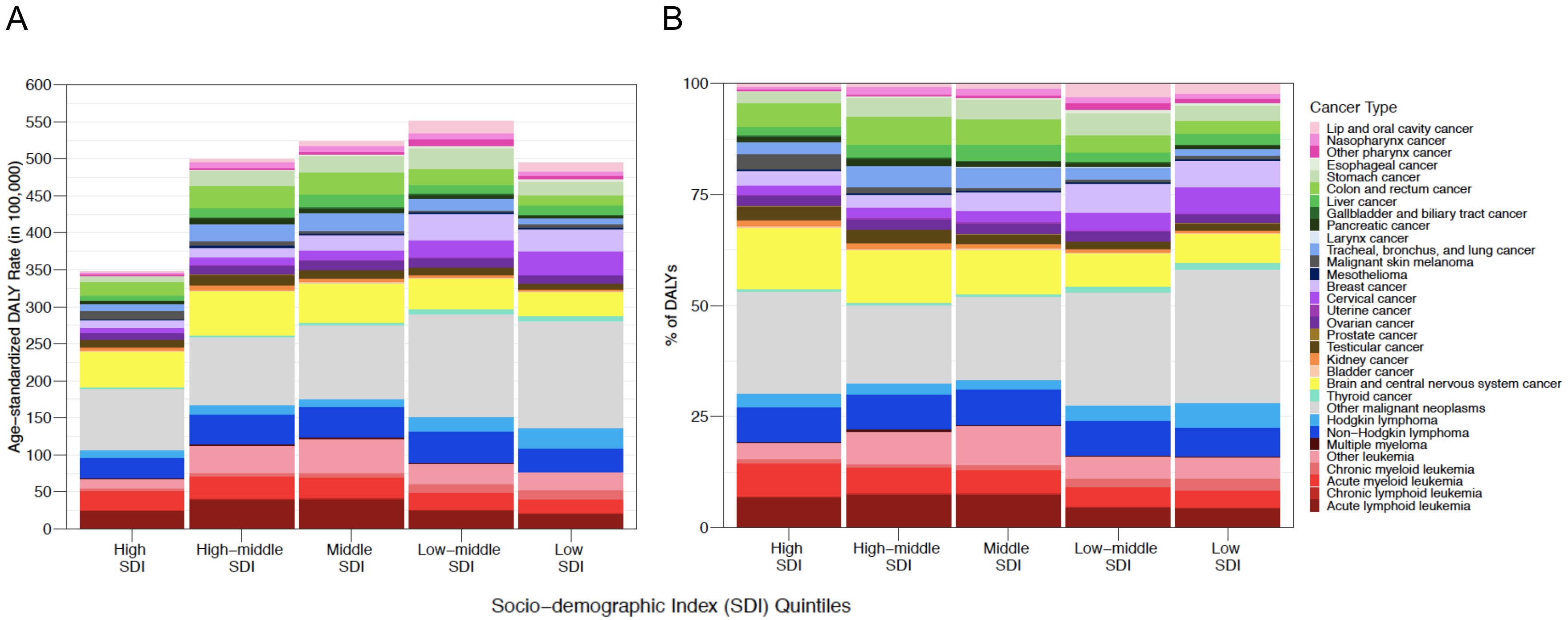

Appendix Figure 18: Adolescent and young adult (15-29 years) cancer, both sexes combined, 2019 by Socio-demographic Index (SDI) quintiles in (A) age-standardised DALY rates and (B) proportional DALY burden. Rates are expressed per 100,000 person-years. Other malignant neoplasms: all malignancies without a separate GBD cause category listed; this category does not include non-melanoma skin cancers and myelodysplastic/myeloproliferative neoplasms, which are separate GBD cause categories not included in this analysis. Other leukaemia included leukaemias not otherwise specified. DALYs=disability-adjusted life-years.

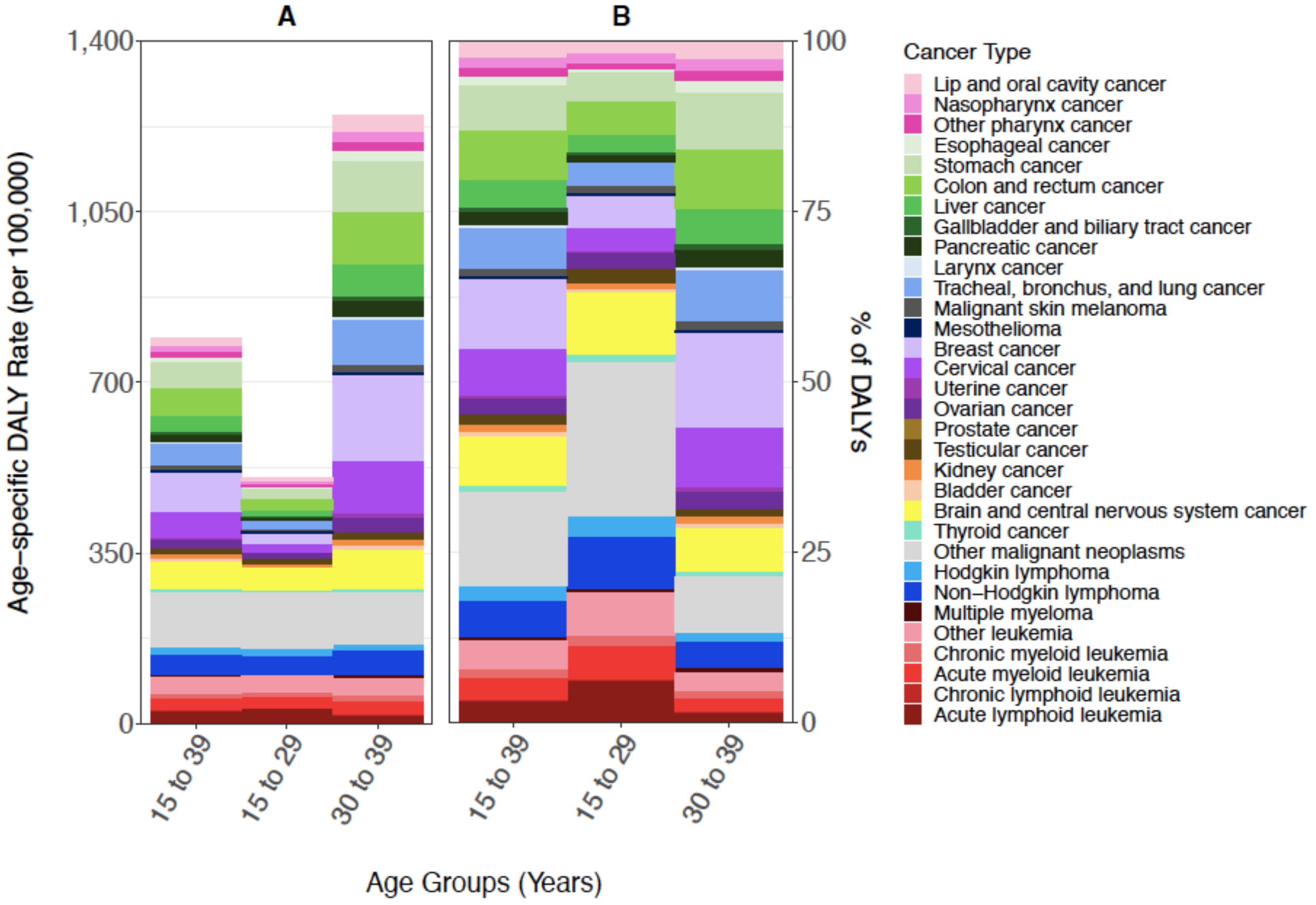

Appendix Figure 19: Adolescent and young adult cancer burden by 15-39, 15-29, and 30-39 year-old age groups for both sexes, 2019, in (A) age-specific DALY rates and (B) proportional DALY burden. Rates are expressed per 100,000 person-years. Other malignant neoplasms: all malignancies without a separate GBD cause category listed; this category does not include non-melanoma skin cancers and myelodysplastic/myeloproliferative neoplasms, which are separate GBD cause categories not included in this analysis. Other leukaemia included leukaemias not otherwise specified. DALYs=disability-adjusted life-years.

References

1 GBD 2019 Cancer Collaborators. Morbidity and mortality for 29 cancer groups by country and territory and Socio-demographic Index, 1990-2019: a systematic analysis for the Global Burden of Disease Study 2019. *JAMA Oncology*; **In Press**.

2 Force LM, Abdollahpour I, Advani SM, *et al.* The global burden of childhood and adolescent cancer in 2017: an analysis of the Global Burden of Disease Study 2017. *The Lancet Oncology* 2019; **20**: 1211–25.

3 Vos T, Lim SS, Abbafati C. Global burden of 369 diseases and injuries in 204 countries and territories, 1990–2019: a systematic analysis for the Global Burden of Disease Study 2019. *The Lancet* 2020; **396**: 1204–22.

4 Stevens G, Alkema L, Black R, et al. Guidelines for Accurate and Transparent Health Estimates Reporting: the GATHER statement. *The Lancet* 2016; **388**: 19–23.

5 What Should the Age Range Be for AYA Oncology? *J Adolesc Young Adult Oncol* 2011; **1**: 3–10.

6 National Service Delivery Framework for Adolescents and Young Adults with Cancer | Cancer Australia. https://www.canceraustralia.gov.au/publications-and-resources/cancer-australia-publications/national-service-delivery-framework-adolescents-and-young-adults-cancer (accessed July 21, 2021).

7 Li CK, Dalvi R, Yonemori K, *et al.* Care of adolescents and young adults with cancer in Asia: results of an ESMO/SIOPE/SIOP Asia survey. *ESMO Open* 2019; **4**: e000467.

8 Surveillance, Epidemiology, and End Results (SEER) Program (Www.Seer.Cancer.Gov) SEER*Stat Database: Incidence - SEER 18.

9 Doll R, Payne P, Waterhouse J, editors. Cancer Incidence in Five Continents, Vol. I. Geneva: Union Internationale Contre le Cancer, 1966 https://publications.iarc.fr/Non-Series-Publications/Other-Non-Series-Publications/Cancer-Incidence-In-Five-Continents-Volume-I-1966 (accessed Feb 24, 2021).

10 Doll R, Muir CS, Waterhouse JA. Cancer Incidence in Five Continents, Vol. II. Geneva: Union Internationale Contre le Cancer, 1970.

11 Waterhouse J, Muir C, Correa P, Powell J. Cancer Incidence in Five Continents III. Lyon: IARC; 1976.

12 Waterhouse J, Muir C, Shanmugaratnam K, Powell J. Cancer Incidence in Five Continents IV. Lyon: IARC; 1982.

13 Muir C, Mack T, Powell J, Whelan S. Cancer Incidence in Five Continents V. Lyon: IARC; 1987.

14 Parkin D, Muir C, Whelan S, Gao Y, Ferlay J, Powell J. Cancer Incidence in Five Continents VI. Lyon: IARC; 1992.

15 Parkin D, Whelan S, Ferlay J, Raymond L, Young J. Cancer Incidence in Five Continents VII. Lyon: IARC; 1997.

16 Parkin D, Whelan S, Ferlay J, Teppo L, Thomas D. Cancer Incidence in Five Continents VIII. Lyon: IARC; 2002.

17 Curado M, Edwards B, Shin H, et al. Cancer Incidence in Five Continents IX. Lyon: IARC; 2007. http://www.iarc.fr/en/publ.

18 Forman D, Bray F, Brewster D, et al. Cancer Incidence in Five Continents X. http://ci5.iarc.fr. Published 2013.

19 Bray F, Colombet M, Mery L, *et al.*, editors. Cancer Incidence in Five Continents. Lyon, France: International Agency for Research on Cancer, 2017 https://ci5.iarc.fr.

20 Steliarova-Foucher E, O'Callaghan M, Ferlay J, *et al.* The European Cancer Observatory: A new data resource. *Eur J Cancer* 2015; **51**: 1131–43.

21 Engholm G, Ferlay J, Christensen N, *et al.* NORDCAN--a Nordic tool for cancer information, planning, quality control and research. *Acta Oncol* 2010; **49**: 725–36.

22 Murray CJL, Aravkin AY, Zheng P. Global burden of 87 risk factors in 204 countries and territories, 1990–2019: a systematic analysis for the Global Burden of Disease Study 2019. *The Lancet* 2020; **396**: 1223–49.

23 Barber RM, Fullman N, Sorensen RJD, et al. Healthcare Access and Quality Index based on mortality from causes amenable to personal health care in 195 countries and territories, 1990-2015: a novel analysis from the Global Burden of Disease Study 2015. *Lancet* 2017; **390**: 231–66.

24 Foreman KJ, Lozano R, Lopez AD, Murray CJ. Modeling causes of death: an integrated approach using CODEm. *Popul Health Metr* 2012; **10**: 1.

25 Wang H, Abbas KM, Abbasifard M. Global age-sex-specific fertility, mortality, healthy life expectancy (HALE), and population estimates in 204 countries and territories, 1950-2019: a comprehensive demographic analysis for the Global Burden of Disease Study 2019. *Lancet* 2020; **396**: 1160–203.

26 Asadzadeh Vostakolaei F, Karim-Kos HE, Janssen-Heijnen MLG, Visser O, Verbeek ALM, Kiemeney LALM. The validity of the mortality to incidence ratio as a proxy for site-specific cancer survival. *Eur J Public Health* 2011; **21**: 573–7.

27 SEER*Stat Software. 2014 http://seer.cancer.gov/seerstat/.

28 Fitzmaurice C, Abate D, Abbasi N, *et al.* Global, Regional, and National Cancer Incidence, Mortality, Years of Life Lost, Years Lived With Disability, and Disability-Adjusted Life-Years for 29 Cancer Groups, 1990 to 2017: A Systematic Analysis for the Global Burden of Disease Study. *JAMA Oncol* 2019; **5**: 1749–68.

29 SEER Cancer Statistics Review 1975-2011. http://seer.cancer.gov/csr/1975_2011/results_merged/topic_survival_by_year_dx.p.

30 Neal RD, Din NU, Hamilton W, *et al.* Comparison of cancer diagnostic intervals before and after implementation of NICE guidelines: analysis of data from the UK General Practice Research Database. *British Journal of Cancer* 2014; **110**: 584–92.

31 Surveillance, Epidemiology, and End Results (SEER) Program (www.seer.cancer.gov) SEER*Stat Database: Incidence - SEER 18 Regs Research Data + Hurricane Katrina Impacted Louisiana Cases, Nov 2012 Sub (1973-2010 varying) - Linked To County Attributes - Total U.S., 1969-2011 Counties, National Cancer Institute, DCCPS, Surveillance Research Program, Surveillance Systems Branch, released April 2013, based on the November 2012 submission.

32 Allgar VL, Neal RD. Delays in the diagnosis of six cancers: analysis of data from the National Survey of NHS Patients: Cancer. *Br J Cancer* 2005; **92**: 1959–70.

33 Neal RD, Cannings-John R, Hood K, *et al.* Excision of malignant melanomas in North Wales: effect of location and surgeon on time to diagnosis and quality of excision. *Family Practice* 2008; **25**: 221–7.

34 Kewalramani T, Nimer SD, Zelenetz AD, *et al.* Progressive disease following autologous transplantation in patients with chemosensitive relapsed or primary refractory Hodgkin's disease or aggressive non-Hodgkin's lymphoma. *Bone Marrow Transplant* 2003; **32**: 673–9.

35 Esteban D, Tovar N, Jiménez R, *et al.* Patients with relapsed/refractory chronic lymphocytic leukaemia may benefit from inclusion in clinical trials irrespective of the therapy received: a case-control retrospective analsysis. *Blood Cancer J* 2015; **5**: e356.

36 Canova C, Giorato E, Roveron G, Turrini P, Zanotti R. Validation of a stoma-specific quality of life questionnaire in a sample of patients with colostomy or ileostomy. *Colorectal Dis* 2013; **15**: e692-698.

37 Caricato M, Ausania F, Ripetti V, Bartolozzi F, Campoli G, Coppola R. Retrospective analysis of long-term defunctioning stoma complications after colorectal surgery. *Colorectal Dis* 2007; **9**: 559–61.

38 Erwin-Toth P, Thompson SJ, Davis JS. Factors impacting the quality of life of people with an ostomy in North America: results from the Dialogue Study. *J Wound Ostomy Continence Nurs* 2012; **39**: 417–22; quiz 423–4.

39 Catalona WJ, Carvalhal GF, Mager DE, Smith DS. Potency, continence and complication rates in 1,870 consecutive radical retropubic prostatectomies. *J Urol* 1999; **162**: 433–8.

40 Donnellan SM, Duncan HJ, MacGregor RJ, Russell JM. Prospective assessment of incontinence after radical retropubic prostatectomy: objective and subjective analysis. *Urology* 1997; **49**: 225–30.

41 Eastham JA, Kattan MW, Rogers E, *et al.* Risk factors for urinary incontinence after radical prostatectomy. *J Urol* 1996; **156**: 1707–13.

42 Kundu SD, Roehl KA, Eggener SE, Antenor JAV, Han M, Catalona WJ. Potency, continence and complications in 3,477 consecutive radical retropubic prostatectomies. *J Urol* 2004; **172**: 2227–31.

43 Potosky AL, Davis WW, Hoffman RM, *et al.* Five-Year Outcomes After Prostatectomy or Radiotherapy for Prostate Cancer: The Prostate Cancer Outcomes Study. *JNCI Journal of the National Cancer Institute* 2004; **96**: 1358–67.

44 Sacco E, Prayer-Galetti T, Pinto F, *et al.* Urinary incontinence after radical prostatectomy: incidence by definition, risk factors and temporal trend in a large series with a long-term follow-up. *BJU Int* 2006; **97**: 1234–41.

45 Stanford JL, Feng Z, Hamilton AS, *et al.* Urinary and sexual function after radical prostatectomy for clinically localized prostate cancer: the Prostate Cancer Outcomes Study. *JAMA* 2000; **283**: 354–60.

46 Walsh PC, Marschke P, Ricker D, Burnett AL. Patient-reported urinary continence and sexual function after anatomic radical prostatectomy. *Urology* 2000; **55**: 58–61.

47 World Health Organization. Global initiative for childhood cancer. 2019; http://www.who.int/cancer/childhood-cancer/en/.

48 Cervical cancer elimination initiative. https://www.who.int/initiatives/cervical-cancer-elimination-initiative (accessed Aug 2, 2021).

<u>Author Contributions</u>

Managing the estimation or publications process
Lisa M Force, Kelly Compton, Christopher J L Murray, and Christina Fitzmaurice.

Writing the first draft of the manuscript
Elysia Alvarez, Lisa M Force, Rixing Xu, Kelly Compton, Theresa Keegan, Hany Ariffin, Ronald Barr, Yana Erdomaeva, Sanjeeva Gunasekera, Yetunde John-Akinola, Tyler Ketterl, Tezer Kutluk, Marcio Malogolowkin, Prashant Mathur, Venkatraman Radhakrishnan, Lynn Ries, Carlos Rodriguez-Galindo, Garik Sagoyan, Iyad Sultan, Archie Bleyer, and Nickhill Bhakta.

Primary responsibility for applying analytical methods to produce estimates
Lisa M Force, Rixing Xu, Dan Lu, Jonathan Kocarnik, James Harvey, Alyssa Pennini, Frances E Dean, Weijia Fu, Christopher J L Murray and Christina Fitzmaurice.

Primary responsibility for seeking, cataloguing, extracting, or cleaning data; designing or coding figures and tables
Elysia Alvarez, Lisa M Force, Rixing Xu, Dan Lu, Hannah Henrikson, Jonathan Kocarnik, James Harvey, Alyssa Pennini, Martina Vargas, Archie Bleyer, and Nickhill Bhakta.

Providing data or critical feedback on data sources
Lisa M Force, Jonathan M Kocarnik, Hany Ariffin, Ronald D Barr, D Sanjeeva Gunasekera, Yetunde O John-Akinola, Venkatraman Radhakrishnan, Hedayat Abbastabar, Sherief Abd-Elsalam, Amir Abdoli, Aidin Abedi, Hassan Abidi, Hassan Abolhassani, Hiwa Abubaker Ali, Basavaprabhu Achappa, Isaac Akinkunmi Adedeji, Qorinah Estiningtyas Sakilah Adnani, Bright Opoku Ahinkorah, Sajjad Ahmad, Sepideh Ahmadi, Muktar Beshir Ahmed, Tarik Ahmed Rashid, Yusra Ahmed Salih, Fares Alahdab, Abdulhadi A AlAmodi, Fahad Mashhour Alanezi, Turki M Alanzi, Yosef Alemayehu, Fadwa Naji Alhalaiqa, Hanadi Al Hamad, Robert Kaba Alhassan, Saqib Ali, Syed Mohamed Aljunid, Motasem Alkhayyat, Sami Almustanyir, Nelson Alvis-Guzman, Edward Kwabena Ameyaw, Saeed Amini, Hubert Amu, Fereshteh Ansari, Davood Anvari, Jalal Arabloo, Morteza Arab-Zozani, Ayele Mamo Argaw, Mohammad Reza Atashzar, Alok Atreya, Avinash Aujayeb, Marcel Ausloos, Beatriz Paulina Ayala Quintanilla, Alemu Degu Ayele, Solomon Shitu Ayen, Mohammed A Azab, Hiva Azami, Ahmed Y Azzam, Saeed Bahadory, Atif Amin Baig, Jennifer L Baker, Maciej Banach, Till Winfried Bärnighausen, Amadou Barrow, Abdul-Monim Mohammad Batiha, Masoud Behzadifar, Rebuma Belete, Akshaya Srikanth Bhagavathula, Sonu Bhaskar, Vijayalakshmi S Bhojaraja, Sadia Bibi, Setognal Birara, Obasanjo Afolabi Bolarinwa, Srinivasa Rao Bolla, Archith Boloor, Dejana Braithwaite, Katrin Burkart, Ferrán Catalá-López, Francieli Cembranel, Raja Chandra Chakinala, Vijay Kumar Chattu, Pankaj Chaturvedi, Akhilanand Chaurasia, Devasahayam J Christopher, Dinh-Toi Chu, Joao Conde, Omar B Da'ar, Saad M A Dahlawi, Xiaochen Dai, Giovanni Damiani, Lalit Dandona, Rakhi Dandona, Ahmad Daryani, Fernando Pio De la Hoz, Rupak Desai, Samath Dhamminda Dharmaratne, Mostafa Dianatinasab, Mojtaba Didehdar, Huyen Phuc Do, Saeid Doaei, Fariba Dorostkar, Wendel Mombaque dos Santos, Thomas M Drake, Michael Ekholuenetale, Hassan El-Abid, Maysaa El Sayed Zaki, Jawad Fares, Abidemi Omolara Fasanmi, Getahun Fetensa, Irina Filip, Takeshi Fukumoto, Peter Andras Gaal, Mohamed M Gad, Tushar Garg, Teferi Gebru Gebremeskel, Belete Negese Belete Gemeda, Ahmad Ghashghaee, Jamshid Gholizadeh Navashenaq, Abraham Tamirat Gizaw, Mahaveer Golechha, Pouya Goleij, Bárbara Niegia Garcia Goulart, Ayman Grada, Bhawna Gupta, Vivek Kumar Gupta, Veer Bala Gupta, Nima Hafezi-Nejad, Alemayehu Hailu, Arvin Haj-Mirzaian, Rabih Halwani, Shafiul Haque, Josep Maria Haro, Ahmed I Hasaballah, Treska S Hassan, Soheil Hassanipour, Claudiu Herteliu, Demisu Zenbaba Heyi, Kamal Hezam, Nobuyuki Horita, Md Mahbub Hossain, Mohammad-Salar Hosseini, Mehdi Hosseinzadeh,

Mowafa Househ, Nawfal R Hussein, Segun Emmanuel Ibitoye, Kaire Innos, Sheikh Mohammed Shariful Islam, Nahlah Elkudssiah Ismail, Gaetano Isola, Vardhmaan Jain, Mihajlo Jakovljevic, Amirreza Javadi Mamaghani, Shubha Jayaram, Seyed Behzad Jazayeri, Tamas Joo, Nitin Joseph, Farahnaz Joukar, Mikk Jürisson, Billingsley Kaambwa, Leila R Kalankesh, Feroze Kaliyadan, Zul Kamal, Himal Kandel, Ibraheem M Karaye, Bekalu Getnet Kassa, Joonas H Kauppila, Amene Abebe Kerbo, Yousef Saleh Khader, Himanshu Khajuria, Moien AB Khan, Md Nuruzzaman Khan, Maseer Khan, Ejaz Ahmad Khan, Jagdish Khubchandani, Min Seo Kim, Yun Jin Kim, Sezer Kisa, Adnan Kisa, Katarzyna Kissimova-Skarbek, Ali-Asghar Kolahi, Sindhura Lakshmi Koulmane Laxminarayana, G Anil Kumar, Savita Lasrado, Yo Han Lee, Sang-woong Lee, James Leigh, Xuefeng Liu, Stany W Lobo, Hassan Magdy Abd El Razek, Muhammed Magdy Abd El Razek, Alaa Makki, Mohammed A Mamun, Navid Manafi, Fariborz Mansour-Ghanaei, Borhan Mansouri, Mohammad Ali Mansournia, Clara N Matei, Walter Mendoza, Ritesh G Menezes, Alexios-Fotios A Mentis, Tuomo J Meretoja, Bartosz Miazgowski, Prasanna Mithra, Karzan Abdulmuhsin Mohammad, Mokhtar Mohammadi, Abdollah Mohammadian-Hafshejani, Reza Mohammadpourhodki, Shafiu Mohammed, Ali H Mokdad, Mariam Molokhia, Lorenzo Monasta, Yousef Moradi, Ghobad Moradi, Ebrahim Mostafavi, Sumaira Mubarik, Mohsen Naghavi, Luigi Naldi, Sreenivas Narasimha Swamy, Aparna Ichalangod Narayana, Biswa Prakash Nayak, Javad Nazari, Ionut Negoi, Samata Nepal, Sandhya Neupane Kandel, Haruna Asura Nggada, Cuong Tat Nguyen, Hamed Nosrati, Hasti Nouraei, Vincent Ebuka Nwatah, Bogdan Oancea, Ayodipupo Sikiru Oguntade, Andrew T Olagunju, Tinuke O Olagunju, Emad Omar, Sokking Ong, Obinna E Onwujekwe, Bilcha Oumer, Mayowa O Owolabi, Mahesh P A, Jagadish Rao Padubidri, Keyvan Pakshir, Adrian Pana, Anamika Pandey, Shahina Pardhan, Fatemeh Pashazadeh Kan, Maja Pasovic, Jenil R Patel, Siddhartha Pati, Uttam Paudel, Renato B Pereira, Arokiasamy Perianayagam, Maarten J Postma, Hadi Pourjafar, Akila Prashant, Amir Radfar, Raghu Anekal Radhakrishnan, Alireza Rafiei, Fakher Rahim, Amir Masoud Rahmani, Sowmya J Rao, Chythra R Rao, Sina Rashedi, Goura Kishor Rath, Salman Rawaf, David Laith Rawaf, Lal Rawal, Reza Rawassizadeh, Andre M N Renzaho, Nima Rezaei, Maryam Rezaei, Jefferson Antonio Buendia Rodriguez, Luca Ronfani, Gholamreza Roshandel, Godfrey M Rwegerera, Siamak Sabour, Basema Saddik, Umar Saeed, S Mohammad Sajadi, Marwa Rashad Salem, Hamideh Salimzadeh, Abdallah M Samy, Juan Sanabria, Arash Sarveazad, Brijesh Sathian, Monika Sawhney, Mete Saylan, Abdul-Aziz Seidu, Mario Šekerija, Endalew Gemechu Sendo, Allen Seylani, Kenbon Seyoum, Feng Sha, Masood Ali Shaikh, Mohammed Shannawaz, Sara Sheikhbahaei, B Suresh Kumar Shetty, Adithi Shetty, Jae Il Shin, Reza Shirkoohi, K M Shivakumar, Soraya Siabani, Negussie Boti Sidemo, Diego Augusto Santos Silva, Guilherme Silva Julian, Jasvinder A Singh, Jitendra Kumar Singh, Achintya Dinesh Singh, Valentin Yurievich Skryabin, Anna Aleksandrovna Skryabina, Ranjeeta Subedi, Viktória Szerencsés, Miklós Szócska, Rafael Tabarés-Seisdedos, Takahiro Tabuchi, Amir Taherkhani, Mircea Tampa, Ker-Kan Tan, Jarnail Singh Thakur, Nihal Thomas, Ruoyan Tobe-Gai, Munkhsaikhan Togtmol, Seyed Abolfazl Tohidast, Musliu Adetola Tolani, Mathilde Touvier, Marcos Roberto Tovani-Palone, Bach Xuan Tran, Irfan Ullah, Saif Ullah, Krishna Kishore Umapathi, Bhaskaran Unnikrishnan, Era Upadhyay, Tolassa Wakayo Ushula, Sahel Valadan Tahbaz, Shoban Babu Varthya, Vasily Vlassov, Giang Thu Vu, Yasir Waheed, Adisu Birhanu Weldesenbet, Ronny Westerman, Seyed Hossein Yahyazadeh Jabbari, Sanni Yaya, Alex Yeshaneh, Birhanu Wubale Yirdaw, Naohiro Yonemoto, Mustafa Z Younis, Chuanhua Yu, Ismaeel Yunusa, Vesna Zadnik, Mikhail Sergeevich Zastrozhin, Anasthasia Zastrozhina, Zhi-Jiang Zhang, Arash Ziapour, Mohammad Zoladl, Christopher J L Murray, Christina Fitzmaurice, Archie Bleyer, and Nickhill Bhakta.

Developing methods or computational machinery
Lisa M Force, Rixing Xu, Jonathan M Kocarnik, Alyssa Pennini, Weijia Fu, Hiwa Abubaker Ali, Isaac Akinkunmi Adedeji, Tarik Ahmed Rashid, Yusra Ahmed Salih, Saqib Ali, Davood Anvari, Mohammad Reza Atashzar, Alok Atreya, Mohammed A Azab, Ahmed Y Azzam, Setognal Birara, Xiaochen Dai, Ahmad Daryani, Mostafa Dianatinasab, Saeid Doaei, Maysaa El Sayed Zaki, Rasool Haddadi, Mehdi Hosseinzadeh, Mowafa Househ, Amirreza Javadi Mamaghani, Bekalu Getnet Kassa, Sezer Kisa, Adnan

Kisa, Sang-woong Lee, James Leigh, Hassan Magdy Abd El Razek, Muhammed Magdy Abd El Razek, Alaa Makki, Borhan Mansouri, Mokhtar Mohammadi, Shafiu Mohammed, Ali H Mokdad, Mohsen Naghavi, Samata Nepal, Bilcha Oumer, Navid Rabiee, Mohammad Rabiee, Amir Masoud Rahmani, Reza Rawassizadeh, Misganu Teshoma Regasa, Maryam Rezaei, Umar Saeed, Abdallah M Samy, Mohammed Shannawaz, Negussie Boti Sidemo, Seyed Abolfazl Tohidast, Ronny Westerman, Mustafa Z Younis, Fariba Zare, Arash Ziapour, Christopher J L Murray, Christina Fitzmaurice, and Nickhill Bhakta.

Providing critical feedback on methods or results

Elysia M Alvarez, Lisa M Force, Jonathan M Kocarnik, Frances E Dean, Weijia Fu, Theresa H M Keegan, Hany Ariffin, Ronald D Barr, D Sanjeeva Gunasekera, Yetunde O John-Akinola, Tyler G Ketterl, Tezer Kutluk, Marcio Henrique Malogolowkin, Prashant Mathur, Venkatraman Radhakrishnan, Lynn Ann Gloeckler Ries, Behzad Abbasi, Hedayat Abbastabar, Michael Abdelmasseh, Sherief Abd-Elsalam, Haimanot Abebe, Aidin Abedi, Hassan Abidi, Hassan Abolhassani, Hiwa Abubaker Ali, Eman Abu-Gharbieh, Basavaprabhu Achappa, Juan Manuel Acuna, Isaac Akinkunmi Adedeji, Oyelola A Adegboye, Qorinah Estiningtyas Sakilah Adnani, Shailesh M Advani, Muhammad Sohail Afzal, Mohamad Aghaie Meybodi, Bahman Ahadinezhad, Bright Opoku Ahinkorah, Sajjad Ahmad, Muktar Beshir Ahmed, Tarik Ahmed Rashid, Yusra Ahmed Salih, Wajeeha Aiman, Gizachew Taddesse Akalu, Fares Alahdab, Abdulhadi A AlAmodi, Fahad Mashhour Alanezi, Turki M Alanzi, Adugnaw Zeleke Alem, Dejene Tsegaye Alem, Yosef Alemayehu, Fadwa Naji Alhalaiqa, Hanadi Al Hamad, Robert Kaba Alhassan, Saqib Ali, Gianfranco Alicandro, Vahid Alipour, Syed Mohamed Aljunid, Motasem Alkhayyat, Sadeq Ali Al-Maweri, Sami Almustanyir, Rajaa M Al-Raddadi, Nelson Alvis-Guzman, Edward Kwabena Ameyaw, Saeed Amini, Hubert Amu, Robert Ancuceanu, Tudorel Andrei, Catalina Liliana Andrei, Fereshteh Ansari, Alireza Ansari-Moghaddam, Davood Anvari, Jalal Arabloo, Morteza Arab-Zozani, Muhammad Arshad, Judie Arulappan, Armin Aryannejad, Zatollah Asemi, Mohammad Asghari Jafarabadi, Mohammad Reza Atashzar, Prince Atorkey, Alok Atreya, Sameh Attia, Avinash Aujayeb, Marcel Ausloos, Leticia Avila-Burgos, Atalel Fentahun Awedew, Beatriz Paulina Ayala Quintanilla, Alemu Degu Ayele, Solomon Shitu Ayen, Mohammed A Azab, Sina Azadnajafabad, Hiva Azami, Mohammadreza Azangou-Khyavy, Ghasem Azarian, Amirhossein Azari Jafari, Ahmed Y Azzam, Atif Amin Baig, Jennifer L Baker, Maciej Banach, Till Winfried Bärnighausen, Fabio Barra, Amadou Barrow, Huda Basaleem, Abdul-Monim Mohammad Batiha, Masoud Behzadifar, Niguss Cherie Bekele, Rebuma Belete, Uzma Iqbal Belgaumi, Arielle Wilder Bell, Alemshet Yirga Berhie, Devidas S Bhagat, Akshaya Srikanth Bhagavathula, Nikha Bhardwaj, Pankaj Bhardwaj, Sonu Bhaskar, Krittika Bhattacharyya, Vijayalakshmi S Bhojaraja, Sadia Bibi, Ali Bijani, Antonio Biondi, Setognal Birara, Obasanjo Afolabi Bolarinwa, Srinivasa Rao Bolla, Archith Boloor, Dejana Braithwaite, Hermann Brenner, Norma B Bulamu, Katrin Burkart, Maria Teresa Bustamante-Teixeira, Zahid A Butt, Nadeem Shafique Butt, Florentino Luciano Caetano dos Santos, Yin Cao, Chao Cao, Ferrán Catalá-López, Francieli Cembranel, Ester Cerin, Raja Chandra Chakinala, Promit Ananyo Chakraborty, Vijay Kumar Chattu, Akhilanand Chaurasia, Odgerel Chimed-Ochir, Jee-Young Jasmine Choi, Devasahayam J Christopher, Dinh-Toi Chu, Michael T Chung, Joao Conde, Vera Marisa Costa, Emanuele D'Amico, Omar B Da'ar, Omid Dadras, Saad M A Dahlawi, Xiaochen Dai, Giovanni Damiani, Lalit Dandona, Rakhi Dandona, Amira Hamed Darwish, Ahmad Daryani, Sisay Abebe Debela, Fernando Pio De la Hoz, Takele Gezahegn G Demie, Zeleke Geto Demissie, Getu Debalkie Demissie, Edgar Denova-Gutiérrez, Meseret Derbew Molla, Rupak Desai, Abebaw Alemayehu Desta, Deepak Dhamnetiya, Samath Dhamminda Dharmaratne, Meghnath Dhimal, Mandira Lamichhane Dhimal, Mostafa Dianatinasab, Mojtaba Didehdar, Mengistie Diress, Shirin Djalalinia, Huyen Phuc Do, Saeid Doaei, Wendel Mombaque dos Santos, Thomas M Drake, Michael Ekholuenetale, Hassan El-Abid, Hala Rashad Elhabashy, Muhammed Elhadi, Shaimaa I El-Jaafary, Iman El Sayed, Maysaa El Sayed Zaki, Daniel Berhanie Enyew, Babak Eshrati, Sharareh Eskandarieh, Mohammed Faisaluddin, Jawad Fares, Umar Farooque, Abidemi Omolara Fasanmi, Wafa Fatima, José Miguel P Ferreira de Oliveira, Simone Ferrero, Lorenzo Ferro Desideri, Getahun Fetensa, Irina Filip, Florian Fischer, James L Fisher, Masoud Foroutan, Takeshi Fukumoto, Peter Andras Gaal,

Mohamed M Gad, Piyada Gaewkhiew, Tushar Garg, Teferi Gebru Gebremeskel, Belete Negese Belete Gemeda, Tamiru Getachew, Mansour Ghafourifard, Ahmad Ghashghaee, Fariba Ghassemi, Nermin Ghith, Ali Gholami, Jamshid Gholizadeh Navashenaq, Themba G Ginindza, Abraham Tamirat Gizaw, James C Glasbey, Amit Goel, Mahaveer Golechha, Davide Golinelli, Sameer Vali Gopalani, Giuseppe Gorini, Houman Goudarzi, Bárbara Niegia Garcia Goulart, Ayman Grada, Maximiliano Ribeiro Guerra, Avirup Guha, Bhawna Gupta, Vivek Kumar Gupta, Veer Bala Gupta, Sapna Gupta, Rasool Haddadi, Nima Hafezi-Nejad, Alemayehu Hailu, Rabih Halwani, Randah R Hamadeh, Mitiku Teshome Hambisa, Samer Hamidi, Shafiul Haque, Sanam Hariri, Ahmed I Hasaballah, S M Mahmudul Hasan, Seyedeh Melika Hashemi, Treska S Hassan, Soheil Hassanipour, Simon I Hay, Sultan H Hebo, Golnaz Heidari, Brenda Yuliana Herrera-Serna, Claudiu Herteliu, Demisu Zenbaba Heyi, Kamal Hezam, Ramesh Holla, Nobuyuki Horita, Md Mahbub Hossain, Mohammad Bellal Hossain, Mohammad-Salar Hosseini, Mostafa Hosseini, Mehdi Hosseinzadeh, Ali Hosseinzadeh, Mihaela Hostiuc, Sorin Hostiuc, Mowafa Househ, Mohamed Hsairi, Nawfal R Hussein, Bing-Fang Hwang, Segun Emmanuel Ibitoye, Olayinka Stephen Ilesanmi, Irena M Ilic, Milena D Ilic, Kaire Innos, Lalu Muhammad Irham, Sheikh Mohammed Shariful Islam, Rakibul M Islam, Nahlah Elkudssiah Ismail, Gaetano Isola, Masao Iwagami, Louis Jacob, Farhad Jadidi-Niaragh, Vardhmaan Jain, Mihajlo Jakovljevic, Roksana Janghorban, Amirreza Javadi Mamaghani, Shubha Jayaram, Ranil Jayawardena, Seyed Behzad Jazayeri, Rime Jebai, Ravi Prakash Jha, Tamas Joo, Nitin Joseph, Farahnaz Joukar, Mikk Jürisson, Billingsley Kaambwa, Ali Kabir, Leila R Kalankesh, Feroze Kaliyadan, Ashwin Kamath, Himal Kandel, Sitanshu Sekhar Kar, Ibraheem M Karaye, Amirali Karimi, Bekalu Getnet Kassa, Joonas H Kauppila, Phillip M Kemp Bohan, Amene Abebe Kerbo, Mohammad Keykhaei, Yousef Saleh Khader, Himanshu Khajuria, Moien AB Khan, Md Nuruzzaman Khan, Maseer Khan, Ejaz Ahmad Khan, Javad Khanali, Maryam Khayamzadeh, Omid Khosravizadeh, Jagdish Khubchandani, Roba Khundkar, Min Seo Kim, Yun Jin Kim, Sezer Kisa, Adnan Kisa, Katarzyna Kissimova-Skarbek, Jacek A Kopec, Rajasekaran Koteeswaran, Sindhura Lakshmi Koulmane Laxminarayana, Ai Koyanagi, Nuworza Kugbey, G Anil Kumar, Nithin Kumar, Alexander Kwarteng, Qing Lan, Savita Lasrado, Paolo Lauriola, Carlo La Vecchia, Caterina Ledda, Yo Han Lee, Wei-Chen Lee, Yeong Yeh Lee, Sang-woong Lee, James Leigh, Elvynna Leong, Ming-Chieh Li, Bingyu Li, Jiarui Li, Stephen S Lim, Xuefeng Liu, Joana A Loureiro, Alessandra Lugo, Hassan Magdy Abd El Razek, Muhammed Magdy Abd El Razek, Morteza Mahmoudi, Azeem Majeed, Alaa Makki, Mohammad-Reza Malekpour, Reza Malekzadeh, Ahmad Azam Malik, Mohammed A Mamun, Navid Manafi, Fariborz Mansour-Ghanaei, Borhan Mansouri, Mohammad Ali Mansournia, Santi Martini, Seyedeh Zahra Masoumi, Clara N Matei, Manu Raj Mathur, Colm McAlinden, Walter Mendoza, Ritesh G Menezes, Alexios-Fotios A Mentis, Tuomo J Meretoja, Amanual Getnet Mersha, Mohamed Kamal Mesregah, Tomislav Mestrovic, Junmei Miao Jonasson, Irmina Maria Michalek, Ted R Miller, Alemu Basazin Mingude, Seyyedmohammadsadeq Mirmoeeni, Hamed Mirzaei, Sanjeev Misra, Prasanna Mithra, Mokhtar Mohammadi, Abdollah Mohammadian-Hafshejani, Shafiu Mohammed, Teroj Abdulrahman Mohammed, Arif Mohammed, Nagabhishek Moka, Ali H Mokdad, Mariam Molokhia, Sara Momtazmanesh, Mohammad Ali Moni, Yousef Moradi, Ghobad Moradi, Maliheh Moradzadeh, Rahmatollah Moradzadeh, Ebrahim Mostafavi, Amin Mousavi Khaneghah, Christine Mpundu-Kaambwa, Sumaira Mubarik, Lillian Mwanri, Ashraf F Nabhan, Shankar Prasad Nagaraju, Mohsen Naghavi, Mukhammad David Naimzada, Vinay Nangia, Atta Abbas Naqvi, Sreenivas Narasimha Swamy, Aparna Ichalangod Narayana, Biswa Prakash Nayak, Javad Nazari, Ionut Negoi, Serban Mircea Negru, Seyed Aria Nejadghaderi, Samata Nepal, Sandhya Neupane Kandel, Cuong Tat Nguyen, Chukwudi A Nnaji, Ali Nowroozi, Vincent Ebuka Nwatah, Chimezie Igwegbe Nzoputam, Bogdan Oancea, Oluwakemi Ololade Odukoya, Ayodipupo Sikiru Oguntade, In-Hwan Oh, Andrew T Olagunju, Tinuke O Olagunju, Babayemi Oluwaseun Olakunde, Mojisola Morenike Oluwasanu, Emad Omar, Ahmed Omar Bali, Sokking Ong, Obinna E Onwujekwe, Doris V Ortega-Altamirano, Nikita Otstavnov, Stanislav S Otstavnov, Bilcha Oumer, Mayowa O Owolabi, Mahesh P A, Alicia Padron-Monedero, Jagadish Rao Padubidri, Adrian Pana, Anamika Pandey, Shahina Pardhan, Fatemeh Pashazadeh Kan, Maja Pasovic, Jenil R Patel, Siddhartha Pati, Sanjay M Pattanshetty, Uttam Paudel, Renato B Pereira, Mario F P Peres, Arokiasamy Perianayagam, Maarten J Postma, Hadi Pourjafar, Akram Pourshams, Akila Prashant, Mirza Muhammad

Fahd Fahd Qadir, Navid Rabiee, Mohammad Rabiee, Amir Radfar, Raghu Anekal Radhakrishnan, Ata Rafiee, Sima Rafiei, Alireza Rafiei, Fakher Rahim, Shadi Rahimzadeh, Muhammad Aziz Rahman, Mosiur Rahman, Amir Masoud Rahmani, Aashish Rajesh, Vajiheh Ramezani-Doroh, Kamal Ranabhat, Sowmya J Rao, Chythra R Rao, Sina Rashedi, Mohammad-Mahdi Rashidi, Mahsa Rashidi, Salman Rawaf, David Laith Rawaf, Lal Rawal, Reza Rawassizadeh, Mohammad Sadegh Razeghinia, Misganu Teshoma Regasa, Andre M N Renzaho, Nima Rezaei, Maryam Rezaei, Negar Rezaei, Mohsen Rezaeian, Aziz Rezapour, Sahba Rezazadeh-Khadem, Abanoub Riad, Ligia Estefania Rios Lopez, Jefferson Antonio Buendia Rodriguez, Godfrey M Rwegerera, Maha Mohamed Saber-Ayad, Siamak Sabour, Basema Saddik, Erfan Sadeghi, Saeid Sadeghian, Umar Saeed, KM Saif-Ur-Rahman, S Mohammad Sajadi, Sana Salehi, Marwa Rashad Salem, Hamideh Salimzadeh, Abdallah M Samy, Juan Sanabria, Francesco Sanmarchi, Arash Sarveazad, Brijesh Sathian, Monika Sawhney, Mete Saylan, Ione Jayce Ceola Schneider, Abdul-Aziz Seidu, Mario Šekerija, Endalew Gemechu Sendo, Sadaf G Sepanlou, Allen Seylani, Kenbon Seyoum, Omid Shafaat, Masood Ali Shaikh, Erfan Shamsoddin, Mohammed Shannawaz, Rajesh Sharma, Sara Sheikhbahaei, Pavanchand H Shetty, Jae Il Shin, Reza Shirkoohi, K M Shivakumar, Parnian Shobeiri, Soraya Siabani, Migbar Mekonnen Sibhat, Negussie Boti Sidemo, Diego Augusto Santos Silva, Guilherme Silva Julian, Jasvinder A Singh, Jitendra Kumar Singh, Achintya Dinesh Singh, Abiy H Sinke, Yitagesu Sintayehu, Valentin Yurievich Skryabin, Anna Aleksandrovna Skryabina, Lee Smith, Ahmad Sofi-Mahmudi, Suhang Song, Paschalis Steiropoulos, Ranjeeta Subedi, Mu'awiyyah Babale Sufiyan, Rizwan Suliankatchi Abdulkader, Saima Sultana, Viktória Szerencsés, Miklós Szócska, Rafael Tabarés-Seisdedos, Mohammadreza Tabary, Takahiro Tabuchi, Hooman Tadbiri, Ken Takahashi, Mircea Tampa, Ker-Kan Tan, Ahmad Tavakoli, Abdelghani Tbakhi, Mohamad-Hani Temsah, Fisaha Haile Tesfay, Bekele Tesfaye, Rekha Thapar, Aravind Thavamani, Arulmani Thiyagarajan, Nihal Thomas, Ruoyan Tobe-Gai, Seyed Abolfazl Tohidast, Hamid Reza Tohidinik, Musliu Adetola Tolani, Daniel Nigusse Tollosa, Mathilde Touvier, Marcos Roberto Tovani-Palone, Eugenio Traini, Mai Thi Ngoc Tran, Bach Xuan Tran, Jaya Prasad Tripathy, Biruk Shalmeno Tusa, Gebresilasea Gendisha Ukke, Irfan Ullah, Saif Ullah, Krishna Kishore Umapathi, Bhaskaran Unnikrishnan, Era Upadhyay, Tolassa Wakayo Ushula, Marco Vacante, Sahel Valadan Tahbaz, Shoban Babu Varthya, Massimiliano Veroux, Giang Thu Vu, Yasir Waheed, Paul Ward, Adisu Birhanu Weldesenbet, Yi Feng Wen, Ronny Westerman, Andrea Sylvia Winkler, Befikadu Legesse Wubishet, Suowen Xu, Seyed Hossein Yahyazadeh Jabbari, Lin Yang, Taklo Simeneh Yazie, Sisay Shewasinad Yehualashet, Alex Yeshaneh, Yigizie Yeshaw, Naohiro Yonemoto, Mustafa Z Younis, Chuanhua Yu, Ismaeel Yunusa, Vesna Zadnik, Mazyar Zahir, Telma Zahirian Moghadam, Mohammad Zamani, Maryam Zamanian, Hamed Zandian, Fariba Zare, Mikhail Sergeevich Zastrozhin, Anasthasia Zastrozhina, Zhi-Jiang Zhang, Jianrong Zhang, Arash Ziapour, Mohammad Zoladl, Christopher J L Murray, Christina Fitzmaurice, Archie Bleyer, and Nickhill Bhakta.

Drafting the work or revising it critically for important intellectual content
Elysia M Alvarez, Lisa M Force, Kelly Compton, Jonathan M Kocarnik, Theresa H M Keegan, Hany Ariffin, Ronald D Barr, D Sanjeeva Gunasekera, Yetunde O John-Akinola, Tyler G Ketterl, Tezer Kutluk, Marcio Henrique Malogolowkin, Venkatraman Radhakrishnan, Lynn Ann Gloeckler Ries, Iyad Sultan, Behzad Abbasi, Zeinab Abbasi-Kangevari, Mohsen Abbasi-Kangevari, Hedayat Abbastabar, Michael Abdelmasseh, Sherief Abd-Elsalam, Aidin Abedi, Hassan Abidi, Hassan Abolhassani, Eman Abu-Gharbieh, Juan Manuel Acuna, Isaac Akinkunmi Adedeji, Oyelola A Adegboye, Qorinah Estiningtyas Sakilah Adnani, Shailesh M Advani, Muhammad Sohail Afzal, Bright Opoku Ahinkorah, Sepideh Ahmadi, Muktar Beshir Ahmed, Wajeeha Aiman, Gizachew Taddesse Akalu, Fares Alahdab, Abdulhadi A AlAmodi, Adugnaw Zeleke Alem, Dejene Tsegaye Alem, Robert Kaba Alhassan, Gianfranco Alicandro, Motasem Alkhayyat, Sunitha Alluri, Nihad A Almasri, Sadeq Ali Al-Maweri, Sami Almustanyir, Nelson Alvis-Guzman, Saeed Amini, Hubert Amu, Robert Ancuceanu, Anayochukwu Edward Anyasodor, Jalal Arabloo, Morteza Arab-Zozani, Muhammad Arshad, Judie Arulappan, Mohammad Reza Atashzar, Prince Atorkey, Alok Atreya, Sameh Attia, Avinash Aujayeb, Marcel

Ausloos, Atalel Fentahun Awedew, Beatriz Paulina Ayala Quintanilla, Alemu Degu Ayele, Solomon Shitu Ayen, Mohammed A Azab, Sina Azadnajafabad, Mohammadreza Azangou-Khyavy, Amirhossein Azari Jafari, Ahmed Y Azzam, Saeed Bahadory, Jianjun Bai, Atif Amin Baig, Jennifer L Baker, Maciej Banach, Till Winfried Bärnighausen, Francesco Barone-Adesi, Fabio Barra, Amadou Barrow, Masoud Behzadifar, Rebuma Belete, Uzma Iqbal Belgaumi, Arielle Wilder Bell, Devidas S Bhagat, Akshaya Srikanth Bhagavathula, Sonu Bhaskar, Krittika Bhattacharyya, Vijayalakshmi S Bhojaraja, Sadia Bibi, Antonio Biondi, Tone Bjørge, Dejana Braithwaite, Hermann Brenner, Norma B Bulamu, Maria Teresa Bustamante-Teixeira, Nadeem Shafique Butt, Florentino Luciano Caetano dos Santos, Yin Cao, Chao Cao, Giulia Carreras, Ferrán Catalá-López, Francieli Cembranel, Ester Cerin, Promit Ananyo Chakraborty, Vijay Kumar Chattu, Prachi P Chavan, Dinh-Toi Chu, Michael T Chung, Joao Conde, Vera Marisa Costa, Emanuele D'Amico, Omar B Da'ar, Giovanni Damiani, Parnaz Daneshpajouhnejad, Ahmad Daryani, Fernando Pio De la Hoz, Takele Gezahegn G Demie, Zeleke Geto Demissie, Getu Debalkie Demissie, Edgar Denova-Gutiérrez, Meseret Derbew Molla, Rupak Desai, Deepak Dhamnetiya, Samath Dhamminda Dharmaratne, Meghnath Dhimal, Mandira Lamichhane Dhimal, Mojtaba Didehdar, Mengistie Diress, Huyen Phuc Do, Saeid Doaei, Wendel Mombaque dos Santos, Thomas M Drake, Hassan El-Abid, Mostafa Ahmed Elbahnasawy, Iffat Elbarazi, Hala Rashad Elhabashy, Muhammed Elhadi, Shaimaa I El-Jaafary, Iman El Sayed, Maysaa El Sayed Zaki, Maha El Tantawi, Daniel Berhanie Enyew, Ryenchindorj Erkhembayar, Sharareh Eskandarieh, Mohammed Faisaluddin, Jawad Fares, José Miguel P Ferreira de Oliveira, Simone Ferrero, Lorenzo Ferro Desideri, Irina Filip, Florian Fischer, James L Fisher, Masoud Foroutan, Takeshi Fukumoto, Mohamed M Gad, Piyada Gaewkhiew, Silvano Gallus, Tushar Garg, Teferi Gebru Gebremeskel, Mansour Ghafourifard, Seyyed-Hadi Ghamari, Ahmad Ghashghaee, Fariba Ghassemi, Nermin Ghith, Jamshid Gholizadeh Navashenaq, Syed Amir Gilani, Abraham Tamirat Gizaw, James C Glasbey, Amit Goel, Davide Golinelli, Sameer Vali Gopalani, Giuseppe Gorini, Houman Goudarzi, Bárbara Niegia Garcia Goulart, Ayman Grada, Mohammed Ibrahim Mohialdeen Gubari, Maximiliano Ribeiro Guerra, Bhawna Gupta, Vivek Kumar Gupta, Veer Bala Gupta, Sapna Gupta, Nima Hafezi-Nejad, Alemayehu Hailu, Rabih Halwani, Randah R Hamadeh, Mitiku Teshome Hambisa, Sajid Hameed, Samer Hamidi, Shafiul Haque, Sanam Hariri, Josep Maria Haro, Ahmed I Hasaballah, Treska S Hassan, Simon I Hay, Khezar Hayat, Golnaz Heidari, Claudiu Herteliu, Demisu Zenbaba Heyi, Kamal Hezam, Michael K Hole, Ramesh Holla, Nobuyuki Horita, Md Mahbub Hossain, Mohammad Bellal Hossain, Mohammad-Salar Hosseini, Mostafa Hosseini, Mihaela Hostiuc, Sorin Hostiuc, Mowafa Househ, Junjie Huang, Nawfal R Hussein, Segun Emmanuel Ibitoye, Olayinka Stephen Ilesanmi, Irena M Ilic, Milena D Ilic, Kaire Innos, Lalu Muhammad Irham, Sheikh Mohammed Shariful Islam, Rakibul M Islam, Nahlah Elkudssiah Ismail, Gaetano Isola, Louis Jacob, Vardhmaan Jain, Mihajlo Jakovljevic, Roksana Janghorban, Amirreza Javadi Mamaghani, Shubha Jayaram, Rime Jebai, Ravi Prakash Jha, Nitin Joseph, Mikk Jürisson, Billingsley Kaambwa, Ali Kabir, Leila R Kalankesh, Feroze Kaliyadan, Ashwin Kamath, Sitanshu Sekhar Kar, Bekalu Getnet Kassa, Joonas H Kauppila, Andre Pascal Kengne, Yousef Saleh Khader, Himanshu Khajuria, Neda Khalili, Nastaran Khalili, Moien AB Khan, Md Nuruzzaman Khan, Maseer Khan, Ejaz Ahmad Khan, Gulfaraz Khan, Javad Khanali, Jagdish Khubchandani, Yun Jin Kim, Sezer Kisa, Adnan Kisa, Sindhura Lakshmi Koulmane Laxminarayana, Ai Koyanagi, Nuworza Kugbey, Alexander Kwarteng, Iván Landires, Savita Lasrado, Carlo La Vecchia, Yeong Yeh Lee, Elvynna Leong, Ming-Chieh Li, Stephen S Lim, Joana A Loureiro, Raimundas Lunevicius, Hassan Magdy Abd El Razek, Muhammed Magdy Abd El Razek, Shilpa Male, Mohammad-Reza Malekpour, Ahmad Azam Malik, Navid Manafi, Borhan Mansouri, Clara N Matei, Colm McAlinden, Ravi Mehrotra, Walter Mendoza, Ritesh G Menezes, Alexios-Fotios A Mentis, Tuomo J Meretoja, Mohamed Kamal Mesregah, Tomislav Mestrovic, Junmei Miao Jonasson, Irmina Maria Michalek, Ted R Miller, Seyyedmohammadsadeq Mirmoeeni, Prasanna Mithra, Karzan Abdulmuhsin Mohammad, Seyyede Momeneh Mohammadi, Abdollah Mohammadian-Hafshejani, Shafiu Mohammed, Teroj Abdulrahman Mohammed, Arif Mohammed, Nagabhishek Moka, Ali H Mokdad, Mariam Molokhia, Sara Momtazmanesh, Lorenzo Monasta, Mohammad Ali Moni, Paula Moraga, Shane Douglas Morrison, Amin Mousavi Khaneghah, Lillian Mwanri, Ashraf F Nabhan, Shankar Prasad Nagaraju, Chie Nagata, Mohsen Naghavi, Mukhammad David Naimzada, Sreenivas Narasimha Swamy, Biswa Prakash

Nayak, Vinod C Nayak, Javad Nazari, Sabina Onyinye Nduaguba, Ionut Negoi, Serban Mircea Negru, Seyed Aria Nejadghaderi, Samata Nepal, Cuong Tat Nguyen, Virginia Nuñez-Samudio, Vincent Ebuka Nwatah, Chimezie Igwegbe Nzoputam, Bogdan Oancea, Oluwakemi Ololade Odukoya, Andrew T Olagunju, Tinuke O Olagunju, Mojisola Morenike Oluwasanu, Emad Omar, Obinna E Onwujekwe, Doris V Ortega-Altamirano, Nikita Otstavnov, Stanislav S Otstavnov, Bilcha Oumer, Mayowa O Owolabi, Mahesh P A, Alicia Padron-Monedero, Jagadish Rao Padubidri, Shahina Pardhan, Fatemeh Pashazadeh Kan, Siddhartha Pati, Uttam Paudel, Renato B Pereira, Mario F P Peres, Arokiasamy Perianayagam, Maarten J Postma, Akila Prashant, Thejodhar Pulakunta, Mirza Muhammad Fahd Fahd Qadir, Navid Rabiee, Mohammad Rabiee, Amir Radfar, Raghu Anekal Radhakrishnan, Ata Rafiee, Alireza Rafiei, Fakher Rahim, Muhammad Aziz Rahman, Priyanga Ranasinghe, Sowmya J Rao, Chythra R Rao, Mohammad-Mahdi Rashidi, Mahsa Rashidi, Salman Rawaf, David Laith Rawaf, Lal Rawal, Mohammad Sadegh Razeghinia, Misganu Teshoma Regasa, Andre M N Renzaho, Nima Rezaei, Maryam Rezaei, Sahba Rezazadeh-Khadem, Jefferson Antonio Buendia Rodriguez, Luca Ronfani, Gholamreza Roshandel, Godfrey M Rwegerera, Maha Mohamed Saber-Ayad, Siamak Sabour, Basema Saddik, Umar Saeed, Amirhossein Sahebkar, KM Saif-Ur-Rahman, Sarvenaz Salahi, Sana Salehi, Marwa Rashad Salem, Hamideh Salimzadeh, Abdallah M Samy, Juan Sanabria, Francesco Sanmarchi, Susan M Sawyer, Mete Saylan, Ione Jayce Ceola Schneider, Mario Šekerija, Allen Seylani, Erfan Shamsoddin, Mohammed Shannawaz, Sara Sheikhbahaei, K M Shivakumar, Parnian Shobeiri, Sudeep K Siddappa Malleshappa, Negussie Boti Sidemo, Diego Augusto Santos Silva, Guilherme Silva Julian, Jasvinder A Singh, Surjit Singh, Achintya Dinesh Singh, Lee Smith, Ahmad Sofi-Mahmudi, Mohammad Sadegh Soltani-Zangbar, Paschalis Steiropoulos, Kurt Straif, Ranjeeta Subedi, Mu'awiyyah Babale Sufiyan, Saima Sultana, Seidamir Pasha Tabaeian, Mohammadreza Tabary, Takahiro Tabuchi, Hooman Tadbiri, Majid Taheri, Mircea Tampa, Ker-Kan Tan, Vivian Y Tat, Arash Tehrani-Banihashemi, Mohamad-Hani Temsah, Fisaha Haile Tesfay, Bekele Tesfaye, Jarnail Singh Thakur, Aravind Thavamani, Arulmani Thiyagarajan, Nihal Thomas, Munkhsaikhan Togtmol, Seyed Abolfazl Tohidast, Hamid Reza Tohidinik, Musliu Adetola Tolani, Mathilde Touvier, Marcos Roberto Tovani-Palone, Bach Xuan Tran, Jaya Prasad Tripathy, Biruk Shalmeno Tusa, Gebresilasea Gendisha Ukke, Irfan Ullah, Saif Ullah, Krishna Kishore Umapathi, Bhaskaran Unnikrishnan, Era Upadhyay, Tolassa Wakayo Ushula, Marco Vacante, Sahel Valadan Tahbaz, Shoban Babu Varthya, Massimiliano Veroux, Paul J Villeneuve, Francesco S Violante, Vasily Vlassov, Giang Thu Vu, Yasir Waheed, Ning Wang, Paul Ward, Adisu Birhanu Weldesenbet, Ronny Westerman, Andrea Sylvia Winkler, Seyed Hossein Yahyazadeh Jabbari, Lin Yang, Sanni Yaya, Vahid Yazdi-Feyzabadi, Sisay Shewasinad Yehualashet, Yigizie Yeshaw, Naohiro Yonemoto, Mustafa Z Younis, Zabihollah Yousefi, Mazyar Zahir, Maryam Zamanian, Mikhail Sergeevich Zastrozhin, Anasthasia Zastrozhina, Jianrong Zhang, Arash Ziapour, Mohammad Zoladl, Christopher J L Murray, Archie Bleyer, and Nickhill Bhakta.

Managing the overall research enterprise
Lisa M Force, Kelly Compton, Lalit Dandona, Simon I Hay, Ali H Mokdad, Mohsen Naghavi, and Christopher J L Murray.
